# Comparative Mitogenome Analyses Uncover Mitogenome Features and Phylogenetic Implications of the Rabbitfishes (Perciformes: Siganidae)

**DOI:** 10.1002/ece3.74188

**Published:** 2026-08-14

**Authors:** Hao‐Ran Ma, Le‐Jia Pang, Qin Tang, Teng Wang

**Affiliations:** ^1^ Key Laboratory of South China Sea Fishery Resources Exploitation and Utilization, Ministry of Agriculture and Rural Affairs, South China Sea Fisheries Research Institute Chinese Academy of Fishery Sciences Guangzhou China; ^2^ College of Fisheries Huazhong Agricultural University Wuhan China; ^3^ Scientific Observation and Research Station of Xisha Island Reef Fishery Ecosystem of Hainan Province Sanya Tropical Fisheries Research Institute Sanya China

**Keywords:** coral reef, genome, mitochondria, Siganidae, Xisha Islands

## Abstract

Mitochondria are essential for energy metabolism in coral reef fish, and mitogenomes represent valuable genetic resources for phylogenetic analysis and species identification. To investigate the lineage variation and phylogenetic relationships, we obtained the first complete mitochondrial genome sequences of four Siganidae species ( *Siganus argenteus* , *S. corallinus* , *S. punctatus* , and *S. spinus* ) and conducted comparative analyses with 10 published mitogenomes from this family. Results revealed that siganid mitogenomes were highly conserved in gene content, organization, and base composition. Phylogenetic analysis based on 13 protein‐coding genes (PCGs) classified the Siganidae family into three major clades, which corresponded to divergent ecological habits and morphological characteristics. The control region of mitogenome exhibited the greatest interspecific variability. Structural variations in tRNAs and the Origin of Light‐Strand Replication (O_*L*_) were largely consistent with the mitochondrial phylogeny inferred from PCGs. Selection pressure analysis indicated that purifying selection dominates all PCGs, with varying selective constraints among genes. Notably, *nad5* showed the most purifying selection sites and *atp8* showed the fewest, suggesting their potential as markers for siganid species identification. All data and findings generated herein constitute a valuable resource for future siganid research, laying a foundation for evolutionary exploration and coral reef biodiversity conservation.

## Graphical abstract

This study reports four newly generated complete mitochondrial genomes of rabbitfishes and compares them with 10 published Siganidae mitogenomes. The results show conserved genome organization and base composition, with interspecific variation mainly in the control region, tRNA structures, and origin of light‐strand replication. The dataset provides a comparative mitogenomic reference for future studies on siganid phylogeny, mitochondrial genome evolution, and species identification.

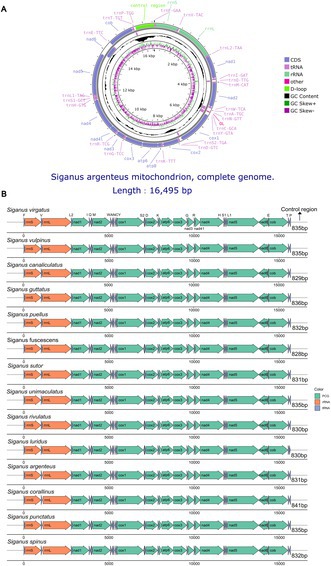

## 1 Introduction

Mitochondrial genomes (mitogenomes) have become widely used molecular markers for investigating animal phylogenetics due to their maternal inheritance, small size, high copy number, rapid evolutionary rate, and low recombination frequency (Boore and Brown 1998; Powell et al. 2020; Ghiselli et al. 2021; Struck et al. 2023). Advancements in sequencing technologies enable researchers to efficiently obtain complete fish mitogenomes, which typically contain 37 genes, including 13 protein‐coding genes (PCGs), 22 transfer RNAs (tRNAs), two ribosomal RNAs (rRNAs), and one D‐loop control region (Boore and Brown 1998; Gissi et al. 2008; Wang et al. 2015; Marnis et al. 2024; Verissimo et al. 2025). Compared to single‐gene fragments, complete mitogenome data can provide higher‐resolution phylogenetic signals and allow more comprehensive analysis of gene arrangements, nucleotide composition biases (e.g., AT/GC‐skew), codon usage patterns, and non‐coding RNA features, thereby contributing to comparative studies of mitochondrial genome evolution and phylogenetic relationships among species (Li et al. 2022; Liao et al. 2024). Satoh et al. (2016) systematically compared the mitogenomes of 250 actinopterygian fishes, and their quantitative analysis revealed preferences in the initiation/termination codons of PCGs, characterized the secondary structures of tRNAs and rRNAs, identified gene rearrangements, and highlighted the advantages of genome‐scale data for comparative evolutionary analyses (Satoh et al. 2016). Yu et al. (2021) analyzed 58 mitogenomes of Cobitinae loaches and identified the universal absence of the D‐arm secondary structure in tRNA‐Ser, a unique 3‐bp insertion (GCA) in the overlapped region of genes *atp8* and *atp6*, and intergeneric nucleotide composition biases (e.g., a negative correlation between AT‐skew and A + T content), which suggest the occurrence of cross‐generic mitochondrial introgression events and support the hypothesis that Cobitinae diverged into northern and southern clades in the late Eocene (Yu et al. 2021). Besides, Alvarenga et al. (2024) focused on fisheries management by sequencing the mitogenomes of 70 South Atlantic commercial fishes (e.g., mackerel, sardines), and found conserved gene order in 95.7% of the species while identifying rearrangements in three species, thereby providing valuable molecular markers applicable to population identification, trade monitoring of endangered species, and sustainable resource utilization (Alvarenga et al. 2024).

The Siganidae is a monotypic family within the order Perciformes, containing only the genus *Siganus* with 29 extant species, which is widely distributed across Indo‐Pacific tropical waters, inhabiting estuaries, mangroves, algal beds, and coral reefs (Zolkaply et al. 2021). As an important herbivore in coral reef and seagrass ecosystems, siganids maintain reef structural and functional stability by continuously grazing on algae, preventing algal overgrowth that competitively excludes coral larvae, thereby directly securing substrate attachment space for coral recruitment (Hughes et al. 2007). However, severe water pollution and habitat degradation have posed a serious threat to wild populations (Baum et al. 2016). Some specific species of the Siganidae family, such as the goldspotted spinefoot ( *Siganus guttatus* ), are important for aquaculture and stock enhancement, as they contribute to water quality improvement and reduction of net‐fouling maintenance costs (Zhang et al. 2024). Thus, investigating the mitogenomic characteristics and phylogenetic relationships of Siganidae species is of great significance. Previous phylogenetic studies on this family predominantly relied on limited mitochondrial gene fragments. For instance, Borsa et al. (2007) reconstructed siganid divergence patterns using the *cytb* and 16S rRNA genes (Borsa et al. 2007), Alyamani (2024) assessed interspecific relationships also based on 16S rRNA genes (Alyamani 2024), and Zolkaply et al. (2021) combined mitochondrial COI (cytochrome oxidase I) and nuclear RHO (rhodopsin retrogene) markers to reassess siganid taxonomy, resolve molecular relationships, and infer lineage‐divergence timing. Comparative mitogenomic analyses are also limited in Siganidae. Full mitogenome data remain inadequate for broad cross‐species analyses due to incomplete taxonomic sampling. This has constrained a more comprehensive assessment of mitogenome conservation, lineage‐associated variation, and mitochondrial phylogenetic patterns within the family. To address this gap, we generated complete mitochondrial genome sequences for four *Siganus* species for which such data were previously unavailable and compared them with 10 published siganid mitogenomes.

Here, four newly reported complete *Siganus* mitogenomes were analyzed alongside 10 available siganid mitogenomes, providing 14 mitogenomes for family‐level comparison. This broader mitogenome sampling allowed assessment of conservation and variation in genome organization, gene arrangement, nucleotide composition, codon usage, and structural features of non‐coding RNAs across Siganidae. We further inferred the Siganidae mitochondrial phylogeny from the 13 PCGs and assessed gene‐specific selective constraints across mitochondrial coding regions. These data expand mitogenomic resources for Siganidae and provide a comparative basis for future phylogenetic, taxonomic, and species‐identification studies of rabbitfishes.

## 2 Materials and Methods

### 2.1 Sample Collection, DNA Extraction, Library Construction, and Sequencing

Specimens representing four *Siganus* species, belonging to the family Siganidae (order Perciformes, class Actinopterygii), were originally collected from the waters of Xisha Islands, China (15°46′–17°08′ N, 111°11′–112°54′ E) between 2020 and 2021, with the assistance of local fishermen using SCUBA‐assisted capture. These samples were subsequently used for DNA extraction, library construction, sequencing, and mitogenomic analyses in 2022. The collected species included *Siganus argenteus* (Streamlined spinefoot), *Siganus corallinus* (Blue‐spotted spinefoot), *Siganus punctatus* (Goldspotted spinefoot), and *Siganus spinus* (Little spinefoot). The four mitogenomes obtained here are available in GenBank under the accession numbers NC_086723 ( *S. argenteus* ), PX112429 ( *S. corallinus* ), PX112430 ( *S. punctatus* ), and PX112431 ( *S. spinus* ). Voucher specimens corresponding to these four mitogenomes are archived at the South China Sea Fisheries Research Institute, Chinese Academy of Fishery Sciences, with the following institutional catalog numbers: dd‐ylzy20210420003 for *S. argenteus* , qly‐awlzy20210715001 for *S. corallinus* , qly‐blzy20210120001 for *S. punctatus* , and qly‐rwlzy20200415001 for *S. spinus* , respectively. These catalog numbers correspond to internal sample records maintained by the institutional specimen/tissue repository and can be verified upon reasonable request by contacting the corresponding authors. For comprehensive comparative analysis, the complete mitochondrial genome sequences of 10 additional *Siganus* species were retrieved from the NCBI GenBank database, with their corresponding accession numbers listed as follows: *Siganus virgatus* (Barhead spinefoot, OQ852681.1), *Siganus vulpinus* (Foxface rabbitfish, NC_025588.1), *Siganus canaliculatus* (White‐spotted spinefoot, NC_024881.1), *Siganus guttatus* (Orange‐spotted spinefoot, NC_024088.1), *Siganus puellus* (Masked spinefoot, NC_024086.1), *Siganus fuscescens* (Mottled spinefoot, NC_009572.1), *Siganus sutor* (Shoemaker spinefoot, MG677546.1), *Siganus unimaculatus* (Blotched foxface, NC_013148.1), *Siganus rivulatus* (Marbled spinefoot, MW376910.1), and *Siganus luridus* (Dusky spinefoot, MW376909.1).

Genomic DNA was isolated from muscle tissue of each Siganidae specimen using the E.Z.N.A. Tissue DNA Kit (OMEGA Bio‐tek, Beijing, China), strictly following the kit's standard protocol. A tiny muscle biopsy was collected from each sample prior to extraction for use as source material. After DNA isolation, approximately 1 μg of genomic DNA from each sample underwent random shearing to produce fragments approximately 300–500 bp in length for subsequent library preparation. The resulting fragments were processed for library preparation with the Illumina TruSeq Nano DNA Sample Prep Kit (Illumina, San Diego, CA, USA) following the kit's recommended procedures. Shanghai Biozeron Co. Ltd. completed the library‐preparation workflow and sequenced the libraries on a NovaSeq 6000 system (Illumina), producing 150‐bp paired‐end reads.

### 2.2 Mitochondrial Genome Assembly, Annotation, and Characterization

Before assembly, raw sequencing reads were processed with Trimmomatic (v0.39) (Bolger et al. 2014) to discard low‐quality reads (*Q* < 20), adapter‐contaminated reads, reads containing ≥ 10% ambiguous bases (“N”), and duplicate sequences. After quality filtering, the retained reads were first assembled into contigs with MitoZ (v2.3) (Meng et al. 2019). Candidate mitochondrial contigs were then screened by comparison with the NCBI mitochondrial genome database. Complete mitochondrial genomes were subsequently reconstructed with GetOrganelle (v1.7.5) (https://github.com/Kinggerm/GetOrganelle; accessed on March 20, 2022) (Jin et al. 2020). To standardize sequence orientation and starting position, each newly generated mitogenome was compared with published complete *Siganus* mitogenomes, using the annotated mitogenome of *Siganus guttatus* (GenBank accession no. NC_024088.1) as a representative reference. To ensure high mapping accuracy and uniform read coverage, candidate mitochondrial contigs were also mapped to the reference mitogenome with a query coverage threshold of 80%. In addition, MUMmer (v3.23) (Kurtz et al. 2004) was used to check whether these contigs were circular. Assembly quality metrics, including clean data volume, estimated theoretical depth based on clean data, assembled mitogenome length, and assembly completeness, are listed in Table S1.

The newly assembled mitogenomes were annotated with MITOS (Bernt et al. 2013) and MitoAnnotator (v3.83) (Iwasaki et al. 2013; Zhu et al. 2023), covering the standard mitochondrial gene set, including the 13 protein‐coding genes, 22 transfer RNAs, and 2 ribosomal RNAs. For functional characterization, PCGs were further subjected to BLAST similarity searches against five public databases. These included the NCBI non‐redundant protein database (Nr), Swiss‐Prot, COG (Clusters of Orthologous Groups), KEGG (Kyoto Encyclopedia of Genes and Genomes), and GO (Gene Ontology), with an *E*‐value threshold of 10^−5^. Transfer RNA genes were additionally checked with tRNAscan‐SE (v2.0) (Chan et al. 2021), and putative secondary structures were generated with RNAplot, as implemented in ViennaRNA package (v2.5.1) (Lorenz et al. 2011).

Nucleotide composition and codon‐use patterns were examined with SnapGene (v8.1.1) (http://www.snapgene.com/) and the Sequence Manipulation Suite (v2) (Stothard 2000). Compositional skew was estimated as AT‐skew = (A − T)/(A + T) and GC‐skew = (G − C)/(G + C) (Gao et al. 2023). To estimate relative synonymous codon usage (RSCU), we used both the “cusp” program in EMBOSS suite (v6.6.0.0) (Rice et al. 2000) and CodonW software implemented on the Linux cluster system, with cross‐validation confirming result consistency. Circular mitogenome maps were prepared in the online platform Proksee (https://proksee.ca/; accessed on March 25, 2025) (Grant et al. 2023). Heatmaps were generated using the R package “ComplexHeatmap”. Conserved sequence block (CSB) regions in the control region were determined by aligning the sampled sequences with published Siganidae species.

### 2.3 Phylogenetic Analysis

Phylogenetic relationships were inferred based on the 13 protein‐coding genes (PCGs) from the mitogenomes of 14 Siganidae fish species. For tree rooting, two non‐siganid coral‐reef fishes were selected as outgroups: the dusky parrotfish *Scarus niger* (GenBank accession OQ349187.1) and the Singapore parrotfish *Scarus prasiognathos* (GenBank accession OQ349189.1). Their placement outside Siganidae made them suitable outgroup taxa, while their complete and well‐annotated mitogenomes contained the corresponding 13 PCGs required for reliable alignment with the siganid sequences. Sequence analysis was conducted using CamlTree, a software developed in our previous study (Sun et al. 2025). Within the CamlTree workflow, MAFFT was used under default settings to align the 13 PCG sequences, and trimAl was then applied to remove low‐confidence regions and ambiguous sites. The curated alignments were combined into a single 13‐PCG matrix, which was analyzed using both Maximum Likelihood (ML) and Bayesian Inference (BI) approaches.

For ML inference, CamlTree called IQ‐TREE on the merged 13‐PCG alignment matrix. The run used the following IQ‐TREE settings: ‐s Siganidae_13PCG_concatenated.fa ‐spp partitions.nex ‐m MFP+MERGE ‐bb 1000 bnni ‐o S.niger,S.prasiognathos ‐nt AUTO. In the partition file, each of the 13 PCGs was defined as an initial gene‐level block; codon‐position‐based starting partitions were not applied. ModelFinder was then used with the MFP+MERGE option to optimize substitution models and merge partitions according to the Bayesian Information Criterion (BIC). The final ML partition scheme consisted of three partitions: (i) *atp6* + *atp8* + *cob* + *nad1* + *nad2* + *nad3* + *nad4* + *nad4l* + *nad5* under TIM2 + F + R3, (ii) *cox1* + *cox2* + *cox3* under TPM2u + F + I + G4, and (iii) *nad6* under TNe + R2. Branch support was evaluated using 1000 ultrafast bootstrap (UFBoot) replicates with the ‐bb 1000 option, and bootstrap tree optimization was performed using the ‐bnni option.

Complementary BI analysis was also carried out using CamlTree software, with the BI tree constructed via its built‐in MrBayes module. To ensure data compatibility, the original alignment file was converted to NEXUS format using embedded ALTER with parameters ‐ia ‐of NEXUS ‐op MrBayes. The same three‐partition scheme was used for the BI analysis. Each partition was assigned a GTR + I + G model in MrBayes (nst = 6 rates = invgamma), with state frequencies, substitution rates, gamma shape, and the proportion of invariant sites unlinked across partitions. For the BI analysis, two independent runs with four Markov Chain Monte Carlo (MCMC) chains were performed for 5,000,000 generations, and tree topologies were recorded at 1000‐generations intervals. The initial quarter of the posterior samples was excluded as burn‐in. Chain convergence was assessed by confirming that the average standard deviation of split frequencies dropped below 0.01. Finally, the resulting ML and BI topologies were rendered and formatted with FigTree (v1.4.4) (http://tree.bio.ed.ac.uk/software/figtree/, accessed on June 10, 2025).

### 2.4 Selection Pressure Analysis

To evaluate selection patterns across mitochondrial PCGs, amino acid sequences of each PCG were first aligned with ClustalW (v2.1) (Thompson et al. 1994; Hung and Weng 2016). The protein alignments, together with their corresponding nucleotide coding sequences, were used as input for PAL2NAL (v14) to generate codon‐based alignments (Suyama et al. 2006). The 13 PCGs were analyzed separately to compare gene‐specific differences in selective constraints. For each PCG, codon‐level selection tests were run in HyPhy (v2.5.39) with two complementary approaches, SLAC and FEL. These abbreviations denote Single Likelihood Ancestor Counting and Fixed Effects Likelihood, respectively (Kosakovsky Pond et al. 2020). The use of both SLAC and FEL allowed cross‐validation of site‐level selection patterns under different statistical frameworks. Both methods estimate nonsynonymous and synonymous substitution rates (*d*_N_ and *d*_S_), and the *d*_N_/*d*_S_ ratio (*ω*) was used to assess selective constraints at codon sites. Sites with *ω* < 1 were interpreted as being under purifying selection, whereas sites with *ω* > 1 were considered candidates for diversifying selection only when statistically significant. A threshold of *p* < 0.05 was applied to both SLAC‐ and FEL‐based tests.

## 3 Results

### 3.1 General Characteristics of the Mitogenomes

**FIGURE 1:**
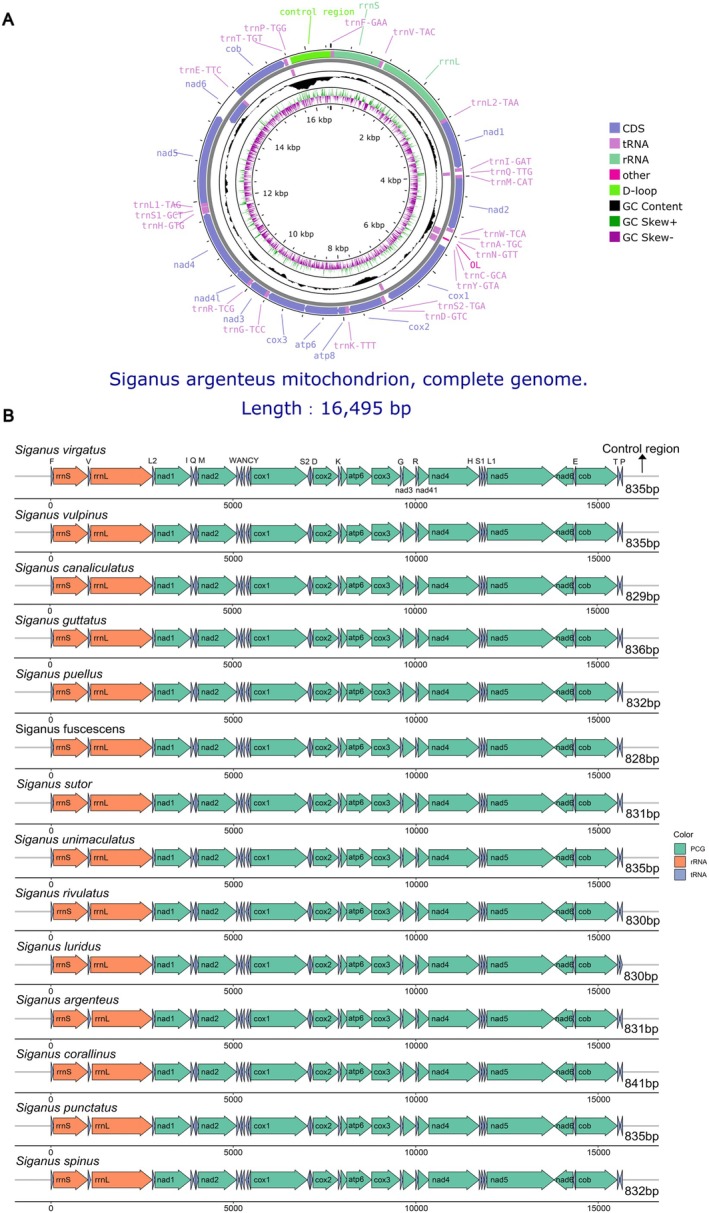
(A) Map of the mitochondrial genome of a Siganidae species, taking *Siganus argenteus* as an example. PCGs are denoted by blue arrows, tRNA genes by lavender arrows, and rRNA genes by light green arrows. tRNA genes are labeled with single‐letter amino acid abbreviations followed by their anticodons. Peaks on the black circle indicate GC content, with outward and inward peaks representing values above or below the average GC content, respectively. Purple and green circles display GC skew, where values ranging from 0 to 1 are shown in purple and those from −1 to 0 in green. Tick marks on the inner circle indicate sequence length. The total sequence length is 16,495 bp. (B) Comparative mitogenomic analysis of gene arrangements across 14 Siganidae species. Gene categories are indicated by different colors, and the length of the control region is shown at the right end of each mitogenome.

The four newly assembled mitogenomes were similar in size, spanning 16,495–16,510 bp, with *S. argenteus* representing the lower end of this range and *S. corallinus* the upper end (Table S1). Each assembly contained the typical vertebrate mitochondrial gene complement: 13 protein‐coding genes, 22 tRNAs, two rRNAs corresponding to *rrnS*/12S rRNA and *rrnL*/16S rRNA, and a control region (Table 1, Figure 1; Table S2, Figure S1). In the representative *S. argenteus* mitogenome, the 13 PCGs together spanned 11,433 bp, corresponding to 69.31% of the 16,495‐bp genome. Strand assignment was consistent with the canonical vertebrate mitogenome pattern. Twelve PCGs were encoded on the H‐strand, whereas *nad6* was the only PCG encoded on the L‐strand and showed the opposite orientation (Table 1, Figure 1). 14 tRNA genes were assigned to the H‐strand and 8 to the L‐strand. Their identities and genomic positions are summarized in Table 1 and Figure 1. This gene arrangement pattern was identical across all examined *Siganus* species within the family Siganidae (Table S2) and the overall arrangement was consistent with the canonical vertebrate mitochondrial organization (Gissi et al. 2008; Wang et al. 2015; Marnis et al. 2024; Verissimo et al. 2025). Genome‐wide comparison across the 14 sampled mitogenomes showed conserved organization and no detectable rearrangement. By contrast, interspecific differences were most evident in the control region, particularly in region length and nucleotide composition (Figure 1B; Figure S2).

**TABLE 1:** Summary of mitochondrial genome information for rabbitfish, using *Siganus argenteus* as an example.

Gene	Start	End	Strand	Size (bp)	Start codon	Stop codon	Anticodons
trnF	1	68	+	68	−	−	GAA
rrnS	69	1014	+	946	−	−	−
trnV	1015	1086	+	72	−	−	TAC
rrnL	1126	2778	+	1653	−	−	−
trnL2	2779	2852	+	74	−	−	TAA
nad1	2853	3827	+	975	ATG	TAA	−
trnI	3832	3901	+	70	−	−	GAT
trnQ	3901	3971	−	71	−	−	TTG
trnM	3971	4039	+	69	−	−	CAT
nad2	4040	5086	+	1047	ATG	TAG	−
trnW	5085	5156	+	72	−	−	TCA
trnA	5157	5225	−	69	−	−	TGC
trnN	5228	5300	−	73	−	−	GTT
trnC	5338	5403	−	66	−	−	GCA
trnY	5404	5474	−	71	−	−	GTA
cox1	5476	7026	+	1551	GTG	TAA	−
trnS2	7029	7099	−	71	−	−	TGA
trnD	7103	7174	+	72	−	−	GTC
cox2	7183	7873	+	691	ATG	T	−
trnK	7874	7948	+	75	−	−	TTT
atp8	7950	8117	+	168	ATG	TAA	−
atp6	8108	8791	+	684	ATG	TAA	−
cox3	8791	9576	+	786	ATG	TAA	−
trnG	9576	9648	+	73	−	−	TCC
nad3	9649	9999	+	351	ATG	TAG	−
trnR	9998	10066	+	69	−	−	TCG
nad4l	10067	10363	+	297	ATG	TAA	−
nad4	10357	11737	+	1381	ATG	T	−
trnH	11738	11806	+	69	−	−	GTG
trnS1	11807	11874	+	68	−	−	GCT
trnL1	11880	11952	+	73	−	−	TAG
nad5	11953	13791	+	1839	ATG	TAA	−
nad6	13788	14309	−	522	ATG	TAG	−
trnE	14310	14378	−	69	−	−	TTC
cob	14383	15523	+	1141	ATG	T	−
trnT	15524	15595	+	72	−	−	TGT
trnP	15595	15664	−	70	−	−	TGG

Across the 14 sampled species, we detected two initiation codons and three termination‐codon forms. For instance, in *S. argenteus* , 12 of the 13 PCGs started with ATG, while *cox1* began with GTG (Table 1). Stop‐codon usage in *S. argenteus* differed among PCGs. TAA was used by *nad1*, *cox1*, *atp8*, *atp6*, *cox3*, *nad4l*, and *nad5*. TAG was used by *nad2*, *nad3*, and *nad6*, whereas a single terminal T was used by *cox2*, *nad4*, and *cob* (Table 1). These start‐ and stop‐codon profiles are consistent with patterns commonly reported in vertebrate mitogenomes, in which ATG is generally the predominant start codon, *cox1* often begins with GTG, and the stop‐codon set typically includes TAA and TAG as complete forms together with a truncated terminal T (Wang et al. 2015; Satoh et al. 2016). Comparable patterns have also been reported in other fish families, including Psettodidae, Scaridae, and Holocentridae (Kundu et al. 2023; Liu et al. 2023; Tang et al. 2023).

### 3.2 Nucleotide Composition and Codon Usage in Protein‐Coding Genes of Siganidae Mitogenomes

**FIGURE 2:**
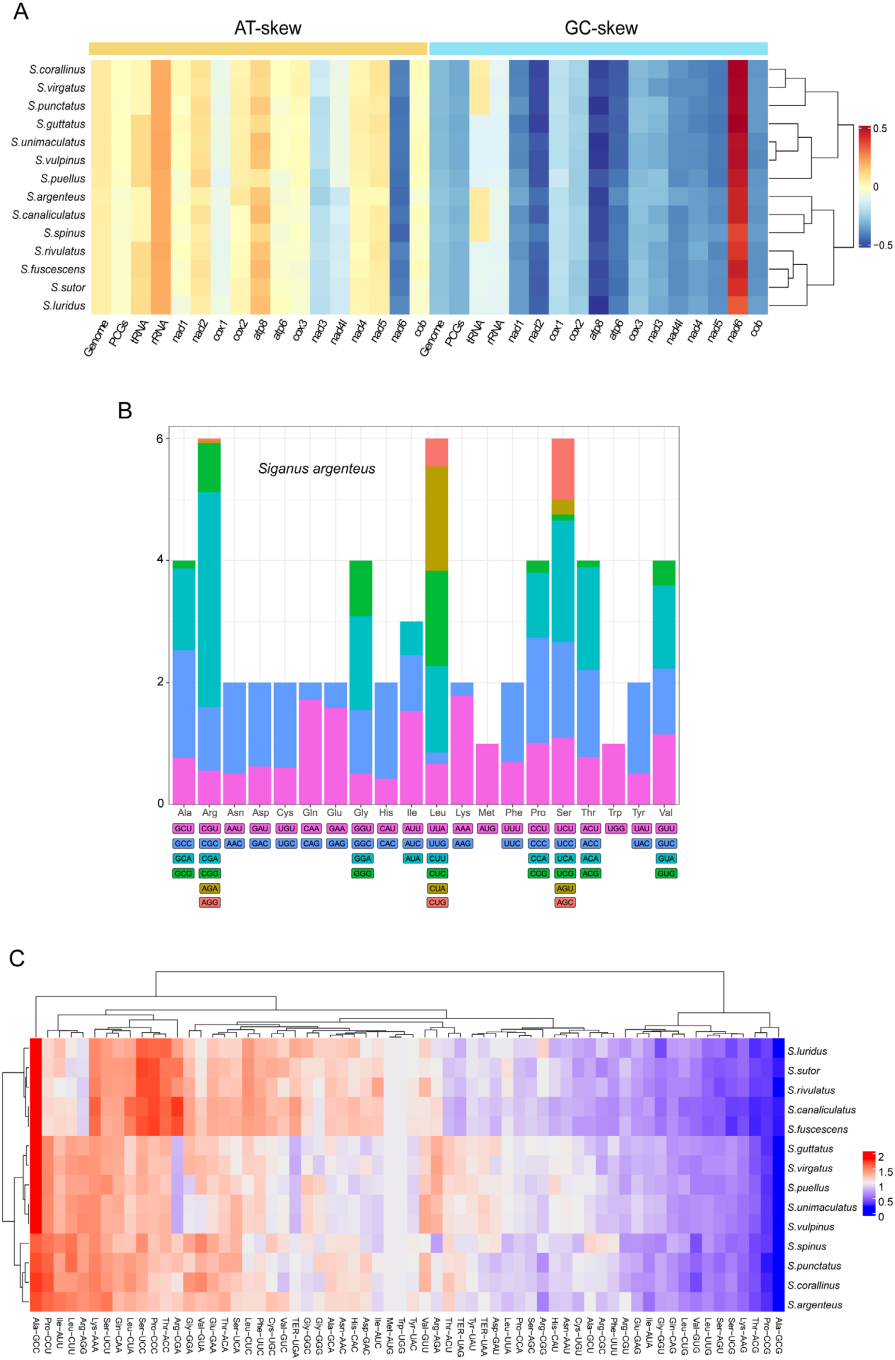
(A) Base composition skew in multiple mitochondrial genome datasets of 14 species, with hierarchical clustering of Siganidae (*y*‐axis) based on AT‐skew and GC‐skew. (B) Relative synonymous codon usage (RSCU) in the mitochondrial genome of a Siganidae fish, taking *Siganus argenteus* as an example. Codon families are annotated at the bottom of the *x*‐axis. (C) Heatmap of RSCU values across 14 Siganidae mitogenomes.

Base composition varied only slightly among the 14 sampled species. Across complete mitogenomes, A + T content was 53.11%–55.01%, with the minimum in *S. luridus* and the maximum in *S. virgatus* . The corresponding G + C content was 44.99%–46.89% (Table S3). Across mitochondrial coding regions, A + T accounted for 53.47% on average, compared with 54.04% at the complete‐mitogenome level. At the gene level, the A + T proportion reached its minimum in *nad4l* (48.56% ± 1.29%) and its maximum in *cox2* (58.22% ± 1.16%) (Table S3). In the representative *S. argenteus* mitogenome, PCGs contained 53.35% A + T (Table S3). At the component level, AT‐richness was evident in the complete mitogenomes, the tRNA and rRNA gene sets, and the majority of PCGs in the sampled siganids (Figure 2A, Table S3). Among these components, rRNA genes consistently displayed relatively high positive AT‐skew values. Unlike the other PCGs, the L‐strand‐encoded *nad6* showed the lowest AT‐skew and a contrasting GC‐skew pattern, with T more abundant than A and G more abundant than C (Figure 2A, Table S3). Most other PCGs showed higher AT‐skew than GC‐skew values. Across the full set of 13 PCGs, the mean compositional skew was below zero for both AT‐skew and GC‐skew (Figure 2A, Table S3). RSCU profiles of mitochondrial PCGs are presented in Figure 2B,C and Table S4. After excluding stop codons, 3811 codons were counted in the *S. argenteus* mitogenome. The most abundant codons corresponded to arginine (Arg), leucine (Leu), and serine (Ser), whereas codons for tryptophan (Trp) and methionine (Met) occurred least often (Figure 2B). The RSCU heatmap revealed highly similar codon usage profiles among the 14 *Siganus* mitogenomes (Figure 2C), indicating that codon usage bias is largely conserved among the sampled siganids. In agreement with their AT‐rich composition, A‐ or T‐ending codons occurred more often than G‐ or C‐ending codons, which is consistent with patterns previously reported for fish mitogenomes (Cui et al. 2017; Tang et al. 2023; Marnis et al. 2024). This pattern suggests that codon usage bias in Siganidae mitochondrial PCGs is more likely associated with the underlying nucleotide composition bias than with a strongly species‐ or lineage‐specific codon usage pattern. Among PCGs, *nad6* was the most divergent in compositional skew, showing negative AT‐skew and an atypical GC‐skew profile, while the other PCGs displayed more comparable strand‐associated trends (Figure 2A, Table S3). Similar strand‐associated skew patterns of *nad6* have also been reported in other fish mitogenomes, suggesting that this deviation is more likely related to strand‐specific compositional bias than to a Siganidae‐specific adaptive signal (Tang et al. 2023; Marnis et al. 2024). Overall, codon‐use and base‐composition patterns in siganid mitogenomes appear largely conserved at the family level, whereas gene‐specific deviations mainly reflect strand location and gene identity.

### 3.3 Transfer RNAs and Ribosomal RNAs

**FIGURE 3:**
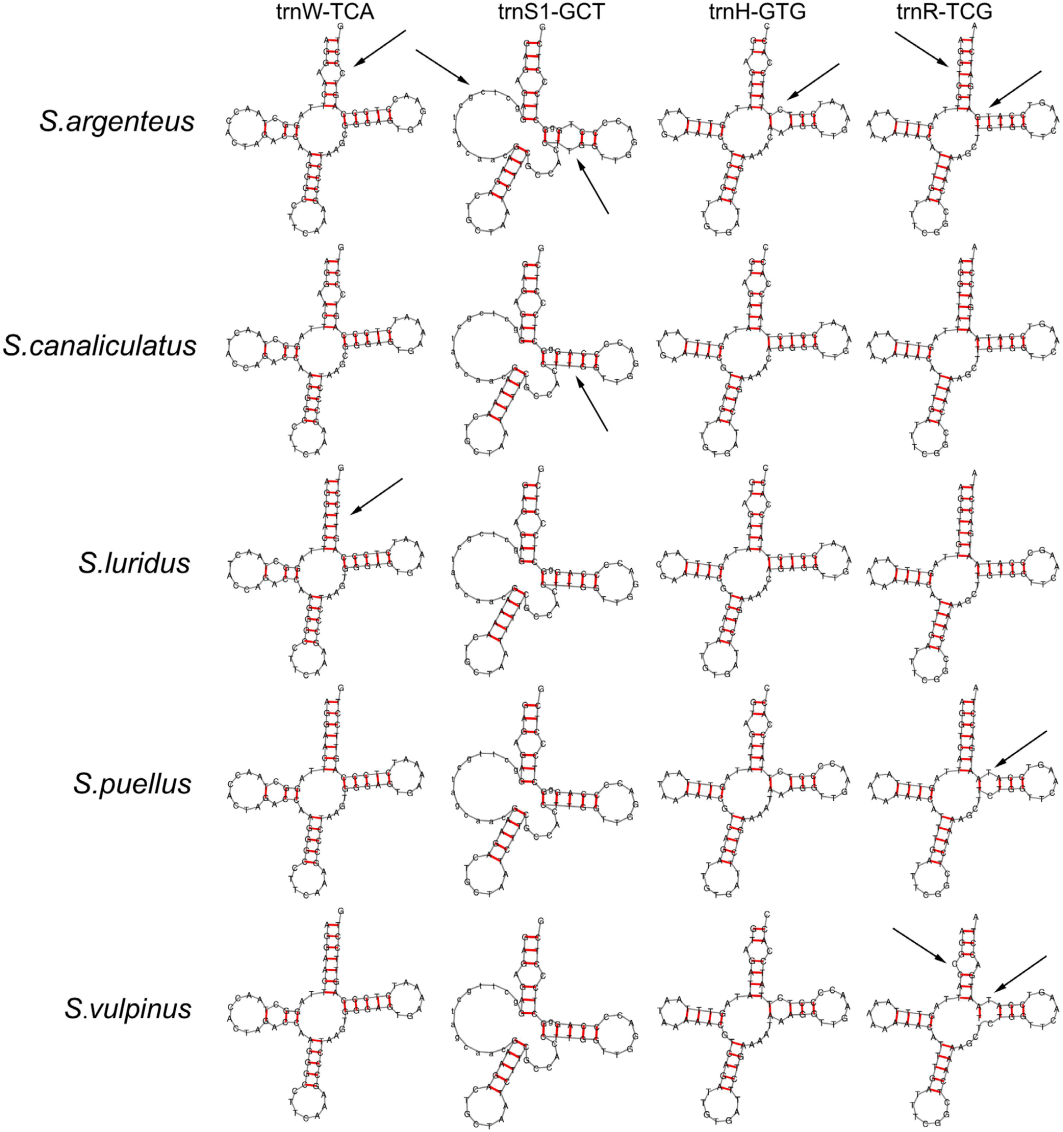
Putative secondary structures of representative divergent mitochondrial tRNAs in Siganidae fishes. Divergent secondary structures of *trnS1‐GCT* (Ser), *trnW‐TCA* (Trp), *trnR‐TCG* (Arg), and *trnH‐GTG* (His) across representative Siganidae species. Arrows highlight distinctive structural features of the four tRNAs. The complete set of putative secondary structures of 22 tRNAs is provided in Figure S3.

Twenty‐two tRNAs, typical of vertebrate mitochondrial genomes, were identified in all siganid mitogenomes (Figure S3). In the representative *S. argenteus* mitogenome, tRNA length varied from 66 to 75 bp, and most retained a canonical cloverleaf‐like secondary structure (Figure S3). Unlike the vast majority of mitochondrial tRNAs that form the classic cloverleaf structure, *trnS1‐GCT* (Ser) in all examined Siganidae species was found to completely lack the D stem, rendering it incapable of folding into the typical cloverleaf structure. In addition, this tRNA also showed a distinctive variable loop and an enlarged loop in the D‐arm region. Representative tRNA structures are shown in Figure 3, whereas the full set of 22 predicted tRNA structures for *S. argenteus* is provided in Figure S3. This recurrent structural feature may represent a family‐level characteristic of Siganidae. In *S. argenteus* , a U–U mismatch in the T stem of *trnS1‐GCT* (Ser) additionally resulted in the formation of a bulge loop. Similarly, a bulge loop formed in the D stem region of *trnW‐TCA* (Trp) in *S. canaliculatus* , *S. sutor* , *S. rivulatus* and *S. argenteus* due to base mismatches. A bulge loop formed in the T stem region of *trnR‐TCG* (Arg) in *S. virgatus* , *S. guttatus* , *S. puellus* , *S. corallinus* , *S. punctatus* , *S. vulpinus* , and *S. unimaculatus* , with *S. vulpinus* and *S. unimaculatus* also forming a bulge loop in the AA stem region. Additionally, a bulge loop formed in the T stem region of *trnH‐GTG* (His) in *S. virgatus* , *S. vulpinus* , *S. guttatus* , *S. puellus* , *S. unimaculatus* , *S. argenteus* , *S. corallinus* , *S. punctatus* , and *S. spinus* (Figure 3). The presence of bulge loops did not disrupt the overall cloverleaf configuration, but it is noteworthy that the occurrence was not random at specific positions on different tRNA genes through our observation. Instead, it exhibits a particular pattern correlated with the phylogenetic relationships among the Siganidae species. The two rRNA genes, *rrnS* and *rrnL*, which encode 12S rRNA and 16S rRNA, respectively, were both positioned on the H‐strand. The *trnV‐TAC* (Val) gene occurred between them, an arrangement widely reported in vertebrate mitogenomes (Wang et al. 2015; Ding et al. 2022; Marnis et al. 2024). In *S. argenteus* , *rrnS* and *rrnL* were 946 and 1653 bp long, respectively. Together, *rrnS* and *rrnL* showed a combined A + T proportion of 53.44%, exceeding their G + C content. For these two rRNA genes in *S. argenteus* , the skew estimates were 0.20 (AT‐skew) and −0.09 (GC‐skew) (Table S3). The remaining taxa followed the same overall rRNA composition pattern.

### 3.4 Overlapping Regions, Control Region, and Origin of Light‐Strand Replication (O_L_ )

**FIGURE 4:**
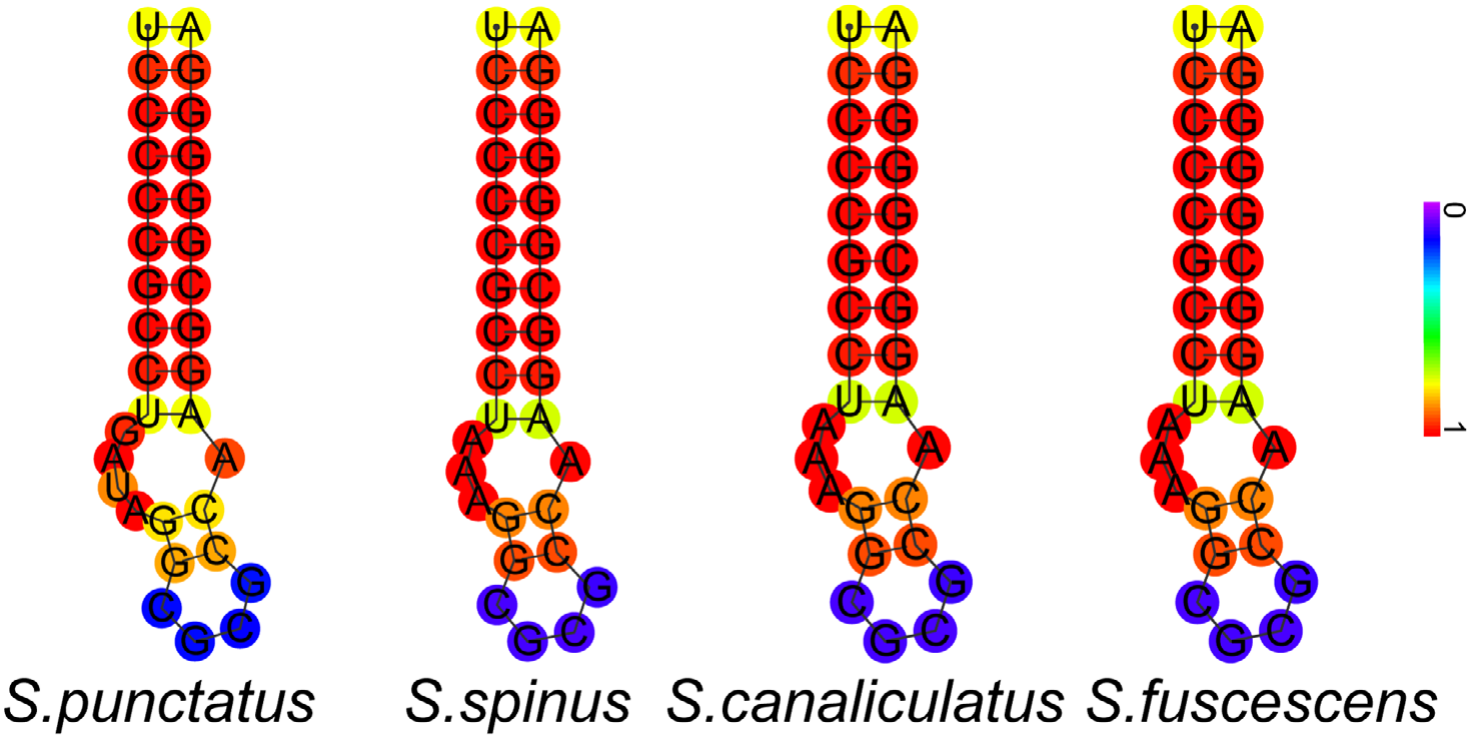
Putative secondary structure of representative origins of light‐strand replication (O_*L*_) in Siganidae mitogenomes. The representative O_*L*_ structures show the eight‐G–C‐pair stem type in *S. punctatus* and *S. spinus* , and the seven‐G–C‐pair stem type in *S. canaliculatus* and *S. fuscescens* . The color gradient scale represents base‐pairing probabilities calculated by CentroidFold software (http://rtools.cbrc.jp/centroidfold/, accessed on June 1, 2025). The complete set of putative O_*L*_ secondary structures across 14 Siganidae mitogenomes is provided in Figure S5.

Among the 12 H‐strand PCGs, three overlapping regions were detected (Table 1; Table S2). In *S. argenteus* , the largest overlap was a 10‐bp interval between *atp8* and *atp6* and contained the conserved motif ATGACACTAA. Another overlap, 7 bp in length, occurred between *nad4l* and *nad4* and contained the sequence ATGCTAA. A shorter 4‐bp CTAA overlap spanned the *nad5* and *nad6* gene boundary. The control region lay on this same strand, was flanked by *trnP* and *trnF*, and displayed the greatest interspecific variability. This region measured 828 bp in *S. fuscescens* and 841 bp in *S. corallinus* , representing the observed size range. Notably, length variation in this region is the primary factor contributing to the overall length differences observed in the mitogenomes of Siganidae fishes. We identified five conserved sequence blocks (CSBs) by aligning control region sequences (Figure S4). The results revealed a high density of conserved sites, with strong consistency in base‐composition patterns within individual CSBs across species. As summarized in Table 2, the CSBs showed different base‐composition profiles, with CSB‐I enriched in G/C, CSB‐II and CSB‐V in A/T, CSB‐III in C, and CSB‐IV in A/C. The O_*L*_ was identified as a specialized non‐coding element of 30–33 bp and was associated with the genomic region containing *nad6* and eight tRNA genes across the sampled species. Within the WANCY tRNA cluster, O_*L*_ formed a stable stem‐loop structure. A key finding is the variation in the number of G–C base pairs within the stem region of O_*L*_ among different Siganidae species. *S. argenteus* , *S. corallinus* , *S. punctatus* , *S. spinus* , *S. virgatus* , *S. vulpinus* , *S. guttatus* , *S. puellus* , *S. sutor* , *S. unimaculatus* , *S. rivulatus* , and *S. luridus* exhibited a compact structure containing eight G–C base pairs, whereas *S. canaliculatus* and *S. fuscescens* contained seven G–C base pairs. Representative O_*L*_ structures are shown in Figure 4, and the complete O_*L*_ structures of all sampled species are provided in Figure S5. This interspecific difference represents structural variation in O_*L*_ among the sampled Siganidae species, and the shared pattern observed in *S. canaliculatus* and *S. fuscescens* is consistent with their close mitochondrial relationship. Although G–C pairing in the stem region was largely retained across species and likely contributed to structural stability, variation in the loop region, particularly the limited occurrence of T bases in some species, further indicates sequence plasticity in this region while maintaining its fundamental function.

**TABLE 2:** Nucleotide composition of conserved sequence blocks (CSBs) in the mitochondrial control region of Siganidae mitogenomes.

Base composition (%)	A	T	G	C
CSB‐I	18.87	24.69	25.31	31.13
CSB‐II	32.00	29.43	14.86	23.71
CSB‐III	15.38	19.15	24.22	41.24
CSB‐IV	33.90	19.97	16.25	29.88
CSB‐V	33.28	28.64	14.24	23.84

### 3.5 Phylogenetic Analysis

**FIGURE 5:**
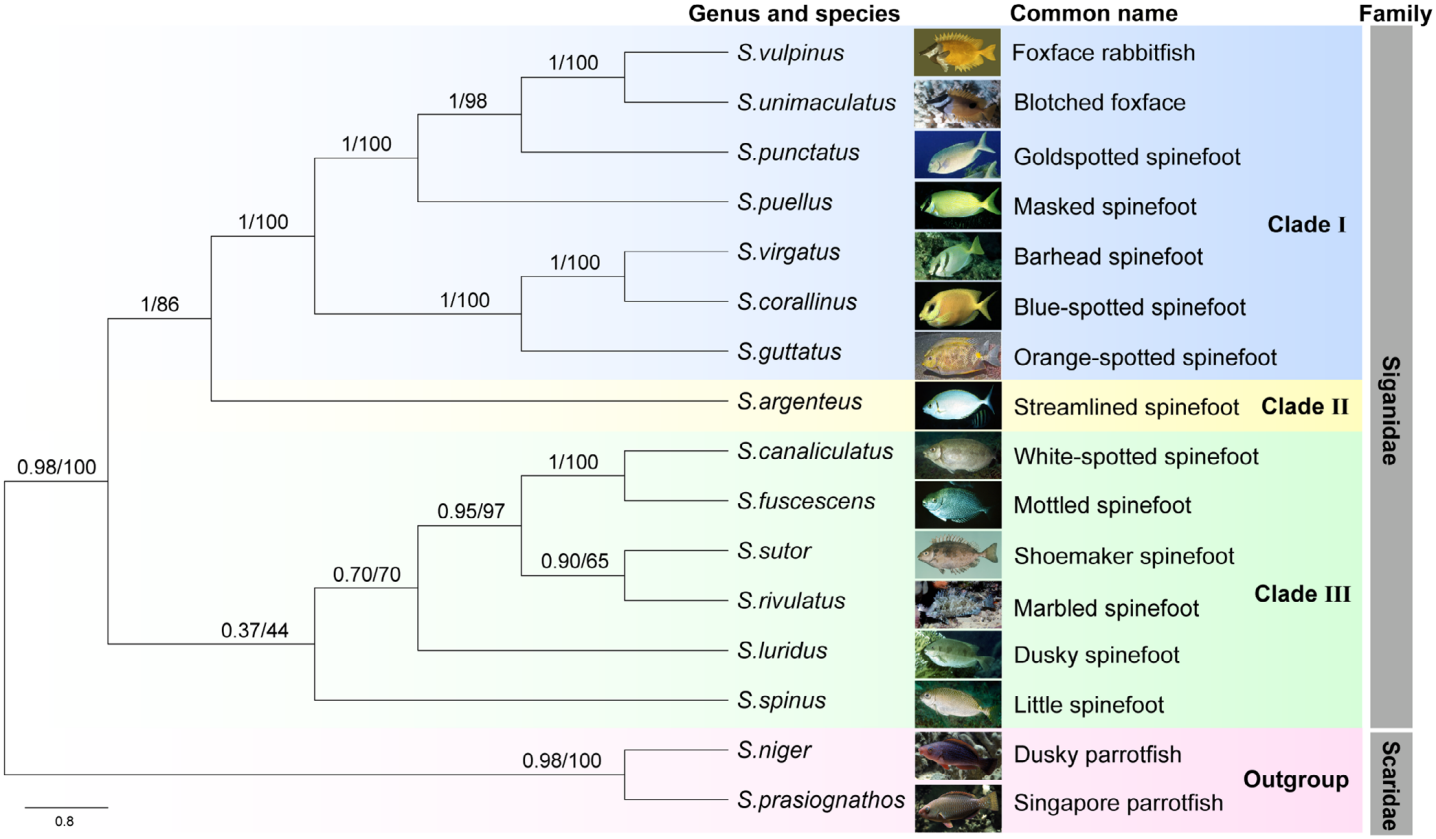
Phylogenetic trees of Siganidae reconstructed from nucleotide sequences of 13 mitochondrial protein‐coding genes (PCGs), with two parrotfish mitogenomes (Scaridae) as outgroups. Analyses employed Bayesian inference (BI) and maximum likelihood (ML). Values at nodes represent posterior probabilities (BI) and bootstrap support (ML).

For phylogenetic inference, alignments of the 13 mitochondrial PCGs from the 14 sampled siganids were concatenated to form one matrix. The resulting dataset was analyzed independently using ML and BI methods, and the trees were rooted using two parrotfish species from our previous study as outgroups (Gao et al. 2023). The ML and BI analyses recovered congruent topologies, and branch support values are shown as BI posterior probability/ML bootstrap percentage (PP/BP) in Figure 5. Both topologies separated the sampled siganids into three principal lineages, here designated as Clades I–III. Clade I included seven species: *S. vulpinus* , *S. unimaculatus* , *S. punctatus* , *S. puellus* , *S. virgatus* , *S. corallinus* , and *S. guttatus* . Clade II was represented by the single species *S. argenteus* , whereas Clade III included *S. canaliculatus* , *S. fuscescens* , *S. sutor* , *S. rivulatus* , *S. luridus* , and *S. spinus* . Within Clade I, *S. unimaculatus* and *S. vulpinus* clustered as a monophyletic group with maximal support, with BI posterior probability (PP) = 1.00 and ML bootstrap proportion (BP) = 100, indicating their close phylogenetic relationship. *S. virgatus* and *S. corallinus* also clustered together with full support (PP = 1, BP = 100), indicating their close mitochondrial relationship. Clade II comprised only the widely distributed species *S. argenteus* , which formed a distinct single‐species mitochondrial clade in the present tree. In Clade III, *S. canaliculatus* and *S. fuscescens* formed one sister pair with exceptionally high support (PP = 1.00, BP = 100), whereas *S. sutor* and *S. rivulatus* formed another sister pair in the inferred topology (PP = 0.90, BP = 65). These two sister pairs together constituted a well‐supported four‐species subclade (PP = 0.95, BP = 97). This topology agrees broadly with previous studies in which the *S. canaliculatus* – *S. fuscescens* pair and the *S. sutor* – *S. rivulatus* pair were placed within the fusiform or slender‐bodied siganid lineage (Borsa et al. 2007; Zolkaply et al. 2021). Furthermore, this subclade clustered with *S. luridus* and *S. spinus* , completing Clade III in the present mitochondrial phylogeny (Figure 5).

### 3.6 Selection Pressure Analysis of Mitochondrial Protein‐Coding Genes

**FIGURE 6:**
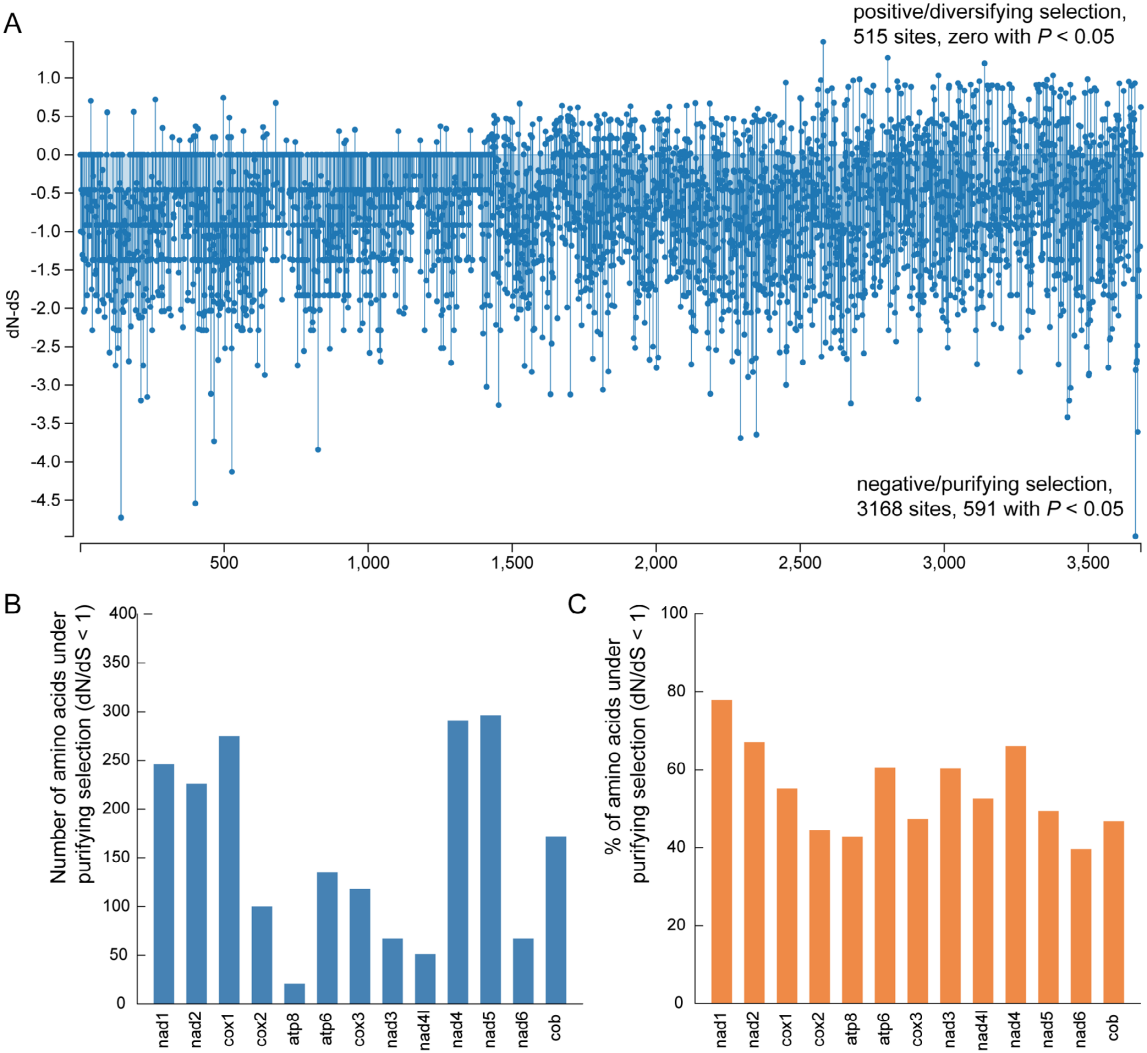
Selection pressure analysis of 13 protein‐coding genes (PCGs) in the mitochondrial genome. (A) Site‐specific *d*_N_–*d*_S_ analysis results obtained using the SLAC method. (B) Number of amino acid sites under purifying selection (*d*_N_/*d*_S_ < 1 or *ω* < 1) identified by the FEL method at *p* < 0.05. (C) The percentage of amino acids under purifying selection (% of amino acids) was determined by the FEL method at *p* < 0.05.

To assess the mechanisms of selective pressure, *d*_N_ and *d*_S_ substitution rates were calculated using the SLAC and FEL approaches, namely Single Likelihood Ancestor Counting and Fixed Effects Likelihood, respectively. The aligned dataset included 3683 amino‐acid positions (Figure 6). In the SLAC analysis, 515 sites showed positive *d*_N_–*d*_S_ values, but none of these signals was statistically significant (*p* > 0.05). Of the remaining 3168 sites, 591 showed significantly negative *d*_N_–*d*_S_ values (*p* < 0.05), consistent with purifying selection, whereas the other sites were not significant (Figure 6A). The FEL analysis identified 2065 sites with *ω* values below 1 (*d*_N_/*d*_S_ < 1) and significant evidence of purifying selection at *p* < 0.05 (Table S5). Among these, *nad5* had the largest number of sites inferred to be under purifying selection, whereas *atp8* had the fewest (Figure 6B). In terms of proportion, *nad1* exhibited the highest proportion of sites under purifying selection, while *nad6* showed the lowest proportion (Figure 6C). Overall, all PCGs showed evidence of predominant purifying selection. At the descriptive level, most sites had negative *d*_N_–*d*_S_ values, consistent with *d*_N_ */d*_S_ < 1 (*ω* < 1), whereas 591 sites were significant for purifying selection under the *p* < 0.05 threshold.

## 4 Discussion

In the present comparative analysis, *trnS1‐GCT* (Ser) emerged as the most distinctive tRNA gene, showing a recurrent structural pattern characterized by a distinctive variable loop, an enlarged loop in the D‐arm region, and loss of the D stem, resulting in an incomplete cloverleaf configuration. Such D‐arm loss has been widely reported in metazoan mitochondrial tRNAs, particularly in mitochondrial tRNAs for serine, and previous studies suggest that structurally reduced mitochondrial tRNAs may remain compatible with mitochondrial translation through specialized RNA‐protein interactions (Watanabe et al. 2014; Kuhle et al. 2022). Meanwhile, base mismatches were present in some tRNAs, leading unpaired bases to bulge outward and form bulge loops. Bulge loops are common non‐canonical nucleic‐acid structural motifs with known thermodynamic effects on secondary‐structure formation and stability (SantaLucia Jr. and Hicks 2004), while tRNA sequence features and modifications may further influence folding stability and decoding performance (Kompatscher et al. 2024). In the present dataset, the distribution of several tRNA structural variations, particularly bulge loops, showed a pattern broadly consistent with the phylogeny inferred from 13 PCGs. A similar phylogeny‐concordant pattern was also observed for O_*L*_, in which the seven‐G–C‐pair stem structure was shared by the sister pair *S. canaliculatus* and *S. fuscescens* , whereas most other sampled siganids contained eight G–C base pairs. Together, these tRNA and O_*L*_ structural features provide useful comparative characters for describing mitochondrial variation within Siganidae and may generate hypotheses for future studies of mitochondrial structural evolution. Their functional significance remains to be evaluated using broader taxon sampling, RNA structural modeling, and functional validation.

Furthermore, the ML and BI trees inferred from the 13 mitochondrial PCGs consistently supported three major mitochondrial clades within Siganidae, rather than representing an isolated pattern. This grouping is generally consistent with previous studies that identified three major siganid lineages and reported associations between these lineages and several morphological, ecological, or distributional characteristics, although the numbering of clades differs among studies (Borsa et al. 2007; Kuriiwa et al. 2007; Lemer et al. 2007; Zolkaply et al. 2021). In the present tree, Clade I mainly includes taxa corresponding to lineages previously characterized as deeper‐bodied, reef‐associated, paired, or mangrove/estuarine‐associated groups. Clade III includes several fusiform or inshore schooling species, whereas Clade II contains only *S. argenteus* , a widely distributed species with a pelagic pre‐juvenile stage (Borsa et al. 2007; Zolkaply et al. 2021). Within Clade III, previously reported distributional records also indicate that *S. canaliculatus* – *S. fuscescens* and *S. sutor* – *S. rivulatus* represent geographically differentiated but phylogenetically related lineages, a pattern compatible with the four‐species subclade recovered here (Borsa et al. 2007; Zolkaply et al. 2021). The observed correspondence should therefore be interpreted as a qualitative pattern consistent with previous taxonomic, ecological, and distributional observations, because the present study did not include quantitative analyses of morphology, habitat use, ecological niche differentiation, trait evolution, or population‐level geographic structure.

Selection analyses indicated that mitochondrial PCGs in the sampled Siganidae species were predominantly under purifying selection, with no codon site showing statistically significant evidence of positive selection in the SLAC analysis. Comparable mitogenomic analyses of reef fishes have likewise reported that mitochondrial PCGs are mainly shaped by purifying constraint, consistent with the pattern recovered here (Gao et al. 2023; Tang et al. 2023). For the sampled siganids, the proportion of sites inferred to be under purifying selection was highest in *nad1* and lowest in *nad6*. Such among‐gene variation may reflect differences in functional constraint and sequence conservation. *nad1* encodes NADH dehydrogenase subunit 1, a membrane‐associated component of mitochondrial respiratory complex I. This complex couples NADH oxidation with quinone reduction and transmembrane proton translocation, thereby contributing to oxidative phosphorylation (Parey et al. 2021; Agip et al. 2023). Therefore, the relatively high proportion of constrained sites in *nad1* is consistent with its conserved bioenergetic function. By contrast, the lower proportion of sites under purifying selection in *nad6* may indicate comparatively weaker constraint or higher sequence variability. Because the overall pattern was dominated by purifying selection, these gene‐level differences should be interpreted primarily as variation in selective constraint among mitochondrial genes and as a basis for generating hypotheses for future functional studies. Besides, *nad5* encodes NADH dehydrogenase subunit 5, a membrane‐associated component of respiratory complex I, whereas *atp8* encodes ATP synthase subunit 8, which contributes to ATP synthase activity during oxidative phosphorylation. Given their different patterns of selective constraint observed in this study, *nad5* and *atp8* may warrant further evaluation as candidate mitochondrial loci for siganid species identification. Their diagnostic utility should be assessed using diagnostic‐site screening, interspecific genetic‐distance comparisons, and broader population‐level sampling.

## 5 Conclusions

In this work, four newly generated siganid mitogenomes were integrated with 10 published Siganidae mitogenomes to assess genome organization, sequence composition, structural variation, and mitochondrial phylogenetic patterns within the family. Characterization revealed that siganid mitogenomes are highly conserved in organization and base composition. The control region exhibited the highest interspecific variation, and structural features of tRNAs and the O_*L*_ showed notable interspecific differences. These structural features were broadly concordant with phylogenetic relationships inferred from mitochondrial PCGs. Phylogenetic analyses based on 13 PCGs consistently divided the sampled *Siganus* species into three major clades, with *S. argenteus* forming a distinct single‐species clade. This topology was broadly consistent with previously reported major siganid lineages. Selection‐pressure results supported predominant purifying selection across mitochondrial PCGs, with variation in constraint strength among genes, and no codon site showed statistically significant evidence of positive selection in the SLAC analysis. The *nad5* and *atp8* genes may represent potential mitochondrial loci for future evaluation in siganid species identification. This research enriches mitogenomic resources for siganids and provides a comparative reference for future studies on siganid phylogeny, mitochondrial genome evolution, and species identification.

## Author Contributions

**Hao‐Ran Ma:** formal analysis (lead), investigation (lead), visualization (lead), writing – original draft (lead). **Le‐Jia Pang:** formal analysis (lead), visualization (equal), writing – original draft (equal), writing – review and editing (supporting). **Qin Tang:** conceptualization (lead), funding acquisition (lead), project administration (lead), supervision (lead), writing – review and editing (equal). **Teng Wang:** conceptualization (lead), funding acquisition (lead), project administration (lead), supervision (lead), writing – review and editing (equal).

## Funding

This research was supported by the National Key R&D Program of China (2024YFD2401801); the National Natural Science Foundation of China (32473161); the Financial Fund of the Ministry of Agriculture and Rural Affairs, P. R. China (NHZX2024, SYHZX2025); Central Public‐interest Scientific Institution Basal Research Fund, CAFS (No. 2023TD16) and the Central Public‐Interest Scientific Institution Basal Research Fund, South China Sea Fisheries Research Institute, CAFS (2025RC03).

## Ethics Statement

All field sampling was conducted in accordance with local fisheries regulations and institutional guidelines. No endangered or protected species were involved in this study. All procedures involving animals were conducted under the supervision of the South China Sea Fisheries Research Institute, Chinese Academy of Fishery Sciences, and were approved by its institutional ethics committee. The ethical review number is nhdf2022‐21.

## Conflicts of Interest

The authors declare no conflicts of interest.

## Supporting information


**Figure S1:** Circular mitochondrial genome maps of the 14 Siganidae species analyzed in this study.

**Figure S2:** Multiple sequence alignment of mitochondrial control regions from the 14 Siganidae mitogenomes.

**Figure S3:** Representative putative secondary structures of the 22 mitochondrial tRNAs in Siganidae, shown using *Siganus argenteus* as an example.

**Figure S4:** Alignment of conserved sequence blocks (CSBs) in mitochondrial control regions of the 14 Siganidae mitogenomes.

**Figure S5:** Putative secondary structures of the origin of light‐strand replication (O_*L*_) across the 14 Siganidae mitogenomes.

**Table S1:** Sequencing, assembly, and assembly‐support metrics for the four newly generated *Siganus* mitogenomes.

**Table S2:** Gene organization and annotation information for the 14 Siganidae mitogenomes analyzed in this study.

**Table S3:** Nucleotide composition and compositional skew of complete mitogenomes, mitochondrial genomic components, and the 13 protein‐coding genes in Siganidae.

**Table S4:** Relative synonymous codon usage (RSCU) values of mitochondrial protein‐coding genes in the 14 sampled siganid mitogenomes.

**Table S5:** Site‐level selection‐pressure estimates for the 13 mitochondrial protein‐coding genes based on the FEL analysis.

## Data Availability

The newly generated mitogenome sequences from this study have been deposited in the NCBI nucleotide database under the accession numbers PX112429, PX112430, PX112431, and NC_086723.
