# Supplementary material for: Comparative Mitogenome Analyses Uncover Mitogenome Features and Phylogenetic Implications of the Rabbitfishes (Perciformes: Siganidae)
Source: Ecol Evol. 2026 Aug 14;16(8):e74188. doi: 10.1002/ece3.74188 (PMC13476152; doi:10.1002/ece3.74188)
Supplement: Supplementary file 1 [file ECE3-16-e74188-s001.docx]

Table S5. Site-level selection-pressure estimates for the 13 mitochondrial protein-coding genes based on the FEL analysis.

| **codon** | **alpha** | **beta** | **alpha=beta** | **LRT** | **p-value** | **Total branch length** | **class** | **gene** |
| --- | --- | --- | --- | --- | --- | --- | --- | --- |
| **1** | **0** | **0** | **0** | **0** | **1** | **0** | **Invariable** | **nad1** |
| **2** | **1.139** | **0** | **0.022** | **13.348** | **0.0003** | **1.227** | **Purifying** | **nad1** |
| **3** | **0** | **0** | **0** | **0** | **1** | **0** | **Invariable** | **nad1** |
| **4** | **0.219** | **0** | **0.008** | **4.918** | **0.0266** | **0.424** | **Purifying** | **nad1** |
| **5** | **0** | **0** | **0** | **0** | **1** | **0** | **Invariable** | **nad1** |
| **6** | **1.14** | **0** | **0.022** | **13.286** | **0.0003** | **1.224** | **Purifying** | **nad1** |
| **7** | **0** | **0** | **0** | **0** | **1** | **0** | **Invariable** | **nad1** |
| **8** | **0.943** | **0** | **0.017** | **8.73** | **0.0031** | **0.972** | **Purifying** | **nad1** |
| **9** | **0.029** | **0** | **0.006** | **2.549** | **0.1104** | **0.348** | **Neutral** | **nad1** |
| **10** | **0.802** | **0** | **0.015** | **10.565** | **0.0012** | **0.865** | **Purifying** | **nad1** |
| **11** | **10000** | **0** | **0.02** | **18.011** | **0** | **1.108** | **Purifying** | **nad1** |
| **12** | **0.503** | **0** | **0.008** | **4.548** | **0.033** | **0.436** | **Purifying** | **nad1** |
| **13** | **3.11** | **0** | **1.016** | **18.661** | **0** | **57.085** | **Purifying** | **nad1** |
| **14** | **0** | **0** | **0** | **0** | **1** | **0** | **Invariable** | **nad1** |
| **15** | **3.281** | **0** | **0.02** | **18.028** | **0** | **1.115** | **Purifying** | **nad1** |
| **16** | **3.704** | **0** | **0.569** | **22.902** | **0** | **31.958** | **Purifying** | **nad1** |
| **17** | **2.447** | **0** | **0.387** | **19.573** | **0** | **21.728** | **Purifying** | **nad1** |
| **18** | **1.219** | **0** | **0.02** | **13.146** | **0.0003** | **1.123** | **Purifying** | **nad1** |
| **19** | **0.55** | **0** | **0.018** | **9.18** | **0.0024** | **1.006** | **Purifying** | **nad1** |
| **20** | **0.027** | **0** | **0.008** | **2.343** | **0.1259** | **0.445** | **Neutral** | **nad1** |
| **21** | **0** | **0** | **0** | **0** | **1** | **0** | **Invariable** | **nad1** |
| **22** | **1.204** | **0** | **0.016** | **13.779** | **0.0002** | **0.883** | **Purifying** | **nad1** |
| **23** | **0.022** | **0** | **0.006** | **2.443** | **0.1181** | **0.316** | **Neutral** | **nad1** |
| **24** | **2.239** | **0** | **0.428** | **22.088** | **0** | **24.065** | **Purifying** | **nad1** |
| **25** | **0.886** | **0** | **0.007** | **7.77** | **0.0053** | **0.407** | **Purifying** | **nad1** |
| **26** | **0.81** | **0** | **0.017** | **8.792** | **0.003** | **0.972** | **Purifying** | **nad1** |
| **27** | **0.636** | **0** | **0.007** | **5.184** | **0.0228** | **0.384** | **Purifying** | **nad1** |
| **28** | **3.235** | **0** | **0.828** | **18.469** | **0** | **46.527** | **Purifying** | **nad1** |
| **29** | **1.746** | **0.011** | **0.68** | **2.934** | **0.0867** | **38.214** | **Neutral** | **nad1** |
| **30** | **0** | **0** | **0** | **0** | **1** | **0** | **Invariable** | **nad1** |
| **31** | **0** | **0** | **0** | **0** | **1** | **0** | **Invariable** | **nad1** |
| **32** | **2.475** | **0** | **0.009** | **9.834** | **0.0017** | **0.484** | **Purifying** | **nad1** |
| **33** | **0.011** | **0** | **0.004** | **1.67** | **0.1963** | **0.251** | **Neutral** | **nad1** |
| **34** | **0.286** | **0** | **0.023** | **6.941** | **0.0084** | **1.288** | **Purifying** | **nad1** |
| **35** | **0.515** | **0** | **0.005** | **5.373** | **0.0205** | **0.286** | **Purifying** | **nad1** |
| **36** | **1** | **0** | **0.008** | **7.648** | **0.0057** | **0.463** | **Purifying** | **nad1** |
| **37** | **0** | **0.335** | **0.385** | **1.214** | **0.2706** | **21.648** | **Neutral** | **nad1** |
| **38** | **0** | **0** | **0** | **0** | **1** | **0** | **Invariable** | **nad1** |
| **39** | **1.193** | **0.016** | **0.422** | **4.27** | **0.0388** | **23.716** | **Purifying** | **nad1** |
| **40** | **1.716** | **0** | **0.333** | **20.885** | **0** | **18.682** | **Purifying** | **nad1** |
| **41** | **0** | **0** | **0** | **0** | **1** | **0** | **Invariable** | **nad1** |
| **42** | **1.426** | **0** | **0.374** | **24.994** | **0** | **20.988** | **Purifying** | **nad1** |
| **43** | **1.847** | **0** | **0.406** | **18.235** | **0** | **22.834** | **Purifying** | **nad1** |
| **44** | **0** | **0** | **0** | **0** | **1** | **0** | **Invariable** | **nad1** |
| **45** | **0.032** | **0.01** | **0.016** | **0.342** | **0.5586** | **0.894** | **Neutral** | **nad1** |
| **46** | **0** | **0** | **0** | **0** | **1** | **0** | **Invariable** | **nad1** |
| **47** | **0.763** | **0** | **0.02** | **16.701** | **0** | **1.124** | **Purifying** | **nad1** |
| **48** | **2.016** | **0** | **0.415** | **18.612** | **0** | **23.295** | **Purifying** | **nad1** |
| **49** | **0** | **0** | **0** | **0** | **1** | **0** | **Invariable** | **nad1** |
| **50** | **0.561** | **0** | **0.005** | **5.066** | **0.0244** | **0.266** | **Purifying** | **nad1** |
| **51** | **1.04** | **0** | **0.028** | **10.376** | **0.0013** | **1.586** | **Purifying** | **nad1** |
| **52** | **2.589** | **0** | **0.662** | **16.441** | **0.0001** | **37.174** | **Purifying** | **nad1** |
| **53** | **1.809** | **0** | **0.006** | **4.609** | **0.0318** | **0.351** | **Purifying** | **nad1** |
| **54** | **1.605** | **0** | **0.381** | **17.535** | **0** | **21.393** | **Purifying** | **nad1** |
| **55** | **0.945** | **0** | **0.115** | **13.859** | **0.0002** | **6.433** | **Purifying** | **nad1** |
| **56** | **0.984** | **0** | **0.017** | **12.793** | **0.0003** | **0.932** | **Purifying** | **nad1** |
| **57** | **0.732** | **0** | **0.007** | **7.632** | **0.0057** | **0.383** | **Purifying** | **nad1** |
| **58** | **0.754** | **0** | **0.021** | **18.917** | **0** | **1.197** | **Purifying** | **nad1** |
| **59** | **0** | **0** | **0** | **0** | **1** | **0** | **Invariable** | **nad1** |
| **60** | **0** | **0** | **0** | **0** | **1** | **0** | **Invariable** | **nad1** |
| **61** | **1.106** | **0** | **0.025** | **11.268** | **0.0008** | **1.419** | **Purifying** | **nad1** |
| **62** | **4.97** | **0** | **0.388** | **24.658** | **0** | **21.816** | **Purifying** | **nad1** |
| **63** | **0.346** | **0** | **0.016** | **7.959** | **0.0048** | **0.897** | **Purifying** | **nad1** |
| **64** | **0** | **0** | **0** | **0** | **1** | **0** | **Invariable** | **nad1** |
| **65** | **2.475** | **0** | **0.019** | **13.178** | **0.0003** | **1.072** | **Purifying** | **nad1** |
| **66** | **1.336** | **0** | **0.02** | **13.129** | **0.0003** | **1.122** | **Purifying** | **nad1** |
| **67** | **2.353** | **0** | **0.357** | **18.836** | **0** | **20.085** | **Purifying** | **nad1** |
| **68** | **0.527** | **0** | **0.008** | **3.246** | **0.0716** | **0.422** | **Neutral** | **nad1** |
| **69** | **2.087** | **0** | **0.022** | **14.403** | **0.0001** | **1.216** | **Purifying** | **nad1** |
| **70** | **0** | **0** | **0** | **0** | **1** | **0** | **Invariable** | **nad1** |
| **71** | **0.515** | **0** | **0.007** | **4.816** | **0.0282** | **0.383** | **Purifying** | **nad1** |
| **72** | **0** | **0** | **0** | **0** | **1** | **0** | **Invariable** | **nad1** |
| **73** | **0.55** | **0** | **0.007** | **4.956** | **0.026** | **0.372** | **Purifying** | **nad1** |
| **74** | **0.712** | **0** | **0.008** | **5.038** | **0.0248** | **0.438** | **Purifying** | **nad1** |
| **75** | **2.556** | **0** | **0.351** | **19.637** | **0** | **19.732** | **Purifying** | **nad1** |
| **76** | **0.825** | **0** | **0.028** | **9.47** | **0.0021** | **1.598** | **Purifying** | **nad1** |
| **77** | **2.309** | **0** | **0.339** | **21.816** | **0** | **19.062** | **Purifying** | **nad1** |
| **78** | **1.477** | **0** | **0.252** | **11.374** | **0.0007** | **14.18** | **Purifying** | **nad1** |
| **79** | **1.666** | **0** | **0.436** | **14.859** | **0.0001** | **24.496** | **Purifying** | **nad1** |
| **80** | **2.902** | **0** | **0.416** | **22.172** | **0** | **23.369** | **Purifying** | **nad1** |
| **81** | **1.836** | **0** | **0.015** | **12.519** | **0.0004** | **0.855** | **Purifying** | **nad1** |
| **82** | **4.199** | **0.01** | **0.541** | **10.56** | **0.0012** | **30.378** | **Purifying** | **nad1** |
| **83** | **0.816** | **0** | **0.252** | **10.756** | **0.001** | **14.16** | **Purifying** | **nad1** |
| **84** | **0.422** | **0** | **0.006** | **4.778** | **0.0288** | **0.344** | **Purifying** | **nad1** |
| **85** | **5.516** | **0** | **1.217** | **23.846** | **0** | **68.387** | **Purifying** | **nad1** |
| **86** | **0.529** | **0** | **0.007** | **4.871** | **0.0273** | **0.383** | **Purifying** | **nad1** |
| **87** | **1.6** | **0** | **0.462** | **13.751** | **0.0002** | **25.972** | **Purifying** | **nad1** |
| **88** | **0.514** | **0** | **0.006** | **5.048** | **0.0247** | **0.345** | **Purifying** | **nad1** |
| **89** | **0.66** | **0** | **0.017** | **8.202** | **0.0042** | **0.967** | **Purifying** | **nad1** |
| **90** | **0.732** | **0** | **0.007** | **5.084** | **0.0241** | **0.402** | **Purifying** | **nad1** |
| **91** | **2.055** | **0** | **0.524** | **16.122** | **0.0001** | **29.428** | **Purifying** | **nad1** |
| **92** | **0** | **0** | **0** | **0** | **1** | **0** | **Invariable** | **nad1** |
| **93** | **3.301** | **0** | **0.452** | **21.076** | **0** | **25.393** | **Purifying** | **nad1** |
| **94** | **0** | **0.31** | **0.365** | **2.135** | **0.1439** | **20.534** | **Neutral** | **nad1** |
| **95** | **0.635** | **0** | **0.008** | **4.915** | **0.0266** | **0.438** | **Purifying** | **nad1** |
| **96** | **1.113** | **0.421** | **0.628** | **0.794** | **0.3728** | **35.257** | **Neutral** | **nad1** |
| **97** | **4.428** | **0** | **0.62** | **23.642** | **0** | **34.859** | **Purifying** | **nad1** |
| **98** | **1.074** | **0** | **0.008** | **7.787** | **0.0053** | **0.463** | **Purifying** | **nad1** |
| **99** | **5.752** | **0** | **0.652** | **25.463** | **0** | **36.625** | **Purifying** | **nad1** |
| **100** | **1.356** | **0** | **0.016** | **13.074** | **0.0003** | **0.903** | **Purifying** | **nad1** |
| **101** | **1.295** | **0** | **0.395** | **12.349** | **0.0004** | **22.167** | **Purifying** | **nad1** |
| **102** | **5.12** | **0** | **0.309** | **27.006** | **0** | **17.361** | **Purifying** | **nad1** |
| **103** | **1.382** | **0** | **0.254** | **12.72** | **0.0004** | **14.263** | **Purifying** | **nad1** |
| **104** | **1.074** | **0** | **0.007** | **7.598** | **0.0058** | **0.385** | **Purifying** | **nad1** |
| **105** | **0.751** | **0** | **0.028** | **9.167** | **0.0025** | **1.589** | **Purifying** | **nad1** |
| **106** | **1.947** | **0** | **0.028** | **24.165** | **0** | **1.562** | **Purifying** | **nad1** |
| **107** | **1.545** | **0** | **0.022** | **14.113** | **0.0002** | **1.235** | **Purifying** | **nad1** |
| **108** | **1.442** | **0** | **0.026** | **12.235** | **0.0005** | **1.436** | **Purifying** | **nad1** |
| **109** | **2.411** | **0** | **0.342** | **22.001** | **0** | **19.221** | **Purifying** | **nad1** |
| **110** | **1.663** | **0** | **0.022** | **14.168** | **0.0002** | **1.233** | **Purifying** | **nad1** |
| **111** | **3.104** | **0** | **0.776** | **16.994** | **0** | **43.625** | **Purifying** | **nad1** |
| **112** | **1.194** | **0** | **0.012** | **12.47** | **0.0004** | **0.701** | **Purifying** | **nad1** |
| **113** | **1.046** | **0** | **0.018** | **9.982** | **0.0016** | **0.985** | **Purifying** | **nad1** |
| **114** | **0** | **0** | **0** | **0** | **1** | **0** | **Invariable** | **nad1** |
| **115** | **1.111** | **0** | **0.006** | **8.59** | **0.0034** | **0.311** | **Purifying** | **nad1** |
| **116** | **2.086** | **0** | **0.78** | **15.775** | **0.0001** | **43.809** | **Purifying** | **nad1** |
| **117** | **1.242** | **0** | **0.274** | **17.981** | **0** | **15.382** | **Purifying** | **nad1** |
| **118** | **3.658** | **0** | **0.357** | **19.755** | **0** | **20.052** | **Purifying** | **nad1** |
| **119** | **10000** | **0** | **0.395** | **30.065** | **0** | **22.178** | **Purifying** | **nad1** |
| **120** | **3.145** | **0** | **0.365** | **22.392** | **0** | **20.523** | **Purifying** | **nad1** |
| **121** | **2.396** | **0** | **0.386** | **19.03** | **0** | **21.708** | **Purifying** | **nad1** |
| **122** | **3.037** | **0** | **0.621** | **17.559** | **0** | **34.893** | **Purifying** | **nad1** |
| **123** | **3.163** | **0** | **0.435** | **28.642** | **0** | **24.448** | **Purifying** | **nad1** |
| **124** | **1.616** | **0** | **0.019** | **13.733** | **0.0002** | **1.041** | **Purifying** | **nad1** |
| **125** | **2.829** | **0** | **0.452** | **28.996** | **0** | **25.417** | **Purifying** | **nad1** |
| **126** | **0.711** | **0** | **0.006** | **5.189** | **0.0227** | **0.359** | **Purifying** | **nad1** |
| **127** | **1.163** | **0** | **0.016** | **13.539** | **0.0002** | **0.889** | **Purifying** | **nad1** |
| **128** | **0** | **0** | **0** | **0** | **1** | **0** | **Invariable** | **nad1** |
| **129** | **1.081** | **0** | **0.016** | **13.308** | **0.0003** | **0.887** | **Purifying** | **nad1** |
| **130** | **1.428** | **0** | **0.009** | **9.276** | **0.0023** | **0.483** | **Purifying** | **nad1** |
| **131** | **10000** | **0** | **0.561** | **31.133** | **0** | **31.533** | **Purifying** | **nad1** |
| **132** | **0.636** | **0** | **0.006** | **5.382** | **0.0203** | **0.345** | **Purifying** | **nad1** |
| **133** | **2.066** | **0** | **0.717** | **16.574** | **0** | **40.26** | **Purifying** | **nad1** |
| **134** | **0.847** | **0** | **0.015** | **10.706** | **0.0011** | **0.865** | **Purifying** | **nad1** |
| **135** | **1.092** | **0** | **0.292** | **22.872** | **0** | **16.381** | **Purifying** | **nad1** |
| **136** | **0** | **0** | **0** | **0** | **1** | **0** | **Invariable** | **nad1** |
| **137** | **3.92** | **0** | **1.071** | **20.112** | **0** | **60.161** | **Purifying** | **nad1** |
| **138** | **0.052** | **0** | **0.007** | **3.056** | **0.0805** | **0.42** | **Neutral** | **nad1** |
| **139** | **0** | **0** | **0** | **0** | **1** | **0** | **Invariable** | **nad1** |
| **140** | **1.362** | **0** | **0.316** | **16.756** | **0** | **17.756** | **Purifying** | **nad1** |
| **141** | **1.672** | **0** | **0.016** | **14.883** | **0.0001** | **0.889** | **Purifying** | **nad1** |
| **142** | **10000** | **0** | **0.764** | **31.728** | **0** | **42.904** | **Purifying** | **nad1** |
| **143** | **0.711** | **0** | **0.007** | **4.983** | **0.0256** | **0.401** | **Purifying** | **nad1** |
| **144** | **2.406** | **0** | **0.349** | **18.653** | **0** | **19.583** | **Purifying** | **nad1** |
| **145** | **0.784** | **0** | **0.013** | **10.512** | **0.0012** | **0.716** | **Purifying** | **nad1** |
| **146** | **0** | **0** | **0** | **0** | **1** | **0** | **Invariable** | **nad1** |
| **147** | **1.428** | **0** | **0.019** | **12.621** | **0.0004** | **1.071** | **Purifying** | **nad1** |
| **148** | **1.615** | **0** | **0.021** | **14.326** | **0.0002** | **1.189** | **Purifying** | **nad1** |
| **149** | **3.513** | **0** | **0.019** | **27.598** | **0** | **1.073** | **Purifying** | **nad1** |
| **150** | **2.68** | **0** | **0.863** | **18.344** | **0** | **48.461** | **Purifying** | **nad1** |
| **151** | **1.457** | **0** | **0.012** | **15.38** | **0.0001** | **0.654** | **Purifying** | **nad1** |
| **152** | **0.85** | **0** | **0.029** | **9.627** | **0.0019** | **1.607** | **Purifying** | **nad1** |
| **153** | **1.139** | **0** | **0.022** | **13.348** | **0.0003** | **1.227** | **Purifying** | **nad1** |
| **154** | **1.536** | **0** | **0.455** | **13.699** | **0.0002** | **25.583** | **Purifying** | **nad1** |
| **155** | **2.356** | **0** | **0.526** | **14.533** | **0.0001** | **29.531** | **Purifying** | **nad1** |
| **156** | **3.336** | **0** | **0.019** | **26.157** | **0** | **1.063** | **Purifying** | **nad1** |
| **157** | **3.317** | **0** | **0.383** | **22.477** | **0** | **21.514** | **Purifying** | **nad1** |
| **158** | **0.816** | **0** | **0.015** | **10.565** | **0.0012** | **0.864** | **Purifying** | **nad1** |
| **159** | **0.656** | **0** | **0.008** | **5.429** | **0.0198** | **0.424** | **Purifying** | **nad1** |
| **160** | **2.003** | **0** | **0.018** | **17.225** | **0** | **1.038** | **Purifying** | **nad1** |
| **161** | **1.723** | **0** | **0.018** | **14.545** | **0.0001** | **1** | **Purifying** | **nad1** |
| **162** | **0.974** | **0** | **0.032** | **19.531** | **0** | **1.776** | **Purifying** | **nad1** |
| **163** | **4.466** | **0** | **0.433** | **31.135** | **0** | **24.314** | **Purifying** | **nad1** |
| **164** | **0.471** | **0** | **0.006** | **4.715** | **0.0299** | **0.339** | **Purifying** | **nad1** |
| **165** | **2.644** | **0** | **0.351** | **20.527** | **0** | **19.712** | **Purifying** | **nad1** |
| **166** | **1.646** | **0** | **0.716** | **14.614** | **0.0001** | **40.204** | **Purifying** | **nad1** |
| **167** | **2.947** | **0** | **0.012** | **10.117** | **0.0015** | **0.666** | **Purifying** | **nad1** |
| **168** | **0.722** | **0** | **0.007** | **5.027** | **0.025** | **0.401** | **Purifying** | **nad1** |
| **169** | **4.214** | **0** | **0.39** | **24.43** | **0** | **21.924** | **Purifying** | **nad1** |
| **170** | **4.551** | **0** | **0.015** | **17.297** | **0** | **0.849** | **Purifying** | **nad1** |
| **171** | **3.995** | **0.009** | **0.641** | **11.154** | **0.0008** | **36.012** | **Purifying** | **nad1** |
| **172** | **1.785** | **0** | **0.325** | **19.992** | **0** | **18.234** | **Purifying** | **nad1** |
| **173** | **0** | **0** | **0** | **0** | **1** | **0** | **Invariable** | **nad1** |
| **174** | **0** | **0** | **0** | **0** | **1** | **0** | **Invariable** | **nad1** |
| **175** | **1.111** | **0** | **0.006** | **8.59** | **0.0034** | **0.311** | **Purifying** | **nad1** |
| **176** | **1.86** | **0.01** | **0.708** | **9.456** | **0.0021** | **39.798** | **Purifying** | **nad1** |
| **177** | **1.856** | **0** | **0.566** | **16.123** | **0.0001** | **31.777** | **Purifying** | **nad1** |
| **178** | **1.908** | **0** | **0.369** | **18.18** | **0** | **20.741** | **Purifying** | **nad1** |
| **179** | **2.589** | **0** | **0.729** | **17.619** | **0** | **40.98** | **Purifying** | **nad1** |
| **180** | **1.526** | **0** | **0.021** | **13.291** | **0.0003** | **1.207** | **Purifying** | **nad1** |
| **181** | **1.613** | **0** | **0.299** | **19.255** | **0** | **16.786** | **Purifying** | **nad1** |
| **182** | **0** | **0** | **0** | **0** | **1** | **0** | **Invariable** | **nad1** |
| **183** | **0.809** | **0** | **0.028** | **9.382** | **0.0022** | **1.597** | **Purifying** | **nad1** |
| **184** | **0.783** | **0** | **0.012** | **10.772** | **0.001** | **0.694** | **Purifying** | **nad1** |
| **185** | **0** | **0** | **0** | **0** | **1** | **0** | **Invariable** | **nad1** |
| **186** | **0** | **0.356** | **0.415** | **2.15** | **0.1426** | **23.305** | **Neutral** | **nad1** |
| **187** | **1.098** | **0** | **0.019** | **14.178** | **0.0002** | **1.063** | **Purifying** | **nad1** |
| **188** | **1.499** | **0** | **0.33** | **16.951** | **0** | **18.549** | **Purifying** | **nad1** |
| **189** | **1.638** | **0** | **0.349** | **20.176** | **0** | **19.599** | **Purifying** | **nad1** |
| **190** | **1.429** | **0** | **0.018** | **13.881** | **0.0002** | **0.996** | **Purifying** | **nad1** |
| **191** | **1.334** | **0** | **0.029** | **11.422** | **0.0007** | **1.617** | **Purifying** | **nad1** |
| **192** | **0.675** | **0** | **0.006** | **5.143** | **0.0233** | **0.359** | **Purifying** | **nad1** |
| **193** | **1.684** | **0** | **0.019** | **12.683** | **0.0004** | **1.07** | **Purifying** | **nad1** |
| **194** | **0.533** | **0** | **0.017** | **9.106** | **0.0025** | **0.949** | **Purifying** | **nad1** |
| **195** | **0** | **0** | **0** | **0** | **1** | **0** | **Invariable** | **nad1** |
| **196** | **0.402** | **0** | **0.007** | **3.006** | **0.0829** | **0.421** | **Neutral** | **nad1** |
| **197** | **1.054** | **0** | **0.006** | **6.03** | **0.0141** | **0.346** | **Purifying** | **nad1** |
| **198** | **2.054** | **0** | **0.022** | **14.309** | **0.0002** | **1.215** | **Purifying** | **nad1** |
| **199** | **2.07** | **0** | **0.014** | **15.263** | **0.0001** | **0.8** | **Purifying** | **nad1** |
| **200** | **0** | **0** | **0** | **0** | **1** | **0** | **Invariable** | **nad1** |
| **201** | **3.171** | **0** | **1.075** | **18.819** | **0** | **60.393** | **Purifying** | **nad1** |
| **202** | **1.488** | **0** | **0.311** | **18.316** | **0** | **17.5** | **Purifying** | **nad1** |
| **203** | **3.196** | **0** | **0.022** | **19.18** | **0** | **1.24** | **Purifying** | **nad1** |
| **204** | **1.744** | **0** | **0.009** | **14.142** | **0.0002** | **0.525** | **Purifying** | **nad1** |
| **205** | **10000** | **0** | **0.023** | **25.572** | **0** | **1.274** | **Purifying** | **nad1** |
| **206** | **0** | **0** | **0** | **0** | **1** | **0** | **Invariable** | **nad1** |
| **207** | **0** | **0** | **0** | **0** | **1** | **0** | **Invariable** | **nad1** |
| **208** | **1.131** | **0** | **0.017** | **9.54** | **0.002** | **0.981** | **Purifying** | **nad1** |
| **209** | **1.029** | **0** | **0.014** | **11.901** | **0.0006** | **0.788** | **Purifying** | **nad1** |
| **210** | **0** | **0** | **0** | **0** | **1** | **0** | **Invariable** | **nad1** |
| **211** | **16.829** | **0** | **0.553** | **35.254** | **0** | **31.074** | **Purifying** | **nad1** |
| **212** | **0.498** | **0** | **0.006** | **4.813** | **0.0282** | **0.34** | **Purifying** | **nad1** |
| **213** | **0** | **0** | **0** | **0** | **1** | **0** | **Invariable** | **nad1** |
| **214** | **0** | **0** | **0** | **0** | **1** | **0** | **Invariable** | **nad1** |
| **215** | **10000** | **0** | **0.022** | **23.804** | **0** | **1.245** | **Purifying** | **nad1** |
| **216** | **1.31** | **0** | **0.02** | **15.39** | **0.0001** | **1.108** | **Purifying** | **nad1** |
| **217** | **0** | **0** | **0** | **0** | **1** | **0** | **Invariable** | **nad1** |
| **218** | **0.71** | **0** | **0.006** | **5.391** | **0.0202** | **0.325** | **Purifying** | **nad1** |
| **219** | **1.842** | **0** | **0.416** | **26.705** | **0** | **23.363** | **Purifying** | **nad1** |
| **220** | **10000** | **0** | **0.617** | **27.601** | **0** | **34.647** | **Purifying** | **nad1** |
| **221** | **1.019** | **0** | **0.007** | **7.965** | **0.0048** | **0.407** | **Purifying** | **nad1** |
| **222** | **0** | **0** | **0** | **0** | **1** | **0** | **Invariable** | **nad1** |
| **223** | **1.288** | **0** | **0.465** | **12.785** | **0.0003** | **26.123** | **Purifying** | **nad1** |
| **224** | **5.909** | **0** | **0.385** | **24.945** | **0** | **21.628** | **Purifying** | **nad1** |
| **225** | **1.019** | **0** | **0.007** | **7.965** | **0.0048** | **0.407** | **Purifying** | **nad1** |
| **226** | **1.305** | **0** | **0.464** | **12.656** | **0.0004** | **26.07** | **Purifying** | **nad1** |
| **227** | **0.795** | **0** | **0.016** | **9.566** | **0.002** | **0.878** | **Purifying** | **nad1** |
| **228** | **1.427** | **0.019** | **0.032** | **5.691** | **0.0171** | **1.807** | **Purifying** | **nad1** |
| **229** | **4.158** | **0.042** | **0.367** | **14.368** | **0.0002** | **20.601** | **Purifying** | **nad1** |
| **230** | **1.392** | **0.011** | **0.127** | **9.713** | **0.0018** | **7.108** | **Purifying** | **nad1** |
| **231** | **2.55** | **0.015** | **0.139** | **13.847** | **0.0002** | **7.808** | **Purifying** | **nad1** |
| **232** | **1.188** | **0.016** | **0.158** | **8.201** | **0.0042** | **8.872** | **Purifying** | **nad1** |
| **233** | **2.798** | **0.013** | **0.988** | **14.857** | **0.0001** | **55.518** | **Purifying** | **nad1** |
| **234** | **1.645** | **0.024** | **0.483** | **7.547** | **0.006** | **27.138** | **Purifying** | **nad1** |
| **235** | **0** | **0** | **0** | **0** | **1** | **0** | **Invariable** | **nad1** |
| **236** | **5.588** | **0** | **0.365** | **26.854** | **0** | **20.487** | **Purifying** | **nad1** |
| **237** | **0.583** | **0** | **0.007** | **4.717** | **0.0299** | **0.401** | **Purifying** | **nad1** |
| **238** | **1.367** | **0** | **0.228** | **12.722** | **0.0004** | **12.822** | **Purifying** | **nad1** |
| **239** | **1.757** | **0** | **0.351** | **20.353** | **0** | **19.719** | **Purifying** | **nad1** |
| **240** | **1.636** | **0** | **0.016** | **14.955** | **0.0001** | **0.889** | **Purifying** | **nad1** |
| **241** | **2.239** | **0** | **0.342** | **19.835** | **0** | **19.237** | **Purifying** | **nad1** |
| **242** | **3.647** | **0** | **0.793** | **18.12** | **0** | **44.527** | **Purifying** | **nad1** |
| **243** | **1.105** | **0** | **0.007** | **8.091** | **0.0044** | **0.408** | **Purifying** | **nad1** |
| **244** | **2.53** | **0** | **0.899** | **17.51** | **0** | **50.511** | **Purifying** | **nad1** |
| **245** | **1.193** | **0** | **0.013** | **14.328** | **0.0002** | **0.752** | **Purifying** | **nad1** |
| **246** | **0.636** | **0** | **0.006** | **5.382** | **0.0203** | **0.345** | **Purifying** | **nad1** |
| **247** | **1.229** | **0.304** | **0.518** | **1.487** | **0.2227** | **29.102** | **Neutral** | **nad1** |
| **248** | **4.742** | **0** | **0.022** | **18.627** | **0** | **1.256** | **Purifying** | **nad1** |
| **249** | **0.634** | **0.01** | **0.21** | **3.609** | **0.0575** | **11.775** | **Neutral** | **nad1** |
| **250** | **6.368** | **0** | **0.833** | **27.741** | **0** | **46.816** | **Purifying** | **nad1** |
| **251** | **0** | **0** | **0** | **0** | **1** | **0** | **Invariable** | **nad1** |
| **252** | **2.268** | **0.01** | **0.52** | **9.093** | **0.0026** | **29.24** | **Purifying** | **nad1** |
| **253** | **0** | **0** | **0** | **0** | **1** | **0** | **Invariable** | **nad1** |
| **254** | **3.889** | **0** | **0.006** | **8.812** | **0.003** | **0.322** | **Purifying** | **nad1** |
| **255** | **1.37** | **0.016** | **0.471** | **4.045** | **0.0443** | **26.479** | **Purifying** | **nad1** |
| **256** | **1.081** | **0** | **0.018** | **13.158** | **0.0003** | **0.992** | **Purifying** | **nad1** |
| **257** | **0.775** | **0** | **0.006** | **5.379** | **0.0204** | **0.36** | **Purifying** | **nad1** |
| **258** | **4.264** | **0.334** | **0.823** | **5.371** | **0.0205** | **46.249** | **Purifying** | **nad1** |
| **259** | **0.883** | **0** | **0.008** | **7.72** | **0.0055** | **0.43** | **Purifying** | **nad1** |
| **260** | **1.054** | **0** | **0.029** | **10.277** | **0.0013** | **1.615** | **Purifying** | **nad1** |
| **261** | **0** | **0.47** | **0.532** | **1.728** | **0.1886** | **29.903** | **Neutral** | **nad1** |
| **262** | **0.779** | **0** | **0.014** | **10.417** | **0.0012** | **0.764** | **Purifying** | **nad1** |
| **263** | **0** | **0** | **0** | **0** | **1** | **0** | **Invariable** | **nad1** |
| **264** | **0.617** | **0** | **0.006** | **4.988** | **0.0255** | **0.359** | **Purifying** | **nad1** |
| **265** | **0.513** | **0** | **0.006** | **5.014** | **0.0251** | **0.345** | **Purifying** | **nad1** |
| **266** | **5.554** | **0** | **0.938** | **21.621** | **0** | **52.713** | **Purifying** | **nad1** |
| **267** | **2.633** | **0** | **0.727** | **16.498** | **0** | **40.829** | **Purifying** | **nad1** |
| **268** | **1.358** | **0** | **0.019** | **13.435** | **0.0002** | **1.04** | **Purifying** | **nad1** |
| **269** | **1.091** | **0.03** | **0.361** | **4.136** | **0.042** | **20.259** | **Purifying** | **nad1** |
| **270** | **2.384** | **0.658** | **1.304** | **2.283** | **0.1308** | **73.24** | **Neutral** | **nad1** |
| **271** | **0.886** | **0** | **0.007** | **7.77** | **0.0053** | **0.407** | **Purifying** | **nad1** |
| **272** | **3.442** | **0** | **0.718** | **17.648** | **0** | **40.319** | **Purifying** | **nad1** |
| **273** | **3.392** | **0** | **0.53** | **22.621** | **0** | **29.768** | **Purifying** | **nad1** |
| **274** | **0.469** | **0** | **0.008** | **3.134** | **0.0767** | **0.421** | **Neutral** | **nad1** |
| **275** | **0** | **0** | **0** | **0** | **1** | **0** | **Invariable** | **nad1** |
| **276** | **1.43** | **0** | **0.016** | **14.028** | **0.0002** | **0.89** | **Purifying** | **nad1** |
| **277** | **0.883** | **0** | **0.008** | **7.5** | **0.0062** | **0.462** | **Purifying** | **nad1** |
| **278** | **0.624** | **0** | **0.008** | **4.482** | **0.0343** | **0.459** | **Purifying** | **nad1** |
| **279** | **0** | **0** | **0** | **0** | **1** | **0** | **Invariable** | **nad1** |
| **280** | **2.216** | **0** | **0.019** | **17.517** | **0** | **1.039** | **Purifying** | **nad1** |
| **281** | **1.138** | **0** | **0.017** | **11.452** | **0.0007** | **0.954** | **Purifying** | **nad1** |
| **282** | **1.13** | **0** | **0.019** | **14.245** | **0.0002** | **1.064** | **Purifying** | **nad1** |
| **283** | **12.546** | **0** | **0.303** | **29.238** | **0** | **17.005** | **Purifying** | **nad1** |
| **284** | **0.767** | **0** | **0.022** | **11.878** | **0.0006** | **1.229** | **Purifying** | **nad1** |
| **285** | **2.428** | **0** | **0.653** | **17.873** | **0** | **36.674** | **Purifying** | **nad1** |
| **286** | **0** | **0.024** | **0.019** | **0.522** | **0.4702** | **1.067** | **Neutral** | **nad1** |
| **287** | **1.261** | **0** | **0.007** | **6.062** | **0.0138** | **0.404** | **Purifying** | **nad1** |
| **288** | **0.261** | **0** | **0.018** | **6.291** | **0.0121** | **1.024** | **Purifying** | **nad1** |
| **289** | **1.902** | **0** | **0.016** | **13.433** | **0.0002** | **0.878** | **Purifying** | **nad1** |
| **290** | **0** | **0** | **0** | **0** | **1** | **0** | **Invariable** | **nad1** |
| **291** | **2.454** | **0** | **0.02** | **17.242** | **0** | **1.108** | **Purifying** | **nad1** |
| **292** | **10000** | **0** | **0.384** | **25.44** | **0** | **21.58** | **Purifying** | **nad1** |
| **293** | **0.455** | **0** | **0.009** | **3.679** | **0.0551** | **0.491** | **Neutral** | **nad1** |
| **294** | **0.573** | **0** | **0.008** | **4.719** | **0.0298** | **0.437** | **Purifying** | **nad1** |
| **295** | **0.392** | **0** | **0.031** | **7.764** | **0.0053** | **1.763** | **Purifying** | **nad1** |
| **296** | **0** | **0** | **0** | **0** | **1** | **0** | **Invariable** | **nad1** |
| **297** | **0.022** | **0** | **0.007** | **2.01** | **0.1562** | **0.393** | **Neutral** | **nad1** |
| **298** | **0.547** | **0** | **0.006** | **5.116** | **0.0237** | **0.345** | **Purifying** | **nad1** |
| **299** | **1.037** | **0** | **0.017** | **9.607** | **0.0019** | **0.978** | **Purifying** | **nad1** |
| **300** | **0.029** | **0** | **0.006** | **2.55** | **0.1103** | **0.348** | **Neutral** | **nad1** |
| **301** | **0.571** | **0** | **0.008** | **5.233** | **0.0222** | **0.423** | **Purifying** | **nad1** |
| **302** | **4.264** | **0** | **0.022** | **17.466** | **0** | **1.251** | **Purifying** | **nad1** |
| **303** | **1.26** | **0** | **0.025** | **11.702** | **0.0006** | **1.428** | **Purifying** | **nad1** |
| **304** | **0.802** | **0** | **0.009** | **8.431** | **0.0037** | **0.53** | **Purifying** | **nad1** |
| **305** | **0.376** | **0** | **0.009** | **3.525** | **0.0604** | **0.489** | **Neutral** | **nad1** |
| **306** | **3.658** | **0** | **0.522** | **22.096** | **0** | **29.318** | **Purifying** | **nad1** |
| **307** | **1.213** | **0** | **0.179** | **14.796** | **0.0001** | **10.051** | **Purifying** | **nad1** |
| **308** | **0** | **0** | **0** | **0** | **1** | **0** | **Invariable** | **nad1** |
| **309** | **3.555** | **0** | **0.368** | **23.571** | **0** | **20.659** | **Purifying** | **nad1** |
| **310** | **1.613** | **0** | **0.322** | **19.569** | **0** | **18.106** | **Purifying** | **nad1** |
| **311** | **1.929** | **0** | **0.023** | **22.989** | **0** | **1.273** | **Purifying** | **nad1** |
| **312** | **0.749** | **0** | **0.017** | **8.473** | **0.0036** | **0.969** | **Purifying** | **nad1** |
| **313** | **1.75** | **0** | **0.399** | **17.998** | **0** | **22.42** | **Purifying** | **nad1** |
| **314** | **2.147** | **0** | **0.432** | **19.037** | **0** | **24.257** | **Purifying** | **nad1** |
| **315** | **3.665** | **0** | **0.029** | **18.53** | **0** | **1.628** | **Purifying** | **nad1** |
| **316** | **0** | **0** | **0** | **0** | **1** | **0** | **Invariable** | **nad1** |
| **317** | **0** | **0** | **0** | **0** | **1** | **0** | **Invariable** | **nad2** |
| **318** | **0** | **0** | **0** | **0** | **1** | **0** | **Invariable** | **nad2** |
| **319** | **0.01** | **0** | **0.005** | **1.423** | **0.2329** | **0.277** | **Neutral** | **nad2** |
| **320** | **0** | **0.01** | **0.008** | **0.422** | **0.5158** | **0.463** | **Neutral** | **nad2** |
| **321** | **0.504** | **0** | **0.007** | **4.871** | **0.0273** | **0.376** | **Purifying** | **nad2** |
| **322** | **0** | **0** | **0** | **0** | **1** | **0** | **Invariable** | **nad2** |
| **323** | **0** | **0** | **0** | **0** | **1** | **0** | **Invariable** | **nad2** |
| **324** | **1.568** | **0.01** | **0.387** | **6.321** | **0.0119** | **21.764** | **Purifying** | **nad2** |
| **325** | **1.118** | **0** | **0.025** | **11.019** | **0.0009** | **1.433** | **Purifying** | **nad2** |
| **326** | **0.814** | **0** | **0.45** | **11.631** | **0.0006** | **25.284** | **Purifying** | **nad2** |
| **327** | **0** | **0** | **0** | **0** | **1** | **0** | **Invariable** | **nad2** |
| **328** | **1.19** | **0** | **0.022** | **18.253** | **0** | **1.212** | **Purifying** | **nad2** |
| **329** | **0.843** | **0** | **0.029** | **9.53** | **0.002** | **1.602** | **Purifying** | **nad2** |
| **330** | **0** | **0** | **0** | **0** | **1** | **0** | **Invariable** | **nad2** |
| **331** | **0** | **0** | **0** | **0** | **1** | **0** | **Invariable** | **nad2** |
| **332** | **0** | **0** | **0** | **0** | **1** | **0** | **Invariable** | **nad2** |
| **333** | **0.023** | **0** | **0.006** | **2.396** | **0.1216** | **0.322** | **Neutral** | **nad2** |
| **334** | **0** | **0** | **0** | **0** | **1** | **0** | **Invariable** | **nad2** |
| **335** | **0.029** | **0** | **0.006** | **2.548** | **0.1105** | **0.348** | **Neutral** | **nad2** |
| **336** | **0** | **0** | **0** | **0** | **1** | **0** | **Invariable** | **nad2** |
| **337** | **0** | **0** | **0** | **0** | **1** | **0** | **Invariable** | **nad2** |
| **338** | **0** | **0** | **0** | **0** | **1** | **0** | **Invariable** | **nad2** |
| **339** | **0** | **0** | **0** | **0** | **1** | **0** | **Invariable** | **nad2** |
| **340** | **0** | **0** | **0** | **0** | **1** | **0** | **Invariable** | **nad2** |
| **341** | **1.261** | **0** | **0.007** | **6.062** | **0.0138** | **0.404** | **Purifying** | **nad2** |
| **342** | **1.346** | **0** | **0.499** | **14.804** | **0.0001** | **28.029** | **Purifying** | **nad2** |
| **343** | **1.184** | **0** | **0.224** | **12.344** | **0.0004** | **12.564** | **Purifying** | **nad2** |
| **344** | **0** | **0** | **0** | **0** | **1** | **0** | **Invariable** | **nad2** |
| **345** | **0** | **0.01** | **0.008** | **0.297** | **0.5857** | **0.446** | **Neutral** | **nad2** |
| **346** | **0** | **0** | **0** | **0** | **1** | **0** | **Invariable** | **nad2** |
| **347** | **0.022** | **0** | **0.007** | **2.009** | **0.1564** | **0.393** | **Neutral** | **nad2** |
| **348** | **0** | **0** | **0** | **0** | **1** | **0** | **Invariable** | **nad2** |
| **349** | **0** | **0** | **0** | **0** | **1** | **0** | **Invariable** | **nad2** |
| **350** | **9.665** | **0** | **0.413** | **28.911** | **0** | **23.211** | **Purifying** | **nad2** |
| **351** | **0** | **0** | **0** | **0** | **1** | **0** | **Invariable** | **nad2** |
| **352** | **0.361** | **0** | **0.023** | **5.31** | **0.0212** | **1.273** | **Purifying** | **nad2** |
| **353** | **0** | **0** | **0** | **0** | **1** | **0** | **Invariable** | **nad2** |
| **354** | **0.655** | **0** | **0.008** | **5.431** | **0.0198** | **0.424** | **Purifying** | **nad2** |
| **355** | **0.579** | **0** | **0.008** | **5.269** | **0.0217** | **0.423** | **Purifying** | **nad2** |
| **356** | **0.545** | **0** | **0.008** | **4.65** | **0.031** | **0.436** | **Purifying** | **nad2** |
| **357** | **3.349** | **0** | **0.776** | **19.408** | **0** | **43.575** | **Purifying** | **nad2** |
| **358** | **0** | **0** | **0** | **0** | **1** | **0** | **Invariable** | **nad2** |
| **359** | **0** | **0** | **0** | **0** | **1** | **0** | **Invariable** | **nad2** |
| **360** | **10000** | **0** | **0.012** | **12.085** | **0.0005** | **0.665** | **Purifying** | **nad2** |
| **361** | **1.993** | **0** | **0.022** | **16.449** | **0** | **1.25** | **Purifying** | **nad2** |
| **362** | **0.883** | **0** | **0.009** | **7.423** | **0.0064** | **0.495** | **Purifying** | **nad2** |
| **363** | **0** | **0** | **0** | **0** | **1** | **0** | **Invariable** | **nad2** |
| **364** | **0.69** | **0** | **0.008** | **4.651** | **0.031** | **0.46** | **Purifying** | **nad2** |
| **365** | **0.44** | **0** | **0.008** | **3.498** | **0.0614** | **0.424** | **Neutral** | **nad2** |
| **366** | **0** | **0** | **0** | **0** | **1** | **0** | **Invariable** | **nad2** |
| **367** | **0.404** | **0** | **0.006** | **2.508** | **0.1133** | **0.317** | **Neutral** | **nad2** |
| **368** | **0.608** | **0** | **0.008** | **7.989** | **0.0047** | **0.454** | **Purifying** | **nad2** |
| **369** | **0** | **0** | **0** | **0** | **1** | **0** | **Invariable** | **nad2** |
| **370** | **0.987** | **0** | **0.018** | **12.848** | **0.0003** | **0.99** | **Purifying** | **nad2** |
| **371** | **0.023** | **0** | **0.006** | **2.416** | **0.1201** | **0.323** | **Neutral** | **nad2** |
| **372** | **3.877** | **0** | **0.009** | **9.756** | **0.0018** | **0.483** | **Purifying** | **nad2** |
| **373** | **1.173** | **0** | **0.031** | **17.014** | **0** | **1.731** | **Purifying** | **nad2** |
| **374** | **0** | **0** | **0** | **0** | **1** | **0** | **Invariable** | **nad2** |
| **375** | **2.816** | **0** | **0.508** | **16.928** | **0** | **28.517** | **Purifying** | **nad2** |
| **376** | **1.771** | **0** | **0.019** | **14.348** | **0.0002** | **1.061** | **Purifying** | **nad2** |
| **377** | **10000** | **0** | **0.012** | **11.291** | **0.0008** | **0.665** | **Purifying** | **nad2** |
| **378** | **0** | **0** | **0** | **0** | **1** | **0** | **Invariable** | **nad2** |
| **379** | **1.748** | **0** | **0.019** | **14.65** | **0.0001** | **1.064** | **Purifying** | **nad2** |
| **380** | **0.181** | **0** | **0.006** | **4.107** | **0.0427** | **0.356** | **Purifying** | **nad2** |
| **381** | **0.039** | **0** | **0.006** | **3.35** | **0.0672** | **0.338** | **Neutral** | **nad2** |
| **382** | **0.535** | **0** | **0.006** | **5.065** | **0.0244** | **0.345** | **Purifying** | **nad2** |
| **383** | **0** | **0.01** | **0.008** | **0.297** | **0.5857** | **0.446** | **Neutral** | **nad2** |
| **384** | **0.963** | **0** | **0.025** | **10.708** | **0.0011** | **1.43** | **Purifying** | **nad2** |
| **385** | **0.431** | **0** | **0.009** | **3.634** | **0.0566** | **0.491** | **Neutral** | **nad2** |
| **386** | **0** | **0** | **0** | **0** | **1** | **0** | **Invariable** | **nad2** |
| **387** | **0.742** | **0** | **0.006** | **5.31** | **0.0212** | **0.36** | **Purifying** | **nad2** |
| **388** | **1.074** | **0** | **0.005** | **9.077** | **0.0026** | **0.301** | **Purifying** | **nad2** |
| **389** | **0** | **0** | **0** | **0** | **1** | **0** | **Invariable** | **nad2** |
| **390** | **1.295** | **0** | **0.308** | **17.911** | **0** | **17.279** | **Purifying** | **nad2** |
| **391** | **5.81** | **0** | **0.435** | **27.739** | **0** | **24.432** | **Purifying** | **nad2** |
| **392** | **3.024** | **0** | **0.321** | **21.976** | **0** | **18.046** | **Purifying** | **nad2** |
| **393** | **4.147** | **0** | **1.055** | **20.223** | **0** | **59.264** | **Purifying** | **nad2** |
| **394** | **0** | **0.009** | **0.007** | **0.508** | **0.4759** | **0.403** | **Neutral** | **nad2** |
| **395** | **0** | **0** | **0** | **0** | **1** | **0** | **Invariable** | **nad2** |
| **396** | **0** | **0** | **0** | **0** | **1** | **0** | **Invariable** | **nad2** |
| **397** | **3.68** | **0** | **0.018** | **18.306** | **0** | **0.997** | **Purifying** | **nad2** |
| **398** | **1.19** | **0** | **0.022** | **13.419** | **0.0002** | **1.227** | **Purifying** | **nad2** |
| **399** | **0** | **0.014** | **0.012** | **0.335** | **0.5628** | **0.664** | **Neutral** | **nad2** |
| **400** | **3.984** | **0** | **0.719** | **26.95** | **0** | **40.398** | **Purifying** | **nad2** |
| **401** | **0** | **0.105** | **0.024** | **1.157** | **0.282** | **1.333** | **Neutral** | **nad2** |
| **402** | **2.017** | **0** | **0.353** | **20.827** | **0** | **19.846** | **Purifying** | **nad2** |
| **403** | **0.049** | **0** | **0.008** | **3.135** | **0.0766** | **0.452** | **Neutral** | **nad2** |
| **404** | **0** | **0** | **0** | **0** | **1** | **0** | **Invariable** | **nad2** |
| **405** | **1.376** | **0** | **0.025** | **11.717** | **0.0006** | **1.424** | **Purifying** | **nad2** |
| **406** | **0.737** | **0** | **0.008** | **4.824** | **0.0281** | **0.46** | **Purifying** | **nad2** |
| **407** | **3.543** | **0** | **0.415** | **20.576** | **0** | **23.316** | **Purifying** | **nad2** |
| **408** | **0.513** | **0** | **0.007** | **4.85** | **0.0277** | **0.383** | **Purifying** | **nad2** |
| **409** | **0** | **0.021** | **0.013** | **0.36** | **0.5486** | **0.739** | **Neutral** | **nad2** |
| **410** | **2.165** | **0** | **0.388** | **18.799** | **0** | **21.823** | **Purifying** | **nad2** |
| **411** | **1.338** | **0.009** | **0.383** | **7.96** | **0.0048** | **21.517** | **Purifying** | **nad2** |
| **412** | **5.774** | **0.01** | **1.099** | **16.964** | **0** | **61.753** | **Purifying** | **nad2** |
| **413** | **0.775** | **0** | **0.006** | **5.379** | **0.0204** | **0.36** | **Purifying** | **nad2** |
| **414** | **1.883** | **0** | **0.766** | **15.846** | **0.0001** | **43.04** | **Purifying** | **nad2** |
| **415** | **0.636** | **0** | **0.006** | **5.382** | **0.0203** | **0.345** | **Purifying** | **nad2** |
| **416** | **0.313** | **0** | **0.023** | **6.099** | **0.0135** | **1.318** | **Purifying** | **nad2** |
| **417** | **0** | **0** | **0** | **0** | **1** | **0** | **Invariable** | **nad2** |
| **418** | **0.029** | **0** | **0.006** | **2.549** | **0.1104** | **0.348** | **Neutral** | **nad2** |
| **419** | **2.252** | **0** | **0.017** | **24.391** | **0** | **0.939** | **Purifying** | **nad2** |
| **420** | **0.839** | **0** | **0.034** | **10.373** | **0.0013** | **1.929** | **Purifying** | **nad2** |
| **421** | **1.385** | **0** | **0.016** | **14.444** | **0.0001** | **0.886** | **Purifying** | **nad2** |
| **422** | **3.868** | **0** | **0.574** | **22.28** | **0** | **32.264** | **Purifying** | **nad2** |
| **423** | **1.508** | **0** | **0.028** | **11.261** | **0.0008** | **1.584** | **Purifying** | **nad2** |
| **424** | **0.883** | **0** | **0.009** | **7.423** | **0.0064** | **0.495** | **Purifying** | **nad2** |
| **425** | **2.429** | **0** | **0.018** | **14.457** | **0.0001** | **1.036** | **Purifying** | **nad2** |
| **426** | **1.888** | **0** | **0.453** | **14.665** | **0.0001** | **25.44** | **Purifying** | **nad2** |
| **427** | **0** | **0** | **0** | **0** | **1** | **0** | **Invariable** | **nad2** |
| **428** | **8.809** | **0** | **0.022** | **20.257** | **0** | **1.239** | **Purifying** | **nad2** |
| **429** | **4.698** | **0** | **0.593** | **23.602** | **0** | **33.302** | **Purifying** | **nad2** |
| **430** | **0.455** | **0** | **0.009** | **3.679** | **0.0551** | **0.491** | **Neutral** | **nad2** |
| **431** | **8.832** | **0** | **0.012** | **9.742** | **0.0018** | **0.664** | **Purifying** | **nad2** |
| **432** | **2.404** | **0** | **0.358** | **27.419** | **0** | **20.09** | **Purifying** | **nad2** |
| **433** | **3.723** | **0** | **1.234** | **21.214** | **0** | **69.315** | **Purifying** | **nad2** |
| **434** | **0.987** | **0** | **0.007** | **8.022** | **0.0046** | **0.384** | **Purifying** | **nad2** |
| **435** | **1.367** | **0** | **0.579** | **13.423** | **0.0002** | **32.557** | **Purifying** | **nad2** |
| **436** | **2.186** | **0** | **0.354** | **20.031** | **0** | **19.895** | **Purifying** | **nad2** |
| **437** | **0** | **0** | **0** | **0** | **1** | **0** | **Invariable** | **nad2** |
| **438** | **8.829** | **0** | **0.557** | **39.438** | **0** | **31.313** | **Purifying** | **nad2** |
| **439** | **0** | **0** | **0** | **0** | **1** | **0** | **Invariable** | **nad2** |
| **440** | **1.405** | **0.01** | **0.382** | **6.175** | **0.013** | **21.461** | **Purifying** | **nad2** |
| **441** | **1.064** | **0** | **0.025** | **11.091** | **0.0009** | **1.413** | **Purifying** | **nad2** |
| **442** | **0.742** | **0** | **0.008** | **4.968** | **0.0258** | **0.423** | **Purifying** | **nad2** |
| **443** | **0.952** | **0** | **0.014** | **11.061** | **0.0009** | **0.767** | **Purifying** | **nad2** |
| **444** | **1.669** | **0** | **0.012** | **8.68** | **0.0032** | **0.662** | **Purifying** | **nad2** |
| **445** | **0** | **0** | **0** | **0** | **1** | **0** | **Invariable** | **nad2** |
| **446** | **1.215** | **0** | **0.59** | **13.128** | **0.0003** | **33.166** | **Purifying** | **nad2** |
| **447** | **2.663** | **0** | **0.317** | **20.982** | **0** | **17.799** | **Purifying** | **nad2** |
| **448** | **0** | **0** | **0** | **0** | **1** | **0** | **Invariable** | **nad2** |
| **449** | **444.287** | **0** | **0.408** | **27.973** | **0** | **22.899** | **Purifying** | **nad2** |
| **450** | **0** | **0** | **0** | **0** | **1** | **0** | **Invariable** | **nad2** |
| **451** | **0.995** | **0.011** | **0.447** | **4.514** | **0.0336** | **25.131** | **Purifying** | **nad2** |
| **452** | **7.164** | **0** | **0.985** | **24.198** | **0** | **55.35** | **Purifying** | **nad2** |
| **453** | **3.45** | **0** | **1.198** | **20.061** | **0** | **67.334** | **Purifying** | **nad2** |
| **454** | **5.453** | **0** | **0.547** | **25.895** | **0** | **30.732** | **Purifying** | **nad2** |
| **455** | **1.459** | **0.498** | **0.845** | **1.164** | **0.2806** | **47.5** | **Neutral** | **nad2** |
| **456** | **2.501** | **0** | **0.025** | **11.875** | **0.0006** | **1.386** | **Purifying** | **nad2** |
| **457** | **4.07** | **0** | **0.601** | **23.037** | **0** | **33.758** | **Purifying** | **nad2** |
| **458** | **1.631** | **0** | **0.018** | **14.644** | **0.0001** | **1** | **Purifying** | **nad2** |
| **459** | **2.938** | **0** | **0.02** | **18.067** | **0** | **1.109** | **Purifying** | **nad2** |
| **460** | **0** | **0** | **0** | **0** | **1** | **0** | **Invariable** | **nad2** |
| **461** | **0.545** | **0.575** | **0.567** | **0.002** | **0.9656** | **31.838** | **Neutral** | **nad2** |
| **462** | **1.345** | **0.016** | **0.451** | **4.533** | **0.0333** | **25.355** | **Purifying** | **nad2** |
| **463** | **2.315** | **0** | **0.862** | **17.022** | **0** | **48.446** | **Purifying** | **nad2** |
| **464** | **0.795** | **0** | **0.056** | **13.454** | **0.0002** | **3.145** | **Purifying** | **nad2** |
| **465** | **10.456** | **0.293** | **0.525** | **5.235** | **0.0221** | **29.523** | **Purifying** | **nad2** |
| **466** | **2.013** | **0** | **0.521** | **14.195** | **0.0002** | **29.249** | **Purifying** | **nad2** |
| **467** | **1.518** | **0** | **0.012** | **15.546** | **0.0001** | **0.653** | **Purifying** | **nad2** |
| **468** | **2.113** | **0** | **0.722** | **15.461** | **0.0001** | **40.542** | **Purifying** | **nad2** |
| **469** | **0** | **0.014** | **0.009** | **0.672** | **0.4122** | **0.526** | **Neutral** | **nad2** |
| **470** | **0.635** | **0** | **0.007** | **5.204** | **0.0225** | **0.372** | **Purifying** | **nad2** |
| **471** | **0.589** | **0** | **0.007** | **5.031** | **0.0249** | **0.384** | **Purifying** | **nad2** |
| **472** | **2.071** | **0** | **0.672** | **14.868** | **0.0001** | **37.782** | **Purifying** | **nad2** |
| **473** | **2.009** | **0.352** | **0.739** | **2.962** | **0.0852** | **41.542** | **Neutral** | **nad2** |
| **474** | **0.552** | **0** | **0.006** | **5** | **0.0253** | **0.324** | **Purifying** | **nad2** |
| **475** | **3.298** | **0** | **0.027** | **26.084** | **0** | **1.529** | **Purifying** | **nad2** |
| **476** | **0.627** | **0** | **0.006** | **5.178** | **0.0229** | **0.325** | **Purifying** | **nad2** |
| **477** | **3.64** | **0** | **0.413** | **30.318** | **0** | **23.225** | **Purifying** | **nad2** |
| **478** | **3.142** | **0** | **0.719** | **17.424** | **0** | **40.42** | **Purifying** | **nad2** |
| **479** | **10000** | **0** | **0.553** | **32.866** | **0** | **31.085** | **Purifying** | **nad2** |
| **480** | **3.892** | **0** | **0.012** | **9.098** | **0.0026** | **0.663** | **Purifying** | **nad2** |
| **481** | **1.411** | **0** | **0.019** | **13.71** | **0.0002** | **1.056** | **Purifying** | **nad2** |
| **482** | **3.183** | **0** | **0.029** | **17.773** | **0** | **1.619** | **Purifying** | **nad2** |
| **483** | **0.848** | **0** | **0.187** | **10.591** | **0.0011** | **10.522** | **Purifying** | **nad2** |
| **484** | **0.437** | **0** | **0.007** | **3.073** | **0.0796** | **0.421** | **Neutral** | **nad2** |
| **485** | **0** | **0** | **0** | **0** | **1** | **0** | **Invariable** | **nad2** |
| **486** | **2.925** | **0** | **0.554** | **21.938** | **0** | **31.102** | **Purifying** | **nad2** |
| **487** | **1.666** | **0** | **0.662** | **14.221** | **0.0002** | **37.177** | **Purifying** | **nad2** |
| **488** | **0.595** | **0** | **0.006** | **5.234** | **0.0222** | **0.345** | **Purifying** | **nad2** |
| **489** | **3.29** | **0** | **0.032** | **25.129** | **0** | **1.79** | **Purifying** | **nad2** |
| **490** | **2.101** | **0** | **0.019** | **14.566** | **0.0001** | **1.043** | **Purifying** | **nad2** |
| **491** | **0.772** | **0** | **0.008** | **5.037** | **0.0248** | **0.424** | **Purifying** | **nad2** |
| **492** | **1.93** | **0** | **0.364** | **18.119** | **0** | **20.438** | **Purifying** | **nad2** |
| **493** | **1.623** | **0** | **0.016** | **14.52** | **0.0001** | **0.886** | **Purifying** | **nad2** |
| **494** | **3.54** | **0** | **0.486** | **24.968** | **0** | **27.279** | **Purifying** | **nad2** |
| **495** | **0.861** | **0** | **0.017** | **8.703** | **0.0032** | **0.978** | **Purifying** | **nad2** |
| **496** | **3.515** | **0** | **0.02** | **26.393** | **0** | **1.112** | **Purifying** | **nad2** |
| **497** | **0** | **0.491** | **0.548** | **1.749** | **0.186** | **30.81** | **Neutral** | **nad2** |
| **498** | **0.953** | **0** | **0.022** | **12.585** | **0.0004** | **1.209** | **Purifying** | **nad2** |
| **499** | **2.181** | **0** | **0.82** | **16.985** | **0** | **46.086** | **Purifying** | **nad2** |
| **500** | **1.496** | **0.01** | **0.395** | **6.427** | **0.0112** | **22.212** | **Purifying** | **nad2** |
| **501** | **0.769** | **0** | **0.204** | **10.469** | **0.0012** | **11.442** | **Purifying** | **nad2** |
| **502** | **1.669** | **0** | **0.012** | **8.68** | **0.0032** | **0.662** | **Purifying** | **nad2** |
| **503** | **116.502** | **0.008** | **0.809** | **23.581** | **0** | **45.437** | **Purifying** | **nad2** |
| **504** | **1.18** | **0** | **0.018** | **12.728** | **0.0004** | **1.031** | **Purifying** | **nad2** |
| **505** | **2.426** | **0** | **0.407** | **19.211** | **0** | **22.86** | **Purifying** | **nad2** |
| **506** | **1.755** | **0** | **0.019** | **14.465** | **0.0001** | **1.046** | **Purifying** | **nad2** |
| **507** | **1.414** | **0** | **0.405** | **14.046** | **0.0002** | **22.741** | **Purifying** | **nad2** |
| **508** | **3.035** | **0** | **0.505** | **23.05** | **0** | **28.367** | **Purifying** | **nad2** |
| **509** | **0** | **0** | **0** | **0** | **1** | **0** | **Invariable** | **nad2** |
| **510** | **1.923** | **0** | **0.581** | **16.184** | **0.0001** | **32.62** | **Purifying** | **nad2** |
| **511** | **2.682** | **0** | **0.375** | **21.195** | **0** | **21.047** | **Purifying** | **nad2** |
| **512** | **1.734** | **0** | **0.584** | **15.244** | **0.0001** | **32.788** | **Purifying** | **nad2** |
| **513** | **1.666** | **0** | **0.585** | **14.175** | **0.0002** | **32.866** | **Purifying** | **nad2** |
| **514** | **0** | **0.008** | **0.007** | **0.465** | **0.4953** | **0.373** | **Neutral** | **nad2** |
| **515** | **4.688** | **0** | **0.394** | **27.472** | **0** | **22.144** | **Purifying** | **nad2** |
| **516** | **2.136** | **0** | **0.332** | **21.548** | **0** | **18.68** | **Purifying** | **nad2** |
| **517** | **1.571** | **0.01** | **0.418** | **6.416** | **0.0113** | **23.508** | **Purifying** | **nad2** |
| **518** | **0** | **0.304** | **0.421** | **1.707** | **0.1913** | **23.671** | **Neutral** | **nad2** |
| **519** | **1.312** | **0** | **0.019** | **13.383** | **0.0003** | **1.054** | **Purifying** | **nad2** |
| **520** | **0** | **0** | **0** | **0** | **1** | **0** | **Invariable** | **nad2** |
| **521** | **0.059** | **0** | **0.006** | **3.383** | **0.0659** | **0.364** | **Neutral** | **nad2** |
| **522** | **0** | **0** | **0** | **0** | **1** | **0** | **Invariable** | **nad2** |
| **523** | **0.886** | **0** | **0.018** | **11.911** | **0.0006** | **0.99** | **Purifying** | **nad2** |
| **524** | **0** | **0.014** | **0.007** | **1.149** | **0.2837** | **0.392** | **Neutral** | **nad2** |
| **525** | **1.919** | **0** | **0.021** | **14.68** | **0.0001** | **1.192** | **Purifying** | **nad2** |
| **526** | **3.929** | **0** | **0.487** | **25.607** | **0** | **27.381** | **Purifying** | **nad2** |
| **527** | **10.515** | **0** | **0.57** | **32.313** | **0** | **32.048** | **Purifying** | **nad2** |
| **528** | **0.254** | **0** | **0.009** | **3.347** | **0.0673** | **0.488** | **Neutral** | **nad2** |
| **529** | **0** | **0** | **0** | **0** | **1** | **0** | **Invariable** | **nad2** |
| **530** | **3.473** | **0** | **0.009** | **10.129** | **0.0015** | **0.484** | **Purifying** | **nad2** |
| **531** | **0.714** | **0.009** | **0.016** | **2.877** | **0.0899** | **0.897** | **Neutral** | **nad2** |
| **532** | **2.267** | **0** | **0.338** | **20.175** | **0** | **18.983** | **Purifying** | **nad2** |
| **533** | **0.154** | **0** | **0.007** | **4.789** | **0.0286** | **0.42** | **Purifying** | **nad2** |
| **534** | **1.886** | **0** | **0.39** | **18.454** | **0** | **21.899** | **Purifying** | **nad2** |
| **535** | **10000** | **0** | **0.392** | **31.701** | **0** | **22.026** | **Purifying** | **nad2** |
| **536** | **0.722** | **0** | **0.014** | **6.887** | **0.0087** | **0.787** | **Purifying** | **nad2** |
| **537** | **1.429** | **0** | **0.026** | **11.874** | **0.0006** | **1.441** | **Purifying** | **nad2** |
| **538** | **0.665** | **0** | **0.006** | **5.47** | **0.0194** | **0.346** | **Purifying** | **nad2** |
| **539** | **0.542** | **0** | **0.017** | **9.128** | **0.0025** | **0.95** | **Purifying** | **nad2** |
| **540** | **1.161** | **0** | **0.016** | **13.395** | **0.0003** | **0.888** | **Purifying** | **nad2** |
| **541** | **2.349** | **0** | **0.329** | **21.495** | **0** | **18.485** | **Purifying** | **nad2** |
| **542** | **10.279** | **0** | **0.009** | **11.069** | **0.0009** | **0.485** | **Purifying** | **nad2** |
| **543** | **0.536** | **0** | **0.006** | **5.1** | **0.0239** | **0.345** | **Purifying** | **nad2** |
| **544** | **2.203** | **0** | **0.396** | **18.908** | **0** | **22.245** | **Purifying** | **nad2** |
| **545** | **0.569** | **0.287** | **0.351** | **0.207** | **0.6487** | **19.697** | **Neutral** | **nad2** |
| **546** | **3.404** | **0** | **0.728** | **19.389** | **0** | **40.897** | **Purifying** | **nad2** |
| **547** | **0.713** | **0** | **0.007** | **5.03** | **0.0249** | **0.401** | **Purifying** | **nad2** |
| **548** | **0** | **0** | **0** | **0** | **1** | **0** | **Invariable** | **nad2** |
| **549** | **4.75** | **0** | **0.701** | **19.009** | **0** | **39.382** | **Purifying** | **nad2** |
| **550** | **1.046** | **0** | **0.026** | **14.659** | **0.0001** | **1.47** | **Purifying** | **nad2** |
| **551** | **1.55** | **0** | **0.02** | **13.645** | **0.0002** | **1.127** | **Purifying** | **nad2** |
| **552** | **1.887** | **0** | **0.698** | **15.874** | **0.0001** | **39.202** | **Purifying** | **nad2** |
| **553** | **1.356** | **0.315** | **0.623** | **2.078** | **0.1494** | **34.978** | **Neutral** | **nad2** |
| **554** | **2.199** | **0** | **0.484** | **15.907** | **0.0001** | **27.19** | **Purifying** | **nad2** |
| **555** | **0** | **0** | **0** | **0** | **1** | **0** | **Invariable** | **nad2** |
| **556** | **1.484** | **0** | **0.013** | **12.969** | **0.0003** | **0.721** | **Purifying** | **nad2** |
| **557** | **2.213** | **0** | **0.586** | **16.422** | **0.0001** | **32.929** | **Purifying** | **nad2** |
| **558** | **0** | **0** | **0** | **0** | **1** | **0** | **Invariable** | **nad2** |
| **559** | **5.967** | **0** | **0.461** | **34.585** | **0** | **25.882** | **Purifying** | **nad2** |
| **560** | **1.908** | **0** | **0.444** | **14.772** | **0.0001** | **24.967** | **Purifying** | **nad2** |
| **561** | **0.503** | **0** | **0.008** | **4.548** | **0.033** | **0.436** | **Purifying** | **nad2** |
| **562** | **1.706** | **0** | **0.387** | **17.982** | **0** | **21.727** | **Purifying** | **nad2** |
| **563** | **2.308** | **0** | **0.639** | **17.383** | **0** | **35.899** | **Purifying** | **nad2** |
| **564** | **2.022** | **0** | **0.018** | **15.282** | **0.0001** | **1.004** | **Purifying** | **nad2** |
| **565** | **1.619** | **0** | **0.305** | **24.553** | **0** | **17.124** | **Purifying** | **nad2** |
| **566** | **2.266** | **0** | **0.02** | **20.396** | **0** | **1.11** | **Purifying** | **nad2** |
| **567** | **0** | **0.012** | **0.011** | **0.097** | **0.7554** | **0.607** | **Neutral** | **nad2** |
| **568** | **1.829** | **0** | **0.022** | **14.117** | **0.0002** | **1.216** | **Purifying** | **nad2** |
| **569** | **9.086** | **0** | **0.009** | **10.924** | **0.0009** | **0.484** | **Purifying** | **nad2** |
| **570** | **4.797** | **0** | **0.872** | **19.774** | **0** | **48.998** | **Purifying** | **nad2** |
| **571** | **3.673** | **0** | **0.545** | **22.405** | **0** | **30.644** | **Purifying** | **nad2** |
| **572** | **2.82** | **0** | **0.661** | **18.069** | **0** | **37.122** | **Purifying** | **nad2** |
| **573** | **0** | **0** | **0** | **0** | **1** | **0** | **Invariable** | **nad2** |
| **574** | **4.183** | **0** | **0.013** | **19.412** | **0** | **0.743** | **Purifying** | **nad2** |
| **575** | **2.464** | **0** | **1.055** | **17.743** | **0** | **59.291** | **Purifying** | **nad2** |
| **576** | **1.519** | **0.698** | **0.855** | **0.619** | **0.4314** | **48.023** | **Neutral** | **nad2** |
| **577** | **10000** | **0** | **0.023** | **21.041** | **0** | **1.3** | **Purifying** | **nad2** |
| **578** | **0** | **0** | **0** | **0** | **1** | **0** | **Invariable** | **nad2** |
| **579** | **0.886** | **0** | **0.007** | **7.897** | **0.005** | **0.383** | **Purifying** | **nad2** |
| **580** | **1.621** | **0** | **0.468** | **14.245** | **0.0002** | **26.309** | **Purifying** | **nad2** |
| **581** | **1** | **0** | **0.012** | **14.164** | **0.0002** | **0.651** | **Purifying** | **nad2** |
| **582** | **2.276** | **0** | **0.66** | **15.892** | **0.0001** | **37.11** | **Purifying** | **nad2** |
| **583** | **0.701** | **0** | **0.007** | **4.978** | **0.0257** | **0.401** | **Purifying** | **nad2** |
| **584** | **1.269** | **0** | **0.149** | **16.522** | **0** | **8.373** | **Purifying** | **nad2** |
| **585** | **1.444** | **0** | **0.019** | **13.859** | **0.0002** | **1.06** | **Purifying** | **nad2** |
| **586** | **1.254** | **0** | **0.456** | **12.527** | **0.0004** | **25.599** | **Purifying** | **nad2** |
| **587** | **2.656** | **0** | **0.504** | **24.403** | **0** | **28.296** | **Purifying** | **nad2** |
| **588** | **0** | **0** | **0** | **0** | **1** | **0** | **Invariable** | **nad2** |
| **589** | **0.348** | **0** | **0.009** | **2.412** | **0.1204** | **0.521** | **Neutral** | **nad2** |
| **590** | **3.072** | **0** | **0.579** | **24.286** | **0** | **32.547** | **Purifying** | **nad2** |
| **591** | **0.513** | **0** | **0.006** | **5.014** | **0.0251** | **0.345** | **Purifying** | **nad2** |
| **592** | **1.304** | **0** | **0.026** | **11.606** | **0.0007** | **1.44** | **Purifying** | **nad2** |
| **593** | **1.52** | **0** | **0.487** | **13.094** | **0.0003** | **27.349** | **Purifying** | **nad2** |
| **594** | **1.074** | **0** | **0.005** | **9.077** | **0.0026** | **0.301** | **Purifying** | **nad2** |
| **595** | **2.567** | **0** | **0.896** | **17.468** | **0** | **50.357** | **Purifying** | **nad2** |
| **596** | **7.839** | **0** | **0.438** | **28.911** | **0** | **24.6** | **Purifying** | **nad2** |
| **597** | **2.931** | **0** | **0.018** | **18.284** | **0** | **1.039** | **Purifying** | **nad2** |
| **598** | **3.618** | **0** | **0.031** | **25.097** | **0** | **1.739** | **Purifying** | **nad2** |
| **599** | **0.353** | **0** | **0.009** | **3.494** | **0.0616** | **0.489** | **Neutral** | **nad2** |
| **600** | **0.559** | **0** | **0.008** | **3.319** | **0.0685** | **0.422** | **Neutral** | **nad2** |
| **601** | **1.515** | **0** | **0.49** | **13.485** | **0.0002** | **27.509** | **Purifying** | **nad2** |
| **602** | **0** | **0** | **0** | **0** | **1** | **0** | **Invariable** | **nad2** |
| **603** | **2.123** | **0** | **0.03** | **22.218** | **0** | **1.706** | **Purifying** | **nad2** |
| **604** | **0.616** | **0** | **0.005** | **3.288** | **0.0698** | **0.297** | **Neutral** | **nad2** |
| **605** | **0.715** | **0.383** | **0.453** | **0.197** | **0.6568** | **25.435** | **Neutral** | **nad2** |
| **606** | **0** | **0** | **0** | **0** | **1** | **0** | **Invariable** | **nad2** |
| **607** | **0.315** | **0** | **0.009** | **2.291** | **0.1301** | **0.52** | **Neutral** | **nad2** |
| **608** | **0** | **0** | **0** | **0** | **1** | **0** | **Invariable** | **nad2** |
| **609** | **1.47** | **0.01** | **0.358** | **6.43** | **0.0112** | **20.087** | **Purifying** | **nad2** |
| **610** | **1.517** | **0** | **0.016** | **14.449** | **0.0001** | **0.893** | **Purifying** | **nad2** |
| **611** | **1.881** | **0** | **0.022** | **14.047** | **0.0002** | **1.214** | **Purifying** | **nad2** |
| **612** | **2.598** | **0** | **0.02** | **17.498** | **0** | **1.107** | **Purifying** | **nad2** |
| **613** | **0.886** | **0.009** | **0.02** | **4.883** | **0.0271** | **1.107** | **Purifying** | **nad2** |
| **614** | **0.599** | **0** | **0.009** | **3.454** | **0.0631** | **0.53** | **Neutral** | **nad2** |
| **615** | **1.145** | **0** | **0.022** | **13.317** | **0.0003** | **1.225** | **Purifying** | **nad2** |
| **616** | **4.281** | **0** | **0.612** | **35.077** | **0** | **34.366** | **Purifying** | **nad2** |
| **617** | **0** | **0** | **0** | **0** | **1** | **0** | **Invariable** | **nad2** |
| **618** | **1.913** | **0** | **0.328** | **19.475** | **0** | **18.435** | **Purifying** | **nad2** |
| **619** | **4.954** | **0** | **0.641** | **23.772** | **0** | **36.012** | **Purifying** | **nad2** |
| **620** | **0.636** | **0** | **0.007** | **3.412** | **0.0647** | **0.419** | **Neutral** | **nad2** |
| **621** | **1.555** | **0** | **0.425** | **14.506** | **0.0001** | **23.876** | **Purifying** | **nad2** |
| **622** | **3.029** | **0** | **0.436** | **20.406** | **0** | **24.497** | **Purifying** | **nad2** |
| **623** | **0.713** | **0** | **0.007** | **5.325** | **0.021** | **0.373** | **Purifying** | **nad2** |
| **624** | **1.639** | **0** | **0.018** | **13.933** | **0.0002** | **1.039** | **Purifying** | **nad2** |
| **625** | **0** | **0** | **0** | **0** | **1** | **0** | **Invariable** | **nad2** |
| **626** | **1.552** | **0.017** | **0.649** | **4.426** | **0.0354** | **36.469** | **Purifying** | **nad2** |
| **627** | **0.636** | **0** | **0.007** | **5.184** | **0.0228** | **0.384** | **Purifying** | **nad2** |
| **628** | **2.123** | **0** | **0.477** | **15.749** | **0.0001** | **26.778** | **Purifying** | **nad2** |
| **629** | **3.517** | **0** | **0.444** | **20.224** | **0** | **24.94** | **Purifying** | **nad2** |
| **630** | **4.122** | **0** | **0.958** | **18.904** | **0** | **53.839** | **Purifying** | **nad2** |
| **631** | **0.911** | **0.285** | **0.459** | **0.969** | **0.3248** | **25.779** | **Neutral** | **nad2** |
| **632** | **0.029** | **0** | **0.006** | **2.55** | **0.1103** | **0.348** | **Neutral** | **nad2** |
| **633** | **0** | **0** | **0** | **0** | **1** | **0** | **Invariable** | **nad2** |
| **634** | **1.487** | **0** | **0.018** | **14.373** | **0.0001** | **1.002** | **Purifying** | **nad2** |
| **635** | **0** | **0.059** | **0.023** | **0.991** | **0.3195** | **1.294** | **Neutral** | **nad2** |
| **636** | **0** | **0.008** | **0.006** | **0.454** | **0.5007** | **0.36** | **Neutral** | **nad2** |
| **637** | **1.637** | **0** | **0.015** | **14.376** | **0.0001** | **0.856** | **Purifying** | **nad2** |
| **638** | **1.297** | **0.01** | **0.463** | **5.435** | **0.0197** | **26.001** | **Purifying** | **nad2** |
| **639** | **0** | **0** | **0** | **0** | **1** | **0** | **Invariable** | **nad2** |
| **640** | **2.957** | **0** | **0.773** | **17.678** | **0** | **43.449** | **Purifying** | **nad2** |
| **641** | **6.521** | **0** | **1.238** | **23.528** | **0** | **69.54** | **Purifying** | **nad2** |
| **642** | **0.545** | **0** | **0.008** | **4.65** | **0.031** | **0.436** | **Purifying** | **nad2** |
| **643** | **1.403** | **0** | **0.428** | **13.42** | **0.0002** | **24.072** | **Purifying** | **nad2** |
| **644** | **0.62** | **0.01** | **0.019** | **2.113** | **0.146** | **1.057** | **Neutral** | **nad2** |
| **645** | **1.224** | **0** | **0.021** | **12.627** | **0.0004** | **1.203** | **Purifying** | **nad2** |
| **646** | **0.023** | **0** | **0.005** | **2.57** | **0.1089** | **0.295** | **Neutral** | **nad2** |
| **647** | **0.725** | **0.288** | **0.351** | **0.334** | **0.5633** | **19.723** | **Neutral** | **nad2** |
| **648** | **0** | **0.528** | **0.553** | **0.866** | **0.352** | **31.047** | **Neutral** | **nad2** |
| **649** | **0.715** | **0** | **0.006** | **5.506** | **0.019** | **0.346** | **Purifying** | **nad2** |
| **650** | **1.037** | **0** | **0.025** | **11.191** | **0.0008** | **1.422** | **Purifying** | **nad2** |
| **651** | **0.456** | **0.016** | **0.025** | **1.332** | **0.2484** | **1.431** | **Neutral** | **nad2** |
| **652** | **0** | **0** | **0** | **0** | **1** | **0** | **Invariable** | **nad2** |
| **653** | **1.243** | **0** | **0.288** | **12.574** | **0.0004** | **16.159** | **Purifying** | **nad2** |
| **654** | **0** | **0** | **0** | **0** | **1** | **0** | **Invariable** | **cox1** |
| **655** | **0** | **0** | **0** | **0** | **1** | **0** | **Invariable** | **cox1** |
| **656** | **0** | **0** | **0** | **0** | **1** | **0** | **Invariable** | **cox1** |
| **657** | **0** | **0** | **0** | **0** | **1** | **0** | **Invariable** | **cox1** |
| **658** | **0** | **0** | **0** | **0** | **1** | **0** | **Invariable** | **cox1** |
| **659** | **0** | **0** | **0** | **0** | **1** | **0** | **Invariable** | **cox1** |
| **660** | **0** | **0** | **0** | **0** | **1** | **0** | **Invariable** | **cox1** |
| **661** | **0** | **0** | **0** | **0** | **1** | **0** | **Invariable** | **cox1** |
| **662** | **0.83** | **0** | **0.018** | **12.244** | **0.0005** | **0.984** | **Purifying** | **cox1** |
| **663** | **0** | **0** | **0** | **0** | **1** | **0** | **Invariable** | **cox1** |
| **664** | **0** | **0** | **0** | **0** | **1** | **0** | **Invariable** | **cox1** |
| **665** | **0** | **0** | **0** | **0** | **1** | **0** | **Invariable** | **cox1** |
| **666** | **0.886** | **0** | **0.007** | **7.897** | **0.005** | **0.383** | **Purifying** | **cox1** |
| **667** | **0** | **0** | **0** | **0** | **1** | **0** | **Invariable** | **cox1** |
| **668** | **1.311** | **0** | **0.012** | **17.153** | **0** | **0.673** | **Purifying** | **cox1** |
| **669** | **0** | **0** | **0** | **0** | **1** | **0** | **Invariable** | **cox1** |
| **670** | **0** | **0** | **0** | **0** | **1** | **0** | **Invariable** | **cox1** |
| **671** | **0** | **0** | **0** | **0** | **1** | **0** | **Invariable** | **cox1** |
| **672** | **0.046** | **0** | **0.009** | **2.433** | **0.1188** | **0.489** | **Neutral** | **cox1** |
| **673** | **0** | **0** | **0** | **0** | **1** | **0** | **Invariable** | **cox1** |
| **674** | **0** | **0** | **0** | **0** | **1** | **0** | **Invariable** | **cox1** |
| **675** | **0** | **0** | **0** | **0** | **1** | **0** | **Invariable** | **cox1** |
| **676** | **0** | **0** | **0** | **0** | **1** | **0** | **Invariable** | **cox1** |
| **677** | **0** | **0** | **0** | **0** | **1** | **0** | **Invariable** | **cox1** |
| **678** | **0.998** | **0** | **0.013** | **13.342** | **0.0003** | **0.748** | **Purifying** | **cox1** |
| **679** | **0** | **0.292** | **0.385** | **1.613** | **0.2041** | **21.609** | **Neutral** | **cox1** |
| **680** | **1.304** | **0** | **0.136** | **14.725** | **0.0001** | **7.633** | **Purifying** | **cox1** |
| **681** | **1.38** | **0** | **0.013** | **14.438** | **0.0001** | **0.75** | **Purifying** | **cox1** |
| **682** | **0** | **0** | **0** | **0** | **1** | **0** | **Invariable** | **cox1** |
| **683** | **1.444** | **0** | **0.282** | **18.554** | **0** | **15.871** | **Purifying** | **cox1** |
| **684** | **0.015** | **0** | **0.006** | **1.749** | **0.186** | **0.325** | **Neutral** | **cox1** |
| **685** | **0** | **0** | **0** | **0** | **1** | **0** | **Invariable** | **cox1** |
| **686** | **0** | **0** | **0** | **0** | **1** | **0** | **Invariable** | **cox1** |
| **687** | **0.705** | **0** | **0.191** | **10.283** | **0.0013** | **10.703** | **Purifying** | **cox1** |
| **688** | **0** | **0** | **0** | **0** | **1** | **0** | **Invariable** | **cox1** |
| **689** | **0** | **0** | **0** | **0** | **1** | **0** | **Invariable** | **cox1** |
| **690** | **0** | **0** | **0** | **0** | **1** | **0** | **Invariable** | **cox1** |
| **691** | **0** | **0** | **0** | **0** | **1** | **0** | **Invariable** | **cox1** |
| **692** | **0.837** | **0** | **0.025** | **10.256** | **0.0014** | **1.393** | **Purifying** | **cox1** |
| **693** | **0** | **0** | **0** | **0** | **1** | **0** | **Invariable** | **cox1** |
| **694** | **0** | **0** | **0** | **0** | **1** | **0** | **Invariable** | **cox1** |
| **695** | **0** | **0** | **0** | **0** | **1** | **0** | **Invariable** | **cox1** |
| **696** | **0** | **0** | **0** | **0** | **1** | **0** | **Invariable** | **cox1** |
| **697** | **0.422** | **0** | **0.006** | **4.778** | **0.0288** | **0.344** | **Purifying** | **cox1** |
| **698** | **0.254** | **0** | **0.009** | **3.347** | **0.0673** | **0.488** | **Neutral** | **cox1** |
| **699** | **0.022** | **0** | **0.007** | **2.005** | **0.1567** | **0.392** | **Neutral** | **cox1** |
| **700** | **0.039** | **0** | **0.005** | **3.518** | **0.0607** | **0.304** | **Neutral** | **cox1** |
| **701** | **2.108** | **0** | **0.03** | **22.824** | **0** | **1.706** | **Purifying** | **cox1** |
| **702** | **0** | **0** | **0** | **0** | **1** | **0** | **Invariable** | **cox1** |
| **703** | **0.513** | **0** | **0.006** | **2.172** | **0.1405** | **0.347** | **Neutral** | **cox1** |
| **704** | **0** | **0** | **0** | **0** | **1** | **0** | **Invariable** | **cox1** |
| **705** | **0** | **0** | **0** | **0** | **1** | **0** | **Invariable** | **cox1** |
| **706** | **1.304** | **0** | **0.02** | **15.663** | **0.0001** | **1.096** | **Purifying** | **cox1** |
| **707** | **1.564** | **0** | **0.02** | **15.668** | **0.0001** | **1.119** | **Purifying** | **cox1** |
| **708** | **0** | **0** | **0** | **0** | **1** | **0** | **Invariable** | **cox1** |
| **709** | **0** | **0** | **0** | **0** | **1** | **0** | **Invariable** | **cox1** |
| **710** | **1.362** | **0** | **0.018** | **13.725** | **0.0002** | **0.995** | **Purifying** | **cox1** |
| **711** | **0** | **0** | **0** | **0** | **1** | **0** | **Invariable** | **cox1** |
| **712** | **0** | **0** | **0** | **0** | **1** | **0** | **Invariable** | **cox1** |
| **713** | **0** | **0** | **0** | **0** | **1** | **0** | **Invariable** | **cox1** |
| **714** | **0** | **0** | **0** | **0** | **1** | **0** | **Invariable** | **cox1** |
| **715** | **0** | **0** | **0** | **0** | **1** | **0** | **Invariable** | **cox1** |
| **716** | **0** | **0.01** | **0.008** | **0.296** | **0.5867** | **0.445** | **Neutral** | **cox1** |
| **717** | **0** | **0** | **0** | **0** | **1** | **0** | **Invariable** | **cox1** |
| **718** | **0** | **0** | **0** | **0** | **1** | **0** | **Invariable** | **cox1** |
| **719** | **0** | **0** | **0** | **0** | **1** | **0** | **Invariable** | **cox1** |
| **720** | **0** | **0** | **0** | **0** | **1** | **0** | **Invariable** | **cox1** |
| **721** | **0** | **0** | **0** | **0** | **1** | **0** | **Invariable** | **cox1** |
| **722** | **0** | **0** | **0** | **0** | **1** | **0** | **Invariable** | **cox1** |
| **723** | **0** | **0** | **0** | **0** | **1** | **0** | **Invariable** | **cox1** |
| **724** | **0.87** | **0** | **0.015** | **10.784** | **0.001** | **0.866** | **Purifying** | **cox1** |
| **725** | **0** | **0** | **0** | **0** | **1** | **0** | **Invariable** | **cox1** |
| **726** | **0.454** | **0** | **0.008** | **4.923** | **0.0265** | **0.422** | **Purifying** | **cox1** |
| **727** | **0** | **0** | **0** | **0** | **1** | **0** | **Invariable** | **cox1** |
| **728** | **0.01** | **0** | **0.004** | **1.981** | **0.1593** | **0.21** | **Neutral** | **cox1** |
| **729** | **2.077** | **0** | **0.018** | **17.234** | **0** | **1.038** | **Purifying** | **cox1** |
| **730** | **0** | **0** | **0** | **0** | **1** | **0** | **Invariable** | **cox1** |
| **731** | **0** | **0** | **0** | **0** | **1** | **0** | **Invariable** | **cox1** |
| **732** | **0.665** | **0** | **0.028** | **8.861** | **0.0029** | **1.583** | **Purifying** | **cox1** |
| **733** | **0.655** | **0** | **0.008** | **5.431** | **0.0198** | **0.424** | **Purifying** | **cox1** |
| **734** | **0.537** | **0** | **0.008** | **4.634** | **0.0313** | **0.437** | **Purifying** | **cox1** |
| **735** | **0** | **0** | **0** | **0** | **1** | **0** | **Invariable** | **cox1** |
| **736** | **0** | **0** | **0** | **0** | **1** | **0** | **Invariable** | **cox1** |
| **737** | **2.251** | **0** | **0.39** | **18.92** | **0** | **21.898** | **Purifying** | **cox1** |
| **738** | **0.681** | **0** | **0.004** | **3.559** | **0.0592** | **0.239** | **Neutral** | **cox1** |
| **739** | **1.224** | **0** | **0.145** | **16.405** | **0.0001** | **8.13** | **Purifying** | **cox1** |
| **740** | **0.961** | **0** | **0.02** | **12.169** | **0.0005** | **1.114** | **Purifying** | **cox1** |
| **741** | **0.987** | **0** | **0.007** | **8.022** | **0.0046** | **0.384** | **Purifying** | **cox1** |
| **742** | **0** | **0** | **0** | **0** | **1** | **0** | **Invariable** | **cox1** |
| **743** | **0** | **0** | **0** | **0** | **1** | **0** | **Invariable** | **cox1** |
| **744** | **0** | **0** | **0** | **0** | **1** | **0** | **Invariable** | **cox1** |
| **745** | **0.732** | **0** | **0.008** | **4.804** | **0.0284** | **0.46** | **Purifying** | **cox1** |
| **746** | **0** | **0** | **0** | **0** | **1** | **0** | **Invariable** | **cox1** |
| **747** | **0** | **0.007** | **0.007** | **0.17** | **0.6803** | **0.374** | **Neutral** | **cox1** |
| **748** | **1.105** | **0** | **0.008** | **8** | **0.0047** | **0.431** | **Purifying** | **cox1** |
| **749** | **0** | **0** | **0** | **0** | **1** | **0** | **Invariable** | **cox1** |
| **750** | **0** | **0** | **0** | **0** | **1** | **0** | **Invariable** | **cox1** |
| **751** | **0** | **0** | **0** | **0** | **1** | **0** | **Invariable** | **cox1** |
| **752** | **0** | **0** | **0** | **0** | **1** | **0** | **Invariable** | **cox1** |
| **753** | **2.58** | **0** | **0.66** | **18.093** | **0** | **37.093** | **Purifying** | **cox1** |
| **754** | **1.546** | **0** | **0.321** | **13.742** | **0.0002** | **18.025** | **Purifying** | **cox1** |
| **755** | **3.454** | **0** | **0.749** | **23.961** | **0** | **42.079** | **Purifying** | **cox1** |
| **756** | **2.537** | **0** | **0.418** | **19.151** | **0** | **23.51** | **Purifying** | **cox1** |
| **757** | **1.105** | **0** | **0.013** | **13.087** | **0.0003** | **0.735** | **Purifying** | **cox1** |
| **758** | **0** | **0** | **0** | **0** | **1** | **0** | **Invariable** | **cox1** |
| **759** | **2.932** | **0** | **0.926** | **17.859** | **0** | **52.046** | **Purifying** | **cox1** |
| **760** | **0** | **0** | **0** | **0** | **1** | **0** | **Invariable** | **cox1** |
| **761** | **2.428** | **0** | **0.64** | **17.757** | **0** | **35.976** | **Purifying** | **cox1** |
| **762** | **1.572** | **0** | **0.689** | **14.534** | **0.0001** | **38.719** | **Purifying** | **cox1** |
| **763** | **0** | **0** | **0** | **0** | **1** | **0** | **Invariable** | **cox1** |
| **764** | **0.52** | **0** | **0.007** | **4.872** | **0.0273** | **0.371** | **Purifying** | **cox1** |
| **765** | **0.393** | **0** | **0.007** | **4.493** | **0.034** | **0.37** | **Purifying** | **cox1** |
| **766** | **0.699** | **0** | **0.006** | **5.414** | **0.02** | **0.325** | **Purifying** | **cox1** |
| **767** | **0** | **0** | **0** | **0** | **1** | **0** | **Invariable** | **cox1** |
| **768** | **0** | **0** | **0** | **0** | **1** | **0** | **Invariable** | **cox1** |
| **769** | **2.356** | **0** | **0.324** | **20.772** | **0** | **18.223** | **Purifying** | **cox1** |
| **770** | **0.01** | **0** | **0.004** | **1.977** | **0.1597** | **0.209** | **Neutral** | **cox1** |
| **771** | **1.564** | **0** | **0.307** | **19.324** | **0** | **17.265** | **Purifying** | **cox1** |
| **772** | **2.232** | **0** | **0.297** | **25.593** | **0** | **16.691** | **Purifying** | **cox1** |
| **773** | **0.422** | **0** | **0.007** | **4.581** | **0.0323** | **0.382** | **Purifying** | **cox1** |
| **774** | **0.875** | **0** | **0.013** | **15.339** | **0.0001** | **0.752** | **Purifying** | **cox1** |
| **775** | **1.621** | **0** | **0.019** | **14.16** | **0.0002** | **1.06** | **Purifying** | **cox1** |
| **776** | **1.886** | **0** | **0.451** | **19.377** | **0** | **25.313** | **Purifying** | **cox1** |
| **777** | **10000** | **0** | **0.65** | **34.243** | **0** | **36.501** | **Purifying** | **cox1** |
| **778** | **1.054** | **0** | **0.137** | **14.697** | **0.0001** | **7.71** | **Purifying** | **cox1** |
| **779** | **1.587** | **0** | **0.378** | **17.456** | **0** | **21.246** | **Purifying** | **cox1** |
| **780** | **0.046** | **0** | **0.009** | **2.427** | **0.1193** | **0.49** | **Neutral** | **cox1** |
| **781** | **2.373** | **0** | **0.311** | **20.235** | **0** | **17.458** | **Purifying** | **cox1** |
| **782** | **1.417** | **0** | **0.262** | **23.476** | **0** | **14.701** | **Purifying** | **cox1** |
| **783** | **4.12** | **0** | **0.327** | **25.387** | **0** | **18.345** | **Purifying** | **cox1** |
| **784** | **1.247** | **0** | **0.456** | **12.554** | **0.0004** | **25.595** | **Purifying** | **cox1** |
| **785** | **0.715** | **0** | **0.006** | **5.236** | **0.0221** | **0.36** | **Purifying** | **cox1** |
| **786** | **0.987** | **0** | **0.009** | **7.507** | **0.0061** | **0.496** | **Purifying** | **cox1** |
| **787** | **0.834** | **0** | **0.012** | **10.989** | **0.0009** | **0.695** | **Purifying** | **cox1** |
| **788** | **1.212** | **0** | **0.021** | **20.814** | **0** | **1.189** | **Purifying** | **cox1** |
| **789** | **0** | **0** | **0** | **0** | **1** | **0** | **Invariable** | **cox1** |
| **790** | **1.136** | **0** | **0.008** | **5.534** | **0.0187** | **0.424** | **Purifying** | **cox1** |
| **791** | **2.153** | **0** | **0.331** | **17.683** | **0** | **18.619** | **Purifying** | **cox1** |
| **792** | **1.105** | **0** | **0.007** | **8.219** | **0.0041** | **0.384** | **Purifying** | **cox1** |
| **793** | **0.892** | **0** | **0.028** | **9.626** | **0.0019** | **1.599** | **Purifying** | **cox1** |
| **794** | **0.393** | **0** | **0.007** | **4.472** | **0.0345** | **0.382** | **Purifying** | **cox1** |
| **795** | **0.454** | **0** | **0.008** | **4.923** | **0.0265** | **0.422** | **Purifying** | **cox1** |
| **796** | **1.01** | **0** | **0.007** | **7.927** | **0.0049** | **0.407** | **Purifying** | **cox1** |
| **797** | **1.224** | **0** | **0.016** | **13.816** | **0.0002** | **0.89** | **Purifying** | **cox1** |
| **798** | **3.118** | **0** | **0.919** | **18.68** | **0** | **51.619** | **Purifying** | **cox1** |
| **799** | **0.477** | **0** | **0.007** | **4.418** | **0.0356** | **0.4** | **Purifying** | **cox1** |
| **800** | **0.046** | **0** | **0.009** | **2.432** | **0.1189** | **0.489** | **Neutral** | **cox1** |
| **801** | **2.439** | **0** | **0.444** | **22.603** | **0** | **24.943** | **Purifying** | **cox1** |
| **802** | **0.961** | **0** | **0.008** | **10.336** | **0.0013** | **0.46** | **Purifying** | **cox1** |
| **803** | **0** | **0** | **0** | **0** | **1** | **0** | **Invariable** | **cox1** |
| **804** | **0.56** | **0** | **0.007** | **4.976** | **0.0257** | **0.372** | **Purifying** | **cox1** |
| **805** | **0** | **0** | **0** | **0** | **1** | **0** | **Invariable** | **cox1** |
| **806** | **0.029** | **0** | **0.006** | **2.549** | **0.1104** | **0.348** | **Neutral** | **cox1** |
| **807** | **3.122** | **0** | **0.829** | **19.158** | **0** | **46.577** | **Purifying** | **cox1** |
| **808** | **0.382** | **0** | **0.011** | **8.157** | **0.0043** | **0.622** | **Purifying** | **cox1** |
| **809** | **2.905** | **0** | **0.492** | **24.341** | **0** | **27.651** | **Purifying** | **cox1** |
| **810** | **0.967** | **0** | **0.022** | **12.648** | **0.0004** | **1.211** | **Purifying** | **cox1** |
| **811** | **9.81** | **0** | **0.358** | **28.46** | **0** | **20.104** | **Purifying** | **cox1** |
| **812** | **3.26** | **0** | **0.387** | **23.855** | **0** | **21.746** | **Purifying** | **cox1** |
| **813** | **2.008** | **0** | **0.35** | **17.99** | **0** | **19.67** | **Purifying** | **cox1** |
| **814** | **0** | **0** | **0** | **0** | **1** | **0** | **Invariable** | **cox1** |
| **815** | **2.496** | **0** | **0.498** | **22.31** | **0** | **27.984** | **Purifying** | **cox1** |
| **816** | **0.029** | **0** | **0.006** | **2.548** | **0.1105** | **0.348** | **Neutral** | **cox1** |
| **817** | **0.805** | **0** | **0.015** | **10.523** | **0.0012** | **0.864** | **Purifying** | **cox1** |
| **818** | **1.019** | **0** | **0.008** | **7.874** | **0.005** | **0.43** | **Purifying** | **cox1** |
| **819** | **0** | **0** | **0** | **0** | **1** | **0** | **Invariable** | **cox1** |
| **820** | **1.684** | **0** | **0.009** | **9.337** | **0.0022** | **0.483** | **Purifying** | **cox1** |
| **821** | **0.023** | **0** | **0.006** | **2.163** | **0.1413** | **0.359** | **Neutral** | **cox1** |
| **822** | **0.523** | **0** | **0.008** | **4.582** | **0.0323** | **0.436** | **Purifying** | **cox1** |
| **823** | **0** | **0** | **0** | **0** | **1** | **0** | **Invariable** | **cox1** |
| **824** | **1.492** | **0** | **0.338** | **17.079** | **0** | **19.018** | **Purifying** | **cox1** |
| **825** | **0** | **0** | **0** | **0** | **1** | **0** | **Invariable** | **cox1** |
| **826** | **2.453** | **0** | **0.536** | **24.132** | **0** | **30.136** | **Purifying** | **cox1** |
| **827** | **1.398** | **0** | **0.008** | **9.349** | **0.0022** | **0.47** | **Purifying** | **cox1** |
| **828** | **1.221** | **0** | **0.012** | **9.666** | **0.0019** | **0.675** | **Purifying** | **cox1** |
| **829** | **3.854** | **0** | **0.527** | **24.466** | **0** | **29.591** | **Purifying** | **cox1** |
| **830** | **2.373** | **0** | **0.359** | **18.346** | **0** | **20.152** | **Purifying** | **cox1** |
| **831** | **0.405** | **0** | **0.121** | **9** | **0.0027** | **6.776** | **Purifying** | **cox1** |
| **832** | **2.077** | **0** | **0.168** | **19.326** | **0** | **9.433** | **Purifying** | **cox1** |
| **833** | **0.404** | **0** | **0.011** | **6.294** | **0.0121** | **0.607** | **Purifying** | **cox1** |
| **834** | **0.536** | **0** | **0.006** | **5.088** | **0.0241** | **0.345** | **Purifying** | **cox1** |
| **835** | **0.513** | **0** | **0.007** | **4.904** | **0.0268** | **0.376** | **Purifying** | **cox1** |
| **836** | **0.301** | **0** | **0.009** | **2.233** | **0.1351** | **0.52** | **Neutral** | **cox1** |
| **837** | **0** | **0** | **0** | **0** | **1** | **0** | **Invariable** | **cox1** |
| **838** | **0** | **0** | **0** | **0** | **1** | **0** | **Invariable** | **cox1** |
| **839** | **0** | **0** | **0** | **0** | **1** | **0** | **Invariable** | **cox1** |
| **840** | **0** | **0** | **0** | **0** | **1** | **0** | **Invariable** | **cox1** |
| **841** | **0.71** | **0** | **0.017** | **8.404** | **0.0037** | **0.971** | **Purifying** | **cox1** |
| **842** | **1.505** | **0** | **0.45** | **14.02** | **0.0002** | **25.29** | **Purifying** | **cox1** |
| **843** | **0.735** | **0** | **0.023** | **9.453** | **0.0021** | **1.267** | **Purifying** | **cox1** |
| **844** | **1.165** | **0** | **0.383** | **13.435** | **0.0002** | **21.517** | **Purifying** | **cox1** |
| **845** | **0.545** | **0** | **0.006** | **3.032** | **0.0816** | **0.318** | **Neutral** | **cox1** |
| **846** | **1.735** | **0** | **0.753** | **14.91** | **0.0001** | **42.296** | **Purifying** | **cox1** |
| **847** | **1.317** | **0** | **0.358** | **16.852** | **0** | **20.141** | **Purifying** | **cox1** |
| **848** | **1.362** | **0** | **0.318** | **16.71** | **0** | **17.85** | **Purifying** | **cox1** |
| **849** | **0.526** | **0** | **0.292** | **10.258** | **0.0014** | **16.385** | **Purifying** | **cox1** |
| **850** | **1.174** | **0** | **0.274** | **17.923** | **0** | **15.378** | **Purifying** | **cox1** |
| **851** | **0.326** | **0** | **0.006** | **4.445** | **0.035** | **0.343** | **Purifying** | **cox1** |
| **852** | **2.083** | **0** | **0.28** | **24.704** | **0** | **15.745** | **Purifying** | **cox1** |
| **853** | **0** | **0** | **0** | **0** | **1** | **0** | **Invariable** | **cox1** |
| **854** | **0** | **0** | **0** | **0** | **1** | **0** | **Invariable** | **cox1** |
| **855** | **0** | **0** | **0** | **0** | **1** | **0** | **Invariable** | **cox1** |
| **856** | **0.023** | **0** | **0.007** | **2.026** | **0.1546** | **0.394** | **Neutral** | **cox1** |
| **857** | **2.179** | **0** | **0.661** | **16.211** | **0.0001** | **37.158** | **Purifying** | **cox1** |
| **858** | **0** | **0** | **0** | **0** | **1** | **0** | **Invariable** | **cox1** |
| **859** | **1.941** | **0** | **0.018** | **17.31** | **0** | **0.994** | **Purifying** | **cox1** |
| **860** | **0.487** | **0** | **0.008** | **3.142** | **0.0763** | **0.422** | **Neutral** | **cox1** |
| **861** | **1.78** | **0** | **0.015** | **15.944** | **0.0001** | **0.852** | **Purifying** | **cox1** |
| **862** | **2.308** | **0** | **0.723** | **15.962** | **0.0001** | **40.642** | **Purifying** | **cox1** |
| **863** | **0.701** | **0** | **0.008** | **7.309** | **0.0069** | **0.429** | **Purifying** | **cox1** |
| **864** | **3.521** | **0** | **0.506** | **23.805** | **0** | **28.447** | **Purifying** | **cox1** |
| **865** | **3.949** | **0** | **0.365** | **22.518** | **0** | **20.518** | **Purifying** | **cox1** |
| **866** | **0** | **0** | **0** | **0** | **1** | **0** | **Invariable** | **cox1** |
| **867** | **4.9** | **0** | **0.474** | **26.193** | **0** | **26.633** | **Purifying** | **cox1** |
| **868** | **0.155** | **0** | **0.007** | **5.002** | **0.0253** | **0.376** | **Purifying** | **cox1** |
| **869** | **3.122** | **0** | **0.676** | **23.017** | **0** | **37.969** | **Purifying** | **cox1** |
| **870** | **0** | **0** | **0** | **0** | **1** | **0** | **Invariable** | **cox1** |
| **871** | **2.229** | **0** | **0.357** | **25.746** | **0** | **20.073** | **Purifying** | **cox1** |
| **872** | **1.454** | **0** | **0.009** | **13.203** | **0.0003** | **0.488** | **Purifying** | **cox1** |
| **873** | **1.236** | **0** | **0.018** | **20.094** | **0** | **0.989** | **Purifying** | **cox1** |
| **874** | **2.99** | **0** | **0.018** | **20.971** | **0** | **1.038** | **Purifying** | **cox1** |
| **875** | **1.69** | **0** | **0.022** | **14.196** | **0.0002** | **1.218** | **Purifying** | **cox1** |
| **876** | **0.455** | **0** | **0.006** | **2.922** | **0.0874** | **0.35** | **Neutral** | **cox1** |
| **877** | **2.25** | **0** | **0.683** | **16.642** | **0** | **38.374** | **Purifying** | **cox1** |
| **878** | **0.883** | **0** | **0.008** | **7.542** | **0.006** | **0.463** | **Purifying** | **cox1** |
| **879** | **8.832** | **0** | **0.012** | **9.742** | **0.0018** | **0.664** | **Purifying** | **cox1** |
| **880** | **0** | **0** | **0** | **0** | **1** | **0** | **Invariable** | **cox1** |
| **881** | **1.752** | **0** | **0.71** | **14.493** | **0.0001** | **39.867** | **Purifying** | **cox1** |
| **882** | **0.883** | **0** | **0.007** | **7.811** | **0.0052** | **0.407** | **Purifying** | **cox1** |
| **883** | **0** | **0** | **0** | **0** | **1** | **0** | **Invariable** | **cox1** |
| **884** | **0** | **0** | **0** | **0** | **1** | **0** | **Invariable** | **cox1** |
| **885** | **0.523** | **0** | **0.005** | **5.385** | **0.0203** | **0.286** | **Purifying** | **cox1** |
| **886** | **3.806** | **0** | **0.022** | **17.378** | **0** | **1.249** | **Purifying** | **cox1** |
| **887** | **1.666** | **0** | **0.345** | **17.267** | **0** | **19.39** | **Purifying** | **cox1** |
| **888** | **0.159** | **0** | **0.008** | **5.099** | **0.0239** | **0.425** | **Purifying** | **cox1** |
| **889** | **1.423** | **0** | **0.324** | **16.908** | **0** | **18.208** | **Purifying** | **cox1** |
| **890** | **1.054** | **0** | **0.008** | **7.63** | **0.0057** | **0.462** | **Purifying** | **cox1** |
| **891** | **0** | **0** | **0** | **0** | **1** | **0** | **Invariable** | **cox1** |
| **892** | **0** | **0** | **0** | **0** | **1** | **0** | **Invariable** | **cox1** |
| **893** | **0** | **0** | **0** | **0** | **1** | **0** | **Invariable** | **cox1** |
| **894** | **1.6** | **0** | **0.68** | **14.686** | **0.0001** | **38.2** | **Purifying** | **cox1** |
| **895** | **4.02** | **0** | **0.649** | **23.866** | **0** | **36.457** | **Purifying** | **cox1** |
| **896** | **1.536** | **0** | **0.009** | **14.094** | **0.0002** | **0.525** | **Purifying** | **cox1** |
| **897** | **0** | **0** | **0** | **0** | **1** | **0** | **Invariable** | **cox1** |
| **898** | **1.557** | **0** | **0.023** | **18.034** | **0** | **1.28** | **Purifying** | **cox1** |
| **899** | **0** | **0.012** | **0.011** | **0.094** | **0.7595** | **0.603** | **Neutral** | **cox1** |
| **900** | **2.276** | **0** | **0.389** | **18.891** | **0** | **21.856** | **Purifying** | **cox1** |
| **901** | **0.635** | **0** | **0.007** | **5.204** | **0.0225** | **0.372** | **Purifying** | **cox1** |
| **902** | **1.01** | **0** | **0.009** | **7.539** | **0.006** | **0.495** | **Purifying** | **cox1** |
| **903** | **0.93** | **0** | **0.026** | **13.303** | **0.0003** | **1.481** | **Purifying** | **cox1** |
| **904** | **0** | **0** | **0** | **0** | **1** | **0** | **Invariable** | **cox1** |
| **905** | **1.788** | **0** | **0.311** | **19.695** | **0** | **17.491** | **Purifying** | **cox1** |
| **906** | **1.105** | **0** | **0.008** | **7.828** | **0.0051** | **0.463** | **Purifying** | **cox1** |
| **907** | **2.189** | **0** | **0.022** | **18.047** | **0** | **1.247** | **Purifying** | **cox1** |
| **908** | **0.578** | **0** | **0.008** | **4.563** | **0.0327** | **0.422** | **Purifying** | **cox1** |
| **909** | **0.708** | **0** | **0.011** | **12.349** | **0.0004** | **0.629** | **Purifying** | **cox1** |
| **910** | **0** | **0** | **0** | **0** | **1** | **0** | **Invariable** | **cox1** |
| **911** | **0** | **0** | **0** | **0** | **1** | **0** | **Invariable** | **cox1** |
| **912** | **3.473** | **0** | **0.008** | **10.222** | **0.0014** | **0.46** | **Purifying** | **cox1** |
| **913** | **0** | **0** | **0** | **0** | **1** | **0** | **Invariable** | **cox1** |
| **914** | **0** | **0** | **0** | **0** | **1** | **0** | **Invariable** | **cox1** |
| **915** | **5.388** | **0** | **0.557** | **35.196** | **0** | **31.283** | **Purifying** | **cox1** |
| **916** | **3.237** | **0** | **0.03** | **23.487** | **0** | **1.709** | **Purifying** | **cox1** |
| **917** | **0.028** | **0.005** | **0.005** | **-0.022** | **1** | **0.269** | **Neutral** | **cox1** |
| **918** | **3.601** | **0** | **0.388** | **29.49** | **0** | **21.785** | **Purifying** | **cox1** |
| **919** | **0** | **0** | **0** | **0** | **1** | **0** | **Invariable** | **cox1** |
| **920** | **2.164** | **0** | **0.346** | **18.321** | **0** | **19.449** | **Purifying** | **cox1** |
| **921** | **0.395** | **0** | **0.006** | **4.67** | **0.0307** | **0.344** | **Purifying** | **cox1** |
| **922** | **0** | **0** | **0** | **0** | **1** | **0** | **Invariable** | **cox1** |
| **923** | **0** | **0.01** | **0.008** | **0.295** | **0.5868** | **0.446** | **Neutral** | **cox1** |
| **924** | **0** | **0** | **0** | **0** | **1** | **0** | **Invariable** | **cox1** |
| **925** | **1.458** | **0** | **0.337** | **17.005** | **0** | **18.909** | **Purifying** | **cox1** |
| **926** | **0** | **0** | **0** | **0** | **1** | **0** | **Invariable** | **cox1** |
| **927** | **0.022** | **0** | **0.007** | **2.009** | **0.1564** | **0.393** | **Neutral** | **cox1** |
| **928** | **1.459** | **0** | **0.515** | **13.946** | **0.0002** | **28.908** | **Purifying** | **cox1** |
| **929** | **0.535** | **0** | **0.005** | **5.39** | **0.0203** | **0.286** | **Purifying** | **cox1** |
| **930** | **2.169** | **0** | **0.336** | **21.682** | **0** | **18.868** | **Purifying** | **cox1** |
| **931** | **0** | **0** | **0** | **0** | **1** | **0** | **Invariable** | **cox1** |
| **932** | **2.475** | **0** | **0.471** | **20.535** | **0** | **26.435** | **Purifying** | **cox1** |
| **933** | **2.637** | **0** | **0.318** | **21.643** | **0** | **17.879** | **Purifying** | **cox1** |
| **934** | **1.115** | **0** | **0.017** | **13.045** | **0.0003** | **0.935** | **Purifying** | **cox1** |
| **935** | **0** | **0** | **0** | **0** | **1** | **0** | **Invariable** | **cox1** |
| **936** | **0** | **0** | **0** | **0** | **1** | **0** | **Invariable** | **cox1** |
| **937** | **0.489** | **0** | **0.015** | **10.274** | **0.0013** | **0.819** | **Purifying** | **cox1** |
| **938** | **0** | **0** | **0** | **0** | **1** | **0** | **Invariable** | **cox1** |
| **939** | **0** | **0** | **0** | **0** | **1** | **0** | **Invariable** | **cox1** |
| **940** | **2.019** | **0** | **0.027** | **23.112** | **0** | **1.504** | **Purifying** | **cox1** |
| **941** | **0** | **0** | **0** | **0** | **1** | **0** | **Invariable** | **cox1** |
| **942** | **1.01** | **0** | **0.007** | **8.054** | **0.0045** | **0.384** | **Purifying** | **cox1** |
| **943** | **0** | **0** | **0** | **0** | **1** | **0** | **Invariable** | **cox1** |
| **944** | **0** | **0** | **0** | **0** | **1** | **0** | **Invariable** | **cox1** |
| **945** | **1.814** | **0** | **0.338** | **19.198** | **0** | **18.963** | **Purifying** | **cox1** |
| **946** | **0.937** | **0** | **0.017** | **10.98** | **0.0009** | **0.941** | **Purifying** | **cox1** |
| **947** | **0.536** | **0** | **0.006** | **5.101** | **0.0239** | **0.345** | **Purifying** | **cox1** |
| **948** | **0** | **0** | **0** | **0** | **1** | **0** | **Invariable** | **cox1** |
| **949** | **0.987** | **0** | **0.007** | **7.894** | **0.005** | **0.407** | **Purifying** | **cox1** |
| **950** | **0.742** | **0** | **0.007** | **5.104** | **0.0239** | **0.402** | **Purifying** | **cox1** |
| **951** | **0.987** | **0** | **0.016** | **12.997** | **0.0003** | **0.886** | **Purifying** | **cox1** |
| **952** | **0** | **0** | **0** | **0** | **1** | **0** | **Invariable** | **cox1** |
| **953** | **0** | **0** | **0** | **0** | **1** | **0** | **Invariable** | **cox1** |
| **954** | **0** | **0.015** | **0.012** | **0.169** | **0.6813** | **0.681** | **Neutral** | **cox1** |
| **955** | **0.621** | **0** | **0.008** | **5.35** | **0.0207** | **0.423** | **Purifying** | **cox1** |
| **956** | **0.841** | **0** | **0.02** | **11.78** | **0.0006** | **1.114** | **Purifying** | **cox1** |
| **957** | **0.513** | **0** | **0.006** | **5.014** | **0.0251** | **0.345** | **Purifying** | **cox1** |
| **958** | **0.621** | **0** | **0.006** | **3.517** | **0.0607** | **0.352** | **Neutral** | **cox1** |
| **959** | **1.666** | **0** | **0.021** | **13.378** | **0.0003** | **1.207** | **Purifying** | **cox1** |
| **960** | **1.864** | **0** | **0.019** | **14.61** | **0.0001** | **1.063** | **Purifying** | **cox1** |
| **961** | **0.01** | **0** | **0.004** | **1.976** | **0.1598** | **0.209** | **Neutral** | **cox1** |
| **962** | **1.793** | **0** | **0.021** | **14.358** | **0.0002** | **1.188** | **Purifying** | **cox1** |
| **963** | **0** | **0** | **0** | **0** | **1** | **0** | **Invariable** | **cox1** |
| **964** | **0.561** | **0** | **0.007** | **5.012** | **0.0252** | **0.377** | **Purifying** | **cox1** |
| **965** | **0** | **0** | **0** | **0** | **1** | **0** | **Invariable** | **cox1** |
| **966** | **0** | **0** | **0** | **0** | **1** | **0** | **Invariable** | **cox1** |
| **967** | **1.171** | **0** | **0.45** | **12.399** | **0.0004** | **25.272** | **Purifying** | **cox1** |
| **968** | **2.397** | **0** | **0.312** | **20.612** | **0** | **17.53** | **Purifying** | **cox1** |
| **969** | **0.547** | **0** | **0.007** | **4.918** | **0.0266** | **0.383** | **Purifying** | **cox1** |
| **970** | **1.388** | **0** | **0.297** | **13.324** | **0.0003** | **16.69** | **Purifying** | **cox1** |
| **971** | **2.123** | **0** | **0.36** | **22.124** | **0** | **20.245** | **Purifying** | **cox1** |
| **972** | **4.895** | **0** | **0.517** | **33.714** | **0** | **29.028** | **Purifying** | **cox1** |
| **973** | **0.01** | **0** | **0.004** | **1.974** | **0.16** | **0.209** | **Neutral** | **cox1** |
| **974** | **0** | **0** | **0** | **0** | **1** | **0** | **Invariable** | **cox1** |
| **975** | **0.029** | **0** | **0.006** | **2.552** | **0.1101** | **0.348** | **Neutral** | **cox1** |
| **976** | **0** | **0** | **0** | **0** | **1** | **0** | **Invariable** | **cox1** |
| **977** | **0** | **0** | **0** | **0** | **1** | **0** | **Invariable** | **cox1** |
| **978** | **1.564** | **0** | **0.018** | **15.643** | **0.0001** | **1.025** | **Purifying** | **cox1** |
| **979** | **0.718** | **0** | **0.019** | **10.745** | **0.001** | **1.04** | **Purifying** | **cox1** |
| **980** | **1.192** | **0** | **0.018** | **10.351** | **0.0013** | **0.986** | **Purifying** | **cox1** |
| **981** | **3.594** | **0** | **0.85** | **20.365** | **0** | **47.772** | **Purifying** | **cox1** |
| **982** | **0** | **0** | **0** | **0** | **1** | **0** | **Invariable** | **cox1** |
| **983** | **1.572** | **0** | **0.653** | **14.131** | **0.0002** | **36.709** | **Purifying** | **cox1** |
| **984** | **0** | **0** | **0** | **0** | **1** | **0** | **Invariable** | **cox1** |
| **985** | **1.803** | **0** | **0.014** | **16.12** | **0.0001** | **0.807** | **Purifying** | **cox1** |
| **986** | **0.827** | **0** | **0.152** | **13.695** | **0.0002** | **8.544** | **Purifying** | **cox1** |
| **987** | **0** | **0** | **0** | **0** | **1** | **0** | **Invariable** | **cox1** |
| **988** | **1.264** | **0** | **0.469** | **12.668** | **0.0004** | **26.376** | **Purifying** | **cox1** |
| **989** | **1.123** | **0** | **0.014** | **13.633** | **0.0002** | **0.795** | **Purifying** | **cox1** |
| **990** | **0** | **0** | **0** | **0** | **1** | **0** | **Invariable** | **cox1** |
| **991** | **1.374** | **0** | **0.206** | **15.344** | **0.0001** | **11.575** | **Purifying** | **cox1** |
| **992** | **0** | **0** | **0** | **0** | **1** | **0** | **Invariable** | **cox1** |
| **993** | **2.924** | **0** | **0.38** | **28.458** | **0** | **21.331** | **Purifying** | **cox1** |
| **994** | **3.069** | **0** | **0.92** | **17.843** | **0** | **51.711** | **Purifying** | **cox1** |
| **995** | **0.66** | **0** | **0.017** | **8.339** | **0.0039** | **0.93** | **Purifying** | **cox1** |
| **996** | **0.649** | **0** | **0.006** | **5.235** | **0.0221** | **0.324** | **Purifying** | **cox1** |
| **997** | **1.167** | **0** | **0.022** | **13.672** | **0.0002** | **1.231** | **Purifying** | **cox1** |
| **998** | **1.081** | **0** | **0.019** | **13.189** | **0.0003** | **1.068** | **Purifying** | **cox1** |
| **999** | **1.214** | **0** | **0.49** | **13.756** | **0.0002** | **27.51** | **Purifying** | **cox1** |
| **1,000** | **1.469** | **0** | **0.016** | **14.416** | **0.0001** | **0.886** | **Purifying** | **cox1** |
| **1,001** | **0.479** | **0** | **0.006** | **4.668** | **0.0307** | **0.355** | **Purifying** | **cox1** |
| **1,002** | **1.128** | **0** | **0.013** | **12.144** | **0.0005** | **0.722** | **Purifying** | **cox1** |
| **1,003** | **0.545** | **0** | **0.006** | **3.032** | **0.0816** | **0.318** | **Neutral** | **cox1** |
| **1,004** | **1.666** | **0** | **0.486** | **13.374** | **0.0003** | **27.285** | **Purifying** | **cox1** |
| **1,005** | **5.272** | **0** | **0.317** | **27.745** | **0** | **17.811** | **Purifying** | **cox1** |
| **1,006** | **0.919** | **0** | **0.134** | **13.892** | **0.0002** | **7.549** | **Purifying** | **cox1** |
| **1,007** | **3.517** | **0** | **0.521** | **22.747** | **0** | **29.296** | **Purifying** | **cox1** |
| **1,008** | **0.781** | **0** | **0.243** | **11.198** | **0.0008** | **13.642** | **Purifying** | **cox1** |
| **1,009** | **2.745** | **0** | **0.022** | **16.417** | **0.0001** | **1.237** | **Purifying** | **cox1** |
| **1,010** | **10000** | **0** | **0.316** | **30.984** | **0** | **17.745** | **Purifying** | **cox1** |
| **1,011** | **0** | **0** | **0** | **0** | **1** | **0** | **Invariable** | **cox1** |
| **1,012** | **0.883** | **0** | **0.008** | **7.542** | **0.006** | **0.463** | **Purifying** | **cox1** |
| **1,013** | **0.681** | **0** | **0.007** | **5.067** | **0.0244** | **0.394** | **Purifying** | **cox1** |
| **1,014** | **0** | **0** | **0** | **0** | **1** | **0** | **Invariable** | **cox1** |
| **1,015** | **0.023** | **0** | **0.006** | **2.442** | **0.1181** | **0.316** | **Neutral** | **cox1** |
| **1,016** | **0** | **0** | **0** | **0** | **1** | **0** | **Invariable** | **cox1** |
| **1,017** | **1.261** | **0** | **0.017** | **11.19** | **0.0008** | **0.98** | **Purifying** | **cox1** |
| **1,018** | **0** | **0** | **0** | **0** | **1** | **0** | **Invariable** | **cox1** |
| **1,019** | **1.308** | **0** | **0.022** | **15.107** | **0.0001** | **1.235** | **Purifying** | **cox1** |
| **1,020** | **0** | **0** | **0** | **0** | **1** | **0** | **Invariable** | **cox1** |
| **1,021** | **0** | **0** | **0** | **0** | **1** | **0** | **Invariable** | **cox1** |
| **1,022** | **1.085** | **0** | **0.018** | **10.226** | **0.0014** | **0.988** | **Purifying** | **cox1** |
| **1,023** | **0.713** | **0.009** | **0.294** | **6.945** | **0.0084** | **16.528** | **Purifying** | **cox1** |
| **1,024** | **0** | **0** | **0** | **0** | **1** | **0** | **Invariable** | **cox1** |
| **1,025** | **0.6** | **0** | **0.005** | **3.499** | **0.0614** | **0.254** | **Neutral** | **cox1** |
| **1,026** | **0** | **0** | **0** | **0** | **1** | **0** | **Invariable** | **cox1** |
| **1,027** | **1.136** | **0** | **0.007** | **5.724** | **0.0167** | **0.396** | **Purifying** | **cox1** |
| **1,028** | **1.727** | **0** | **0.015** | **17.324** | **0** | **0.825** | **Purifying** | **cox1** |
| **1,029** | **0** | **0** | **0** | **0** | **1** | **0** | **Invariable** | **cox1** |
| **1,030** | **0.029** | **0** | **0.006** | **2.55** | **0.1103** | **0.348** | **Neutral** | **cox1** |
| **1,031** | **0.453** | **0** | **0.006** | **2.659** | **0.103** | **0.318** | **Neutral** | **cox1** |
| **1,032** | **0.534** | **0** | **0.006** | **4.849** | **0.0277** | **0.359** | **Purifying** | **cox1** |
| **1,033** | **0.023** | **0** | **0.005** | **2.566** | **0.1092** | **0.295** | **Neutral** | **cox1** |
| **1,034** | **1.074** | **0** | **0.016** | **10.757** | **0.001** | **0.922** | **Purifying** | **cox1** |
| **1,035** | **2.462** | **0** | **0.465** | **20.376** | **0** | **26.099** | **Purifying** | **cox1** |
| **1,036** | **0** | **0** | **0** | **0** | **1** | **0** | **Invariable** | **cox1** |
| **1,037** | **0** | **0** | **0** | **0** | **1** | **0** | **Invariable** | **cox1** |
| **1,038** | **1.436** | **0** | **0.017** | **13.278** | **0.0003** | **0.954** | **Purifying** | **cox1** |
| **1,039** | **0** | **0** | **0** | **0** | **1** | **0** | **Invariable** | **cox1** |
| **1,040** | **4.557** | **0** | **0.374** | **23.995** | **0** | **20.997** | **Purifying** | **cox1** |
| **1,041** | **0** | **0** | **0** | **0** | **1** | **0** | **Invariable** | **cox1** |
| **1,042** | **1.606** | **0** | **0.018** | **19.293** | **0** | **1.035** | **Purifying** | **cox1** |
| **1,043** | **7.242** | **0** | **0.422** | **28.233** | **0** | **23.725** | **Purifying** | **cox1** |
| **1,044** | **1.817** | **0** | **0.52** | **21.356** | **0** | **29.231** | **Purifying** | **cox1** |
| **1,045** | **0.022** | **0** | **0.007** | **2.01** | **0.1562** | **0.393** | **Neutral** | **cox1** |
| **1,046** | **3.222** | **0** | **0.022** | **16.925** | **0** | **1.249** | **Purifying** | **cox1** |
| **1,047** | **1.043** | **0** | **0.006** | **8.494** | **0.0036** | **0.311** | **Purifying** | **cox1** |
| **1,048** | **0.023** | **0** | **0.006** | **2.416** | **0.1201** | **0.323** | **Neutral** | **cox1** |
| **1,049** | **0.023** | **0** | **0.006** | **2.393** | **0.1219** | **0.322** | **Neutral** | **cox1** |
| **1,050** | **0** | **0** | **0** | **0** | **1** | **0** | **Invariable** | **cox1** |
| **1,051** | **1.454** | **0** | **0.022** | **13.937** | **0.0002** | **1.233** | **Purifying** | **cox1** |
| **1,052** | **0** | **0** | **0** | **0** | **1** | **0** | **Invariable** | **cox1** |
| **1,053** | **2.804** | **0** | **0.014** | **16.885** | **0** | **0.804** | **Purifying** | **cox1** |
| **1,054** | **1.527** | **0** | **0.286** | **23.63** | **0** | **16.063** | **Purifying** | **cox1** |
| **1,055** | **0.335** | **0** | **0.016** | **7.915** | **0.0049** | **0.897** | **Purifying** | **cox1** |
| **1,056** | **0** | **0** | **0** | **0** | **1** | **0** | **Invariable** | **cox1** |
| **1,057** | **0.987** | **0** | **0.007** | **7.894** | **0.005** | **0.407** | **Purifying** | **cox1** |
| **1,058** | **0.647** | **0** | **0.007** | **4.929** | **0.0264** | **0.394** | **Purifying** | **cox1** |
| **1,059** | **0.691** | **0** | **0.035** | **18.56** | **0** | **1.968** | **Purifying** | **cox1** |
| **1,060** | **0** | **0** | **0** | **0** | **1** | **0** | **Invariable** | **cox1** |
| **1,061** | **0.943** | **0** | **0.008** | **7.761** | **0.0053** | **0.43** | **Purifying** | **cox1** |
| **1,062** | **0.337** | **0** | **0.009** | **2.385** | **0.1225** | **0.522** | **Neutral** | **cox1** |
| **1,063** | **0.023** | **0** | **0.006** | **2.396** | **0.1216** | **0.322** | **Neutral** | **cox1** |
| **1,064** | **0** | **0** | **0** | **0** | **1** | **0** | **Invariable** | **cox1** |
| **1,065** | **0** | **0** | **0** | **0** | **1** | **0** | **Invariable** | **cox1** |
| **1,066** | **0.772** | **0** | **0.008** | **4.893** | **0.027** | **0.461** | **Purifying** | **cox1** |
| **1,067** | **2.501** | **0** | **0.012** | **9.177** | **0.0025** | **0.663** | **Purifying** | **cox1** |
| **1,068** | **2.467** | **0** | **0.022** | **16.683** | **0** | **1.249** | **Purifying** | **cox1** |
| **1,069** | **0** | **0** | **0** | **0** | **1** | **0** | **Invariable** | **cox1** |
| **1,070** | **0.715** | **0** | **0.017** | **8.418** | **0.0037** | **0.97** | **Purifying** | **cox1** |
| **1,071** | **0.515** | **0** | **0.005** | **5.373** | **0.0205** | **0.286** | **Purifying** | **cox1** |
| **1,072** | **2.373** | **0** | **0.511** | **16.022** | **0.0001** | **28.707** | **Purifying** | **cox1** |
| **1,073** | **0.75** | **0** | **0.012** | **10.626** | **0.0011** | **0.694** | **Purifying** | **cox1** |
| **1,074** | **0** | **0** | **0** | **0** | **1** | **0** | **Invariable** | **cox1** |
| **1,075** | **0** | **0** | **0** | **0** | **1** | **0** | **Invariable** | **cox1** |
| **1,076** | **0** | **0** | **0** | **0** | **1** | **0** | **Invariable** | **cox1** |
| **1,077** | **1.587** | **0** | **0.017** | **12.388** | **0.0004** | **0.947** | **Purifying** | **cox1** |
| **1,078** | **0.44** | **0** | **0.008** | **3.498** | **0.0614** | **0.424** | **Neutral** | **cox1** |
| **1,079** | **1.105** | **0** | **0.008** | **7.822** | **0.0052** | **0.463** | **Purifying** | **cox1** |
| **1,080** | **0** | **0** | **0** | **0** | **1** | **0** | **Invariable** | **cox1** |
| **1,081** | **12.436** | **0** | **0.018** | **20.622** | **0** | **0.998** | **Purifying** | **cox1** |
| **1,082** | **10000** | **0** | **0.018** | **19.912** | **0** | **0.993** | **Purifying** | **cox1** |
| **1,083** | **0** | **0** | **0** | **0** | **1** | **0** | **Invariable** | **cox1** |
| **1,084** | **0.888** | **0** | **0.007** | **7.938** | **0.0048** | **0.384** | **Purifying** | **cox1** |
| **1,085** | **0.715** | **0** | **0.015** | **9.954** | **0.0016** | **0.853** | **Purifying** | **cox1** |
| **1,086** | **1.083** | **0** | **0.016** | **13.07** | **0.0003** | **0.897** | **Purifying** | **cox1** |
| **1,087** | **1.925** | **0** | **0.35** | **19.642** | **0** | **19.662** | **Purifying** | **cox1** |
| **1,088** | **3.225** | **0** | **0.908** | **18.546** | **0** | **51.04** | **Purifying** | **cox1** |
| **1,089** | **1.083** | **0** | **0.015** | **13.464** | **0.0002** | **0.84** | **Purifying** | **cox1** |
| **1,090** | **1.958** | **0** | **0.019** | **14.545** | **0.0001** | **1.062** | **Purifying** | **cox1** |
| **1,091** | **1.941** | **0** | **0.349** | **18.37** | **0** | **19.611** | **Purifying** | **cox1** |
| **1,092** | **0.513** | **0** | **0.007** | **4.871** | **0.0273** | **0.371** | **Purifying** | **cox1** |
| **1,093** | **0** | **0** | **0** | **0** | **1** | **0** | **Invariable** | **cox1** |
| **1,094** | **1.584** | **0** | **0.342** | **17.274** | **0** | **19.241** | **Purifying** | **cox1** |
| **1,095** | **1.577** | **0** | **0.255** | **23.453** | **0** | **14.316** | **Purifying** | **cox1** |
| **1,096** | **0.507** | **0** | **0.007** | **4.837** | **0.0279** | **0.371** | **Purifying** | **cox1** |
| **1,097** | **1.242** | **0** | **0.402** | **12.245** | **0.0005** | **22.611** | **Purifying** | **cox1** |
| **1,098** | **0.029** | **0** | **0.006** | **2.552** | **0.1101** | **0.348** | **Neutral** | **cox1** |
| **1,099** | **1.282** | **0** | **0.308** | **18.813** | **0** | **17.281** | **Purifying** | **cox1** |
| **1,100** | **0.818** | **0** | **0.017** | **8.702** | **0.0032** | **0.973** | **Purifying** | **cox1** |
| **1,101** | **0** | **0** | **0** | **0** | **1** | **0** | **Invariable** | **cox1** |
| **1,102** | **0** | **0** | **0** | **0** | **1** | **0** | **Invariable** | **cox1** |
| **1,103** | **0.685** | **0** | **0.005** | **3.233** | **0.0722** | **0.287** | **Neutral** | **cox1** |
| **1,104** | **0** | **0** | **0** | **0** | **1** | **0** | **Invariable** | **cox1** |
| **1,105** | **0** | **0.012** | **0.011** | **0.093** | **0.7604** | **0.602** | **Neutral** | **cox1** |
| **1,106** | **0** | **0** | **0** | **0** | **1** | **0** | **Invariable** | **cox1** |
| **1,107** | **0.857** | **0** | **0.028** | **9.52** | **0.002** | **1.598** | **Purifying** | **cox1** |
| **1,108** | **10000** | **0** | **0.395** | **29.569** | **0** | **22.185** | **Purifying** | **cox1** |
| **1,109** | **1.064** | **0** | **0.015** | **11.243** | **0.0008** | **0.868** | **Purifying** | **cox1** |
| **1,110** | **0.579** | **0** | **0.008** | **5.269** | **0.0217** | **0.423** | **Purifying** | **cox1** |
| **1,111** | **0** | **0** | **0** | **0** | **1** | **0** | **Invariable** | **cox1** |
| **1,112** | **0.545** | **0** | **0.005** | **3.189** | **0.0741** | **0.297** | **Neutral** | **cox1** |
| **1,113** | **2.066** | **0** | **0.014** | **15.486** | **0.0001** | **0.801** | **Purifying** | **cox1** |
| **1,114** | **0.024** | **0** | **0.005** | **2.587** | **0.1078** | **0.296** | **Neutral** | **cox1** |
| **1,115** | **0** | **0** | **0** | **0** | **1** | **0** | **Invariable** | **cox1** |
| **1,116** | **0** | **0** | **0** | **0** | **1** | **0** | **Invariable** | **cox1** |
| **1,117** | **0** | **0** | **0** | **0** | **1** | **0** | **Invariable** | **cox1** |
| **1,118** | **0** | **0** | **0** | **0** | **1** | **0** | **Invariable** | **cox1** |
| **1,119** | **0** | **0** | **0** | **0** | **1** | **0** | **Invariable** | **cox1** |
| **1,120** | **1.444** | **0** | **0.277** | **12.727** | **0.0004** | **15.566** | **Purifying** | **cox1** |
| **1,121** | **0** | **0** | **0** | **0** | **1** | **0** | **Invariable** | **cox1** |
| **1,122** | **0.75** | **0** | **0.014** | **10.329** | **0.0013** | **0.778** | **Purifying** | **cox1** |
| **1,123** | **3.54** | **0** | **0.022** | **19.896** | **0** | **1.235** | **Purifying** | **cox1** |
| **1,124** | **0.455** | **0** | **0.009** | **3.679** | **0.0551** | **0.491** | **Neutral** | **cox1** |
| **1,125** | **0** | **0** | **0** | **0** | **1** | **0** | **Invariable** | **cox1** |
| **1,126** | **0** | **0** | **0** | **0** | **1** | **0** | **Invariable** | **cox1** |
| **1,127** | **0** | **0** | **0** | **0** | **1** | **0** | **Invariable** | **cox1** |
| **1,128** | **3.461** | **0** | **0.354** | **25.43** | **0** | **19.906** | **Purifying** | **cox1** |
| **1,129** | **0.618** | **0** | **0.01** | **6.694** | **0.0097** | **0.563** | **Purifying** | **cox1** |
| **1,130** | **0** | **0** | **0** | **0** | **1** | **0** | **Invariable** | **cox1** |
| **1,131** | **0.008** | **0** | **0.005** | **0.906** | **0.3413** | **0.298** | **Neutral** | **cox1** |
| **1,132** | **4.187** | **0** | **0.022** | **17.588** | **0** | **1.249** | **Purifying** | **cox1** |
| **1,133** | **0** | **0** | **0** | **0** | **1** | **0** | **Invariable** | **cox1** |
| **1,134** | **0** | **0** | **0** | **0** | **1** | **0** | **Invariable** | **cox1** |
| **1,135** | **0** | **0** | **0** | **0** | **1** | **0** | **Invariable** | **cox1** |
| **1,136** | **1.669** | **0** | **0.015** | **12.517** | **0.0004** | **0.856** | **Purifying** | **cox1** |
| **1,137** | **1.385** | **0** | **0.015** | **12.461** | **0.0004** | **0.859** | **Purifying** | **cox1** |
| **1,138** | **0.943** | **0** | **0.007** | **5.854** | **0.0155** | **0.396** | **Purifying** | **cox1** |
| **1,139** | **0** | **0** | **0** | **0** | **1** | **0** | **Invariable** | **cox1** |
| **1,140** | **0** | **0** | **0** | **0** | **1** | **0** | **Invariable** | **cox1** |
| **1,141** | **0.943** | **0** | **0.006** | **6.152** | **0.0131** | **0.343** | **Purifying** | **cox1** |
| **1,142** | **0.45** | **0** | **0.008** | **7.39** | **0.0066** | **0.45** | **Purifying** | **cox1** |
| **1,143** | **0** | **0** | **0** | **0** | **1** | **0** | **Invariable** | **cox1** |
| **1,144** | **0.023** | **0** | **0.006** | **2.176** | **0.1402** | **0.36** | **Neutral** | **cox1** |
| **1,145** | **0** | **0** | **0** | **0** | **1** | **0** | **Invariable** | **cox1** |
| **1,146** | **0** | **0** | **0** | **0** | **1** | **0** | **Invariable** | **cox1** |
| **1,147** | **1.25** | **0** | **0.306** | **16.311** | **0.0001** | **17.209** | **Purifying** | **cox1** |
| **1,148** | **0** | **0** | **0** | **0** | **1** | **0** | **Invariable** | **cox1** |
| **1,149** | **0.734** | **0** | **0.014** | **10.25** | **0.0014** | **0.777** | **Purifying** | **cox1** |
| **1,150** | **1.221** | **0** | **0.012** | **9.666** | **0.0019** | **0.675** | **Purifying** | **cox1** |
| **1,151** | **1.902** | **0.008** | **0.322** | **8.163** | **0.0043** | **18.119** | **Purifying** | **cox1** |
| **1,152** | **0** | **0** | **0** | **0** | **1** | **0** | **Invariable** | **cox1** |
| **1,153** | **0** | **0** | **0** | **0** | **1** | **0** | **Invariable** | **cox2** |
| **1,154** | **0.68** | **0** | **0.006** | **5.135** | **0.0234** | **0.359** | **Purifying** | **cox2** |
| **1,155** | **0** | **0** | **0** | **0** | **1** | **0** | **Invariable** | **cox2** |
| **1,156** | **0** | **0** | **0** | **0** | **1** | **0** | **Invariable** | **cox2** |
| **1,157** | **0** | **0** | **0** | **0** | **1** | **0** | **Invariable** | **cox2** |
| **1,158** | **0** | **0** | **0** | **0** | **1** | **0** | **Invariable** | **cox2** |
| **1,159** | **0.337** | **0** | **0.009** | **2.361** | **0.1244** | **0.521** | **Neutral** | **cox2** |
| **1,160** | **0** | **0** | **0** | **0** | **1** | **0** | **Invariable** | **cox2** |
| **1,161** | **0** | **0** | **0** | **0** | **1** | **0** | **Invariable** | **cox2** |
| **1,162** | **0** | **0** | **0** | **0** | **1** | **0** | **Invariable** | **cox2** |
| **1,163** | **0** | **0** | **0** | **0** | **1** | **0** | **Invariable** | **cox2** |
| **1,164** | **0** | **0** | **0** | **0** | **1** | **0** | **Invariable** | **cox2** |
| **1,165** | **0** | **0** | **0** | **0** | **1** | **0** | **Invariable** | **cox2** |
| **1,166** | **0** | **0** | **0** | **0** | **1** | **0** | **Invariable** | **cox2** |
| **1,167** | **0** | **0** | **0** | **0** | **1** | **0** | **Invariable** | **cox2** |
| **1,168** | **0.547** | **0** | **0.007** | **4.973** | **0.0257** | **0.376** | **Purifying** | **cox2** |
| **1,169** | **0** | **0** | **0** | **0** | **1** | **0** | **Invariable** | **cox2** |
| **1,170** | **0** | **0** | **0** | **0** | **1** | **0** | **Invariable** | **cox2** |
| **1,171** | **0** | **0** | **0** | **0** | **1** | **0** | **Invariable** | **cox2** |
| **1,172** | **0** | **0** | **0** | **0** | **1** | **0** | **Invariable** | **cox2** |
| **1,173** | **0** | **0** | **0** | **0** | **1** | **0** | **Invariable** | **cox2** |
| **1,174** | **0.729** | **0** | **0.009** | **7.117** | **0.0076** | **0.494** | **Purifying** | **cox2** |
| **1,175** | **1.633** | **0** | **0.007** | **8.686** | **0.0032** | **0.409** | **Purifying** | **cox2** |
| **1,176** | **0.729** | **0** | **0.009** | **7.117** | **0.0076** | **0.494** | **Purifying** | **cox2** |
| **1,177** | **0** | **0** | **0** | **0** | **1** | **0** | **Invariable** | **cox2** |
| **1,178** | **0.491** | **0** | **0.007** | **4.466** | **0.0346** | **0.4** | **Purifying** | **cox2** |
| **1,179** | **0** | **0** | **0** | **0** | **1** | **0** | **Invariable** | **cox2** |
| **1,180** | **0.333** | **0** | **0.009** | **2.36** | **0.1245** | **0.521** | **Neutral** | **cox2** |
| **1,181** | **0** | **0.01** | **0.008** | **0.296** | **0.5867** | **0.445** | **Neutral** | **cox2** |
| **1,182** | **0.805** | **0** | **0.148** | **13.586** | **0.0002** | **8.316** | **Purifying** | **cox2** |
| **1,183** | **0** | **0** | **0** | **0** | **1** | **0** | **Invariable** | **cox2** |
| **1,184** | **0** | **0** | **0** | **0** | **1** | **0** | **Invariable** | **cox2** |
| **1,185** | **1.058** | **0** | **0.028** | **10.307** | **0.0013** | **1.6** | **Purifying** | **cox2** |
| **1,186** | **0** | **0** | **0** | **0** | **1** | **0** | **Invariable** | **cox2** |
| **1,187** | **0** | **0** | **0** | **0** | **1** | **0** | **Invariable** | **cox2** |
| **1,188** | **0** | **0** | **0** | **0** | **1** | **0** | **Invariable** | **cox2** |
| **1,189** | **1.249** | **0** | **0.285** | **12.01** | **0.0005** | **16.024** | **Purifying** | **cox2** |
| **1,190** | **0** | **0** | **0** | **0** | **1** | **0** | **Invariable** | **cox2** |
| **1,191** | **0** | **0** | **0** | **0** | **1** | **0** | **Invariable** | **cox2** |
| **1,192** | **0.725** | **0** | **0.008** | **7.234** | **0.0072** | **0.462** | **Purifying** | **cox2** |
| **1,193** | **0** | **0** | **0** | **0** | **1** | **0** | **Invariable** | **cox2** |
| **1,194** | **0** | **0** | **0** | **0** | **1** | **0** | **Invariable** | **cox2** |
| **1,195** | **0** | **0** | **0** | **0** | **1** | **0** | **Invariable** | **cox2** |
| **1,196** | **3.955** | **0** | **0.582** | **26.897** | **0** | **32.713** | **Purifying** | **cox2** |
| **1,197** | **0** | **0** | **0** | **0** | **1** | **0** | **Invariable** | **cox2** |
| **1,198** | **0.545** | **0** | **0.006** | **3.068** | **0.0799** | **0.319** | **Neutral** | **cox2** |
| **1,199** | **0** | **0** | **0** | **0** | **1** | **0** | **Invariable** | **cox2** |
| **1,200** | **0.534** | **0** | **0.007** | **4.644** | **0.0312** | **0.4** | **Purifying** | **cox2** |
| **1,201** | **0** | **0** | **0** | **0** | **1** | **0** | **Invariable** | **cox2** |
| **1,202** | **1.442** | **0** | **0.593** | **14.615** | **0.0001** | **33.328** | **Purifying** | **cox2** |
| **1,203** | **0** | **0** | **0** | **0** | **1** | **0** | **Invariable** | **cox2** |
| **1,204** | **0** | **0** | **0** | **0** | **1** | **0** | **Invariable** | **cox2** |
| **1,205** | **0** | **0** | **0** | **0** | **1** | **0** | **Invariable** | **cox2** |
| **1,206** | **1.416** | **0** | **0.016** | **13.777** | **0.0002** | **0.899** | **Purifying** | **cox2** |
| **1,207** | **0.579** | **0** | **0.008** | **5.269** | **0.0217** | **0.423** | **Purifying** | **cox2** |
| **1,208** | **0.856** | **0** | **0.023** | **8.752** | **0.0031** | **1.283** | **Purifying** | **cox2** |
| **1,209** | **0** | **0** | **0** | **0** | **1** | **0** | **Invariable** | **cox2** |
| **1,210** | **3.957** | **0** | **0.521** | **25.334** | **0** | **29.261** | **Purifying** | **cox2** |
| **1,211** | **9.04** | **0** | **0.012** | **10.265** | **0.0014** | **0.664** | **Purifying** | **cox2** |
| **1,212** | **0** | **0** | **0** | **0** | **1** | **0** | **Invariable** | **cox2** |
| **1,213** | **0.816** | **0** | **0.015** | **10.639** | **0.0011** | **0.865** | **Purifying** | **cox2** |
| **1,214** | **0.022** | **0** | **0.005** | **2.575** | **0.1086** | **0.29** | **Neutral** | **cox2** |
| **1,215** | **0** | **0** | **0** | **0** | **1** | **0** | **Invariable** | **cox2** |
| **1,216** | **0** | **0** | **0** | **0** | **1** | **0** | **Invariable** | **cox2** |
| **1,217** | **0.55** | **0** | **0.007** | **4.639** | **0.0313** | **0.4** | **Purifying** | **cox2** |
| **1,218** | **0** | **0** | **0** | **0** | **1** | **0** | **Invariable** | **cox2** |
| **1,219** | **0.727** | **0** | **0.017** | **8.493** | **0.0036** | **0.971** | **Purifying** | **cox2** |
| **1,220** | **0.547** | **0** | **0.008** | **4.36** | **0.0368** | **0.459** | **Purifying** | **cox2** |
| **1,221** | **0.542** | **0** | **0.006** | **4.845** | **0.0277** | **0.359** | **Purifying** | **cox2** |
| **1,222** | **1.3** | **0** | **0.165** | **14.694** | **0.0001** | **9.247** | **Purifying** | **cox2** |
| **1,223** | **1.488** | **0** | **0.022** | **14.241** | **0.0002** | **1.239** | **Purifying** | **cox2** |
| **1,224** | **1.041** | **0** | **0.025** | **10.689** | **0.0011** | **1.397** | **Purifying** | **cox2** |
| **1,225** | **0.827** | **0.009** | **0.026** | **7.233** | **0.0072** | **1.434** | **Purifying** | **cox2** |
| **1,226** | **0.511** | **0** | **0.014** | **7.183** | **0.0074** | **0.775** | **Purifying** | **cox2** |
| **1,227** | **0** | **0** | **0** | **0** | **1** | **0** | **Invariable** | **cox2** |
| **1,228** | **0** | **0** | **0** | **0** | **1** | **0** | **Invariable** | **cox2** |
| **1,229** | **1.086** | **0** | **0.527** | **12.692** | **0.0004** | **29.592** | **Purifying** | **cox2** |
| **1,230** | **0** | **0** | **0** | **0** | **1** | **0** | **Invariable** | **cox2** |
| **1,231** | **0.745** | **0** | **0.008** | **4.864** | **0.0274** | **0.422** | **Purifying** | **cox2** |
| **1,232** | **0.291** | **0** | **0.009** | **2.206** | **0.1374** | **0.52** | **Neutral** | **cox2** |
| **1,233** | **0.706** | **0** | **0.012** | **9.764** | **0.0018** | **0.654** | **Purifying** | **cox2** |
| **1,234** | **1.467** | **0** | **0.018** | **14.189** | **0.0002** | **1.002** | **Purifying** | **cox2** |
| **1,235** | **0.023** | **0** | **0.007** | **2.012** | **0.1561** | **0.393** | **Neutral** | **cox2** |
| **1,236** | **0** | **0** | **0** | **0** | **1** | **0** | **Invariable** | **cox2** |
| **1,237** | **0** | **0** | **0** | **0** | **1** | **0** | **Invariable** | **cox2** |
| **1,238** | **0** | **0.007** | **0.007** | **0.17** | **0.6803** | **0.374** | **Neutral** | **cox2** |
| **1,239** | **2.928** | **0** | **0.031** | **24.484** | **0** | **1.738** | **Purifying** | **cox2** |
| **1,240** | **0** | **0** | **0** | **0** | **1** | **0** | **Invariable** | **cox2** |
| **1,241** | **1.14** | **0** | **0.022** | **13.286** | **0.0003** | **1.224** | **Purifying** | **cox2** |
| **1,242** | **2.09** | **0** | **0.015** | **14.525** | **0.0001** | **0.842** | **Purifying** | **cox2** |
| **1,243** | **0.943** | **0** | **0.007** | **7.98** | **0.0047** | **0.384** | **Purifying** | **cox2** |
| **1,244** | **2.667** | **0** | **0.409** | **19.05** | **0** | **22.995** | **Purifying** | **cox2** |
| **1,245** | **0.883** | **0** | **0.009** | **7.382** | **0.0066** | **0.495** | **Purifying** | **cox2** |
| **1,246** | **1.468** | **0** | **0.491** | **13.227** | **0.0003** | **27.599** | **Purifying** | **cox2** |
| **1,247** | **0** | **0** | **0** | **0** | **1** | **0** | **Invariable** | **cox2** |
| **1,248** | **0** | **0** | **0** | **0** | **1** | **0** | **Invariable** | **cox2** |
| **1,249** | **0** | **0** | **0** | **0** | **1** | **0** | **Invariable** | **cox2** |
| **1,250** | **0** | **0** | **0** | **0** | **1** | **0** | **Invariable** | **cox2** |
| **1,251** | **0** | **0.105** | **0.024** | **1.157** | **0.282** | **1.333** | **Neutral** | **cox2** |
| **1,252** | **0.665** | **0** | **0.006** | **5.272** | **0.0217** | **0.325** | **Purifying** | **cox2** |
| **1,253** | **2.852** | **0** | **0.501** | **24.408** | **0** | **28.137** | **Purifying** | **cox2** |
| **1,254** | **10000** | **0** | **0.029** | **22.043** | **0** | **1.628** | **Purifying** | **cox2** |
| **1,255** | **0** | **0** | **0** | **0** | **1** | **0** | **Invariable** | **cox2** |
| **1,256** | **0** | **0** | **0** | **0** | **1** | **0** | **Invariable** | **cox2** |
| **1,257** | **1.117** | **0** | **0.016** | **13.127** | **0.0003** | **0.897** | **Purifying** | **cox2** |
| **1,258** | **0.868** | **0** | **0.005** | **3.975** | **0.0462** | **0.28** | **Purifying** | **cox2** |
| **1,259** | **5.076** | **0** | **0.406** | **27.938** | **0** | **22.79** | **Purifying** | **cox2** |
| **1,260** | **0.022** | **0** | **0.006** | **2.394** | **0.1218** | **0.322** | **Neutral** | **cox2** |
| **1,261** | **4.238** | **0** | **0.44** | **28.274** | **0** | **24.714** | **Purifying** | **cox2** |
| **1,262** | **1.019** | **0** | **0.008** | **7.695** | **0.0055** | **0.463** | **Purifying** | **cox2** |
| **1,263** | **0** | **0** | **0** | **0** | **1** | **0** | **Invariable** | **cox2** |
| **1,264** | **0** | **0** | **0** | **0** | **1** | **0** | **Invariable** | **cox2** |
| **1,265** | **2.123** | **0** | **0.513** | **14.97** | **0.0001** | **28.824** | **Purifying** | **cox2** |
| **1,266** | **1.804** | **0** | **0.027** | **22.913** | **0** | **1.501** | **Purifying** | **cox2** |
| **1,267** | **0.995** | **0** | **0.006** | **6.711** | **0.0096** | **0.344** | **Purifying** | **cox2** |
| **1,268** | **10000** | **0** | **0.018** | **19.872** | **0** | **0.998** | **Purifying** | **cox2** |
| **1,269** | **1.449** | **0** | **0.019** | **13.574** | **0.0002** | **1.039** | **Purifying** | **cox2** |
| **1,270** | **0** | **0** | **0** | **0** | **1** | **0** | **Invariable** | **cox2** |
| **1,271** | **0** | **0.021** | **0.013** | **0.36** | **0.5486** | **0.739** | **Neutral** | **cox2** |
| **1,272** | **0.798** | **0** | **0.008** | **5.771** | **0.0163** | **0.425** | **Purifying** | **cox2** |
| **1,273** | **3.169** | **0** | **0.588** | **22.079** | **0** | **33.034** | **Purifying** | **cox2** |
| **1,274** | **0** | **0** | **0** | **0** | **1** | **0** | **Invariable** | **cox2** |
| **1,275** | **0** | **0** | **0** | **0** | **1** | **0** | **Invariable** | **cox2** |
| **1,276** | **0** | **0** | **0** | **0** | **1** | **0** | **Invariable** | **cox2** |
| **1,277** | **2.492** | **0** | **0.683** | **16.188** | **0.0001** | **38.367** | **Purifying** | **cox2** |
| **1,278** | **1.866** | **0** | **0.019** | **14.786** | **0.0001** | **1.066** | **Purifying** | **cox2** |
| **1,279** | **1.489** | **0** | **0.375** | **17.289** | **0** | **21.067** | **Purifying** | **cox2** |
| **1,280** | **0.615** | **0** | **0.006** | **5.143** | **0.0233** | **0.324** | **Purifying** | **cox2** |
| **1,281** | **1.398** | **0** | **0.028** | **15.437** | **0.0001** | **1.591** | **Purifying** | **cox2** |
| **1,282** | **0** | **0** | **0** | **0** | **1** | **0** | **Invariable** | **cox2** |
| **1,283** | **0.022** | **0** | **0.005** | **2.5** | **0.1138** | **0.297** | **Neutral** | **cox2** |
| **1,284** | **2.022** | **0** | **0.655** | **15.37** | **0.0001** | **36.789** | **Purifying** | **cox2** |
| **1,285** | **0** | **0** | **0** | **0** | **1** | **0** | **Invariable** | **cox2** |
| **1,286** | **0** | **0** | **0** | **0** | **1** | **0** | **Invariable** | **cox2** |
| **1,287** | **0** | **0** | **0** | **0** | **1** | **0** | **Invariable** | **cox2** |
| **1,288** | **10000** | **0** | **0.508** | **33.168** | **0** | **28.54** | **Purifying** | **cox2** |
| **1,289** | **0.943** | **0** | **0.007** | **5.823** | **0.0158** | **0.404** | **Purifying** | **cox2** |
| **1,290** | **3.035** | **0** | **0.516** | **26.374** | **0** | **28.967** | **Purifying** | **cox2** |
| **1,291** | **0** | **0** | **0** | **0** | **1** | **0** | **Invariable** | **cox2** |
| **1,292** | **2.948** | **0** | **0.021** | **15.994** | **0.0001** | **1.195** | **Purifying** | **cox2** |
| **1,293** | **1.365** | **0** | **0.333** | **16.804** | **0** | **18.707** | **Purifying** | **cox2** |
| **1,294** | **1.681** | **0** | **0.268** | **17.18** | **0** | **15.057** | **Purifying** | **cox2** |
| **1,295** | **0.81** | **0** | **0.014** | **10.62** | **0.0011** | **0.779** | **Purifying** | **cox2** |
| **1,296** | **8.809** | **0** | **0.008** | **10.494** | **0.0012** | **0.46** | **Purifying** | **cox2** |
| **1,297** | **1.385** | **0** | **0.016** | **14.171** | **0.0002** | **0.892** | **Purifying** | **cox2** |
| **1,298** | **2.109** | **0** | **0.619** | **20.555** | **0** | **34.764** | **Purifying** | **cox2** |
| **1,299** | **0.029** | **0** | **0.006** | **2.549** | **0.1104** | **0.348** | **Neutral** | **cox2** |
| **1,300** | **0.05** | **0** | **0.007** | **3.031** | **0.0817** | **0.419** | **Neutral** | **cox2** |
| **1,301** | **0.4** | **0** | **0.014** | **8.116** | **0.0044** | **0.808** | **Purifying** | **cox2** |
| **1,302** | **0.426** | **0** | **0.009** | **2.695** | **0.1007** | **0.522** | **Neutral** | **cox2** |
| **1,303** | **0.636** | **0** | **0.007** | **5.239** | **0.0221** | **0.377** | **Purifying** | **cox2** |
| **1,304** | **0.023** | **0** | **0.006** | **2.407** | **0.1208** | **0.315** | **Neutral** | **cox2** |
| **1,305** | **0** | **0** | **0** | **0** | **1** | **0** | **Invariable** | **cox2** |
| **1,306** | **0** | **0** | **0** | **0** | **1** | **0** | **Invariable** | **cox2** |
| **1,307** | **7.654** | **0** | **0.285** | **26.671** | **0** | **16.004** | **Purifying** | **cox2** |
| **1,308** | **0.753** | **0** | **0.018** | **8.527** | **0.0035** | **0.989** | **Purifying** | **cox2** |
| **1,309** | **2.089** | **0** | **0.461** | **15.392** | **0.0001** | **25.929** | **Purifying** | **cox2** |
| **1,310** | **0** | **0** | **0** | **0** | **1** | **0** | **Invariable** | **cox2** |
| **1,311** | **0.635** | **0** | **0.007** | **5.204** | **0.0225** | **0.372** | **Purifying** | **cox2** |
| **1,312** | **1.489** | **0** | **0.29** | **18.775** | **0** | **16.306** | **Purifying** | **cox2** |
| **1,313** | **0** | **0** | **0** | **0** | **1** | **0** | **Invariable** | **cox2** |
| **1,314** | **0** | **0** | **0** | **0** | **1** | **0** | **Invariable** | **cox2** |
| **1,315** | **0.513** | **0** | **0.006** | **5.014** | **0.0251** | **0.345** | **Purifying** | **cox2** |
| **1,316** | **0.83** | **0** | **0.028** | **9.441** | **0.0021** | **1.6** | **Purifying** | **cox2** |
| **1,317** | **1.199** | **0** | **0.019** | **15.307** | **0.0001** | **1.076** | **Purifying** | **cox2** |
| **1,318** | **0** | **0** | **0** | **0** | **1** | **0** | **Invariable** | **cox2** |
| **1,319** | **0** | **0** | **0** | **0** | **1** | **0** | **Invariable** | **cox2** |
| **1,320** | **0** | **0** | **0** | **0** | **1** | **0** | **Invariable** | **cox2** |
| **1,321** | **1.054** | **0** | **0.007** | **8.13** | **0.0044** | **0.384** | **Purifying** | **cox2** |
| **1,322** | **0** | **0** | **0** | **0** | **1** | **0** | **Invariable** | **cox2** |
| **1,323** | **0** | **0** | **0** | **0** | **1** | **0** | **Invariable** | **cox2** |
| **1,324** | **0** | **0** | **0** | **0** | **1** | **0** | **Invariable** | **cox2** |
| **1,325** | **1.387** | **0** | **0.012** | **15.239** | **0.0001** | **0.654** | **Purifying** | **cox2** |
| **1,326** | **1.59** | **0** | **0.017** | **12.552** | **0.0004** | **0.955** | **Purifying** | **cox2** |
| **1,327** | **0** | **0** | **0** | **0** | **1** | **0** | **Invariable** | **cox2** |
| **1,328** | **0** | **0** | **0** | **0** | **1** | **0** | **Invariable** | **cox2** |
| **1,329** | **1.669** | **0** | **0.012** | **8.68** | **0.0032** | **0.662** | **Purifying** | **cox2** |
| **1,330** | **0** | **0** | **0** | **0** | **1** | **0** | **Invariable** | **cox2** |
| **1,331** | **1.555** | **0** | **0.016** | **16.053** | **0.0001** | **0.904** | **Purifying** | **cox2** |
| **1,332** | **0.517** | **0** | **0.006** | **4.868** | **0.0274** | **0.34** | **Purifying** | **cox2** |
| **1,333** | **0.485** | **0** | **0.008** | **5.02** | **0.0251** | **0.422** | **Purifying** | **cox2** |
| **1,334** | **0.722** | **0** | **0.006** | **5.233** | **0.0222** | **0.36** | **Purifying** | **cox2** |
| **1,335** | **0.422** | **0** | **0.007** | **4.602** | **0.0319** | **0.371** | **Purifying** | **cox2** |
| **1,336** | **0.578** | **0** | **0.007** | **3.316** | **0.0686** | **0.42** | **Neutral** | **cox2** |
| **1,337** | **0** | **0** | **0** | **0** | **1** | **0** | **Invariable** | **cox2** |
| **1,338** | **0** | **0** | **0** | **0** | **1** | **0** | **Invariable** | **cox2** |
| **1,339** | **0** | **0** | **0** | **0** | **1** | **0** | **Invariable** | **cox2** |
| **1,340** | **0** | **0** | **0** | **0** | **1** | **0** | **Invariable** | **cox2** |
| **1,341** | **0** | **0** | **0** | **0** | **1** | **0** | **Invariable** | **cox2** |
| **1,342** | **2.59** | **0** | **0.023** | **25.33** | **0** | **1.297** | **Purifying** | **cox2** |
| **1,343** | **0** | **0** | **0** | **0** | **1** | **0** | **Invariable** | **cox2** |
| **1,344** | **0.681** | **0** | **0.005** | **5.681** | **0.0171** | **0.285** | **Purifying** | **cox2** |
| **1,345** | **0.022** | **0** | **0.006** | **2.407** | **0.1208** | **0.315** | **Neutral** | **cox2** |
| **1,346** | **0** | **0** | **0** | **0** | **1** | **0** | **Invariable** | **cox2** |
| **1,347** | **0.379** | **0** | **0.007** | **4.689** | **0.0304** | **0.42** | **Purifying** | **cox2** |
| **1,348** | **1.743** | **0** | **0.013** | **17.61** | **0** | **0.715** | **Purifying** | **cox2** |
| **1,349** | **0.742** | **0** | **0.006** | **5.466** | **0.0194** | **0.325** | **Purifying** | **cox2** |
| **1,350** | **1.197** | **0** | **0.017** | **13.465** | **0.0002** | **0.937** | **Purifying** | **cox2** |
| **1,351** | **0** | **0** | **0** | **0** | **1** | **0** | **Invariable** | **cox2** |
| **1,352** | **0.491** | **0** | **0.007** | **4.466** | **0.0346** | **0.4** | **Purifying** | **cox2** |
| **1,353** | **0** | **0** | **0** | **0** | **1** | **0** | **Invariable** | **cox2** |
| **1,354** | **0** | **0** | **0** | **0** | **1** | **0** | **Invariable** | **cox2** |
| **1,355** | **0** | **0.012** | **0.011** | **0.109** | **0.7415** | **0.611** | **Neutral** | **cox2** |
| **1,356** | **0.023** | **0** | **0.006** | **2.164** | **0.1412** | **0.36** | **Neutral** | **cox2** |
| **1,357** | **1.215** | **0** | **0.151** | **14.699** | **0.0001** | **8.476** | **Purifying** | **cox2** |
| **1,358** | **0** | **0** | **0** | **0** | **1** | **0** | **Invariable** | **cox2** |
| **1,359** | **0** | **0** | **0** | **0** | **1** | **0** | **Invariable** | **cox2** |
| **1,360** | **0** | **0** | **0** | **0** | **1** | **0** | **Invariable** | **cox2** |
| **1,361** | **0** | **0** | **0** | **0** | **1** | **0** | **Invariable** | **cox2** |
| **1,362** | **2.02** | **0** | **0.256** | **16.471** | **0** | **14.382** | **Purifying** | **cox2** |
| **1,363** | **0** | **0** | **0** | **0** | **1** | **0** | **Invariable** | **cox2** |
| **1,364** | **0.421** | **0** | **0.008** | **3.259** | **0.071** | **0.43** | **Neutral** | **cox2** |
| **1,365** | **0** | **0** | **0** | **0** | **1** | **0** | **Invariable** | **cox2** |
| **1,366** | **0** | **0** | **0** | **0** | **1** | **0** | **Invariable** | **cox2** |
| **1,367** | **0** | **0** | **0** | **0** | **1** | **0** | **Invariable** | **cox2** |
| **1,368** | **0** | **0** | **0** | **0** | **1** | **0** | **Invariable** | **cox2** |
| **1,369** | **0** | **0** | **0** | **0** | **1** | **0** | **Invariable** | **cox2** |
| **1,370** | **0.705** | **0** | **0.008** | **4.858** | **0.0275** | **0.423** | **Purifying** | **cox2** |
| **1,371** | **0.443** | **0** | **0.007** | **4.27** | **0.0388** | **0.42** | **Purifying** | **cox2** |
| **1,372** | **0.316** | **0** | **0.009** | **2.294** | **0.1298** | **0.52** | **Neutral** | **cox2** |
| **1,373** | **0** | **0** | **0** | **0** | **1** | **0** | **Invariable** | **cox2** |
| **1,374** | **0.022** | **0** | **0.007** | **2.009** | **0.1564** | **0.393** | **Neutral** | **cox2** |
| **1,375** | **0** | **0** | **0** | **0** | **1** | **0** | **Invariable** | **cox2** |
| **1,376** | **0** | **0** | **0** | **0** | **1** | **0** | **Invariable** | **cox2** |
| **1,377** | **0** | **0** | **0** | **0** | **1** | **0** | **Invariable** | **cox2** |
| **1,378** | **0** | **0** | **0** | **0** | **1** | **0** | **Invariable** | **atp8** |
| **1,379** | **0** | **0** | **0** | **0** | **1** | **0** | **Invariable** | **atp8** |
| **1,380** | **0** | **0** | **0** | **0** | **1** | **0** | **Invariable** | **atp8** |
| **1,381** | **0** | **0** | **0** | **0** | **1** | **0** | **Invariable** | **atp8** |
| **1,382** | **0** | **0** | **0** | **0** | **1** | **0** | **Invariable** | **atp8** |
| **1,383** | **0.515** | **0** | **0.008** | **4.582** | **0.0323** | **0.436** | **Purifying** | **atp8** |
| **1,384** | **0** | **0** | **0** | **0** | **1** | **0** | **Invariable** | **atp8** |
| **1,385** | **0.023** | **0** | **0.006** | **2.163** | **0.1413** | **0.359** | **Neutral** | **atp8** |
| **1,386** | **0** | **0** | **0** | **0** | **1** | **0** | **Invariable** | **atp8** |
| **1,387** | **0.395** | **0** | **0.006** | **4.67** | **0.0307** | **0.344** | **Purifying** | **atp8** |
| **1,388** | **0.454** | **0** | **0.008** | **4.923** | **0.0265** | **0.422** | **Purifying** | **atp8** |
| **1,389** | **0.315** | **0** | **0.009** | **2.283** | **0.1308** | **0.52** | **Neutral** | **atp8** |
| **1,390** | **0** | **0** | **0** | **0** | **1** | **0** | **Invariable** | **atp8** |
| **1,391** | **0.987** | **0** | **0.007** | **7.894** | **0.005** | **0.407** | **Purifying** | **atp8** |
| **1,392** | **0.545** | **0** | **0.006** | **3.032** | **0.0816** | **0.318** | **Neutral** | **atp8** |
| **1,393** | **0** | **0** | **0** | **0** | **1** | **0** | **Invariable** | **atp8** |
| **1,394** | **0.75** | **0** | **0.182** | **13.584** | **0.0002** | **10.232** | **Purifying** | **atp8** |
| **1,395** | **4.199** | **0** | **0.018** | **18.364** | **0** | **1.038** | **Purifying** | **atp8** |
| **1,396** | **1.638** | **0** | **0.68** | **15.224** | **0.0001** | **38.229** | **Purifying** | **atp8** |
| **1,397** | **0** | **0** | **0** | **0** | **1** | **0** | **Invariable** | **atp8** |
| **1,398** | **2.338** | **0.457** | **0.837** | **2.72** | **0.0991** | **47.042** | **Neutral** | **atp8** |
| **1,399** | **2.03** | **0** | **0.895** | **15.989** | **0.0001** | **50.264** | **Purifying** | **atp8** |
| **1,400** | **2.166** | **0** | **0.024** | **16.064** | **0.0001** | **1.342** | **Purifying** | **atp8** |
| **1,401** | **0.329** | **0** | **0.016** | **7.341** | **0.0067** | **0.873** | **Purifying** | **atp8** |
| **1,402** | **0** | **0** | **0** | **0** | **1** | **0** | **Invariable** | **atp8** |
| **1,403** | **0** | **0** | **0** | **0** | **1** | **0** | **Invariable** | **atp8** |
| **1,404** | **0** | **0.005** | **0.005** | **0.019** | **0.8892** | **0.294** | **Neutral** | **atp8** |
| **1,405** | **0.059** | **0** | **0.006** | **3.557** | **0.0593** | **0.339** | **Neutral** | **atp8** |
| **1,406** | **2.164** | **0** | **0.033** | **17.385** | **0** | **1.839** | **Purifying** | **atp8** |
| **1,407** | **0.023** | **0** | **0.006** | **2.396** | **0.1216** | **0.322** | **Neutral** | **atp8** |
| **1,408** | **0** | **0** | **0** | **0** | **1** | **0** | **Invariable** | **atp8** |
| **1,409** | **0.547** | **0** | **0.008** | **4.36** | **0.0368** | **0.459** | **Purifying** | **atp8** |
| **1,410** | **0.943** | **0** | **0.006** | **6.077** | **0.0137** | **0.358** | **Purifying** | **atp8** |
| **1,411** | **5.047** | **0** | **0.041** | **22.141** | **0** | **2.323** | **Purifying** | **atp8** |
| **1,412** | **4.471** | **0** | **0.654** | **24.267** | **0** | **36.724** | **Purifying** | **atp8** |
| **1,413** | **1.236** | **0** | **0.018** | **13.513** | **0.0002** | **0.994** | **Purifying** | **atp8** |
| **1,414** | **1.814** | **0** | **0.442** | **15.054** | **0.0001** | **24.826** | **Purifying** | **atp8** |
| **1,415** | **0** | **0** | **0** | **0** | **1** | **0** | **Invariable** | **atp8** |
| **1,416** | **0** | **0.006** | **0.006** | **0.287** | **0.5921** | **0.311** | **Neutral** | **atp8** |
| **1,417** | **0.474** | **0** | **0.007** | **4.437** | **0.0352** | **0.399** | **Purifying** | **atp8** |
| **1,418** | **1.428** | **0** | **0.008** | **9.37** | **0.0022** | **0.459** | **Purifying** | **atp8** |
| **1,419** | **0** | **0** | **0** | **0** | **1** | **0** | **Invariable** | **atp8** |
| **1,420** | **0** | **0.011** | **0.008** | **0.591** | **0.4419** | **0.459** | **Neutral** | **atp8** |
| **1,421** | **0** | **0** | **0** | **0** | **1** | **0** | **Invariable** | **atp8** |
| **1,422** | **0** | **0** | **0** | **0** | **1** | **0** | **Invariable** | **atp8** |
| **1,423** | **1.32** | **0** | **0.031** | **17.419** | **0** | **1.729** | **Purifying** | **atp8** |
| **1,424** | **0** | **0** | **0** | **0** | **1** | **0** | **Invariable** | **atp8** |
| **1,425** | **1.376** | **0** | **0.018** | **13.796** | **0.0002** | **0.993** | **Purifying** | **atp8** |
| **1,426** | **0** | **0** | **0** | **0** | **1** | **0** | **Invariable** | **atp8** |
| **1,427** | **0** | **0** | **0** | **0** | **1** | **0** | **Invariable** | **atp6** |
| **1,428** | **0.607** | **0.014** | **0.021** | **1.491** | **0.2221** | **1.163** | **Neutral** | **atp6** |
| **1,429** | **0.063** | **0.032** | **0.038** | **0.178** | **0.6733** | **2.161** | **Neutral** | **atp6** |
| **1,430** | **0.043** | **0.037** | **0.039** | **0.008** | **0.9283** | **2.219** | **Neutral** | **atp6** |
| **1,431** | **0** | **0.008** | **0.007** | **0.396** | **0.529** | **0.371** | **Neutral** | **atp6** |
| **1,432** | **0.108** | **0.024** | **0.033** | **0.539** | **0.4627** | **1.865** | **Neutral** | **atp6** |
| **1,433** | **1.079** | **0.011** | **0.036** | **6.296** | **0.0121** | **2.043** | **Purifying** | **atp6** |
| **1,434** | **3.073** | **0.036** | **0.101** | **9.418** | **0.0021** | **5.683** | **Purifying** | **atp6** |
| **1,435** | **0.154** | **0.051** | **0.06** | **0.175** | **0.6758** | **3.391** | **Neutral** | **atp6** |
| **1,436** | **0.469** | **0.01** | **0.048** | **6.205** | **0.0127** | **2.691** | **Purifying** | **atp6** |
| **1,437** | **4502.579** | **0.026** | **0.034** | **0.909** | **0.3403** | **1.927** | **Neutral** | **atp6** |
| **1,438** | **0** | **0.029** | **0.022** | **1.238** | **0.2659** | **1.264** | **Neutral** | **atp6** |
| **1,439** | **0.028** | **0.016** | **0.021** | **0.109** | **0.7411** | **1.168** | **Neutral** | **atp6** |
| **1,440** | **0** | **0.015** | **0.01** | **0.722** | **0.3956** | **0.574** | **Neutral** | **atp6** |
| **1,441** | **2.57** | **0.014** | **0.123** | **11.723** | **0.0006** | **6.886** | **Purifying** | **atp6** |
| **1,442** | **0.04** | **0.056** | **0.048** | **0.054** | **0.8166** | **2.714** | **Neutral** | **atp6** |
| **1,443** | **0.587** | **0.007** | **0.015** | **3.198** | **0.0737** | **0.829** | **Neutral** | **atp6** |
| **1,444** | **3.593** | **0.061** | **0.766** | **9.98** | **0.0016** | **43.041** | **Purifying** | **atp6** |
| **1,445** | **0.031** | **0.047** | **0.041** | **0.079** | **0.779** | **2.327** | **Neutral** | **atp6** |
| **1,446** | **0.023** | **0.049** | **0.038** | **0.271** | **0.6024** | **2.114** | **Neutral** | **atp6** |
| **1,447** | **0** | **0.072** | **0.048** | **1.516** | **0.2182** | **2.681** | **Neutral** | **atp6** |
| **1,448** | **0** | **0.025** | **0.018** | **1.329** | **0.249** | **0.988** | **Neutral** | **atp6** |
| **1,449** | **0.294** | **0.016** | **0.08** | **1.649** | **0.1991** | **4.516** | **Neutral** | **atp6** |
| **1,450** | **0.29** | **0.008** | **0.021** | **2.287** | **0.1304** | **1.173** | **Neutral** | **atp6** |
| **1,451** | **1.735** | **0.018** | **0.347** | **10.565** | **0.0012** | **19.519** | **Purifying** | **atp6** |
| **1,452** | **0.731** | **0.037** | **0.046** | **1.477** | **0.2242** | **2.611** | **Neutral** | **atp6** |
| **1,453** | **5.291** | **0.042** | **1.482** | **17.152** | **0** | **83.281** | **Purifying** | **atp6** |
| **1,454** | **1.686** | **0.023** | **0.169** | **9.132** | **0.0025** | **9.519** | **Purifying** | **atp6** |
| **1,455** | **0.793** | **0.01** | **0.213** | **4.196** | **0.0405** | **11.955** | **Purifying** | **atp6** |
| **1,456** | **1.856** | **0.054** | **0.455** | **8.145** | **0.0043** | **25.559** | **Purifying** | **atp6** |
| **1,457** | **19.023** | **0.022** | **0.438** | **23.552** | **0** | **24.614** | **Purifying** | **atp6** |
| **1,458** | **1.46** | **0.024** | **0.067** | **8.285** | **0.004** | **3.761** | **Purifying** | **atp6** |
| **1,459** | **1.419** | **0** | **0.407** | **19.061** | **0** | **22.846** | **Purifying** | **atp6** |
| **1,460** | **3.219** | **0.017** | **0.446** | **13.871** | **0.0002** | **25.037** | **Purifying** | **atp6** |
| **1,461** | **1.932** | **0.033** | **0.083** | **7.656** | **0.0057** | **4.668** | **Purifying** | **atp6** |
| **1,462** | **0.892** | **0.044** | **0.069** | **2.859** | **0.0908** | **3.858** | **Neutral** | **atp6** |
| **1,463** | **0.176** | **0.013** | **0.037** | **2.772** | **0.0959** | **2.093** | **Neutral** | **atp6** |
| **1,464** | **0.948** | **0** | **0.13** | **10.852** | **0.001** | **7.296** | **Purifying** | **atp6** |
| **1,465** | **1.101** | **0.044** | **0.083** | **3.952** | **0.0468** | **4.644** | **Purifying** | **atp6** |
| **1,466** | **0.038** | **0.04** | **0.04** | **0.002** | **0.9684** | **2.224** | **Neutral** | **atp6** |
| **1,467** | **0.641** | **0.01** | **0.042** | **5.077** | **0.0242** | **2.358** | **Purifying** | **atp6** |
| **1,468** | **0.028** | **0.012** | **0.017** | **0.288** | **0.5913** | **0.937** | **Neutral** | **atp6** |
| **1,469** | **1.418** | **0.036** | **0.435** | **7.085** | **0.0078** | **24.447** | **Purifying** | **atp6** |
| **1,470** | **0.503** | **0.024** | **0.05** | **3.507** | **0.0611** | **2.81** | **Neutral** | **atp6** |
| **1,471** | **0.296** | **0.021** | **0.046** | **1.574** | **0.2097** | **2.565** | **Neutral** | **atp6** |
| **1,472** | **0** | **0.016** | **0.014** | **0.359** | **0.5492** | **0.787** | **Neutral** | **atp6** |
| **1,473** | **0.564** | **0.034** | **0.044** | **2.224** | **0.1358** | **2.449** | **Neutral** | **atp6** |
| **1,474** | **2.21** | **0.011** | **0.043** | **12.243** | **0.0005** | **2.442** | **Purifying** | **atp6** |
| **1,475** | **1.821** | **0.018** | **0.362** | **10.798** | **0.001** | **20.351** | **Purifying** | **atp6** |
| **1,476** | **0.086** | **0.046** | **0.051** | **0.063** | **0.8024** | **2.871** | **Neutral** | **atp6** |
| **1,477** | **1.241** | **0.028** | **0.069** | **6.252** | **0.0124** | **3.876** | **Purifying** | **atp6** |
| **1,478** | **1.14** | **0.009** | **0.052** | **11.083** | **0.0009** | **2.944** | **Purifying** | **atp6** |
| **1,479** | **0** | **0.031** | **0.022** | **1.223** | **0.2688** | **1.26** | **Neutral** | **atp6** |
| **1,480** | **8.951** | **0.036** | **0.069** | **5.113** | **0.0237** | **3.895** | **Purifying** | **atp6** |
| **1,481** | **60.054** | **0.019** | **0.068** | **13.678** | **0.0002** | **3.845** | **Purifying** | **atp6** |
| **1,482** | **0.695** | **0.012** | **0.055** | **6.351** | **0.0117** | **3.083** | **Purifying** | **atp6** |
| **1,483** | **1.477** | **0** | **0.429** | **14.314** | **0.0002** | **24.104** | **Purifying** | **atp6** |
| **1,484** | **0.52** | **0.023** | **0.034** | **1.528** | **0.2163** | **1.925** | **Neutral** | **atp6** |
| **1,485** | **2.596** | **0.017** | **0.453** | **13.205** | **0.0003** | **25.445** | **Purifying** | **atp6** |
| **1,486** | **0.315** | **0.449** | **0.392** | **0.069** | **0.7934** | **22.052** | **Neutral** | **atp6** |
| **1,487** | **1.915** | **0.03** | **0.454** | **5.4** | **0.0201** | **25.532** | **Purifying** | **atp6** |
| **1,488** | **1.62** | **0.033** | **0.384** | **9.61** | **0.0019** | **21.559** | **Purifying** | **atp6** |
| **1,489** | **1.556** | **0.023** | **0.238** | **12.25** | **0.0005** | **13.365** | **Purifying** | **atp6** |
| **1,490** | **1.503** | **0** | **0.012** | **16.688** | **0** | **0.662** | **Purifying** | **atp6** |
| **1,491** | **1.176** | **0.017** | **0.02** | **2.449** | **0.1176** | **1.12** | **Neutral** | **atp6** |
| **1,492** | **0.167** | **0.043** | **0.053** | **0.204** | **0.6512** | **2.95** | **Neutral** | **atp6** |
| **1,493** | **0** | **0.031** | **0.019** | **1.486** | **0.2228** | **1.051** | **Neutral** | **atp6** |
| **1,494** | **1.28** | **0.036** | **0.32** | **7.052** | **0.0079** | **17.999** | **Purifying** | **atp6** |
| **1,495** | **2.902** | **0.038** | **0.686** | **10.103** | **0.0015** | **38.547** | **Purifying** | **atp6** |
| **1,496** | **0.319** | **0.465** | **0.419** | **0.066** | **0.7973** | **23.533** | **Neutral** | **atp6** |
| **1,497** | **0.716** | **0.008** | **0.031** | **5.958** | **0.0147** | **1.75** | **Purifying** | **atp6** |
| **1,498** | **3.314** | **0.01** | **0.363** | **16.943** | **0** | **20.399** | **Purifying** | **atp6** |
| **1,499** | **1.494** | **0.039** | **0.568** | **7.376** | **0.0066** | **31.921** | **Purifying** | **atp6** |
| **1,500** | **0** | **0.379** | **0.381** | **1.397** | **0.2372** | **21.395** | **Neutral** | **atp6** |
| **1,501** | **3.127** | **0.02** | **0.777** | **14.3** | **0.0002** | **43.63** | **Purifying** | **atp6** |
| **1,502** | **0.039** | **0.01** | **0.016** | **0.628** | **0.428** | **0.926** | **Neutral** | **atp6** |
| **1,503** | **1.41** | **0.019** | **0.423** | **9.537** | **0.002** | **23.745** | **Purifying** | **atp6** |
| **1,504** | **0.339** | **0.019** | **0.047** | **2.581** | **0.1081** | **2.616** | **Neutral** | **atp6** |
| **1,505** | **0.069** | **0.018** | **0.028** | **0.295** | **0.5869** | **1.593** | **Neutral** | **atp6** |
| **1,506** | **0.836** | **0.008** | **0.041** | **9.552** | **0.002** | **2.322** | **Purifying** | **atp6** |
| **1,507** | **3.231** | **0.018** | **0.084** | **14.777** | **0.0001** | **4.728** | **Purifying** | **atp6** |
| **1,508** | **0.284** | **0.025** | **0.041** | **1.05** | **0.3056** | **2.279** | **Neutral** | **atp6** |
| **1,509** | **4.034** | **0.033** | **0.789** | **11.622** | **0.0007** | **44.351** | **Purifying** | **atp6** |
| **1,510** | **0.499** | **0.008** | **0.041** | **7.526** | **0.0061** | **2.316** | **Purifying** | **atp6** |
| **1,511** | **1.548** | **0.013** | **0.425** | **10.823** | **0.001** | **23.881** | **Purifying** | **atp6** |
| **1,512** | **0.313** | **0** | **0.024** | **5.788** | **0.0161** | **1.333** | **Purifying** | **atp6** |
| **1,513** | **0.533** | **0.012** | **0.039** | **4.346** | **0.0371** | **2.176** | **Purifying** | **atp6** |
| **1,514** | **5.957** | **0.037** | **0.392** | **15.702** | **0.0001** | **22.048** | **Purifying** | **atp6** |
| **1,515** | **0.534** | **0** | **0.021** | **9.286** | **0.0023** | **1.168** | **Purifying** | **atp6** |
| **1,516** | **13.284** | **0.011** | **0.606** | **25.046** | **0** | **34.041** | **Purifying** | **atp6** |
| **1,517** | **0.568** | **0.01** | **0.017** | **2.45** | **0.1176** | **0.939** | **Neutral** | **atp6** |
| **1,518** | **2.055** | **0.033** | **0.153** | **8.427** | **0.0037** | **8.622** | **Purifying** | **atp6** |
| **1,519** | **1.287** | **0.024** | **0.303** | **10.148** | **0.0014** | **17.041** | **Purifying** | **atp6** |
| **1,520** | **0.698** | **0.009** | **0.025** | **3.301** | **0.0692** | **1.382** | **Neutral** | **atp6** |
| **1,521** | **0** | **0.012** | **0.01** | **0.471** | **0.4925** | **0.554** | **Neutral** | **atp6** |
| **1,522** | **1.848** | **0.054** | **0.501** | **8.538** | **0.0035** | **28.137** | **Purifying** | **atp6** |
| **1,523** | **1.085** | **0.033** | **0.089** | **6.785** | **0.0092** | **5.022** | **Purifying** | **atp6** |
| **1,524** | **1.989** | **0.013** | **0.589** | **12.686** | **0.0004** | **33.109** | **Purifying** | **atp6** |
| **1,525** | **0.986** | **0.014** | **0.064** | **5.902** | **0.0151** | **3.584** | **Purifying** | **atp6** |
| **1,526** | **0** | **0.241** | **0.268** | **1.632** | **0.2014** | **15.083** | **Neutral** | **atp6** |
| **1,527** | **0.639** | **0.009** | **0.038** | **4.729** | **0.0297** | **2.134** | **Purifying** | **atp6** |
| **1,528** | **0.769** | **0** | **0.083** | **10.935** | **0.0009** | **4.639** | **Purifying** | **atp6** |
| **1,529** | **0.811** | **0.01** | **0.087** | **8.162** | **0.0043** | **4.907** | **Purifying** | **atp6** |
| **1,530** | **2.081** | **0.037** | **0.424** | **11.24** | **0.0008** | **23.85** | **Purifying** | **atp6** |
| **1,531** | **1.852** | **0.012** | **0.401** | **13.004** | **0.0003** | **22.54** | **Purifying** | **atp6** |
| **1,532** | **1.362** | **0** | **0.712** | **14.003** | **0.0002** | **39.981** | **Purifying** | **atp6** |
| **1,533** | **2.043** | **0.013** | **0.672** | **11.352** | **0.0008** | **37.733** | **Purifying** | **atp6** |
| **1,534** | **2.023** | **0.013** | **0.335** | **13.576** | **0.0002** | **18.823** | **Purifying** | **atp6** |
| **1,535** | **0.734** | **0.024** | **0.054** | **5.48** | **0.0192** | **3.041** | **Purifying** | **atp6** |
| **1,536** | **2.565** | **0.019** | **0.459** | **14.199** | **0.0002** | **25.789** | **Purifying** | **atp6** |
| **1,537** | **0.969** | **0.033** | **0.15** | **6.237** | **0.0125** | **8.402** | **Purifying** | **atp6** |
| **1,538** | **0.677** | **0.01** | **0.027** | **4.262** | **0.039** | **1.535** | **Purifying** | **atp6** |
| **1,539** | **1.361** | **0.025** | **0.314** | **12.298** | **0.0005** | **17.627** | **Purifying** | **atp6** |
| **1,540** | **0.011** | **0.141** | **0.066** | **0.061** | **0.8046** | **3.702** | **Neutral** | **atp6** |
| **1,541** | **0.055** | **0.035** | **0.041** | **0.052** | **0.8196** | **2.298** | **Neutral** | **atp6** |
| **1,542** | **0.889** | **0.043** | **0.064** | **2.988** | **0.0839** | **3.577** | **Neutral** | **atp6** |
| **1,543** | **10.224** | **0.041** | **0.886** | **20.586** | **0** | **49.756** | **Purifying** | **atp6** |
| **1,544** | **1.827** | **0.012** | **0.415** | **13.254** | **0.0003** | **23.344** | **Purifying** | **atp6** |
| **1,545** | **0.443** | **0.01** | **0.043** | **5.398** | **0.0202** | **2.413** | **Purifying** | **atp6** |
| **1,546** | **1.128** | **0.314** | **0.529** | **1.306** | **0.2531** | **29.728** | **Neutral** | **atp6** |
| **1,547** | **2.655** | **0.019** | **0.464** | **16.716** | **0** | **26.091** | **Purifying** | **atp6** |
| **1,548** | **0.94** | **0.05** | **0.127** | **4.003** | **0.0454** | **7.134** | **Purifying** | **atp6** |
| **1,549** | **2.209** | **0.02** | **0.074** | **10.737** | **0.0011** | **4.144** | **Purifying** | **atp6** |
| **1,550** | **1.141** | **0.041** | **0.058** | **3.194** | **0.0739** | **3.28** | **Neutral** | **atp6** |
| **1,551** | **2.486** | **0.043** | **0.922** | **10.52** | **0.0012** | **51.802** | **Purifying** | **atp6** |
| **1,552** | **4.045** | **0** | **0.508** | **17.775** | **0** | **28.557** | **Purifying** | **atp6** |
| **1,553** | **0.715** | **0.023** | **0.054** | **5.719** | **0.0168** | **3.057** | **Purifying** | **atp6** |
| **1,554** | **0.161** | **0.028** | **0.036** | **0.395** | **0.5296** | **2.024** | **Neutral** | **atp6** |
| **1,555** | **2.907** | **0.007** | **0.276** | **20.132** | **0** | **15.528** | **Purifying** | **atp6** |
| **1,556** | **1.444** | **0.015** | **0.084** | **8.564** | **0.0034** | **4.71** | **Purifying** | **atp6** |
| **1,557** | **4.381** | **0.012** | **0.605** | **18.187** | **0** | **33.987** | **Purifying** | **atp6** |
| **1,558** | **0.735** | **0.008** | **0.033** | **5.827** | **0.0158** | **1.88** | **Purifying** | **atp6** |
| **1,559** | **2.019** | **0.012** | **0.052** | **10.466** | **0.0012** | **2.9** | **Purifying** | **atp6** |
| **1,560** | **1.268** | **0.037** | **0.261** | **5.591** | **0.0181** | **14.655** | **Purifying** | **atp6** |
| **1,561** | **1.473** | **0.009** | **0.343** | **12.628** | **0.0004** | **19.279** | **Purifying** | **atp6** |
| **1,562** | **1.937** | **0.015** | **0.456** | **12.293** | **0.0005** | **25.592** | **Purifying** | **atp6** |
| **1,563** | **0.067** | **0.013** | **0.026** | **0.872** | **0.3503** | **1.454** | **Neutral** | **atp6** |
| **1,564** | **0.042** | **0.045** | **0.044** | **0.002** | **0.9663** | **2.457** | **Neutral** | **atp6** |
| **1,565** | **0.318** | **0.02** | **0.033** | **1.661** | **0.1975** | **1.873** | **Neutral** | **atp6** |
| **1,566** | **1.329** | **0.013** | **0.086** | **8.668** | **0.0032** | **4.85** | **Purifying** | **atp6** |
| **1,567** | **0.636** | **0.019** | **0.068** | **3.584** | **0.0583** | **3.811** | **Neutral** | **atp6** |
| **1,568** | **100.328** | **0.027** | **0.622** | **27.795** | **0** | **34.946** | **Purifying** | **atp6** |
| **1,569** | **0** | **0.008** | **0.007** | **0.465** | **0.4953** | **0.373** | **Neutral** | **atp6** |
| **1,570** | **2.276** | **0.022** | **0.38** | **12.16** | **0.0005** | **21.357** | **Purifying** | **atp6** |
| **1,571** | **0** | **0.022** | **0.018** | **0.849** | **0.3567** | **1.007** | **Neutral** | **atp6** |
| **1,572** | **1.097** | **0** | **0.457** | **12.579** | **0.0004** | **25.679** | **Purifying** | **atp6** |
| **1,573** | **4.902** | **0.011** | **0.491** | **19.939** | **0** | **27.613** | **Purifying** | **atp6** |
| **1,574** | **0.029** | **0** | **0.006** | **2.548** | **0.1104** | **0.348** | **Neutral** | **atp6** |
| **1,575** | **0.766** | **0.011** | **0.07** | **10.718** | **0.0011** | **3.906** | **Purifying** | **atp6** |
| **1,576** | **1.837** | **0.012** | **0.392** | **12.879** | **0.0003** | **22.052** | **Purifying** | **atp6** |
| **1,577** | **2.535** | **0.023** | **0.839** | **12.164** | **0.0005** | **47.164** | **Purifying** | **atp6** |
| **1,578** | **0.586** | **0** | **0.019** | **9.902** | **0.0017** | **1.047** | **Purifying** | **atp6** |
| **1,579** | **2.079** | **0.038** | **0.507** | **8.6** | **0.0034** | **28.495** | **Purifying** | **atp6** |
| **1,580** | **1.269** | **0.009** | **0.042** | **9.891** | **0.0017** | **2.364** | **Purifying** | **atp6** |
| **1,581** | **2.051** | **0.009** | **0.167** | **11.323** | **0.0008** | **9.405** | **Purifying** | **atp6** |
| **1,582** | **0.883** | **0.025** | **0.089** | **6.807** | **0.0091** | **4.99** | **Purifying** | **atp6** |
| **1,583** | **2.802** | **0.016** | **0.682** | **11.599** | **0.0007** | **38.312** | **Purifying** | **atp6** |
| **1,584** | **0.815** | **0.031** | **0.075** | **5.674** | **0.0172** | **4.189** | **Purifying** | **atp6** |
| **1,585** | **1.102** | **0.025** | **0.168** | **8.18** | **0.0042** | **9.434** | **Purifying** | **atp6** |
| **1,586** | **1.075** | **0.044** | **0.075** | **3.571** | **0.0588** | **4.223** | **Neutral** | **atp6** |
| **1,587** | **0.726** | **0** | **0.093** | **10.251** | **0.0014** | **5.206** | **Purifying** | **atp6** |
| **1,588** | **0.515** | **0.009** | **0.025** | **2.981** | **0.0842** | **1.378** | **Neutral** | **atp6** |
| **1,589** | **0.715** | **0.011** | **0.044** | **4.595** | **0.0321** | **2.449** | **Purifying** | **atp6** |
| **1,590** | **2.593** | **0.02** | **0.316** | **16.5** | **0** | **17.777** | **Purifying** | **atp6** |
| **1,591** | **0.122** | **0.038** | **0.047** | **0.406** | **0.524** | **2.651** | **Neutral** | **atp6** |
| **1,592** | **2.103** | **0.033** | **0.697** | **9.4** | **0.0022** | **39.138** | **Purifying** | **atp6** |
| **1,593** | **0.351** | **0.025** | **0.077** | **1.645** | **0.1997** | **4.304** | **Neutral** | **atp6** |
| **1,594** | **0.078** | **0.018** | **0.039** | **0.899** | **0.343** | **2.204** | **Neutral** | **atp6** |
| **1,595** | **0** | **0.048** | **0.039** | **1.096** | **0.2951** | **2.209** | **Neutral** | **atp6** |
| **1,596** | **1.495** | **0.039** | **0.484** | **6.956** | **0.0084** | **27.181** | **Purifying** | **atp6** |
| **1,597** | **0.077** | **0.01** | **0.026** | **1.999** | **0.1574** | **1.466** | **Neutral** | **atp6** |
| **1,598** | **0.072** | **0.025** | **0.035** | **0.456** | **0.4997** | **1.942** | **Neutral** | **atp6** |
| **1,599** | **0.557** | **0.031** | **0.059** | **2.773** | **0.0959** | **3.33** | **Neutral** | **atp6** |
| **1,600** | **0.967** | **0.022** | **0.057** | **7.122** | **0.0076** | **3.223** | **Purifying** | **atp6** |
| **1,601** | **0.999** | **0.025** | **0.064** | **6.957** | **0.0083** | **3.621** | **Purifying** | **atp6** |
| **1,602** | **1.138** | **0.01** | **0.071** | **8.22** | **0.0041** | **3.981** | **Purifying** | **atp6** |
| **1,603** | **1.518** | **0.019** | **0.048** | **8.744** | **0.0031** | **2.687** | **Purifying** | **atp6** |
| **1,604** | **1.316** | **0** | **0.049** | **12.506** | **0.0004** | **2.761** | **Purifying** | **atp6** |
| **1,605** | **2.159** | **0** | **0.58** | **16.641** | **0** | **32.573** | **Purifying** | **atp6** |
| **1,606** | **1.286** | **0.042** | **0.255** | **6.7** | **0.0096** | **14.34** | **Purifying** | **atp6** |
| **1,607** | **3.126** | **0.016** | **0.725** | **12.389** | **0.0004** | **40.74** | **Purifying** | **atp6** |
| **1,608** | **0.607** | **0.014** | **0.021** | **1.491** | **0.2221** | **1.163** | **Neutral** | **atp6** |
| **1,609** | **0.45** | **0.028** | **0.052** | **1.983** | **0.1591** | **2.9** | **Neutral** | **atp6** |
| **1,610** | **0.061** | **0.008** | **0.016** | **1.426** | **0.2325** | **0.921** | **Neutral** | **atp6** |
| **1,611** | **0.392** | **0.009** | **0.036** | **4.803** | **0.0284** | **2.047** | **Purifying** | **atp6** |
| **1,612** | **0.614** | **0.019** | **0.041** | **3.79** | **0.0516** | **2.323** | **Neutral** | **atp6** |
| **1,613** | **1.164** | **0.042** | **0.169** | **5.997** | **0.0143** | **9.477** | **Purifying** | **atp6** |
| **1,614** | **0.955** | **0.032** | **0.115** | **5.67** | **0.0173** | **6.473** | **Purifying** | **atp6** |
| **1,615** | **0.065** | **0.049** | **0.053** | **0.019** | **0.8889** | **2.954** | **Neutral** | **atp6** |
| **1,616** | **0.019** | **0.038** | **0.032** | **0.168** | **0.6819** | **1.796** | **Neutral** | **atp6** |
| **1,617** | **0.536** | **0.029** | **0.045** | **1.931** | **0.1647** | **2.532** | **Neutral** | **atp6** |
| **1,618** | **2.581** | **0.016** | **0.468** | **9.528** | **0.002** | **26.29** | **Purifying** | **atp6** |
| **1,619** | **0.834** | **0** | **0.122** | **10.568** | **0.0012** | **6.86** | **Purifying** | **atp6** |
| **1,620** | **0.875** | **0.01** | **0.03** | **5.285** | **0.0215** | **1.693** | **Purifying** | **atp6** |
| **1,621** | **2.05** | **0** | **0.455** | **15.563** | **0.0001** | **25.571** | **Purifying** | **atp6** |
| **1,622** | **0.425** | **0.02** | **0.056** | **2.507** | **0.1133** | **3.124** | **Neutral** | **atp6** |
| **1,623** | **0.538** | **0.031** | **0.062** | **3.036** | **0.0814** | **3.488** | **Neutral** | **atp6** |
| **1,624** | **1.252** | **0.037** | **0.521** | **7.732** | **0.0054** | **29.268** | **Purifying** | **atp6** |
| **1,625** | **2.281** | **0** | **0.689** | **15.065** | **0.0001** | **38.701** | **Purifying** | **atp6** |
| **1,626** | **0** | **0.011** | **0.01** | **0.279** | **0.5975** | **0.544** | **Neutral** | **atp6** |
| **1,627** | **1.38** | **0.012** | **0.073** | **8.224** | **0.0041** | **4.098** | **Purifying** | **atp6** |
| **1,628** | **2.3** | **0.026** | **0.327** | **12.479** | **0.0004** | **18.383** | **Purifying** | **atp6** |
| **1,629** | **0.069** | **0** | **0.008** | **4.383** | **0.0363** | **0.449** | **Purifying** | **atp6** |
| **1,630** | **1.447** | **0.019** | **0.137** | **9.796** | **0.0017** | **7.714** | **Purifying** | **atp6** |
| **1,631** | **0.023** | **0.26** | **0.239** | **0.37** | **0.5433** | **13.415** | **Neutral** | **atp6** |
| **1,632** | **0.304** | **0.044** | **0.055** | **0.484** | **0.4865** | **3.101** | **Neutral** | **atp6** |
| **1,633** | **5.531** | **0** | **0.531** | **25.491** | **0** | **29.833** | **Purifying** | **atp6** |
| **1,634** | **1.286** | **0** | **0.094** | **16.106** | **0.0001** | **5.257** | **Purifying** | **atp6** |
| **1,635** | **0** | **0.01** | **0.009** | **0.435** | **0.5097** | **0.478** | **Neutral** | **atp6** |
| **1,636** | **0.683** | **0.009** | **0.026** | **3.752** | **0.0528** | **1.454** | **Neutral** | **atp6** |
| **1,637** | **42.405** | **0.011** | **0.386** | **20.539** | **0** | **21.703** | **Purifying** | **atp6** |
| **1,638** | **1.425** | **0.037** | **0.386** | **9.323** | **0.0023** | **21.664** | **Purifying** | **atp6** |
| **1,639** | **1.223** | **0.023** | **0.215** | **6.613** | **0.0101** | **12.081** | **Purifying** | **atp6** |
| **1,640** | **0.989** | **0.016** | **0.084** | **6.076** | **0.0137** | **4.704** | **Purifying** | **atp6** |
| **1,641** | **0.891** | **0.05** | **0.287** | **4.757** | **0.0292** | **16.131** | **Purifying** | **atp6** |
| **1,642** | **1.354** | **0.01** | **0.149** | **11.775** | **0.0006** | **8.391** | **Purifying** | **atp6** |
| **1,643** | **0.425** | **0.042** | **0.052** | **1.039** | **0.308** | **2.905** | **Neutral** | **atp6** |
| **1,644** | **0.038** | **0.031** | **0.033** | **0.014** | **0.9072** | **1.854** | **Neutral** | **atp6** |
| **1,645** | **0.017** | **0.014** | **0.016** | **0.011** | **0.9148** | **0.872** | **Neutral** | **atp6** |
| **1,646** | **0.149** | **0.023** | **0.034** | **0.623** | **0.4298** | **1.922** | **Neutral** | **atp6** |
| **1,647** | **0** | **0.026** | **0.022** | **0.766** | **0.3816** | **1.211** | **Neutral** | **atp6** |
| **1,648** | **0.124** | **0.021** | **0.027** | **0.28** | **0.5964** | **1.508** | **Neutral** | **atp6** |
| **1,649** | **0.064** | **0.009** | **0.018** | **1.461** | **0.2267** | **1.021** | **Neutral** | **atp6** |
| **1,650** | **1403.211** | **0.007** | **0.012** | **1.738** | **0.1874** | **0.698** | **Neutral** | **cox3** |
| **1,651** | **0.028** | **0** | **0.006** | **2.971** | **0.0848** | **0.324** | **Neutral** | **cox3** |
| **1,652** | **0** | **0.04** | **0.03** | **1.439** | **0.2303** | **1.678** | **Neutral** | **cox3** |
| **1,653** | **0** | **0.02** | **0.014** | **0.832** | **0.3617** | **0.774** | **Neutral** | **cox3** |
| **1,654** | **0.072** | **0.025** | **0.035** | **0.456** | **0.4997** | **1.942** | **Neutral** | **cox3** |
| **1,655** | **0.016** | **0.053** | **0.049** | **0.069** | **0.7922** | **2.758** | **Neutral** | **cox3** |
| **1,656** | **0.069** | **0.008** | **0.016** | **1.729** | **0.1886** | **0.918** | **Neutral** | **cox3** |
| **1,657** | **10000** | **0.014** | **0.397** | **18.501** | **0** | **22.315** | **Purifying** | **cox3** |
| **1,658** | **1.154** | **0.024** | **0.044** | **4.205** | **0.0403** | **2.487** | **Purifying** | **cox3** |
| **1,659** | **0.092** | **0.017** | **0.034** | **0.896** | **0.3438** | **1.933** | **Neutral** | **cox3** |
| **1,660** | **0.546** | **0.009** | **0.041** | **5.191** | **0.0227** | **2.289** | **Purifying** | **cox3** |
| **1,661** | **0.039** | **0.023** | **0.027** | **0.108** | **0.7429** | **1.502** | **Neutral** | **cox3** |
| **1,662** | **0.758** | **0.023** | **0.053** | **3.579** | **0.0585** | **2.968** | **Neutral** | **cox3** |
| **1,663** | **0.044** | **0.022** | **0.026** | **0.198** | **0.6561** | **1.436** | **Neutral** | **cox3** |
| **1,664** | **0** | **0.023** | **0.014** | **1.843** | **0.1746** | **0.771** | **Neutral** | **cox3** |
| **1,665** | **0** | **0.066** | **0.036** | **1.9** | **0.1681** | **2** | **Neutral** | **cox3** |
| **1,666** | **0.995** | **0.019** | **0.081** | **7.028** | **0.008** | **4.564** | **Purifying** | **cox3** |
| **1,667** | **0** | **0** | **0** | **0** | **1** | **0** | **Invariable** | **cox3** |
| **1,668** | **0.569** | **0.021** | **0.042** | **4.941** | **0.0262** | **2.367** | **Purifying** | **cox3** |
| **1,669** | **0** | **0.025** | **0.018** | **1.056** | **0.3041** | **1.039** | **Neutral** | **cox3** |
| **1,670** | **0.017** | **0.012** | **0.013** | **0.026** | **0.8721** | **0.747** | **Neutral** | **cox3** |
| **1,671** | **0.022** | **0.01** | **0.014** | **0.25** | **0.6173** | **0.814** | **Neutral** | **cox3** |
| **1,672** | **0.028** | **0.025** | **0.026** | **0.007** | **0.9345** | **1.47** | **Neutral** | **cox3** |
| **1,673** | **0** | **0.039** | **0.023** | **1.788** | **0.1811** | **1.28** | **Neutral** | **cox3** |
| **1,674** | **2.025** | **0.038** | **0.531** | **8.286** | **0.004** | **29.827** | **Purifying** | **cox3** |
| **1,675** | **0.151** | **0.038** | **0.049** | **0.246** | **0.6201** | **2.737** | **Neutral** | **cox3** |
| **1,676** | **0.737** | **0.031** | **0.05** | **2.594** | **0.1073** | **2.824** | **Neutral** | **cox3** |
| **1,677** | **0.729** | **0.016** | **0.047** | **4.185** | **0.0408** | **2.663** | **Purifying** | **cox3** |
| **1,678** | **0.023** | **0.023** | **0.023** | **0** | **0.9971** | **1.297** | **Neutral** | **cox3** |
| **1,679** | **0.055** | **0** | **0.011** | **3.094** | **0.0786** | **0.592** | **Neutral** | **cox3** |
| **1,680** | **0** | **0.008** | **0.006** | **0.449** | **0.5028** | **0.36** | **Neutral** | **cox3** |
| **1,681** | **0.044** | **0** | **0.008** | **3.251** | **0.0714** | **0.448** | **Neutral** | **cox3** |
| **1,682** | **0** | **0.011** | **0.009** | **0.469** | **0.4933** | **0.509** | **Neutral** | **cox3** |
| **1,683** | **0.944** | **0.012** | **0.022** | **4.612** | **0.0317** | **1.263** | **Purifying** | **cox3** |
| **1,684** | **0.538** | **0.024** | **0.079** | **4.927** | **0.0264** | **4.417** | **Purifying** | **cox3** |
| **1,685** | **2.016** | **0.008** | **0.03** | **13.297** | **0.0003** | **1.707** | **Purifying** | **cox3** |
| **1,686** | **0.782** | **0.029** | **0.047** | **5.374** | **0.0204** | **2.616** | **Purifying** | **cox3** |
| **1,687** | **0.676** | **0.019** | **0.046** | **3.964** | **0.0465** | **2.587** | **Purifying** | **cox3** |
| **1,688** | **0** | **0.04** | **0.027** | **1.235** | **0.2664** | **1.499** | **Neutral** | **cox3** |
| **1,689** | **2.154** | **0.05** | **0.72** | **8.887** | **0.0029** | **40.445** | **Purifying** | **cox3** |
| **1,690** | **0.153** | **0.028** | **0.035** | **0.422** | **0.5158** | **1.959** | **Neutral** | **cox3** |
| **1,691** | **4.157** | **0.036** | **0.584** | **14.338** | **0.0002** | **32.813** | **Purifying** | **cox3** |
| **1,692** | **0.096** | **0.011** | **0.03** | **1.64** | **0.2003** | **1.678** | **Neutral** | **cox3** |
| **1,693** | **0** | **0.02** | **0.016** | **0.736** | **0.3908** | **0.927** | **Neutral** | **cox3** |
| **1,694** | **0** | **0.023** | **0.018** | **1.03** | **0.3102** | **0.994** | **Neutral** | **cox3** |
| **1,695** | **0** | **0.035** | **0.024** | **1.52** | **0.2177** | **1.371** | **Neutral** | **cox3** |
| **1,696** | **0.024** | **0.016** | **0.02** | **0.054** | **0.8162** | **1.102** | **Neutral** | **cox3** |
| **1,697** | **0.035** | **0.039** | **0.037** | **0.003** | **0.9585** | **2.104** | **Neutral** | **cox3** |
| **1,698** | **2.464** | **0.013** | **0.656** | **12.609** | **0.0004** | **36.872** | **Purifying** | **cox3** |
| **1,699** | **1.486** | **0.014** | **0.491** | **9.437** | **0.0021** | **27.57** | **Purifying** | **cox3** |
| **1,700** | **1.89** | **0.02** | **0.046** | **8.946** | **0.0028** | **2.611** | **Purifying** | **cox3** |
| **1,701** | **0** | **0.076** | **0.056** | **1.418** | **0.2337** | **3.143** | **Neutral** | **cox3** |
| **1,702** | **10000** | **0.032** | **0.626** | **23.778** | **0** | **35.192** | **Purifying** | **cox3** |
| **1,703** | **0** | **0.026** | **0.022** | **0.679** | **0.4099** | **1.226** | **Neutral** | **cox3** |
| **1,704** | **0.051** | **0.013** | **0.022** | **0.722** | **0.3953** | **1.237** | **Neutral** | **cox3** |
| **1,705** | **0.141** | **0.024** | **0.032** | **0.437** | **0.5087** | **1.825** | **Neutral** | **cox3** |
| **1,706** | **1.733** | **0.041** | **0.162** | **7.065** | **0.0079** | **9.081** | **Purifying** | **cox3** |
| **1,707** | **0.328** | **0.009** | **0.045** | **4.253** | **0.0392** | **2.546** | **Purifying** | **cox3** |
| **1,708** | **0** | **0.037** | **0.024** | **1.086** | **0.2973** | **1.358** | **Neutral** | **cox3** |
| **1,709** | **0.232** | **0.027** | **0.051** | **1.798** | **0.1799** | **2.843** | **Neutral** | **cox3** |
| **1,710** | **0** | **0.023** | **0.017** | **1.225** | **0.2683** | **0.937** | **Neutral** | **cox3** |
| **1,711** | **0.066** | **0.032** | **0.038** | **0.159** | **0.6899** | **2.127** | **Neutral** | **cox3** |
| **1,712** | **0** | **0.015** | **0.011** | **0.648** | **0.4207** | **0.592** | **Neutral** | **cox3** |
| **1,713** | **0.13** | **0.053** | **0.06** | **0.105** | **0.7456** | **3.388** | **Neutral** | **cox3** |
| **1,714** | **2.35** | **0.025** | **0.316** | **15.433** | **0.0001** | **17.739** | **Purifying** | **cox3** |
| **1,715** | **0.002** | **0.027** | **0.026** | **0.062** | **0.8036** | **1.446** | **Neutral** | **cox3** |
| **1,716** | **3.083** | **0.038** | **0.105** | **9.318** | **0.0023** | **5.902** | **Purifying** | **cox3** |
| **1,717** | **1.369** | **0.029** | **0.125** | **7.746** | **0.0054** | **7.023** | **Purifying** | **cox3** |
| **1,718** | **0.631** | **0.023** | **0.03** | **2.013** | **0.156** | **1.698** | **Neutral** | **cox3** |
| **1,719** | **1.219** | **0.023** | **0.35** | **10.016** | **0.0016** | **19.682** | **Purifying** | **cox3** |
| **1,720** | **0.14** | **0.012** | **0.024** | **1.276** | **0.2587** | **1.324** | **Neutral** | **cox3** |
| **1,721** | **0.04** | **0.025** | **0.028** | **0.071** | **0.7906** | **1.593** | **Neutral** | **cox3** |
| **1,722** | **10.474** | **0.041** | **0.084** | **6.306** | **0.012** | **4.72** | **Purifying** | **cox3** |
| **1,723** | **0.793** | **0.022** | **0.054** | **3.855** | **0.0496** | **3.03** | **Purifying** | **cox3** |
| **1,724** | **0.06** | **0.031** | **0.036** | **0.131** | **0.7172** | **2.033** | **Neutral** | **cox3** |
| **1,725** | **0.058** | **0.028** | **0.035** | **0.197** | **0.6576** | **1.99** | **Neutral** | **cox3** |
| **1,726** | **1.413** | **0** | **0.02** | **15.474** | **0.0001** | **1.107** | **Purifying** | **cox3** |
| **1,727** | **2.873** | **0.02** | **0.424** | **18.95** | **0** | **23.835** | **Purifying** | **cox3** |
| **1,728** | **0** | **0.053** | **0.039** | **1.484** | **0.2231** | **2.213** | **Neutral** | **cox3** |
| **1,729** | **1.704** | **0.009** | **0.052** | **10.094** | **0.0015** | **2.926** | **Purifying** | **cox3** |
| **1,730** | **2.284** | **0** | **0.636** | **17.194** | **0** | **35.708** | **Purifying** | **cox3** |
| **1,731** | **1.665** | **0.022** | **0.065** | **9.654** | **0.0019** | **3.659** | **Purifying** | **cox3** |
| **1,732** | **0.955** | **0.033** | **0.102** | **6** | **0.0143** | **5.709** | **Purifying** | **cox3** |
| **1,733** | **1.178** | **0** | **0.014** | **12.121** | **0.0005** | **0.772** | **Purifying** | **cox3** |
| **1,734** | **1.288** | **0.01** | **0.295** | **13.026** | **0.0003** | **16.582** | **Purifying** | **cox3** |
| **1,735** | **0** | **0** | **0** | **0** | **1** | **0** | **Invariable** | **cox3** |
| **1,736** | **0.037** | **0.015** | **0.019** | **0.064** | **0.7998** | **1.069** | **Neutral** | **cox3** |
| **1,737** | **0.129** | **0.027** | **0.039** | **0.465** | **0.4953** | **2.202** | **Neutral** | **cox3** |
| **1,738** | **0.129** | **0.027** | **0.039** | **0.465** | **0.4953** | **2.202** | **Neutral** | **cox3** |
| **1,739** | **3.41** | **0.008** | **0.062** | **15.186** | **0.0001** | **3.511** | **Purifying** | **cox3** |
| **1,740** | **0.796** | **0.014** | **0.221** | **6.912** | **0.0086** | **12.411** | **Purifying** | **cox3** |
| **1,741** | **1.859** | **0.025** | **0.411** | **15.164** | **0.0001** | **23.09** | **Purifying** | **cox3** |
| **1,742** | **0.103** | **0.029** | **0.038** | **0.386** | **0.5344** | **2.124** | **Neutral** | **cox3** |
| **1,743** | **0** | **0.023** | **0.018** | **0.993** | **0.3191** | **1.004** | **Neutral** | **cox3** |
| **1,744** | **0.426** | **0.019** | **0.024** | **1.53** | **0.2162** | **1.352** | **Neutral** | **cox3** |
| **1,745** | **0.103** | **0.029** | **0.038** | **0.386** | **0.5344** | **2.124** | **Neutral** | **cox3** |
| **1,746** | **0.118** | **0.041** | **0.048** | **0.382** | **0.5365** | **2.696** | **Neutral** | **cox3** |
| **1,747** | **2.96** | **0.017** | **0.128** | **11.991** | **0.0005** | **7.174** | **Purifying** | **cox3** |
| **1,748** | **0.757** | **0.035** | **0.077** | **2.59** | **0.1075** | **4.312** | **Neutral** | **cox3** |
| **1,749** | **0.036** | **0.029** | **0.031** | **0.017** | **0.8966** | **1.738** | **Neutral** | **cox3** |
| **1,750** | **1.566** | **0.013** | **0.475** | **9.872** | **0.0017** | **26.685** | **Purifying** | **cox3** |
| **1,751** | **0.58** | **0.013** | **0.086** | **4.898** | **0.0269** | **4.844** | **Purifying** | **cox3** |
| **1,752** | **3.093** | **0.029** | **0.423** | **12.224** | **0.0005** | **23.789** | **Purifying** | **cox3** |
| **1,753** | **0.551** | **0.015** | **0.033** | **2.032** | **0.154** | **1.851** | **Neutral** | **cox3** |
| **1,754** | **0.707** | **0.012** | **0.076** | **8.224** | **0.0041** | **4.276** | **Purifying** | **cox3** |
| **1,755** | **8.927** | **0.035** | **0.08** | **5.925** | **0.0149** | **4.475** | **Purifying** | **cox3** |
| **1,756** | **2.178** | **0.056** | **0.722** | **8.569** | **0.0034** | **40.543** | **Purifying** | **cox3** |
| **1,757** | **2.074** | **0.021** | **0.263** | **14.477** | **0.0001** | **14.777** | **Purifying** | **cox3** |
| **1,758** | **0.176** | **0.01** | **0.038** | **3.79** | **0.0516** | **2.122** | **Neutral** | **cox3** |
| **1,759** | **0.139** | **0.023** | **0.033** | **0.775** | **0.3788** | **1.831** | **Neutral** | **cox3** |
| **1,760** | **1.232** | **0.012** | **0.109** | **9.891** | **0.0017** | **6.101** | **Purifying** | **cox3** |
| **1,761** | **0.554** | **0.012** | **0.038** | **4.134** | **0.042** | **2.114** | **Purifying** | **cox3** |
| **1,762** | **0.581** | **0.008** | **0.033** | **5.459** | **0.0195** | **1.865** | **Purifying** | **cox3** |
| **1,763** | **0.705** | **0.025** | **0.03** | **1.967** | **0.1608** | **1.688** | **Neutral** | **cox3** |
| **1,764** | **2.187** | **0.022** | **0.381** | **11.986** | **0.0005** | **21.393** | **Purifying** | **cox3** |
| **1,765** | **0.069** | **0.019** | **0.028** | **0.693** | **0.4053** | **1.566** | **Neutral** | **cox3** |
| **1,766** | **1.004** | **0** | **0.074** | **15.066** | **0.0001** | **4.147** | **Purifying** | **cox3** |
| **1,767** | **2.009** | **0.016** | **0.669** | **9.641** | **0.0019** | **37.6** | **Purifying** | **cox3** |
| **1,768** | **3.032** | **0.026** | **0.075** | **10.243** | **0.0014** | **4.234** | **Purifying** | **cox3** |
| **1,769** | **1.481** | **0.013** | **0.189** | **9.467** | **0.0021** | **10.637** | **Purifying** | **cox3** |
| **1,770** | **0** | **0.027** | **0.021** | **1.004** | **0.3163** | **1.191** | **Neutral** | **cox3** |
| **1,771** | **1.749** | **0.025** | **0.043** | **5.473** | **0.0193** | **2.429** | **Purifying** | **cox3** |
| **1,772** | **2.68** | **0.02** | **0.37** | **13.352** | **0.0003** | **20.808** | **Purifying** | **cox3** |
| **1,773** | **1.504** | **0.033** | **0.378** | **9.377** | **0.0022** | **21.246** | **Purifying** | **cox3** |
| **1,774** | **0.247** | **0.021** | **0.055** | **1.515** | **0.2184** | **3.088** | **Neutral** | **cox3** |
| **1,775** | **2.661** | **0.048** | **0.631** | **10.854** | **0.001** | **35.44** | **Purifying** | **cox3** |
| **1,776** | **44.206** | **0.018** | **0.35** | **19.255** | **0** | **19.64** | **Purifying** | **cox3** |
| **1,777** | **0.549** | **0.029** | **0.059** | **2.835** | **0.0922** | **3.322** | **Neutral** | **cox3** |
| **1,778** | **1.021** | **0.027** | **0.137** | **6.917** | **0.0085** | **7.724** | **Purifying** | **cox3** |
| **1,779** | **1.994** | **0.026** | **0.075** | **8.329** | **0.0039** | **4.23** | **Purifying** | **cox3** |
| **1,780** | **3.054** | **0.021** | **1.078** | **13.136** | **0.0003** | **60.539** | **Purifying** | **cox3** |
| **1,781** | **0.694** | **0.032** | **0.111** | **4.831** | **0.028** | **6.246** | **Purifying** | **cox3** |
| **1,782** | **0.028** | **0.018** | **0.021** | **0.093** | **0.7606** | **1.181** | **Neutral** | **cox3** |
| **1,783** | **2.539** | **0.033** | **0.348** | **12.113** | **0.0005** | **19.564** | **Purifying** | **cox3** |
| **1,784** | **0.422** | **0.026** | **0.042** | **2.842** | **0.0918** | **2.349** | **Neutral** | **cox3** |
| **1,785** | **2.273** | **0.013** | **0.46** | **13.819** | **0.0002** | **25.823** | **Purifying** | **cox3** |
| **1,786** | **0.066** | **0.032** | **0.038** | **0.159** | **0.6899** | **2.127** | **Neutral** | **cox3** |
| **1,787** | **1.076** | **0.013** | **0.197** | **8.282** | **0.004** | **11.043** | **Purifying** | **cox3** |
| **1,788** | **0.028** | **0** | **0.006** | **2.784** | **0.0952** | **0.357** | **Neutral** | **cox3** |
| **1,789** | **0.511** | **0.019** | **0.024** | **1.788** | **0.1812** | **1.357** | **Neutral** | **cox3** |
| **1,790** | **0.952** | **0.017** | **0.027** | **3.415** | **0.0646** | **1.526** | **Neutral** | **cox3** |
| **1,791** | **3.172** | **0.017** | **0.448** | **15.402** | **0.0001** | **25.194** | **Purifying** | **cox3** |
| **1,792** | **0** | **0.006** | **0.005** | **0.329** | **0.5663** | **0.296** | **Neutral** | **cox3** |
| **1,793** | **0.313** | **0.015** | **0.067** | **3.498** | **0.0614** | **3.757** | **Neutral** | **cox3** |
| **1,794** | **0.014** | **0.289** | **0.177** | **0.844** | **0.3582** | **9.954** | **Neutral** | **cox3** |
| **1,795** | **0.161** | **0.042** | **0.05** | **0.221** | **0.638** | **2.813** | **Neutral** | **cox3** |
| **1,796** | **2.538** | **0.021** | **0.346** | **16.824** | **0** | **19.422** | **Purifying** | **cox3** |
| **1,797** | **0.161** | **0.042** | **0.05** | **0.221** | **0.638** | **2.813** | **Neutral** | **cox3** |
| **1,798** | **0.229** | **0.013** | **0.038** | **2.897** | **0.0887** | **2.109** | **Neutral** | **cox3** |
| **1,799** | **0** | **0.014** | **0.012** | **0.321** | **0.5709** | **0.684** | **Neutral** | **cox3** |
| **1,800** | **2.462** | **0.047** | **0.618** | **8.357** | **0.0038** | **34.718** | **Purifying** | **cox3** |
| **1,801** | **0** | **0.018** | **0.014** | **0.818** | **0.3658** | **0.813** | **Neutral** | **cox3** |
| **1,802** | **0.833** | **0.028** | **0.098** | **6.028** | **0.0141** | **5.48** | **Purifying** | **cox3** |
| **1,803** | **8.9** | **0.028** | **0.1** | **14.19** | **0.0002** | **5.618** | **Purifying** | **cox3** |
| **1,804** | **0.087** | **0.014** | **0.028** | **0.772** | **0.3796** | **1.595** | **Neutral** | **cox3** |
| **1,805** | **1.777** | **0.048** | **0.44** | **8.094** | **0.0044** | **24.737** | **Purifying** | **cox3** |
| **1,806** | **0** | **0.022** | **0.017** | **1.005** | **0.3162** | **0.954** | **Neutral** | **cox3** |
| **1,807** | **5.277** | **0.013** | **1.074** | **19.058** | **0** | **60.329** | **Purifying** | **cox3** |
| **1,808** | **1.836** | **0.03** | **0.336** | **10.865** | **0.001** | **18.884** | **Purifying** | **cox3** |
| **1,809** | **0.447** | **0.01** | **0.028** | **3.427** | **0.0641** | **1.563** | **Neutral** | **cox3** |
| **1,810** | **1.509** | **0.095** | **0.617** | **3.53** | **0.0603** | **34.638** | **Neutral** | **cox3** |
| **1,811** | **0.253** | **0.031** | **0.055** | **1.246** | **0.2643** | **3.1** | **Neutral** | **cox3** |
| **1,812** | **0.536** | **0.007** | **0.024** | **7.711** | **0.0055** | **1.349** | **Purifying** | **cox3** |
| **1,813** | **0.902** | **0.024** | **0.056** | **4.391** | **0.0361** | **3.15** | **Purifying** | **cox3** |
| **1,814** | **0.039** | **0.032** | **0.034** | **0.013** | **0.9083** | **1.91** | **Neutral** | **cox3** |
| **1,815** | **4.148** | **0.01** | **0.459** | **19.311** | **0** | **25.776** | **Purifying** | **cox3** |
| **1,816** | **3.251** | **0.024** | **0.343** | **13.34** | **0.0003** | **19.272** | **Purifying** | **cox3** |
| **1,817** | **0.149** | **0.009** | **0.018** | **1.223** | **0.2687** | **1.02** | **Neutral** | **cox3** |
| **1,818** | **0.681** | **0.038** | **0.193** | **4.691** | **0.0303** | **10.824** | **Purifying** | **cox3** |
| **1,819** | **0.15** | **0.042** | **0.052** | **0.254** | **0.6141** | **2.894** | **Neutral** | **cox3** |
| **1,820** | **0.739** | **0.022** | **0.051** | **3.568** | **0.0589** | **2.875** | **Neutral** | **cox3** |
| **1,821** | **0** | **0.037** | **0.027** | **0.873** | **0.3501** | **1.543** | **Neutral** | **cox3** |
| **1,822** | **10.625** | **0.013** | **0.123** | **14.541** | **0.0001** | **6.896** | **Purifying** | **cox3** |
| **1,823** | **0.038** | **0.041** | **0.04** | **0.002** | **0.9612** | **2.238** | **Neutral** | **cox3** |
| **1,824** | **1.53** | **0.023** | **0.081** | **9.335** | **0.0022** | **4.565** | **Purifying** | **cox3** |
| **1,825** | **9.102** | **0.037** | **0.08** | **6.318** | **0.012** | **4.508** | **Purifying** | **cox3** |
| **1,826** | **0.54** | **0.037** | **0.055** | **1.647** | **0.1994** | **3.076** | **Neutral** | **cox3** |
| **1,827** | **1.071** | **0.034** | **0.151** | **6.404** | **0.0114** | **8.477** | **Purifying** | **cox3** |
| **1,828** | **0.875** | **0.01** | **0.03** | **5.285** | **0.0215** | **1.693** | **Purifying** | **cox3** |
| **1,829** | **0.704** | **0.043** | **0.042** | **1.025** | **0.3113** | **2.384** | **Neutral** | **cox3** |
| **1,830** | **0.883** | **0.009** | **0.045** | **8.241** | **0.0041** | **2.539** | **Purifying** | **cox3** |
| **1,831** | **0.062** | **0.019** | **0.026** | **0.55** | **0.4583** | **1.438** | **Neutral** | **cox3** |
| **1,832** | **2.88** | **0.024** | **0.135** | **11.446** | **0.0007** | **7.608** | **Purifying** | **cox3** |
| **1,833** | **1.276** | **0** | **0.025** | **19.429** | **0** | **1.422** | **Purifying** | **cox3** |
| **1,834** | **2.817** | **0.024** | **0.586** | **15.601** | **0.0001** | **32.95** | **Purifying** | **cox3** |
| **1,835** | **2.158** | **0.041** | **0.084** | **9.027** | **0.0027** | **4.747** | **Purifying** | **cox3** |
| **1,836** | **0** | **0.02** | **0.015** | **1.004** | **0.3164** | **0.849** | **Neutral** | **cox3** |
| **1,837** | **0** | **0.026** | **0.017** | **0.169** | **0.681** | **0.98** | **Neutral** | **cox3** |
| **1,838** | **0.735** | **0.008** | **0.033** | **5.827** | **0.0158** | **1.88** | **Purifying** | **cox3** |
| **1,839** | **0** | **0.01** | **0.008** | **0.498** | **0.4805** | **0.44** | **Neutral** | **cox3** |
| **1,840** | **0.471** | **0.008** | **0.024** | **4.304** | **0.038** | **1.358** | **Purifying** | **cox3** |
| **1,841** | **0** | **0.012** | **0.009** | **0.53** | **0.4664** | **0.508** | **Neutral** | **cox3** |
| **1,842** | **0** | **0.025** | **0.018** | **1.329** | **0.249** | **0.988** | **Neutral** | **cox3** |
| **1,843** | **0.8** | **0.022** | **0.157** | **6.15** | **0.0131** | **8.827** | **Purifying** | **cox3** |
| **1,844** | **1.681** | **0.019** | **0.044** | **9.065** | **0.0026** | **2.495** | **Purifying** | **cox3** |
| **1,845** | **0.997** | **0.015** | **0.039** | **4.465** | **0.0346** | **2.201** | **Purifying** | **cox3** |
| **1,846** | **6.734** | **0.012** | **0.45** | **20.135** | **0** | **25.295** | **Purifying** | **cox3** |
| **1,847** | **0.088** | **0.022** | **0.03** | **0.408** | **0.5229** | **1.713** | **Neutral** | **cox3** |
| **1,848** | **0.723** | **0.019** | **0.066** | **5.228** | **0.0222** | **3.687** | **Purifying** | **cox3** |
| **1,849** | **4.03** | **0.029** | **0.118** | **11.308** | **0.0008** | **6.644** | **Purifying** | **cox3** |
| **1,850** | **1.397** | **0.019** | **0.048** | **8.716** | **0.0032** | **2.695** | **Purifying** | **cox3** |
| **1,851** | **1.638** | **0.031** | **0.087** | **8.708** | **0.0032** | **4.898** | **Purifying** | **cox3** |
| **1,852** | **2.496** | **0.028** | **0.449** | **12.559** | **0.0004** | **25.216** | **Purifying** | **cox3** |
| **1,853** | **1.928** | **0.02** | **0.098** | **11.15** | **0.0008** | **5.508** | **Purifying** | **cox3** |
| **1,854** | **2.973** | **0.029** | **0.366** | **12.658** | **0.0004** | **20.546** | **Purifying** | **cox3** |
| **1,855** | **2.48** | **0.03** | **0.517** | **14.41** | **0.0001** | **29.064** | **Purifying** | **cox3** |
| **1,856** | **0.129** | **0.027** | **0.039** | **0.465** | **0.4953** | **2.202** | **Neutral** | **cox3** |
| **1,857** | **0.554** | **0.013** | **0.052** | **3.582** | **0.0584** | **2.936** | **Neutral** | **cox3** |
| **1,858** | **0.457** | **0.013** | **0.054** | **3.563** | **0.0591** | **3.04** | **Neutral** | **cox3** |
| **1,859** | **0.552** | **0.009** | **0.079** | **6.467** | **0.011** | **4.457** | **Purifying** | **cox3** |
| **1,860** | **0.999** | **0.024** | **0.032** | **3.404** | **0.0651** | **1.787** | **Neutral** | **cox3** |
| **1,861** | **0.79** | **0.033** | **0.159** | **4.872** | **0.0273** | **8.929** | **Purifying** | **cox3** |
| **1,862** | **0.345** | **0.035** | **0.052** | **1.329** | **0.2489** | **2.929** | **Neutral** | **cox3** |
| **1,863** | **1.27** | **0.01** | **0.053** | **9.767** | **0.0018** | **2.969** | **Purifying** | **cox3** |
| **1,864** | **0.15** | **0.042** | **0.052** | **0.254** | **0.6141** | **2.894** | **Neutral** | **cox3** |
| **1,865** | **0.33** | **0.009** | **0.032** | **3.623** | **0.057** | **1.792** | **Neutral** | **cox3** |
| **1,866** | **0.042** | **0.03** | **0.03** | **0.005** | **0.9446** | **1.666** | **Neutral** | **cox3** |
| **1,867** | **3.048** | **0.034** | **0.084** | **9.526** | **0.002** | **4.722** | **Purifying** | **cox3** |
| **1,868** | **0.038** | **0.038** | **0.038** | **0** | **0.9897** | **2.142** | **Neutral** | **cox3** |
| **1,869** | **0.488** | **0.011** | **0.019** | **3.235** | **0.0721** | **1.052** | **Neutral** | **cox3** |
| **1,870** | **2.239** | **0.03** | **0.075** | **8.745** | **0.0031** | **4.221** | **Purifying** | **cox3** |
| **1,871** | **1.318** | **0.016** | **0.057** | **9.182** | **0.0024** | **3.185** | **Purifying** | **cox3** |
| **1,872** | **10.976** | **0.027** | **0.068** | **8.219** | **0.0041** | **3.848** | **Purifying** | **cox3** |
| **1,873** | **4.28** | **0.04** | **0.11** | **9.753** | **0.0018** | **6.179** | **Purifying** | **cox3** |
| **1,874** | **0.143** | **0.034** | **0.047** | **0.383** | **0.5361** | **2.663** | **Neutral** | **cox3** |
| **1,875** | **0.87** | **0.015** | **0.039** | **4.317** | **0.0377** | **2.199** | **Purifying** | **cox3** |
| **1,876** | **1.9** | **0.019** | **0.079** | **10.868** | **0.001** | **4.414** | **Purifying** | **cox3** |
| **1,877** | **0** | **0.023** | **0.018** | **0.993** | **0.3191** | **1.004** | **Neutral** | **cox3** |
| **1,878** | **0** | **0.038** | **0.031** | **1.012** | **0.3145** | **1.733** | **Neutral** | **cox3** |
| **1,879** | **0.65** | **0.019** | **0.031** | **2.326** | **0.1272** | **1.715** | **Neutral** | **cox3** |
| **1,880** | **2.789** | **0.026** | **0.361** | **13.751** | **0.0002** | **20.305** | **Purifying** | **cox3** |
| **1,881** | **0.565** | **0.024** | **0.108** | **5.539** | **0.0186** | **6.075** | **Purifying** | **cox3** |
| **1,882** | **4.756** | **0.034** | **0.108** | **11.84** | **0.0006** | **6.067** | **Purifying** | **cox3** |
| **1,883** | **10000** | **0.042** | **0.143** | **10.152** | **0.0014** | **8.024** | **Purifying** | **cox3** |
| **1,884** | **4.194** | **0.028** | **0.075** | **10.798** | **0.001** | **4.198** | **Purifying** | **cox3** |
| **1,885** | **0.071** | **0.009** | **0.019** | **1.43** | **0.2317** | **1.052** | **Neutral** | **cox3** |
| **1,886** | **0.039** | **0.023** | **0.027** | **0.108** | **0.7429** | **1.502** | **Neutral** | **cox3** |
| **1,887** | **0.52** | **0.019** | **0.034** | **2.503** | **0.1136** | **1.908** | **Neutral** | **cox3** |
| **1,888** | **0.282** | **0.009** | **0.024** | **1.993** | **0.158** | **1.323** | **Neutral** | **cox3** |
| **1,889** | **0.217** | **0.032** | **0.061** | **1.549** | **0.2133** | **3.442** | **Neutral** | **cox3** |
| **1,890** | **0.108** | **0.024** | **0.033** | **0.539** | **0.4627** | **1.865** | **Neutral** | **cox3** |
| **1,891** | **0** | **0.015** | **0.007** | **1.228** | **0.2677** | **0.419** | **Neutral** | **cox3** |
| **1,892** | **0.129** | **0.033** | **0.044** | **0.377** | **0.5392** | **2.448** | **Neutral** | **cox3** |
| **1,893** | **0.068** | **0.032** | **0.039** | **0.19** | **0.663** | **2.217** | **Neutral** | **cox3** |
| **1,894** | **0** | **0.011** | **0.008** | **0.558** | **0.4552** | **0.441** | **Neutral** | **cox3** |
| **1,895** | **1.455** | **0.009** | **0.336** | **12.516** | **0.0004** | **18.903** | **Purifying** | **cox3** |
| **1,896** | **0.071** | **0.033** | **0.038** | **0.099** | **0.7528** | **2.157** | **Neutral** | **cox3** |
| **1,897** | **0.027** | **0.009** | **0.014** | **0.483** | **0.487** | **0.802** | **Neutral** | **cox3** |
| **1,898** | **0** | **0.024** | **0.018** | **1.103** | **0.2937** | **1.013** | **Neutral** | **cox3** |
| **1,899** | **0** | **0.006** | **0.005** | **0.383** | **0.5358** | **0.258** | **Neutral** | **nad3** |
| **1,900** | **1.072** | **0.041** | **0.107** | **6.724** | **0.0095** | **6.034** | **Purifying** | **nad3** |
| **1,901** | **0** | **0.011** | **0.009** | **0.416** | **0.5191** | **0.51** | **Neutral** | **nad3** |
| **1,902** | **0.028** | **0.013** | **0.018** | **0.225** | **0.6354** | **1.033** | **Neutral** | **nad3** |
| **1,903** | **0.691** | **0.015** | **0.054** | **3.788** | **0.0516** | **3.027** | **Neutral** | **nad3** |
| **1,904** | **0.324** | **0.045** | **0.091** | **1.896** | **0.1685** | **5.121** | **Neutral** | **nad3** |
| **1,905** | **0.958** | **0.008** | **0.063** | **10.324** | **0.0013** | **3.546** | **Purifying** | **nad3** |
| **1,906** | **1.252** | **0.01** | **0.047** | **9.816** | **0.0017** | **2.625** | **Purifying** | **nad3** |
| **1,907** | **0.025** | **0.045** | **0.039** | **0.098** | **0.7539** | **2.176** | **Neutral** | **nad3** |
| **1,908** | **1.682** | **0.027** | **0.343** | **10.117** | **0.0015** | **19.247** | **Purifying** | **nad3** |
| **1,909** | **0.399** | **0.281** | **0.309** | **0.056** | **0.8127** | **17.377** | **Neutral** | **nad3** |
| **1,910** | **0** | **0.012** | **0.009** | **0.514** | **0.4734** | **0.508** | **Neutral** | **nad3** |
| **1,911** | **0.425** | **0.665** | **0.6** | **0.151** | **0.6974** | **33.706** | **Neutral** | **nad3** |
| **1,912** | **1.98** | **0** | **0.67** | **14.924** | **0.0001** | **37.651** | **Purifying** | **nad3** |
| **1,913** | **0.547** | **0** | **0.008** | **4.503** | **0.0338** | **0.422** | **Purifying** | **nad3** |
| **1,914** | **0.069** | **0.011** | **0.022** | **1.265** | **0.2607** | **1.237** | **Neutral** | **nad3** |
| **1,915** | **0** | **0.031** | **0.022** | **1.348** | **0.2455** | **1.228** | **Neutral** | **nad3** |
| **1,916** | **0.011** | **0.054** | **0.027** | **1.052** | **0.3051** | **1.496** | **Neutral** | **nad3** |
| **1,917** | **1.062** | **0.028** | **0.052** | **6.267** | **0.0123** | **2.917** | **Purifying** | **nad3** |
| **1,918** | **0.391** | **0.009** | **0.077** | **5.144** | **0.0233** | **4.3** | **Purifying** | **nad3** |
| **1,919** | **0.433** | **0.011** | **0.045** | **4.469** | **0.0345** | **2.513** | **Purifying** | **nad3** |
| **1,920** | **0.741** | **0.035** | **0.077** | **2.521** | **0.1124** | **4.305** | **Neutral** | **nad3** |
| **1,921** | **0.514** | **0.021** | **0.034** | **2.466** | **0.1164** | **1.927** | **Neutral** | **nad3** |
| **1,922** | **3.114** | **0** | **1** | **19.444** | **0** | **56.195** | **Purifying** | **nad3** |
| **1,923** | **1.22** | **0.034** | **0.351** | **8.303** | **0.004** | **19.729** | **Purifying** | **nad3** |
| **1,924** | **0.136** | **0.043** | **0.051** | **0.209** | **0.6476** | **2.892** | **Neutral** | **nad3** |
| **1,925** | **0.113** | **0.032** | **0.041** | **0.319** | **0.5724** | **2.33** | **Neutral** | **nad3** |
| **1,926** | **0** | **0.037** | **0.026** | **1.72** | **0.1896** | **1.444** | **Neutral** | **nad3** |
| **1,927** | **9.65** | **0.012** | **0.791** | **22.3** | **0** | **44.455** | **Purifying** | **nad3** |
| **1,928** | **3.256** | **0.036** | **0.266** | **13.324** | **0.0003** | **14.938** | **Purifying** | **nad3** |
| **1,929** | **1.288** | **0.029** | **0.08** | **8.242** | **0.0041** | **4.523** | **Purifying** | **nad3** |
| **1,930** | **6.102** | **0.039** | **0.309** | **18.316** | **0** | **17.337** | **Purifying** | **nad3** |
| **1,931** | **1.499** | **0.035** | **0.056** | **3.816** | **0.0508** | **3.17** | **Neutral** | **nad3** |
| **1,932** | **0.888** | **0.016** | **0.342** | **7.165** | **0.0074** | **19.202** | **Purifying** | **nad3** |
| **1,933** | **2.38** | **0.009** | **0.512** | **18.097** | **0** | **28.761** | **Purifying** | **nad3** |
| **1,934** | **1.539** | **0.023** | **0.076** | **10.018** | **0.0016** | **4.289** | **Purifying** | **nad3** |
| **1,935** | **0.129** | **0.033** | **0.044** | **0.377** | **0.5392** | **2.448** | **Neutral** | **nad3** |
| **1,936** | **2.542** | **0.012** | **0.062** | **7.063** | **0.0079** | **3.505** | **Purifying** | **nad3** |
| **1,937** | **2.473** | **0.024** | **0.043** | **10.37** | **0.0013** | **2.419** | **Purifying** | **nad3** |
| **1,938** | **6.939** | **0.018** | **0.396** | **21.372** | **0** | **22.249** | **Purifying** | **nad3** |
| **1,939** | **0.133** | **0.011** | **0.019** | **2.036** | **0.1536** | **1.091** | **Neutral** | **nad3** |
| **1,940** | **119.961** | **0.028** | **0.098** | **11.151** | **0.0008** | **5.499** | **Purifying** | **nad3** |
| **1,941** | **0.397** | **0.012** | **0.018** | **1.598** | **0.2062** | **1.032** | **Neutral** | **nad3** |
| **1,942** | **1.845** | **0** | **0.46** | **15.334** | **0.0001** | **25.817** | **Purifying** | **nad3** |
| **1,943** | **2.66** | **0.021** | **0.273** | **15.222** | **0.0001** | **15.326** | **Purifying** | **nad3** |
| **1,944** | **0.384** | **0.034** | **0.031** | **0.114** | **0.7353** | **1.757** | **Neutral** | **nad3** |
| **1,945** | **1.2** | **0.025** | **0.135** | **8.148** | **0.0043** | **7.581** | **Purifying** | **nad3** |
| **1,946** | **1.393** | **0.019** | **0.27** | **12.555** | **0.0004** | **15.194** | **Purifying** | **nad3** |
| **1,947** | **0.442** | **0.023** | **0.049** | **1.777** | **0.1825** | **2.732** | **Neutral** | **nad3** |
| **1,948** | **1.148** | **0.013** | **0.342** | **10.1** | **0.0015** | **19.231** | **Purifying** | **nad3** |
| **1,949** | **1.929** | **0.01** | **0.043** | **11.815** | **0.0006** | **2.423** | **Purifying** | **nad3** |
| **1,950** | **0.029** | **0.022** | **0.024** | **0.047** | **0.8282** | **1.324** | **Neutral** | **nad3** |
| **1,951** | **0.042** | **0** | **0.011** | **2.627** | **0.105** | **0.606** | **Neutral** | **nad3** |
| **1,952** | **0** | **0.016** | **0.01** | **0.871** | **0.3508** | **0.567** | **Neutral** | **nad3** |
| **1,953** | **2.209** | **0.015** | **0.083** | **10.943** | **0.0009** | **4.662** | **Purifying** | **nad3** |
| **1,954** | **0.476** | **0.021** | **0.023** | **1.249** | **0.2638** | **1.291** | **Neutral** | **nad3** |
| **1,955** | **0.307** | **0.039** | **0.047** | **0.11** | **0.7399** | **2.615** | **Neutral** | **nad3** |
| **1,956** | **0.99** | **0.012** | **0.106** | **7.511** | **0.0061** | **5.981** | **Purifying** | **nad3** |
| **1,957** | **0.677** | **0.036** | **0.106** | **4.972** | **0.0258** | **5.983** | **Purifying** | **nad3** |
| **1,958** | **1.449** | **0.018** | **0.332** | **9.833** | **0.0017** | **18.67** | **Purifying** | **nad3** |
| **1,959** | **0.045** | **0.013** | **0.019** | **0.548** | **0.4592** | **1.04** | **Neutral** | **nad3** |
| **1,960** | **2.779** | **0.009** | **0.186** | **14.951** | **0.0001** | **10.477** | **Purifying** | **nad3** |
| **1,961** | **3.667** | **0.013** | **0.89** | **16.022** | **0.0001** | **50.003** | **Purifying** | **nad3** |
| **1,962** | **0.07** | **0** | **0.021** | **3.344** | **0.0674** | **1.157** | **Neutral** | **nad3** |
| **1,963** | **0.567** | **0.011** | **0.034** | **4.789** | **0.0286** | **1.937** | **Purifying** | **nad3** |
| **1,964** | **1.051** | **0.026** | **0.047** | **4.224** | **0.0398** | **2.647** | **Purifying** | **nad3** |
| **1,965** | **0.454** | **0.013** | **0.123** | **5.071** | **0.0243** | **6.934** | **Purifying** | **nad3** |
| **1,966** | **1.464** | **0.035** | **0.079** | **4.789** | **0.0286** | **4.454** | **Purifying** | **nad3** |
| **1,967** | **0.337** | **0.02** | **0.032** | **1.29** | **0.2561** | **1.816** | **Neutral** | **nad3** |
| **1,968** | **0.621** | **0.009** | **0.025** | **2.938** | **0.0865** | **1.389** | **Neutral** | **nad3** |
| **1,969** | **2.393** | **0.032** | **0.469** | **9.991** | **0.0016** | **26.373** | **Purifying** | **nad3** |
| **1,970** | **0.334** | **0.016** | **0.036** | **1.129** | **0.288** | **2.044** | **Neutral** | **nad3** |
| **1,971** | **3.352** | **0** | **0.793** | **18.18** | **0** | **44.546** | **Purifying** | **nad3** |
| **1,972** | **3.647** | **0.029** | **0.422** | **13.171** | **0.0003** | **23.737** | **Purifying** | **nad3** |
| **1,973** | **3.468** | **0** | **0.394** | **20.131** | **0** | **22.154** | **Purifying** | **nad3** |
| **1,974** | **4.31** | **0.012** | **0.625** | **18.705** | **0** | **35.137** | **Purifying** | **nad3** |
| **1,975** | **2.526** | **0.02** | **0.245** | **14.986** | **0.0001** | **13.763** | **Purifying** | **nad3** |
| **1,976** | **2.869** | **0.035** | **0.098** | **9.805** | **0.0017** | **5.531** | **Purifying** | **nad3** |
| **1,977** | **5.501** | **0.026** | **0.548** | **16.482** | **0** | **30.798** | **Purifying** | **nad3** |
| **1,978** | **0.4** | **0** | **0.019** | **5.475** | **0.0193** | **1.095** | **Purifying** | **nad3** |
| **1,979** | **1.974** | **0.052** | **0.602** | **7.236** | **0.0071** | **33.842** | **Purifying** | **nad3** |
| **1,980** | **0.032** | **0.033** | **0.032** | **0** | **1** | **1.821** | **Neutral** | **nad3** |
| **1,981** | **2.544** | **0.042** | **0.118** | **7.84** | **0.0051** | **6.645** | **Purifying** | **nad3** |
| **1,982** | **2.13** | **0** | **0.652** | **15.633** | **0.0001** | **36.655** | **Purifying** | **nad3** |
| **1,983** | **2.693** | **0.02** | **0.841** | **13.214** | **0.0003** | **47.265** | **Purifying** | **nad3** |
| **1,984** | **2.009** | **0.024** | **0.126** | **9.436** | **0.0021** | **7.082** | **Purifying** | **nad3** |
| **1,985** | **3.242** | **0.024** | **0.368** | **14.043** | **0.0002** | **20.693** | **Purifying** | **nad3** |
| **1,986** | **1.262** | **0** | **0.242** | **12.121** | **0.0005** | **13.589** | **Purifying** | **nad3** |
| **1,987** | **0.035** | **0.017** | **0.021** | **0.054** | **0.8166** | **1.197** | **Neutral** | **nad3** |
| **1,988** | **1.08** | **0.028** | **0.34** | **4.872** | **0.0273** | **19.087** | **Purifying** | **nad3** |
| **1,989** | **2.01** | **0.013** | **0.423** | **14.092** | **0.0002** | **23.741** | **Purifying** | **nad3** |
| **1,990** | **0.536** | **0.009** | **0.031** | **4.606** | **0.0319** | **1.738** | **Purifying** | **nad3** |
| **1,991** | **0.793** | **0.039** | **0.216** | **4.476** | **0.0344** | **12.154** | **Purifying** | **nad3** |
| **1,992** | **0.69** | **0.027** | **0.04** | **1.712** | **0.1907** | **2.254** | **Neutral** | **nad3** |
| **1,993** | **0.882** | **0.009** | **0.026** | **7.373** | **0.0066** | **1.435** | **Purifying** | **nad3** |
| **1,994** | **1.628** | **0.021** | **0.497** | **8.208** | **0.0042** | **27.945** | **Purifying** | **nad3** |
| **1,995** | **0** | **0.028** | **0.02** | **1.358** | **0.2438** | **1.143** | **Neutral** | **nad3** |
| **1,996** | **1.699** | **0.038** | **0.491** | **9.129** | **0.0025** | **27.608** | **Purifying** | **nad3** |
| **1,997** | **2.381** | **0.021** | **0.239** | **14.178** | **0.0002** | **13.452** | **Purifying** | **nad3** |
| **1,998** | **1.855** | **0.019** | **0.567** | **11.596** | **0.0007** | **31.87** | **Purifying** | **nad3** |
| **1,999** | **0.319** | **0.031** | **0.053** | **1.557** | **0.2121** | **2.967** | **Neutral** | **nad3** |
| **2,000** | **0.458** | **0.036** | **0.05** | **1.473** | **0.2249** | **2.798** | **Neutral** | **nad3** |
| **2,001** | **10000** | **0.038** | **0.544** | **22.453** | **0** | **30.591** | **Purifying** | **nad3** |
| **2,002** | **0.793** | **0.016** | **0.107** | **5.877** | **0.0153** | **5.998** | **Purifying** | **nad3** |
| **2,003** | **0.071** | **0.021** | **0.032** | **0.199** | **0.6552** | **1.778** | **Neutral** | **nad3** |
| **2,004** | **0** | **0** | **0** | **0** | **1** | **0** | **Invariable** | **nad3** |
| **2,005** | **0** | **0.024** | **0.018** | **1.086** | **0.2975** | **1.018** | **Neutral** | **nad3** |
| **2,006** | **0.759** | **0.024** | **0.21** | **4.756** | **0.0292** | **11.814** | **Purifying** | **nad3** |
| **2,007** | **4.329** | **0.008** | **0.394** | **21.625** | **0** | **22.125** | **Purifying** | **nad3** |
| **2,008** | **0.408** | **0.025** | **0.047** | **2.146** | **0.1429** | **2.616** | **Neutral** | **nad3** |
| **2,009** | **0.033** | **0.013** | **0.019** | **0.293** | **0.5881** | **1.058** | **Neutral** | **nad3** |
| **2,010** | **4046.464** | **0.015** | **0.019** | **1.141** | **0.2854** | **1.078** | **Neutral** | **nad4l** |
| **2,011** | **0** | **0.042** | **0.03** | **1.73** | **0.1885** | **1.692** | **Neutral** | **nad4l** |
| **2,012** | **0.494** | **0.015** | **0.046** | **3.532** | **0.0602** | **2.602** | **Neutral** | **nad4l** |
| **2,013** | **0.021** | **0.019** | **0.02** | **0.007** | **0.9321** | **1.142** | **Neutral** | **nad4l** |
| **2,014** | **0.049** | **0** | **0.008** | **3.135** | **0.0766** | **0.452** | **Neutral** | **nad4l** |
| **2,015** | **0.039** | **0.01** | **0.016** | **0.628** | **0.428** | **0.926** | **Neutral** | **nad4l** |
| **2,016** | **0** | **0.031** | **0.021** | **1.542** | **0.2143** | **1.201** | **Neutral** | **nad4l** |
| **2,017** | **2.405** | **0.009** | **0.031** | **13.039** | **0.0003** | **1.758** | **Purifying** | **nad4l** |
| **2,018** | **0.983** | **0.01** | **0.091** | **8.943** | **0.0028** | **5.131** | **Purifying** | **nad4l** |
| **2,019** | **0.088** | **0.018** | **0.031** | **0.815** | **0.3667** | **1.762** | **Neutral** | **nad4l** |
| **2,020** | **0** | **0.025** | **0.018** | **1.329** | **0.249** | **0.988** | **Neutral** | **nad4l** |
| **2,021** | **0.696** | **0.008** | **0.017** | **4.641** | **0.0312** | **0.943** | **Purifying** | **nad4l** |
| **2,022** | **0.381** | **0.058** | **0.232** | **0.326** | **0.5681** | **13.017** | **Neutral** | **nad4l** |
| **2,023** | **0.821** | **0.049** | **0.112** | **3.725** | **0.0536** | **6.313** | **Neutral** | **nad4l** |
| **2,024** | **0** | **0** | **0** | **0** | **1** | **0** | **Invariable** | **nad4l** |
| **2,025** | **0.427** | **0.024** | **0.036** | **0.632** | **0.4267** | **2.018** | **Neutral** | **nad4l** |
| **2,026** | **0** | **0.007** | **0.006** | **0.092** | **0.7622** | **0.362** | **Neutral** | **nad4l** |
| **2,027** | **0.708** | **0.023** | **0.087** | **6.855** | **0.0088** | **4.861** | **Purifying** | **nad4l** |
| **2,028** | **0.601** | **0.02** | **0.222** | **5.57** | **0.0183** | **12.469** | **Purifying** | **nad4l** |
| **2,029** | **0.011** | **0.024** | **0.018** | **0.317** | **0.5732** | **0.987** | **Neutral** | **nad4l** |
| **2,030** | **0.886** | **0** | **0.016** | **10.021** | **0.0015** | **0.887** | **Purifying** | **nad4l** |
| **2,031** | **0.038** | **0.043** | **0.042** | **0.006** | **0.939** | **2.348** | **Neutral** | **nad4l** |
| **2,032** | **0.061** | **0.035** | **0.042** | **0.122** | **0.7267** | **2.35** | **Neutral** | **nad4l** |
| **2,033** | **1.007** | **0.031** | **0.126** | **6.862** | **0.0088** | **7.078** | **Purifying** | **nad4l** |
| **2,034** | **0.472** | **0.032** | **0.047** | **1.777** | **0.1825** | **2.613** | **Neutral** | **nad4l** |
| **2,035** | **0.257** | **0** | **0.038** | **6.345** | **0.0118** | **2.162** | **Purifying** | **nad4l** |
| **2,036** | **1.119** | **0.039** | **0.399** | **6.683** | **0.0097** | **22.435** | **Purifying** | **nad4l** |
| **2,037** | **2.142** | **0.016** | **0.065** | **11.072** | **0.0009** | **3.632** | **Purifying** | **nad4l** |
| **2,038** | **0.05** | **0.044** | **0.045** | **0.002** | **0.9623** | **2.528** | **Neutral** | **nad4l** |
| **2,039** | **0.917** | **0** | **0.379** | **12.405** | **0.0004** | **21.32** | **Purifying** | **nad4l** |
| **2,040** | **0** | **0.033** | **0.022** | **1.559** | **0.2118** | **1.23** | **Neutral** | **nad4l** |
| **2,041** | **2.45** | **0.024** | **0.054** | **10.827** | **0.001** | **3.011** | **Purifying** | **nad4l** |
| **2,042** | **0.312** | **0.041** | **0.075** | **1.142** | **0.2852** | **4.215** | **Neutral** | **nad4l** |
| **2,043** | **1.441** | **0.019** | **0.034** | **5.913** | **0.015** | **1.893** | **Purifying** | **nad4l** |
| **2,044** | **1.119** | **0.022** | **0.087** | **8.136** | **0.0043** | **4.907** | **Purifying** | **nad4l** |
| **2,045** | **0.079** | **0.063** | **0.068** | **0.016** | **0.9002** | **3.817** | **Neutral** | **nad4l** |
| **2,046** | **0.054** | **0.414** | **0.404** | **0.404** | **0.5252** | **22.677** | **Neutral** | **nad4l** |
| **2,047** | **1.13** | **0.019** | **0.069** | **7.404** | **0.0065** | **3.858** | **Purifying** | **nad4l** |
| **2,048** | **1.564** | **0.01** | **0.332** | **14.115** | **0.0002** | **18.632** | **Purifying** | **nad4l** |
| **2,049** | **0.022** | **0.013** | **0.017** | **0.072** | **0.7884** | **0.929** | **Neutral** | **nad4l** |
| **2,050** | **1.8** | **0.015** | **0.065** | **10.665** | **0.0011** | **3.643** | **Purifying** | **nad4l** |
| **2,051** | **0.839** | **0.018** | **0.076** | **6.085** | **0.0136** | **4.245** | **Purifying** | **nad4l** |
| **2,052** | **1.048** | **0.025** | **0.095** | **7.415** | **0.0065** | **5.33** | **Purifying** | **nad4l** |
| **2,053** | **2.136** | **0.033** | **0.827** | **10.467** | **0.0012** | **46.455** | **Purifying** | **nad4l** |
| **2,054** | **0.032** | **0.009** | **0.014** | **0.647** | **0.4211** | **0.773** | **Neutral** | **nad4l** |
| **2,055** | **1.263** | **0.018** | **0.46** | **8.246** | **0.0041** | **25.834** | **Purifying** | **nad4l** |
| **2,056** | **0.514** | **0.008** | **0.024** | **3.821** | **0.0506** | **1.327** | **Neutral** | **nad4l** |
| **2,057** | **1.193** | **0.028** | **0.23** | **6.809** | **0.0091** | **12.914** | **Purifying** | **nad4l** |
| **2,058** | **1.418** | **0.012** | **0.028** | **5.797** | **0.0161** | **1.555** | **Purifying** | **nad4l** |
| **2,059** | **0.037** | **0.032** | **0.034** | **0.007** | **0.9345** | **1.907** | **Neutral** | **nad4l** |
| **2,060** | **0.368** | **0.006** | **0.018** | **6.32** | **0.0119** | **1.036** | **Purifying** | **nad4l** |
| **2,061** | **1.269** | **0** | **0.1** | **16.319** | **0.0001** | **5.642** | **Purifying** | **nad4l** |
| **2,062** | **1.043** | **0.024** | **0.142** | **7.848** | **0.0051** | **7.996** | **Purifying** | **nad4l** |
| **2,063** | **1.716** | **0.042** | **0.132** | **8.785** | **0.003** | **7.418** | **Purifying** | **nad4l** |
| **2,064** | **0.873** | **0.022** | **0.032** | **3.335** | **0.0678** | **1.773** | **Neutral** | **nad4l** |
| **2,065** | **1.849** | **0.015** | **0.348** | **13.5** | **0.0002** | **19.547** | **Purifying** | **nad4l** |
| **2,066** | **1.849** | **0.025** | **0.307** | **11.559** | **0.0007** | **17.253** | **Purifying** | **nad4l** |
| **2,067** | **0.644** | **0.024** | **0.051** | **3.606** | **0.0576** | **2.88** | **Neutral** | **nad4l** |
| **2,068** | **2.547** | **0.013** | **0.397** | **12.893** | **0.0003** | **22.286** | **Purifying** | **nad4l** |
| **2,069** | **0** | **0.261** | **0.26** | **0.939** | **0.3325** | **14.618** | **Neutral** | **nad4l** |
| **2,070** | **1.001** | **0.02** | **0.065** | **7.668** | **0.0056** | **3.641** | **Purifying** | **nad4l** |
| **2,071** | **1.257** | **0.015** | **0.218** | **6.304** | **0.012** | **12.229** | **Purifying** | **nad4l** |
| **2,072** | **0.789** | **0.039** | **0.336** | **5.447** | **0.0196** | **18.896** | **Purifying** | **nad4l** |
| **2,073** | **0.044** | **0.044** | **0.044** | **0** | **1** | **2.469** | **Neutral** | **nad4l** |
| **2,074** | **1.112** | **0.024** | **0.085** | **7.886** | **0.005** | **4.797** | **Purifying** | **nad4l** |
| **2,075** | **1.332** | **0.014** | **0.113** | **9.703** | **0.0018** | **6.341** | **Purifying** | **nad4l** |
| **2,076** | **1.381** | **0.012** | **0.042** | **8.946** | **0.0028** | **2.36** | **Purifying** | **nad4l** |
| **2,077** | **5.333** | **0.017** | **0.061** | **21.671** | **0** | **3.442** | **Purifying** | **nad4l** |
| **2,078** | **1.718** | **0.035** | **0.077** | **4.792** | **0.0286** | **4.303** | **Purifying** | **nad4l** |
| **2,079** | **4.727** | **0.037** | **0.312** | **13.164** | **0.0003** | **17.519** | **Purifying** | **nad4l** |
| **2,080** | **0** | **0** | **0** | **0** | **1** | **0** | **Invariable** | **nad4l** |
| **2,081** | **0.063** | **0.025** | **0.033** | **0.336** | **0.5624** | **1.856** | **Neutral** | **nad4l** |
| **2,082** | **0.46** | **0.016** | **0.021** | **2.109** | **0.1464** | **1.155** | **Neutral** | **nad4l** |
| **2,083** | **1.986** | **0** | **0.695** | **14.952** | **0.0001** | **39.03** | **Purifying** | **nad4l** |
| **2,084** | **0.532** | **0.024** | **0.051** | **3.146** | **0.0761** | **2.889** | **Neutral** | **nad4l** |
| **2,085** | **3.07** | **0.02** | **0.834** | **13.989** | **0.0002** | **46.84** | **Purifying** | **nad4l** |
| **2,086** | **0.349** | **0.03** | **0.07** | **1.732** | **0.1882** | **3.958** | **Neutral** | **nad4l** |
| **2,087** | **0.022** | **0.011** | **0.013** | **0.077** | **0.7818** | **0.746** | **Neutral** | **nad4l** |
| **2,088** | **1.523** | **0.025** | **0.139** | **8.729** | **0.0031** | **7.832** | **Purifying** | **nad4l** |
| **2,089** | **2.06** | **0.015** | **0.349** | **12.336** | **0.0004** | **19.616** | **Purifying** | **nad4l** |
| **2,090** | **0.542** | **0.019** | **0.03** | **1.959** | **0.1616** | **1.693** | **Neutral** | **nad4l** |
| **2,091** | **1.681** | **0.012** | **0.417** | **13.569** | **0.0002** | **23.419** | **Purifying** | **nad4l** |
| **2,092** | **0.765** | **0.01** | **0.038** | **5.431** | **0.0198** | **2.155** | **Purifying** | **nad4l** |
| **2,093** | **0.679** | **0.017** | **0.04** | **3.39** | **0.0656** | **2.231** | **Neutral** | **nad4l** |
| **2,094** | **1.473** | **0.021** | **0.266** | **13.904** | **0.0002** | **14.96** | **Purifying** | **nad4l** |
| **2,095** | **0.54** | **0.031** | **0.039** | **1.511** | **0.2191** | **2.168** | **Neutral** | **nad4l** |
| **2,096** | **1.09** | **0.028** | **0.057** | **5.018** | **0.0251** | **3.2** | **Purifying** | **nad4l** |
| **2,097** | **0.083** | **0.021** | **0.029** | **0.29** | **0.5902** | **1.65** | **Neutral** | **nad4l** |
| **2,098** | **2.196** | **0.023** | **0.658** | **10.501** | **0.0012** | **36.954** | **Purifying** | **nad4l** |
| **2,099** | **0.167** | **0.022** | **0.036** | **0.705** | **0.401** | **2.04** | **Neutral** | **nad4l** |
| **2,100** | **0** | **0.01** | **0.007** | **0.472** | **0.4921** | **0.418** | **Neutral** | **nad4l** |
| **2,101** | **1.383** | **0.038** | **0.401** | **7.716** | **0.0055** | **22.552** | **Purifying** | **nad4l** |
| **2,102** | **1.02** | **0.041** | **0.054** | **3.02** | **0.0823** | **3.053** | **Neutral** | **nad4l** |
| **2,103** | **2.444** | **0.038** | **0.64** | **11.541** | **0.0007** | **35.979** | **Purifying** | **nad4l** |
| **2,104** | **0.845** | **0.014** | **0.199** | **6.825** | **0.009** | **11.169** | **Purifying** | **nad4l** |
| **2,105** | **0.149** | **0.049** | **0.059** | **0.221** | **0.6382** | **3.313** | **Neutral** | **nad4l** |
| **2,106** | **0.106** | **0.03** | **0.033** | **0.067** | **0.7951** | **1.863** | **Neutral** | **nad4l** |
| **2,107** | **0.069** | **0.011** | **0.016** | **0.49** | **0.484** | **0.911** | **Neutral** | **nad4** |
| **2,108** | **0.043** | **0.014** | **0.023** | **0.484** | **0.4868** | **1.264** | **Neutral** | **nad4** |
| **2,109** | **0** | **0.014** | **0.011** | **0.454** | **0.5004** | **0.621** | **Neutral** | **nad4** |
| **2,110** | **1.993** | **0.01** | **0.372** | **13.832** | **0.0002** | **20.928** | **Purifying** | **nad4** |
| **2,111** | **0.99** | **0** | **0.195** | **11.143** | **0.0008** | **10.946** | **Purifying** | **nad4** |
| **2,112** | **3.067** | **0.018** | **0.514** | **14.283** | **0.0002** | **28.851** | **Purifying** | **nad4** |
| **2,113** | **3.227** | **0.017** | **0.438** | **13.595** | **0.0002** | **24.608** | **Purifying** | **nad4** |
| **2,114** | **0.645** | **0.012** | **0.044** | **4.044** | **0.0443** | **2.495** | **Purifying** | **nad4** |
| **2,115** | **1.526** | **0** | **0.405** | **14.229** | **0.0002** | **22.778** | **Purifying** | **nad4** |
| **2,116** | **9236.621** | **0.006** | **0.006** | **1.461** | **0.2268** | **0.363** | **Neutral** | **nad4** |
| **2,117** | **1.959** | **0.024** | **0.505** | **8.286** | **0.004** | **28.384** | **Purifying** | **nad4** |
| **2,118** | **2.962** | **0.338** | **0.787** | **4.635** | **0.0313** | **44.191** | **Purifying** | **nad4** |
| **2,119** | **0.066** | **0.029** | **0.038** | **0.268** | **0.6044** | **2.112** | **Neutral** | **nad4** |
| **2,120** | **0.715** | **0.009** | **0.024** | **4.267** | **0.0389** | **1.333** | **Purifying** | **nad4** |
| **2,121** | **2.939** | **0.035** | **0.67** | **15.389** | **0.0001** | **37.638** | **Purifying** | **nad4** |
| **2,122** | **3.463** | **0** | **0.841** | **20.668** | **0** | **47.227** | **Purifying** | **nad4** |
| **2,123** | **0.616** | **0.53** | **0.549** | **0.015** | **0.903** | **30.819** | **Neutral** | **nad4** |
| **2,124** | **4.323** | **0.012** | **0.78** | **19.872** | **0** | **43.837** | **Purifying** | **nad4** |
| **2,125** | **0.237** | **0.027** | **0.063** | **2.448** | **0.1177** | **3.534** | **Neutral** | **nad4** |
| **2,126** | **9.059** | **0.009** | **0.023** | **8.14** | **0.0043** | **1.317** | **Purifying** | **nad4** |
| **2,127** | **2.757** | **0** | **0.717** | **16.181** | **0.0001** | **40.294** | **Purifying** | **nad4** |
| **2,128** | **1.529** | **0.011** | **0.104** | **10.707** | **0.0011** | **5.87** | **Purifying** | **nad4** |
| **2,129** | **2.689** | **0.028** | **0.553** | **15.3** | **0.0001** | **31.081** | **Purifying** | **nad4** |
| **2,130** | **2.614** | **0** | **0.587** | **23.545** | **0** | **32.989** | **Purifying** | **nad4** |
| **2,131** | **0.436** | **0.035** | **0.071** | **1.915** | **0.1664** | **3.965** | **Neutral** | **nad4** |
| **2,132** | **0.591** | **0.02** | **0.291** | **6.076** | **0.0137** | **16.343** | **Purifying** | **nad4** |
| **2,133** | **3.069** | **0.015** | **0.056** | **11.927** | **0.0006** | **3.161** | **Purifying** | **nad4** |
| **2,134** | **1.314** | **0.025** | **0.073** | **6.415** | **0.0113** | **4.088** | **Purifying** | **nad4** |
| **2,135** | **0.958** | **0.045** | **0.414** | **5.277** | **0.0216** | **23.264** | **Purifying** | **nad4** |
| **2,136** | **0** | **0.385** | **0.194** | **1.742** | **0.1869** | **10.883** | **Neutral** | **nad4** |
| **2,137** | **2.833** | **0.041** | **0.515** | **12.686** | **0.0004** | **28.946** | **Purifying** | **nad4** |
| **2,138** | **0.743** | **0.019** | **0.025** | **1.876** | **0.1708** | **1.413** | **Neutral** | **nad4** |
| **2,139** | **0.643** | **0.009** | **0.017** | **2.343** | **0.1258** | **0.934** | **Neutral** | **nad4** |
| **2,140** | **1.145** | **0.008** | **0.149** | **11.783** | **0.0006** | **8.373** | **Purifying** | **nad4** |
| **2,141** | **0** | **0.015** | **0.013** | **0.633** | **0.4261** | **0.714** | **Neutral** | **nad4** |
| **2,142** | **1.271** | **0.013** | **0.042** | **8.265** | **0.004** | **2.374** | **Purifying** | **nad4** |
| **2,143** | **1.22** | **0.062** | **0.398** | **3.721** | **0.0537** | **22.35** | **Neutral** | **nad4** |
| **2,144** | **1.451** | **0.019** | **0.291** | **8.007** | **0.0047** | **16.367** | **Purifying** | **nad4** |
| **2,145** | **3.99** | **0.014** | **0.12** | **12.91** | **0.0003** | **6.738** | **Purifying** | **nad4** |
| **2,146** | **1.432** | **0.043** | **0.108** | **7.401** | **0.0065** | **6.092** | **Purifying** | **nad4** |
| **2,147** | **1.308** | **0** | **0.018** | **10.881** | **0.001** | **0.993** | **Purifying** | **nad4** |
| **2,148** | **0.733** | **0.035** | **0.283** | **3.87** | **0.0491** | **15.898** | **Purifying** | **nad4** |
| **2,149** | **2.735** | **0.013** | **0.114** | **13.491** | **0.0002** | **6.406** | **Purifying** | **nad4** |
| **2,150** | **1.481** | **0.031** | **0.075** | **7.374** | **0.0066** | **4.191** | **Purifying** | **nad4** |
| **2,151** | **0.911** | **0.02** | **0.082** | **11.324** | **0.0008** | **4.605** | **Purifying** | **nad4** |
| **2,152** | **1.201** | **0.027** | **0.345** | **5.204** | **0.0225** | **19.37** | **Purifying** | **nad4** |
| **2,153** | **0** | **0.039** | **0.03** | **1.391** | **0.2383** | **1.698** | **Neutral** | **nad4** |
| **2,154** | **1.408** | **0** | **0.295** | **12.316** | **0.0004** | **16.569** | **Purifying** | **nad4** |
| **2,155** | **0.916** | **0.028** | **0.038** | **3.277** | **0.0703** | **2.157** | **Neutral** | **nad4** |
| **2,156** | **2.558** | **0.012** | **0.592** | **15.219** | **0.0001** | **33.27** | **Purifying** | **nad4** |
| **2,157** | **9.85** | **0.039** | **0.126** | **11.626** | **0.0007** | **7.069** | **Purifying** | **nad4** |
| **2,158** | **0.046** | **0.203** | **0.113** | **0.31** | **0.5778** | **6.348** | **Neutral** | **nad4** |
| **2,159** | **1.69** | **0.025** | **0.063** | **8.271** | **0.004** | **3.548** | **Purifying** | **nad4** |
| **2,160** | **0** | **0.008** | **0.006** | **0.541** | **0.4618** | **0.358** | **Neutral** | **nad4** |
| **2,161** | **2.417** | **0.016** | **0.04** | **12.19** | **0.0005** | **2.26** | **Purifying** | **nad4** |
| **2,162** | **1.22** | **0.018** | **0.118** | **8.111** | **0.0044** | **6.644** | **Purifying** | **nad4** |
| **2,163** | **1.002** | **0.057** | **0.168** | **2.831** | **0.0925** | **9.42** | **Neutral** | **nad4** |
| **2,164** | **0.74** | **0.03** | **0.069** | **3.048** | **0.0808** | **3.873** | **Neutral** | **nad4** |
| **2,165** | **0.529** | **0** | **0.015** | **7.213** | **0.0072** | **0.853** | **Purifying** | **nad4** |
| **2,166** | **1.844** | **0.017** | **0.142** | **8.445** | **0.0037** | **7.977** | **Purifying** | **nad4** |
| **2,167** | **1.037** | **0** | **0.017** | **9.607** | **0.0019** | **0.978** | **Purifying** | **nad4** |
| **2,168** | **2.457** | **0.049** | **0.765** | **9.942** | **0.0016** | **42.956** | **Purifying** | **nad4** |
| **2,169** | **1.922** | **0.027** | **0.444** | **11.949** | **0.0005** | **24.934** | **Purifying** | **nad4** |
| **2,170** | **2.741** | **0.016** | **0.754** | **11.353** | **0.0008** | **42.38** | **Purifying** | **nad4** |
| **2,171** | **0.956** | **0.014** | **0.063** | **7.462** | **0.0063** | **3.566** | **Purifying** | **nad4** |
| **2,172** | **0.037** | **0.007** | **0.012** | **0.999** | **0.3174** | **0.675** | **Neutral** | **nad4** |
| **2,173** | **1.691** | **0.02** | **0.44** | **9.552** | **0.002** | **24.707** | **Purifying** | **nad4** |
| **2,174** | **0.252** | **0.014** | **0.032** | **2.14** | **0.1435** | **1.823** | **Neutral** | **nad4** |
| **2,175** | **0.647** | **0.016** | **0.04** | **2.772** | **0.0959** | **2.247** | **Neutral** | **nad4** |
| **2,176** | **1.513** | **0.039** | **0.48** | **6.844** | **0.0089** | **26.991** | **Purifying** | **nad4** |
| **2,177** | **0.085** | **0** | **0.009** | **4.264** | **0.0389** | **0.491** | **Purifying** | **nad4** |
| **2,178** | **2.084** | **0.01** | **0.353** | **13.459** | **0.0002** | **19.817** | **Purifying** | **nad4** |
| **2,179** | **2.802** | **0** | **0.763** | **16.816** | **0** | **42.861** | **Purifying** | **nad4** |
| **2,180** | **0.543** | **0.025** | **0.041** | **2.188** | **0.1391** | **2.327** | **Neutral** | **nad4** |
| **2,181** | **189.58** | **0.01** | **0.065** | **19.643** | **0** | **3.675** | **Purifying** | **nad4** |
| **2,182** | **104.581** | **0.038** | **0.547** | **15.7** | **0.0001** | **30.753** | **Purifying** | **nad4** |
| **2,183** | **3.768** | **0.041** | **0.29** | **13.295** | **0.0003** | **16.268** | **Purifying** | **nad4** |
| **2,184** | **0.891** | **0.012** | **0.026** | **5.454** | **0.0195** | **1.444** | **Purifying** | **nad4** |
| **2,185** | **0.726** | **0.01** | **0.028** | **5.546** | **0.0185** | **1.548** | **Purifying** | **nad4** |
| **2,186** | **0** | **0.053** | **0.036** | **2.044** | **0.1528** | **2.038** | **Neutral** | **nad4** |
| **2,187** | **1.76** | **0.017** | **0.535** | **2.572** | **0.1088** | **30.042** | **Neutral** | **nad4** |
| **2,188** | **10.416** | **0.026** | **0.374** | **17.393** | **0** | **20.996** | **Purifying** | **nad4** |
| **2,189** | **2.038** | **0.033** | **0.536** | **11.858** | **0.0006** | **30.108** | **Purifying** | **nad4** |
| **2,190** | **1.354** | **0.026** | **0.506** | **4.752** | **0.0293** | **28.412** | **Purifying** | **nad4** |
| **2,191** | **42.458** | **0.036** | **0.097** | **9.463** | **0.0021** | **5.443** | **Purifying** | **nad4** |
| **2,192** | **2.306** | **0.008** | **0.363** | **16.985** | **0** | **20.397** | **Purifying** | **nad4** |
| **2,193** | **0** | **0.043** | **0.035** | **1.08** | **0.2988** | **1.947** | **Neutral** | **nad4** |
| **2,194** | **0.996** | **0.021** | **0.066** | **6.314** | **0.012** | **3.709** | **Purifying** | **nad4** |
| **2,195** | **2.119** | **0.32** | **0.879** | **4.449** | **0.0349** | **49.37** | **Purifying** | **nad4** |
| **2,196** | **2.458** | **0.035** | **0.153** | **10.357** | **0.0013** | **8.579** | **Purifying** | **nad4** |
| **2,197** | **0.821** | **0** | **0.034** | **12.114** | **0.0005** | **1.928** | **Purifying** | **nad4** |
| **2,198** | **2.104** | **0.026** | **0.377** | **6.6** | **0.0102** | **21.207** | **Purifying** | **nad4** |
| **2,199** | **0** | **0.012** | **0.008** | **0.615** | **0.4327** | **0.463** | **Neutral** | **nad4** |
| **2,200** | **2.266** | **0.037** | **0.624** | **10.437** | **0.0012** | **35.041** | **Purifying** | **nad4** |
| **2,201** | **0.615** | **0.019** | **0.026** | **1.515** | **0.2184** | **1.476** | **Neutral** | **nad4** |
| **2,202** | **0.063** | **0.03** | **0.036** | **0.207** | **0.649** | **2.007** | **Neutral** | **nad4** |
| **2,203** | **1.262** | **0.012** | **0.081** | **8.528** | **0.0035** | **4.542** | **Purifying** | **nad4** |
| **2,204** | **88.024** | **0.012** | **0.06** | **19.794** | **0** | **3.36** | **Purifying** | **nad4** |
| **2,205** | **1.696** | **0.02** | **0.159** | **9.422** | **0.0021** | **8.91** | **Purifying** | **nad4** |
| **2,206** | **0** | **0.027** | **0.021** | **1.174** | **0.2785** | **1.175** | **Neutral** | **nad4** |
| **2,207** | **0.766** | **0** | **0.165** | **10.253** | **0.0014** | **9.27** | **Purifying** | **nad4** |
| **2,208** | **4.173** | **0** | **0.66** | **24.343** | **0** | **37.101** | **Purifying** | **nad4** |
| **2,209** | **0** | **0.03** | **0.024** | **1** | **0.3174** | **1.324** | **Neutral** | **nad4** |
| **2,210** | **0.54** | **0.008** | **0.026** | **5.73** | **0.0167** | **1.452** | **Purifying** | **nad4** |
| **2,211** | **0** | **0.01** | **0.009** | **0.202** | **0.6529** | **0.498** | **Neutral** | **nad4** |
| **2,212** | **0.53** | **0.021** | **0.027** | **1.843** | **0.1746** | **1.517** | **Neutral** | **nad4** |
| **2,213** | **0.072** | **0.025** | **0.035** | **0.456** | **0.4997** | **1.942** | **Neutral** | **nad4** |
| **2,214** | **0.51** | **0.01** | **0.017** | **2.315** | **0.1281** | **0.937** | **Neutral** | **nad4** |
| **2,215** | **3.945** | **0.027** | **0.074** | **11.72** | **0.0006** | **4.167** | **Purifying** | **nad4** |
| **2,216** | **1.739** | **0.025** | **0.122** | **7.869** | **0.005** | **6.866** | **Purifying** | **nad4** |
| **2,217** | **0.558** | **0.022** | **0.054** | **3.536** | **0.0601** | **3.053** | **Neutral** | **nad4** |
| **2,218** | **0** | **0.182** | **0.073** | **0.278** | **0.5981** | **4.077** | **Neutral** | **nad4** |
| **2,219** | **8.667** | **0.032** | **0.348** | **14.437** | **0.0001** | **19.539** | **Purifying** | **nad4** |
| **2,220** | **5.09** | **0.042** | **0.403** | **15.427** | **0.0001** | **22.661** | **Purifying** | **nad4** |
| **2,221** | **1.523** | **0.012** | **0.076** | **8.759** | **0.0031** | **4.276** | **Purifying** | **nad4** |
| **2,222** | **0.029** | **0.262** | **0.166** | **0.673** | **0.4121** | **9.299** | **Neutral** | **nad4** |
| **2,223** | **0.504** | **0.01** | **0.015** | **1.822** | **0.1771** | **0.833** | **Neutral** | **nad4** |
| **2,224** | **0.027** | **0.019** | **0.019** | **0.003** | **0.9533** | **1.079** | **Neutral** | **nad4** |
| **2,225** | **0** | **0** | **0** | **0** | **1** | **0** | **Invariable** | **nad4** |
| **2,226** | **0.511** | **0.024** | **0.03** | **1.607** | **0.205** | **1.686** | **Neutral** | **nad4** |
| **2,227** | **1.58** | **0.039** | **0.741** | **8.626** | **0.0033** | **41.624** | **Purifying** | **nad4** |
| **2,228** | **2.603** | **0.041** | **0.506** | **12.245** | **0.0005** | **28.448** | **Purifying** | **nad4** |
| **2,229** | **0.762** | **0.023** | **0.05** | **3.44** | **0.0636** | **2.83** | **Neutral** | **nad4** |
| **2,230** | **1.492** | **0.031** | **0.082** | **7.64** | **0.0057** | **4.634** | **Purifying** | **nad4** |
| **2,231** | **0** | **0.012** | **0.009** | **0.372** | **0.5419** | **0.533** | **Neutral** | **nad4** |
| **2,232** | **3.871** | **0.011** | **0.386** | **17.592** | **0** | **21.677** | **Purifying** | **nad4** |
| **2,233** | **3.518** | **0.04** | **0.874** | **13.112** | **0.0003** | **49.104** | **Purifying** | **nad4** |
| **2,234** | **1.573** | **0.018** | **0.353** | **10.331** | **0.0013** | **19.84** | **Purifying** | **nad4** |
| **2,235** | **0.735** | **0.008** | **0.031** | **5.604** | **0.0179** | **1.764** | **Purifying** | **nad4** |
| **2,236** | **0.461** | **0.008** | **0.035** | **4.954** | **0.026** | **1.981** | **Purifying** | **nad4** |
| **2,237** | **2.391** | **0.025** | **0.363** | **16.137** | **0.0001** | **20.387** | **Purifying** | **nad4** |
| **2,238** | **0.986** | **0.041** | **0.054** | **2.952** | **0.0858** | **3.049** | **Neutral** | **nad4** |
| **2,239** | **2.88** | **0.019** | **0.122** | **11.343** | **0.0008** | **6.882** | **Purifying** | **nad4** |
| **2,240** | **0.071** | **0.031** | **0.04** | **0.269** | **0.6043** | **2.258** | **Neutral** | **nad4** |
| **2,241** | **8.927** | **0.035** | **0.05** | **4.852** | **0.0276** | **2.827** | **Purifying** | **nad4** |
| **2,242** | **0.646** | **0.025** | **0.069** | **5.157** | **0.0232** | **3.875** | **Purifying** | **nad4** |
| **2,243** | **0.081** | **0.032** | **0.043** | **0.376** | **0.5398** | **2.438** | **Neutral** | **nad4** |
| **2,244** | **1.047** | **0.041** | **0.054** | **2.963** | **0.0852** | **3.04** | **Neutral** | **nad4** |
| **2,245** | **0** | **0.022** | **0.017** | **1** | **0.3173** | **0.949** | **Neutral** | **nad4** |
| **2,246** | **1.479** | **0.023** | **0.062** | **8.957** | **0.0028** | **3.461** | **Purifying** | **nad4** |
| **2,247** | **0.029** | **0.031** | **0.03** | **0.004** | **0.9499** | **1.705** | **Neutral** | **nad4** |
| **2,248** | **0.901** | **0.026** | **0.063** | **5.918** | **0.015** | **3.524** | **Purifying** | **nad4** |
| **2,249** | **0.663** | **0.034** | **0.05** | **2.287** | **0.1305** | **2.812** | **Neutral** | **nad4** |
| **2,250** | **1.001** | **0.019** | **0.223** | **7.152** | **0.0075** | **12.522** | **Purifying** | **nad4** |
| **2,251** | **3.035** | **0.027** | **0.38** | **14.403** | **0.0001** | **21.375** | **Purifying** | **nad4** |
| **2,252** | **9.246** | **0.023** | **0.554** | **20.748** | **0** | **31.105** | **Purifying** | **nad4** |
| **2,253** | **1.613** | **0** | **0.328** | **18.819** | **0** | **18.422** | **Purifying** | **nad4** |
| **2,254** | **1.722** | **0** | **0.719** | **15.087** | **0.0001** | **40.414** | **Purifying** | **nad4** |
| **2,255** | **1.629** | **0.009** | **0.126** | **11.189** | **0.0008** | **7.106** | **Purifying** | **nad4** |
| **2,256** | **0.607** | **0.023** | **0.045** | **3.308** | **0.0689** | **2.511** | **Neutral** | **nad4** |
| **2,257** | **2.424** | **0.016** | **0.482** | **16.188** | **0.0001** | **27.08** | **Purifying** | **nad4** |
| **2,258** | **0.451** | **0.038** | **0.055** | **1.403** | **0.2362** | **3.072** | **Neutral** | **nad4** |
| **2,259** | **1.345** | **0.012** | **0.105** | **9.881** | **0.0017** | **5.919** | **Purifying** | **nad4** |
| **2,260** | **1.642** | **0.021** | **0.43** | **9.36** | **0.0022** | **24.133** | **Purifying** | **nad4** |
| **2,261** | **1.03** | **0.013** | **0.241** | **7.63** | **0.0057** | **13.549** | **Purifying** | **nad4** |
| **2,262** | **0.99** | **0.024** | **0.077** | **6.954** | **0.0084** | **4.348** | **Purifying** | **nad4** |
| **2,263** | **3.514** | **0.026** | **0.326** | **12.951** | **0.0003** | **18.337** | **Purifying** | **nad4** |
| **2,264** | **3.064** | **0.013** | **0.797** | **15.546** | **0.0001** | **44.758** | **Purifying** | **nad4** |
| **2,265** | **2.323** | **0.024** | **0.879** | **10.866** | **0.001** | **49.376** | **Purifying** | **nad4** |
| **2,266** | **1.11** | **0.013** | **0.275** | **9.076** | **0.0026** | **15.443** | **Purifying** | **nad4** |
| **2,267** | **1.445** | **0** | **0.072** | **12.951** | **0.0003** | **4.055** | **Purifying** | **nad4** |
| **2,268** | **0** | **0.048** | **0.039** | **1.096** | **0.2951** | **2.209** | **Neutral** | **nad4** |
| **2,269** | **0.863** | **0.043** | **0.071** | **2.69** | **0.101** | **3.968** | **Neutral** | **nad4** |
| **2,270** | **0.094** | **0.014** | **0.03** | **0.949** | **0.3299** | **1.703** | **Neutral** | **nad4** |
| **2,271** | **0.023** | **0.008** | **0.013** | **0.371** | **0.5422** | **0.703** | **Neutral** | **nad4** |
| **2,272** | **2.185** | **0.007** | **0.236** | **18.58** | **0** | **13.271** | **Purifying** | **nad4** |
| **2,273** | **0.557** | **0.014** | **0.078** | **5.913** | **0.015** | **4.357** | **Purifying** | **nad4** |
| **2,274** | **0.437** | **0** | **0.03** | **7.316** | **0.0068** | **1.686** | **Purifying** | **nad4** |
| **2,275** | **0.035** | **0.032** | **0.032** | **0.003** | **0.9564** | **1.809** | **Neutral** | **nad4** |
| **2,276** | **1.111** | **0.033** | **0.398** | **7.115** | **0.0076** | **22.386** | **Purifying** | **nad4** |
| **2,277** | **2.382** | **0.029** | **0.694** | **10.419** | **0.0012** | **38.991** | **Purifying** | **nad4** |
| **2,278** | **1.233** | **0.031** | **0.059** | **6.951** | **0.0084** | **3.289** | **Purifying** | **nad4** |
| **2,279** | **2.195** | **0** | **0.748** | **15.891** | **0.0001** | **42.028** | **Purifying** | **nad4** |
| **2,280** | **0.13** | **0.049** | **0.057** | **0.142** | **0.7062** | **3.187** | **Neutral** | **nad4** |
| **2,281** | **2.247** | **0** | **0.34** | **21.842** | **0** | **19.127** | **Purifying** | **nad4** |
| **2,282** | **0.569** | **0.016** | **0.046** | **4.334** | **0.0373** | **2.573** | **Purifying** | **nad4** |
| **2,283** | **0.107** | **0.029** | **0.036** | **0.273** | **0.6017** | **2.03** | **Neutral** | **nad4** |
| **2,284** | **4.328** | **0.041** | **0.714** | **15.703** | **0.0001** | **40.109** | **Purifying** | **nad4** |
| **2,285** | **1.962** | **0.01** | **0.632** | **8.319** | **0.0039** | **35.489** | **Purifying** | **nad4** |
| **2,286** | **0.605** | **0** | **0.017** | **6.96** | **0.0083** | **0.946** | **Purifying** | **nad4** |
| **2,287** | **0.351** | **0** | **0.057** | **7.241** | **0.0071** | **3.224** | **Purifying** | **nad4** |
| **2,288** | **0.051** | **0.051** | **0.051** | **0** | **1** | **2.85** | **Neutral** | **nad4** |
| **2,289** | **0.534** | **0.024** | **0.047** | **2.966** | **0.085** | **2.668** | **Neutral** | **nad4** |
| **2,290** | **0.935** | **0.034** | **0.069** | **4.35** | **0.037** | **3.85** | **Purifying** | **nad4** |
| **2,291** | **1.532** | **0.037** | **0.301** | **9.574** | **0.002** | **16.917** | **Purifying** | **nad4** |
| **2,292** | **1.091** | **0.034** | **0.046** | **3.236** | **0.072** | **2.606** | **Neutral** | **nad4** |
| **2,293** | **10000** | **0.034** | **0.751** | **29.027** | **0** | **42.222** | **Purifying** | **nad4** |
| **2,294** | **1.36** | **0.014** | **0.492** | **9.387** | **0.0022** | **27.669** | **Purifying** | **nad4** |
| **2,295** | **0** | **0.533** | **0.526** | **0.772** | **0.3796** | **29.535** | **Neutral** | **nad4** |
| **2,296** | **1.121** | **0** | **0.016** | **13.764** | **0.0002** | **0.884** | **Purifying** | **nad4** |
| **2,297** | **1.708** | **0.017** | **0.044** | **11.319** | **0.0008** | **2.472** | **Purifying** | **nad4** |
| **2,298** | **3.077** | **0.016** | **0.911** | **12.182** | **0.0005** | **51.158** | **Purifying** | **nad4** |
| **2,299** | **0.712** | **0.133** | **0.404** | **1.231** | **0.2673** | **22.696** | **Neutral** | **nad4** |
| **2,300** | **1.484** | **0** | **0.156** | **17.173** | **0** | **8.743** | **Purifying** | **nad4** |
| **2,301** | **0.108** | **0.022** | **0.031** | **0.723** | **0.3952** | **1.734** | **Neutral** | **nad4** |
| **2,302** | **0.753** | **0.016** | **0.194** | **5.819** | **0.0159** | **10.877** | **Purifying** | **nad4** |
| **2,303** | **1.741** | **0.009** | **0.162** | **10.841** | **0.001** | **9.117** | **Purifying** | **nad4** |
| **2,304** | **0.217** | **0.02** | **0.028** | **0.76** | **0.3833** | **1.568** | **Neutral** | **nad4** |
| **2,305** | **0.005** | **0.425** | **0.367** | **1.173** | **0.2788** | **20.615** | **Neutral** | **nad4** |
| **2,306** | **3.27** | **0.032** | **0.592** | **14.044** | **0.0002** | **33.241** | **Purifying** | **nad4** |
| **2,307** | **1.413** | **0.016** | **0.403** | **8.715** | **0.0032** | **22.637** | **Purifying** | **nad4** |
| **2,308** | **1.064** | **0.03** | **0.056** | **6.882** | **0.0087** | **3.131** | **Purifying** | **nad4** |
| **2,309** | **2.03** | **0.009** | **0.261** | **15.908** | **0.0001** | **14.664** | **Purifying** | **nad4** |
| **2,310** | **0.603** | **0.009** | **0.041** | **5.351** | **0.0207** | **2.302** | **Purifying** | **nad4** |
| **2,311** | **1.023** | **0.038** | **0.053** | **3.084** | **0.0791** | **2.982** | **Neutral** | **nad4** |
| **2,312** | **1.979** | **0** | **0.589** | **16.604** | **0** | **33.082** | **Purifying** | **nad4** |
| **2,313** | **3.241** | **0** | **0.77** | **19.597** | **0** | **43.276** | **Purifying** | **nad4** |
| **2,314** | **2.691** | **0.041** | **0.513** | **11.737** | **0.0006** | **28.803** | **Purifying** | **nad4** |
| **2,315** | **2.993** | **0.035** | **0.057** | **5.316** | **0.0211** | **3.206** | **Purifying** | **nad4** |
| **2,316** | **0.065** | **0.01** | **0.021** | **1.165** | **0.2804** | **1.197** | **Neutral** | **nad4** |
| **2,317** | **1.795** | **0.024** | **0.089** | **10.519** | **0.0012** | **4.982** | **Purifying** | **nad4** |
| **2,318** | **0.053** | **0.044** | **0.046** | **0.004** | **0.9497** | **2.576** | **Neutral** | **nad4** |
| **2,319** | **113.23** | **0.008** | **0.499** | **22.813** | **0** | **28.053** | **Purifying** | **nad4** |
| **2,320** | **1.447** | **0.019** | **0.103** | **9.799** | **0.0017** | **5.796** | **Purifying** | **nad4** |
| **2,321** | **0.018** | **0.035** | **0.03** | **0.128** | **0.7207** | **1.686** | **Neutral** | **nad4** |
| **2,322** | **1.784** | **0.025** | **0.305** | **9.904** | **0.0016** | **17.11** | **Purifying** | **nad4** |
| **2,323** | **1.078** | **0.019** | **0.053** | **8.337** | **0.0039** | **2.987** | **Purifying** | **nad4** |
| **2,324** | **2.249** | **0.023** | **0.09** | **10.51** | **0.0012** | **5.083** | **Purifying** | **nad4** |
| **2,325** | **0.817** | **0.024** | **0.052** | **3.882** | **0.0488** | **2.917** | **Purifying** | **nad4** |
| **2,326** | **0** | **0.398** | **0.416** | **1.084** | **0.2977** | **23.395** | **Neutral** | **nad4** |
| **2,327** | **1.316** | **0.013** | **0.178** | **8.736** | **0.0031** | **10.004** | **Purifying** | **nad4** |
| **2,328** | **1.39** | **0.048** | **0.413** | **7.044** | **0.008** | **23.215** | **Purifying** | **nad4** |
| **2,329** | **1.856** | **0.011** | **0.409** | **14.46** | **0.0001** | **22.998** | **Purifying** | **nad4** |
| **2,330** | **0.722** | **0.025** | **0.033** | **1.657** | **0.1981** | **1.872** | **Neutral** | **nad4** |
| **2,331** | **1.486** | **0.019** | **0.048** | **9.095** | **0.0026** | **2.719** | **Purifying** | **nad4** |
| **2,332** | **4.593** | **0.048** | **1.299** | **16.038** | **0.0001** | **72.986** | **Purifying** | **nad4** |
| **2,333** | **0.815** | **0.051** | **0.388** | **4.681** | **0.0305** | **21.813** | **Purifying** | **nad4** |
| **2,334** | **0** | **0.04** | **0.032** | **0.975** | **0.3234** | **1.808** | **Neutral** | **nad4** |
| **2,335** | **0.324** | **0.048** | **0.084** | **0.874** | **0.3498** | **4.724** | **Neutral** | **nad4** |
| **2,336** | **2.728** | **0.019** | **0.498** | **19.709** | **0** | **27.983** | **Purifying** | **nad4** |
| **2,337** | **2.156** | **0.025** | **0.403** | **16.221** | **0.0001** | **22.616** | **Purifying** | **nad4** |
| **2,338** | **1.122** | **0.011** | **0.035** | **9.041** | **0.0026** | **1.952** | **Purifying** | **nad4** |
| **2,339** | **0.654** | **0.018** | **0.052** | **5.085** | **0.0241** | **2.948** | **Purifying** | **nad4** |
| **2,340** | **0.032** | **0.256** | **0.138** | **0.595** | **0.4406** | **7.776** | **Neutral** | **nad4** |
| **2,341** | **0.315** | **0.027** | **0.034** | **0.465** | **0.4954** | **1.911** | **Neutral** | **nad4** |
| **2,342** | **2.597** | **0.027** | **0.472** | **14.563** | **0.0001** | **26.537** | **Purifying** | **nad4** |
| **2,343** | **0.325** | **0.018** | **0.038** | **1.124** | **0.289** | **2.132** | **Neutral** | **nad4** |
| **2,344** | **1.629** | **0.037** | **0.801** | **8.579** | **0.0034** | **45.005** | **Purifying** | **nad4** |
| **2,345** | **1.42** | **0.009** | **0.303** | **12.529** | **0.0004** | **17.018** | **Purifying** | **nad4** |
| **2,346** | **4.716** | **0.009** | **0.659** | **21.759** | **0** | **37.03** | **Purifying** | **nad4** |
| **2,347** | **2.529** | **0.024** | **0.689** | **9.604** | **0.0019** | **38.703** | **Purifying** | **nad4** |
| **2,348** | **1.643** | **0.026** | **0.347** | **14.577** | **0.0001** | **19.519** | **Purifying** | **nad4** |
| **2,349** | **0** | **0.035** | **0.024** | **1.65** | **0.199** | **1.325** | **Neutral** | **nad4** |
| **2,350** | **0.734** | **0.019** | **0.171** | **6.008** | **0.0142** | **9.63** | **Purifying** | **nad4** |
| **2,351** | **1.389** | **0.01** | **0.049** | **10.36** | **0.0013** | **2.775** | **Purifying** | **nad4** |
| **2,352** | **0.251** | **0.033** | **0.04** | **0.192** | **0.6614** | **2.256** | **Neutral** | **nad4** |
| **2,353** | **10000** | **0.035** | **0.149** | **15.698** | **0.0001** | **8.352** | **Purifying** | **nad4** |
| **2,354** | **1.621** | **0.016** | **0.517** | **8.798** | **0.003** | **29.03** | **Purifying** | **nad4** |
| **2,355** | **3.462** | **0.023** | **0.068** | **12.957** | **0.0003** | **3.8** | **Purifying** | **nad4** |
| **2,356** | **3.581** | **0.042** | **0.368** | **14.031** | **0.0002** | **20.674** | **Purifying** | **nad4** |
| **2,357** | **1.219** | **0.043** | **0.432** | **8.445** | **0.0037** | **24.283** | **Purifying** | **nad4** |
| **2,358** | **0.504** | **0.011** | **0.034** | **4.6** | **0.032** | **1.93** | **Purifying** | **nad4** |
| **2,359** | **1.372** | **0.015** | **0.328** | **10.109** | **0.0015** | **18.405** | **Purifying** | **nad4** |
| **2,360** | **0** | **0.03** | **0.021** | **1.4** | **0.2367** | **1.195** | **Neutral** | **nad4** |
| **2,361** | **0.974** | **0.01** | **0.019** | **4.376** | **0.0365** | **1.076** | **Purifying** | **nad4** |
| **2,362** | **0.614** | **0.019** | **0.041** | **3.79** | **0.0516** | **2.323** | **Neutral** | **nad4** |
| **2,363** | **0.509** | **0.032** | **0.07** | **2.497** | **0.114** | **3.955** | **Neutral** | **nad4** |
| **2,364** | **0.303** | **0.019** | **0.049** | **3.804** | **0.0511** | **2.772** | **Neutral** | **nad4** |
| **2,365** | **0.029** | **0.029** | **0.029** | **0** | **0.9938** | **1.642** | **Neutral** | **nad4** |
| **2,366** | **0.56** | **0.031** | **0.066** | **2.867** | **0.0904** | **3.681** | **Neutral** | **nad4** |
| **2,367** | **0.012** | **0.386** | **0.32** | **1.096** | **0.2952** | **18.004** | **Neutral** | **nad4** |
| **2,368** | **1.92** | **0.012** | **0.118** | **10.583** | **0.0011** | **6.625** | **Purifying** | **nad4** |
| **2,369** | **0** | **0.019** | **0.015** | **0.96** | **0.3271** | **0.85** | **Neutral** | **nad4** |
| **2,370** | **0.389** | **0.03** | **0.042** | **1.101** | **0.2939** | **2.359** | **Neutral** | **nad4** |
| **2,371** | **1.443** | **0.022** | **0.332** | **10.204** | **0.0014** | **18.679** | **Purifying** | **nad4** |
| **2,372** | **2.79** | **0.02** | **0.054** | **12.523** | **0.0004** | **3.008** | **Purifying** | **nad4** |
| **2,373** | **1.363** | **0.016** | **0.223** | **7.417** | **0.0065** | **12.533** | **Purifying** | **nad4** |
| **2,374** | **0** | **0.026** | **0.018** | **1.339** | **0.2472** | **1.037** | **Neutral** | **nad4** |
| **2,375** | **5.717** | **0.025** | **0.091** | **13.867** | **0.0002** | **5.102** | **Purifying** | **nad4** |
| **2,376** | **0.06** | **0.024** | **0.031** | **0.276** | **0.5991** | **1.745** | **Neutral** | **nad4** |
| **2,377** | **0.855** | **0.024** | **0.033** | **3.316** | **0.0686** | **1.852** | **Neutral** | **nad4** |
| **2,378** | **1.46** | **0** | **0.524** | **13.482** | **0.0002** | **29.44** | **Purifying** | **nad4** |
| **2,379** | **72.078** | **0.014** | **0.111** | **14.971** | **0.0001** | **6.244** | **Purifying** | **nad4** |
| **2,380** | **0.561** | **0.034** | **0.063** | **2.667** | **0.1024** | **3.539** | **Neutral** | **nad4** |
| **2,381** | **1.383** | **0.038** | **0.181** | **6.328** | **0.0119** | **10.147** | **Purifying** | **nad4** |
| **2,382** | **0.848** | **0.033** | **0.163** | **5.932** | **0.0149** | **9.139** | **Purifying** | **nad4** |
| **2,383** | **2.842** | **0.01** | **0.393** | **16.116** | **0.0001** | **22.054** | **Purifying** | **nad4** |
| **2,384** | **0.979** | **0.011** | **0.023** | **4.642** | **0.0312** | **1.286** | **Purifying** | **nad4** |
| **2,385** | **1.847** | **0.014** | **0.468** | **14.123** | **0.0002** | **26.278** | **Purifying** | **nad4** |
| **2,386** | **0.689** | **0.031** | **0.048** | **1.668** | **0.1966** | **2.688** | **Neutral** | **nad4** |
| **2,387** | **1.808** | **0.009** | **0.339** | **13.153** | **0.0003** | **19.024** | **Purifying** | **nad4** |
| **2,388** | **2.787** | **0.008** | **0.038** | **13.563** | **0.0002** | **2.152** | **Purifying** | **nad4** |
| **2,389** | **1.048** | **0.041** | **0.065** | **3.188** | **0.0742** | **3.639** | **Neutral** | **nad4** |
| **2,390** | **0** | **0.05** | **0.024** | **2.527** | **0.1119** | **1.366** | **Neutral** | **nad4** |
| **2,391** | **2.253** | **0.018** | **0.056** | **14.84** | **0.0001** | **3.128** | **Purifying** | **nad4** |
| **2,392** | **3.771** | **0.024** | **0.972** | **13.621** | **0.0002** | **54.631** | **Purifying** | **nad4** |
| **2,393** | **0.615** | **0.009** | **0.043** | **5.143** | **0.0233** | **2.397** | **Purifying** | **nad4** |
| **2,394** | **1.007** | **0.037** | **0.187** | **5.909** | **0.0151** | **10.528** | **Purifying** | **nad4** |
| **2,395** | **4.293** | **0** | **0.52** | **31.523** | **0** | **29.21** | **Purifying** | **nad4** |
| **2,396** | **2.992** | **0.022** | **0.32** | **16.434** | **0.0001** | **17.971** | **Purifying** | **nad4** |
| **2,397** | **0.881** | **0.01** | **0.161** | **9.343** | **0.0022** | **9.061** | **Purifying** | **nad4** |
| **2,398** | **0.791** | **0.02** | **0.173** | **5.864** | **0.0155** | **9.723** | **Purifying** | **nad4** |
| **2,399** | **2.139** | **0.031** | **0.395** | **11.275** | **0.0008** | **22.202** | **Purifying** | **nad4** |
| **2,400** | **0.149** | **0.049** | **0.059** | **0.221** | **0.6382** | **3.313** | **Neutral** | **nad4** |
| **2,401** | **0** | **0.031** | **0.021** | **1.542** | **0.2143** | **1.201** | **Neutral** | **nad4** |
| **2,402** | **2.486** | **0.023** | **0.48** | **13.444** | **0.0002** | **26.95** | **Purifying** | **nad4** |
| **2,403** | **1.775** | **0.008** | **0.224** | **17.408** | **0** | **12.56** | **Purifying** | **nad4** |
| **2,404** | **0.497** | **0.011** | **0.031** | **6.04** | **0.014** | **1.729** | **Purifying** | **nad4** |
| **2,405** | **0.735** | **0.008** | **0.033** | **5.827** | **0.0158** | **1.88** | **Purifying** | **nad4** |
| **2,406** | **0.744** | **0.016** | **0.052** | **10.395** | **0.0013** | **2.939** | **Purifying** | **nad4** |
| **2,407** | **2.536** | **0.008** | **0.325** | **16.7** | **0** | **18.264** | **Purifying** | **nad4** |
| **2,408** | **1.527** | **0.013** | **0.118** | **9.329** | **0.0023** | **6.652** | **Purifying** | **nad4** |
| **2,409** | **1.474** | **0.041** | **0.296** | **8.31** | **0.0039** | **16.618** | **Purifying** | **nad4** |
| **2,410** | **1.605** | **0.05** | **0.664** | **7.487** | **0.0062** | **37.314** | **Purifying** | **nad4** |
| **2,411** | **0** | **0.555** | **0.546** | **1.452** | **0.2282** | **30.684** | **Neutral** | **nad4** |
| **2,412** | **3.762** | **0.01** | **0.528** | **17.837** | **0** | **29.692** | **Purifying** | **nad4** |
| **2,413** | **0.569** | **0.037** | **0.055** | **1.743** | **0.1868** | **3.086** | **Neutral** | **nad4** |
| **2,414** | **10000** | **0.026** | **0.429** | **17.404** | **0** | **24.092** | **Purifying** | **nad4** |
| **2,415** | **0.714** | **0.007** | **0.015** | **3.508** | **0.0611** | **0.831** | **Neutral** | **nad4** |
| **2,416** | **3.591** | **0.039** | **0.723** | **10.095** | **0.0015** | **40.648** | **Purifying** | **nad4** |
| **2,417** | **0.649** | **0.01** | **0.018** | **2.23** | **0.1354** | **0.985** | **Neutral** | **nad4** |
| **2,418** | **1.249** | **0.024** | **0.056** | **8.276** | **0.004** | **3.134** | **Purifying** | **nad4** |
| **2,419** | **4.389** | **0.015** | **0.391** | **16.52** | **0** | **21.976** | **Purifying** | **nad4** |
| **2,420** | **0.064** | **0** | **0.007** | **4.403** | **0.0359** | **0.398** | **Purifying** | **nad4** |
| **2,421** | **1.331** | **0.015** | **0.198** | **7.808** | **0.0052** | **11.138** | **Purifying** | **nad4** |
| **2,422** | **0.129** | **0.027** | **0.039** | **0.465** | **0.4953** | **2.202** | **Neutral** | **nad4** |
| **2,423** | **0.038** | **0.02** | **0.023** | **0.18** | **0.6711** | **1.307** | **Neutral** | **nad4** |
| **2,424** | **1.731** | **0.02** | **0.582** | **9.976** | **0.0016** | **32.686** | **Purifying** | **nad4** |
| **2,425** | **1.356** | **0.018** | **0.104** | **9.767** | **0.0018** | **5.861** | **Purifying** | **nad4** |
| **2,426** | **1.031** | **0.028** | **0.062** | **4.612** | **0.0317** | **3.506** | **Purifying** | **nad4** |
| **2,427** | **0.895** | **0.031** | **0.069** | **5.801** | **0.016** | **3.877** | **Purifying** | **nad4** |
| **2,428** | **1.737** | **0.043** | **0.104** | **9.609** | **0.0019** | **5.864** | **Purifying** | **nad4** |
| **2,429** | **3.346** | **0.029** | **0.541** | **16.509** | **0** | **30.401** | **Purifying** | **nad4** |
| **2,430** | **0** | **0.024** | **0.021** | **0.631** | **0.427** | **1.155** | **Neutral** | **nad4** |
| **2,431** | **1.714** | **0.028** | **0.085** | **8.815** | **0.003** | **4.79** | **Purifying** | **nad4** |
| **2,432** | **1.992** | **0** | **0.355** | **20.151** | **0** | **19.945** | **Purifying** | **nad4** |
| **2,433** | **0.474** | **0.029** | **0.032** | **1.414** | **0.2345** | **1.778** | **Neutral** | **nad4** |
| **2,434** | **0.039** | **0.008** | **0.014** | **0.87** | **0.3509** | **0.788** | **Neutral** | **nad4** |
| **2,435** | **0.458** | **0.008** | **0.035** | **4.961** | **0.0259** | **1.982** | **Purifying** | **nad4** |
| **2,436** | **2.89** | **0** | **0.546** | **23.253** | **0** | **30.687** | **Purifying** | **nad4** |
| **2,437** | **0** | **0.054** | **0.032** | **1.261** | **0.2614** | **1.799** | **Neutral** | **nad4** |
| **2,438** | **0.78** | **0.009** | **0.164** | **8.899** | **0.0029** | **9.237** | **Purifying** | **nad4** |
| **2,439** | **0.337** | **0.061** | **0.094** | **0.736** | **0.3909** | **5.274** | **Neutral** | **nad4** |
| **2,440** | **0.063** | **0.025** | **0.033** | **0.336** | **0.5624** | **1.856** | **Neutral** | **nad4** |
| **2,441** | **0.512** | **0.03** | **0.061** | **1.948** | **0.1628** | **3.445** | **Neutral** | **nad4** |
| **2,442** | **0.721** | **0.023** | **0.038** | **2.151** | **0.1425** | **2.12** | **Neutral** | **nad4** |
| **2,443** | **0.842** | **0.013** | **0.072** | **7.403** | **0.0065** | **4.041** | **Purifying** | **nad4** |
| **2,444** | **1.678** | **0.019** | **0.066** | **5.642** | **0.0175** | **3.719** | **Purifying** | **nad4** |
| **2,445** | **0** | **0.016** | **0.012** | **0.504** | **0.4778** | **0.669** | **Neutral** | **nad4** |
| **2,446** | **0.77** | **0.011** | **0.02** | **2.371** | **0.1236** | **1.123** | **Neutral** | **nad4** |
| **2,447** | **1.139** | **0.054** | **0.191** | **5.12** | **0.0237** | **10.754** | **Purifying** | **nad4** |
| **2,448** | **0.492** | **0.012** | **0.018** | **1.846** | **0.1743** | **1.035** | **Neutral** | **nad4** |
| **2,449** | **5.627** | **0.02** | **0.993** | **14.739** | **0.0001** | **55.819** | **Purifying** | **nad4** |
| **2,450** | **0** | **0.218** | **0.246** | **2.065** | **0.1508** | **13.8** | **Neutral** | **nad4** |
| **2,451** | **1.959** | **0.013** | **0.509** | **15.153** | **0.0001** | **28.583** | **Purifying** | **nad4** |
| **2,452** | **22.458** | **0.031** | **0.588** | **15.486** | **0.0001** | **33.062** | **Purifying** | **nad4** |
| **2,453** | **150.708** | **0.015** | **0.562** | **23.246** | **0** | **31.55** | **Purifying** | **nad4** |
| **2,454** | **0.824** | **0** | **0.034** | **12.085** | **0.0005** | **1.925** | **Purifying** | **nad4** |
| **2,455** | **3.67** | **0.018** | **0.299** | **17.649** | **0** | **16.825** | **Purifying** | **nad4** |
| **2,456** | **0.518** | **0.041** | **0.058** | **1.245** | **0.2644** | **3.286** | **Neutral** | **nad4** |
| **2,457** | **1.65** | **0.019** | **0.514** | **9.209** | **0.0024** | **28.89** | **Purifying** | **nad4** |
| **2,458** | **0.441** | **0.025** | **0.032** | **1.386** | **0.2391** | **1.791** | **Neutral** | **nad4** |
| **2,459** | **0.992** | **0.042** | **0.078** | **3.808** | **0.051** | **4.408** | **Neutral** | **nad4** |
| **2,460** | **0.09** | **0.049** | **0.058** | **0.15** | **0.699** | **3.252** | **Neutral** | **nad4** |
| **2,461** | **0.837** | **0.009** | **0.066** | **8.636** | **0.0033** | **3.726** | **Purifying** | **nad4** |
| **2,462** | **2.469** | **0.038** | **0.7** | **10.588** | **0.0011** | **39.303** | **Purifying** | **nad4** |
| **2,463** | **2.993** | **0.012** | **0.461** | **15.223** | **0.0001** | **25.91** | **Purifying** | **nad4** |
| **2,464** | **3.918** | **0.012** | **0.458** | **16.26** | **0.0001** | **25.737** | **Purifying** | **nad4** |
| **2,465** | **2.001** | **0** | **0.733** | **15.032** | **0.0001** | **41.162** | **Purifying** | **nad4** |
| **2,466** | **0.673** | **0.017** | **0.028** | **1.722** | **0.1894** | **1.565** | **Neutral** | **nad4** |
| **2,467** | **1.905** | **0.018** | **0.112** | **12.099** | **0.0005** | **6.286** | **Purifying** | **nad4** |
| **2,468** | **0.44** | **0.016** | **0.043** | **2.178** | **0.14** | **2.388** | **Neutral** | **nad4** |
| **2,469** | **0** | **0.34** | **0.405** | **1.628** | **0.202** | **22.747** | **Neutral** | **nad4** |
| **2,470** | **2.211** | **0.021** | **0.224** | **14.5** | **0.0001** | **12.579** | **Purifying** | **nad4** |
| **2,471** | **11.462** | **0.035** | **0.305** | **19.973** | **0** | **17.136** | **Purifying** | **nad4** |
| **2,472** | **3.457** | **0.024** | **0.913** | **12.697** | **0.0004** | **51.302** | **Purifying** | **nad4** |
| **2,473** | **0.032** | **0.273** | **0.143** | **0.646** | **0.4216** | **8.024** | **Neutral** | **nad4** |
| **2,474** | **0.476** | **0.02** | **0.026** | **1.808** | **0.1787** | **1.487** | **Neutral** | **nad4** |
| **2,475** | **1.865** | **0.022** | **0.382** | **11.623** | **0.0007** | **21.438** | **Purifying** | **nad4** |
| **2,476** | **0.5** | **0.013** | **0.021** | **2.126** | **0.1448** | **1.206** | **Neutral** | **nad4** |
| **2,477** | **1.85** | **0** | **0.048** | **16.696** | **0** | **2.721** | **Purifying** | **nad4** |
| **2,478** | **3.268** | **0** | **0.484** | **16.341** | **0.0001** | **27.215** | **Purifying** | **nad4** |
| **2,479** | **0.76** | **0.039** | **0.191** | **4.11** | **0.0426** | **10.73** | **Purifying** | **nad4** |
| **2,480** | **0.999** | **0.023** | **0.047** | **4.547** | **0.033** | **2.637** | **Purifying** | **nad4** |
| **2,481** | **0.755** | **0.009** | **0.034** | **5.311** | **0.0212** | **1.897** | **Purifying** | **nad4** |
| **2,482** | **1.188** | **0.031** | **0.081** | **7.051** | **0.0079** | **4.544** | **Purifying** | **nad4** |
| **2,483** | **4.357** | **0.024** | **1.219** | **15.15** | **0.0001** | **68.475** | **Purifying** | **nad4** |
| **2,484** | **1.116** | **0.039** | **0.242** | **5.36** | **0.0206** | **13.608** | **Purifying** | **nad4** |
| **2,485** | **4.457** | **0.02** | **0.928** | **15.526** | **0.0001** | **52.137** | **Purifying** | **nad4** |
| **2,486** | **1.297** | **0.02** | **0.284** | **9.484** | **0.0021** | **15.967** | **Purifying** | **nad4** |
| **2,487** | **0.8** | **0** | **0.161** | **20.189** | **0** | **9.065** | **Purifying** | **nad4** |
| **2,488** | **3.297** | **0.081** | **0.694** | **9.154** | **0.0025** | **38.991** | **Purifying** | **nad4** |
| **2,489** | **0.473** | **0.023** | **0.051** | **2.901** | **0.0885** | **2.883** | **Neutral** | **nad4** |
| **2,490** | **0.069** | **0.015** | **0.028** | **0.881** | **0.348** | **1.567** | **Neutral** | **nad4** |
| **2,491** | **1.331** | **0.033** | **0.448** | **7.376** | **0.0066** | **25.156** | **Purifying** | **nad4** |
| **2,492** | **1.115** | **0.042** | **0.135** | **5.779** | **0.0162** | **7.564** | **Purifying** | **nad4** |
| **2,493** | **1.108** | **0.009** | **0.035** | **9.285** | **0.0023** | **1.97** | **Purifying** | **nad4** |
| **2,494** | **1.015** | **0.025** | **0.125** | **7.469** | **0.0063** | **6.998** | **Purifying** | **nad4** |
| **2,495** | **2.446** | **0.009** | **0.437** | **20.398** | **0** | **24.555** | **Purifying** | **nad4** |
| **2,496** | **4.211** | **0.03** | **0.571** | **17.687** | **0** | **32.096** | **Purifying** | **nad4** |
| **2,497** | **1.216** | **0.034** | **0.073** | **6.757** | **0.0093** | **4.104** | **Purifying** | **nad4** |
| **2,498** | **1.528** | **0.013** | **0.456** | **10.183** | **0.0014** | **25.607** | **Purifying** | **nad4** |
| **2,499** | **43.657** | **0.014** | **0.504** | **19.879** | **0** | **28.323** | **Purifying** | **nad4** |
| **2,500** | **0.013** | **0.216** | **0.169** | **0.578** | **0.447** | **9.494** | **Neutral** | **nad4** |
| **2,501** | **131.489** | **0.009** | **0.383** | **21.785** | **0** | **21.538** | **Purifying** | **nad4** |
| **2,502** | **1.901** | **0.015** | **0.463** | **10.67** | **0.0011** | **26.031** | **Purifying** | **nad4** |
| **2,503** | **0** | **0.101** | **0.066** | **1.554** | **0.2125** | **3.685** | **Neutral** | **nad4** |
| **2,504** | **0.054** | **0.052** | **0.052** | **0** | **0.9892** | **2.93** | **Neutral** | **nad4** |
| **2,505** | **3.773** | **0.019** | **0.06** | **5.864** | **0.0155** | **3.356** | **Purifying** | **nad4** |
| **2,506** | **0.029** | **0.017** | **0.021** | **0.067** | **0.7953** | **1.177** | **Neutral** | **nad4** |
| **2,507** | **1.256** | **0.018** | **0.047** | **9.188** | **0.0024** | **2.657** | **Purifying** | **nad4** |
| **2,508** | **0.744** | **0.013** | **0.056** | **4.229** | **0.0397** | **3.137** | **Purifying** | **nad4** |
| **2,509** | **2.106** | **0** | **0.595** | **16.651** | **0** | **33.418** | **Purifying** | **nad4** |
| **2,510** | **0.468** | **0.017** | **0.027** | **1.703** | **0.192** | **1.512** | **Neutral** | **nad4** |
| **2,511** | **0.575** | **0.025** | **0.041** | **2.269** | **0.132** | **2.331** | **Neutral** | **nad4** |
| **2,512** | **1.717** | **0.041** | **0.059** | **3.774** | **0.052** | **3.309** | **Neutral** | **nad4** |
| **2,513** | **0.91** | **0.033** | **0.044** | **2.994** | **0.0835** | **2.454** | **Neutral** | **nad4** |
| **2,514** | **1.298** | **0.017** | **0.682** | **2.037** | **0.1535** | **38.335** | **Neutral** | **nad4** |
| **2,515** | **0.646** | **0.065** | **0.106** | **1.354** | **0.2445** | **5.973** | **Neutral** | **nad4** |
| **2,516** | **2.636** | **0** | **0.495** | **15.174** | **0.0001** | **27.787** | **Purifying** | **nad4** |
| **2,517** | **0** | **0.032** | **0.025** | **1.242** | **0.2651** | **1.377** | **Neutral** | **nad4** |
| **2,518** | **2.588** | **0.033** | **0.448** | **11.39** | **0.0007** | **25.168** | **Purifying** | **nad4** |
| **2,519** | **1.534** | **0.016** | **0.113** | **10.325** | **0.0013** | **6.325** | **Purifying** | **nad4** |
| **2,520** | **1.116** | **0.028** | **0.069** | **5.518** | **0.0188** | **3.898** | **Purifying** | **nad4** |
| **2,521** | **0.575** | **0.01** | **0.035** | **5.409** | **0.02** | **1.976** | **Purifying** | **nad4** |
| **2,522** | **0.6** | **0.02** | **0.046** | **3.993** | **0.0457** | **2.576** | **Purifying** | **nad4** |
| **2,523** | **3.499** | **0.035** | **0.05** | **4.572** | **0.0325** | **2.83** | **Purifying** | **nad4** |
| **2,524** | **3.019** | **0.04** | **0.116** | **10.161** | **0.0014** | **6.512** | **Purifying** | **nad4** |
| **2,525** | **2.458** | **0.035** | **0.657** | **11.572** | **0.0007** | **36.902** | **Purifying** | **nad4** |
| **2,526** | **0.07** | **0.036** | **0.041** | **0.148** | **0.7009** | **2.277** | **Neutral** | **nad4** |
| **2,527** | **0.351** | **0.02** | **0.032** | **1.328** | **0.2491** | **1.818** | **Neutral** | **nad4** |
| **2,528** | **0.761** | **0.019** | **0.038** | **4.071** | **0.0436** | **2.125** | **Purifying** | **nad4** |
| **2,529** | **0.26** | **0.018** | **0.027** | **1.27** | **0.2598** | **1.52** | **Neutral** | **nad4** |
| **2,530** | **0** | **0.017** | **0.012** | **0.733** | **0.392** | **0.648** | **Neutral** | **nad4** |
| **2,531** | **1.344** | **0.039** | **0.44** | **6.802** | **0.0091** | **24.702** | **Purifying** | **nad4** |
| **2,532** | **1.474** | **0.065** | **0.394** | **4.036** | **0.0446** | **22.153** | **Purifying** | **nad4** |
| **2,533** | **5.519** | **0.012** | **0.466** | **17.379** | **0** | **26.18** | **Purifying** | **nad4** |
| **2,534** | **2.728** | **0.023** | **0.957** | **12.301** | **0.0005** | **53.792** | **Purifying** | **nad4** |
| **2,535** | **1.229** | **0.023** | **0.249** | **6.812** | **0.0091** | **14.014** | **Purifying** | **nad4** |
| **2,536** | **0.391** | **0.038** | **0.046** | **0.988** | **0.3203** | **2.563** | **Neutral** | **nad4** |
| **2,537** | **1.492** | **0.019** | **0.415** | **9.834** | **0.0017** | **23.343** | **Purifying** | **nad4** |
| **2,538** | **0.534** | **0.03** | **0.052** | **1.613** | **0.204** | **2.91** | **Neutral** | **nad4** |
| **2,539** | **0.451** | **0** | **0.024** | **6.222** | **0.0126** | **1.353** | **Purifying** | **nad4** |
| **2,540** | **1.803** | **0.014** | **0.068** | **7.369** | **0.0066** | **3.838** | **Purifying** | **nad4** |
| **2,541** | **0.068** | **0.029** | **0.039** | **0.301** | **0.5835** | **2.17** | **Neutral** | **nad4** |
| **2,542** | **70.371** | **0.026** | **0.242** | **18.734** | **0** | **13.602** | **Purifying** | **nad4** |
| **2,543** | **0.374** | **0.013** | **0.049** | **2.812** | **0.0936** | **2.729** | **Neutral** | **nad4** |
| **2,544** | **1.181** | **0.01** | **0.138** | **10.128** | **0.0015** | **7.737** | **Purifying** | **nad4** |
| **2,545** | **1.203** | **0.023** | **0.283** | **12.135** | **0.0005** | **15.89** | **Purifying** | **nad4** |
| **2,546** | **0** | **0.031** | **0.022** | **1.223** | **0.2688** | **1.26** | **Neutral** | **nad4** |
| **2,547** | **0.512** | **0.008** | **0.025** | **3.757** | **0.0526** | **1.417** | **Neutral** | **nad4** |
| **2,548** | **0.066** | **0.057** | **0.059** | **0.005** | **0.9443** | **3.314** | **Neutral** | **nad5** |
| **2,549** | **0.101** | **0.068** | **0.08** | **0.066** | **0.7973** | **4.513** | **Neutral** | **nad5** |
| **2,550** | **0.081** | **0.049** | **0.064** | **0.095** | **0.7583** | **3.599** | **Neutral** | **nad5** |
| **2,551** | **1.541** | **0.025** | **0.333** | **9.654** | **0.0019** | **18.72** | **Purifying** | **nad5** |
| **2,552** | **0.161** | **0** | **0.019** | **8.207** | **0.0042** | **1.066** | **Purifying** | **nad5** |
| **2,553** | **1.416** | **0.144** | **0.533** | **4.233** | **0.0397** | **29.919** | **Purifying** | **nad5** |
| **2,554** | **0.288** | **0.19** | **0.212** | **0.054** | **0.8161** | **11.925** | **Neutral** | **nad5** |
| **2,555** | **0.31** | **0.025** | **0.081** | **2.613** | **0.106** | **4.567** | **Neutral** | **nad5** |
| **2,556** | **0.558** | **0.073** | **0.099** | **1.041** | **0.3076** | **5.565** | **Neutral** | **nad5** |
| **2,557** | **0.759** | **0.102** | **0.216** | **2.292** | **0.13** | **12.12** | **Neutral** | **nad5** |
| **2,558** | **2.211** | **0.126** | **0.276** | **6.125** | **0.0133** | **15.506** | **Purifying** | **nad5** |
| **2,559** | **0.232** | **0.071** | **0.073** | **0.207** | **0.6494** | **4.127** | **Neutral** | **nad5** |
| **2,560** | **0.172** | **0.062** | **0.095** | **0.526** | **0.4685** | **5.318** | **Neutral** | **nad5** |
| **2,561** | **0.526** | **0.174** | **0.216** | **0.481** | **0.488** | **12.16** | **Neutral** | **nad5** |
| **2,562** | **0** | **0.085** | **0.065** | **2.208** | **0.1373** | **3.658** | **Neutral** | **nad5** |
| **2,563** | **0.647** | **0.088** | **0.095** | **1.073** | **0.3002** | **5.362** | **Neutral** | **nad5** |
| **2,564** | **1.321** | **0.118** | **0.3** | **4.459** | **0.0347** | **16.841** | **Purifying** | **nad5** |
| **2,565** | **0.53** | **0.134** | **0.188** | **0.8** | **0.3712** | **10.59** | **Neutral** | **nad5** |
| **2,566** | **0.339** | **0.076** | **0.161** | **1.168** | **0.2798** | **9.049** | **Neutral** | **nad5** |
| **2,567** | **0.324** | **0.303** | **0.31** | **0.003** | **0.959** | **17.421** | **Neutral** | **nad5** |
| **2,568** | **2.378** | **0.185** | **0.673** | **6.446** | **0.0111** | **37.816** | **Purifying** | **nad5** |
| **2,569** | **0.586** | **0.079** | **0.214** | **1.919** | **0.166** | **12.042** | **Neutral** | **nad5** |
| **2,570** | **0.783** | **0** | **0.209** | **15.673** | **0.0001** | **11.763** | **Purifying** | **nad5** |
| **2,571** | **1.207** | **0.337** | **0.554** | **1.299** | **0.2545** | **31.138** | **Neutral** | **nad5** |
| **2,572** | **0** | **0.147** | **0.133** | **2.241** | **0.1344** | **7.456** | **Neutral** | **nad5** |
| **2,573** | **0.223** | **0.12** | **0.149** | **0.205** | **0.6506** | **8.362** | **Neutral** | **nad5** |
| **2,574** | **9.671** | **0.262** | **1.761** | **12.558** | **0.0004** | **98.95** | **Purifying** | **nad5** |
| **2,575** | **1.388** | **0.2** | **0.388** | **2.646** | **0.1038** | **21.779** | **Neutral** | **nad5** |
| **2,576** | **5.587** | **0.065** | **0.624** | **13.391** | **0.0003** | **35.059** | **Purifying** | **nad5** |
| **2,577** | **0.576** | **0.116** | **0.145** | **0.848** | **0.3571** | **8.137** | **Neutral** | **nad5** |
| **2,578** | **0.783** | **0.158** | **0.256** | **1.455** | **0.2277** | **14.4** | **Neutral** | **nad5** |
| **2,579** | **0** | **0.054** | **0.036** | **2.023** | **0.1549** | **1.995** | **Neutral** | **nad5** |
| **2,580** | **0** | **0.227** | **0.213** | **3.379** | **0.066** | **11.975** | **Neutral** | **nad5** |
| **2,581** | **1.108** | **0.528** | **0.677** | **0.56** | **0.4542** | **38.05** | **Neutral** | **nad5** |
| **2,582** | **0.909** | **0.225** | **0.305** | **1.062** | **0.3028** | **17.149** | **Neutral** | **nad5** |
| **2,583** | **2.327** | **0.03** | **0.327** | **10.022** | **0.0015** | **18.379** | **Purifying** | **nad5** |
| **2,584** | **2.45** | **0** | **0.595** | **17.015** | **0** | **33.446** | **Purifying** | **nad5** |
| **2,585** | **0.605** | **0.196** | **0.254** | **0.586** | **0.4439** | **14.257** | **Neutral** | **nad5** |
| **2,586** | **1.626** | **0.218** | **0.598** | **3.401** | **0.0652** | **33.604** | **Neutral** | **nad5** |
| **2,587** | **2.192** | **0.099** | **0.372** | **7.927** | **0.0049** | **20.878** | **Purifying** | **nad5** |
| **2,588** | **4.839** | **0.018** | **0.06** | **14.898** | **0.0001** | **3.395** | **Purifying** | **nad5** |
| **2,589** | **0.054** | **0.093** | **0.081** | **0.081** | **0.7754** | **4.533** | **Neutral** | **nad5** |
| **2,590** | **0.534** | **0** | **0.006** | **4.849** | **0.0277** | **0.359** | **Purifying** | **nad5** |
| **2,591** | **1.056** | **0.068** | **0.263** | **4.299** | **0.0381** | **14.79** | **Purifying** | **nad5** |
| **2,592** | **58.275** | **0.113** | **0.226** | **5.325** | **0.021** | **12.685** | **Purifying** | **nad5** |
| **2,593** | **2.032** | **0.111** | **0.691** | **6.26** | **0.0123** | **38.824** | **Purifying** | **nad5** |
| **2,594** | **0.223** | **0.059** | **0.095** | **0.831** | **0.362** | **5.359** | **Neutral** | **nad5** |
| **2,595** | **0** | **0.094** | **0.074** | **2.567** | **0.1091** | **4.183** | **Neutral** | **nad5** |
| **2,596** | **4.111** | **0.23** | **0.557** | **7.058** | **0.0079** | **31.283** | **Purifying** | **nad5** |
| **2,597** | **4.438** | **0.059** | **0.554** | **15.099** | **0.0001** | **31.148** | **Purifying** | **nad5** |
| **2,598** | **1.023** | **0.028** | **0.15** | **4.71** | **0.03** | **8.445** | **Purifying** | **nad5** |
| **2,599** | **1.509** | **0.185** | **0.495** | **4.275** | **0.0387** | **27.794** | **Purifying** | **nad5** |
| **2,600** | **4.887** | **0.138** | **1.176** | **12.108** | **0.0005** | **66.098** | **Purifying** | **nad5** |
| **2,601** | **1.532** | **0.033** | **0.33** | **8.015** | **0.0046** | **18.52** | **Purifying** | **nad5** |
| **2,602** | **4.097** | **0.139** | **0.993** | **10.254** | **0.0014** | **55.78** | **Purifying** | **nad5** |
| **2,603** | **1.632** | **0.022** | **0.201** | **8.92** | **0.0028** | **11.311** | **Purifying** | **nad5** |
| **2,604** | **2.48** | **0.167** | **0.621** | **7.712** | **0.0055** | **34.88** | **Purifying** | **nad5** |
| **2,605** | **0.93** | **0.064** | **0.103** | **1.912** | **0.1668** | **5.792** | **Neutral** | **nad5** |
| **2,606** | **1.909** | **0.184** | **0.521** | **5.336** | **0.0209** | **29.261** | **Purifying** | **nad5** |
| **2,607** | **44.23** | **0.056** | **0.684** | **22.273** | **0** | **38.409** | **Purifying** | **nad5** |
| **2,608** | **3.931** | **0.064** | **0.147** | **5.05** | **0.0246** | **8.276** | **Purifying** | **nad5** |
| **2,609** | **1.907** | **0.097** | **0.378** | **8.687** | **0.0032** | **21.239** | **Purifying** | **nad5** |
| **2,610** | **0** | **0.081** | **0.065** | **1.837** | **0.1752** | **3.643** | **Neutral** | **nad5** |
| **2,611** | **3.418** | **0.15** | **0.341** | **7.146** | **0.0075** | **19.138** | **Purifying** | **nad5** |
| **2,612** | **1.653** | **0.078** | **0.232** | **5.163** | **0.0231** | **13.032** | **Purifying** | **nad5** |
| **2,613** | **1.247** | **0.156** | **0.286** | **3.286** | **0.0699** | **16.07** | **Neutral** | **nad5** |
| **2,614** | **1.502** | **0.148** | **0.369** | **5.155** | **0.0232** | **20.721** | **Purifying** | **nad5** |
| **2,615** | **2.031** | **0.059** | **0.25** | **5.929** | **0.0149** | **14.027** | **Purifying** | **nad5** |
| **2,616** | **0.111** | **0.049** | **0.071** | **0.245** | **0.6209** | **3.996** | **Neutral** | **nad5** |
| **2,617** | **0** | **0.062** | **0.035** | **2.18** | **0.1398** | **1.967** | **Neutral** | **nad5** |
| **2,618** | **0.283** | **0.192** | **0.212** | **0.046** | **0.8302** | **11.89** | **Neutral** | **nad5** |
| **2,619** | **2.914** | **0.075** | **0.468** | **10.175** | **0.0014** | **26.296** | **Purifying** | **nad5** |
| **2,620** | **0** | **0.064** | **0.032** | **2.293** | **0.13** | **1.804** | **Neutral** | **nad5** |
| **2,621** | **3.546** | **0.11** | **1.153** | **10.542** | **0.0012** | **64.792** | **Purifying** | **nad5** |
| **2,622** | **0** | **0.02** | **0.014** | **1.136** | **0.2865** | **0.81** | **Neutral** | **nad5** |
| **2,623** | **0.443** | **0.029** | **0.092** | **2.941** | **0.0864** | **5.193** | **Neutral** | **nad5** |
| **2,624** | **3.163** | **0.025** | **0.124** | **13.076** | **0.0003** | **6.962** | **Purifying** | **nad5** |
| **2,625** | **1.576** | **0.339** | **0.572** | **1.87** | **0.1715** | **32.158** | **Neutral** | **nad5** |
| **2,626** | **2.106** | **0.076** | **0.312** | **6.007** | **0.0143** | **17.515** | **Purifying** | **nad5** |
| **2,627** | **1.329** | **0.112** | **0.377** | **5.641** | **0.0175** | **21.172** | **Purifying** | **nad5** |
| **2,628** | **0.86** | **0.025** | **0.054** | **2.957** | **0.0855** | **3.013** | **Neutral** | **nad5** |
| **2,629** | **0.204** | **0.034** | **0.08** | **1.259** | **0.2619** | **4.508** | **Neutral** | **nad5** |
| **2,630** | **1.656** | **0.018** | **0.031** | **5.342** | **0.0208** | **1.767** | **Purifying** | **nad5** |
| **2,631** | **3.601** | **0** | **0.351** | **23.043** | **0** | **19.728** | **Purifying** | **nad5** |
| **2,632** | **0.959** | **0.077** | **0.155** | **2.114** | **0.1459** | **8.726** | **Neutral** | **nad5** |
| **2,633** | **2.82** | **0.129** | **0.377** | **6.03** | **0.0141** | **21.172** | **Purifying** | **nad5** |
| **2,634** | **1.019** | **0** | **0.029** | **12.652** | **0.0004** | **1.625** | **Purifying** | **nad5** |
| **2,635** | **1.823** | **0.113** | **0.396** | **7.085** | **0.0078** | **22.232** | **Purifying** | **nad5** |
| **2,636** | **0.928** | **0.156** | **0.274** | **2.522** | **0.1123** | **15.392** | **Neutral** | **nad5** |
| **2,637** | **1.138** | **0.061** | **0.212** | **5.118** | **0.0237** | **11.938** | **Purifying** | **nad5** |
| **2,638** | **4.816** | **0.124** | **0.442** | **10.486** | **0.0012** | **24.844** | **Purifying** | **nad5** |
| **2,639** | **0.688** | **0.073** | **0.205** | **2.441** | **0.1182** | **11.509** | **Neutral** | **nad5** |
| **2,640** | **10.636** | **0.075** | **0.86** | **19.304** | **0** | **48.297** | **Purifying** | **nad5** |
| **2,641** | **0.895** | **0** | **0.237** | **14.419** | **0.0001** | **13.342** | **Purifying** | **nad5** |
| **2,642** | **0.488** | **0.101** | **0.148** | **1.165** | **0.2804** | **8.315** | **Neutral** | **nad5** |
| **2,643** | **3.346** | **0.058** | **1.044** | **12.174** | **0.0005** | **58.677** | **Purifying** | **nad5** |
| **2,644** | **3.585** | **0.029** | **0.341** | **9.813** | **0.0017** | **19.162** | **Purifying** | **nad5** |
| **2,645** | **2.498** | **0.042** | **0.416** | **10.552** | **0.0012** | **23.365** | **Purifying** | **nad5** |
| **2,646** | **0.515** | **0.067** | **0.086** | **1.128** | **0.2883** | **4.809** | **Neutral** | **nad5** |
| **2,647** | **0.812** | **0.064** | **0.24** | **2.372** | **0.1235** | **13.479** | **Neutral** | **nad5** |
| **2,648** | **1.552** | **0.09** | **0.311** | **4.866** | **0.0274** | **17.49** | **Purifying** | **nad5** |
| **2,649** | **1.499** | **0** | **0.5** | **13.079** | **0.0003** | **28.067** | **Purifying** | **nad5** |
| **2,650** | **1.909** | **0.061** | **0.279** | **7.577** | **0.0059** | **15.682** | **Purifying** | **nad5** |
| **2,651** | **1.494** | **0** | **0.149** | **18.573** | **0** | **8.348** | **Purifying** | **nad5** |
| **2,652** | **0.567** | **0.097** | **0.155** | **1.331** | **0.2486** | **8.721** | **Neutral** | **nad5** |
| **2,653** | **0.23** | **0.117** | **0.147** | **0.214** | **0.6437** | **8.248** | **Neutral** | **nad5** |
| **2,654** | **1.347** | **0.155** | **0.269** | **2.25** | **0.1336** | **15.138** | **Neutral** | **nad5** |
| **2,655** | **0.016** | **0.084** | **0.067** | **0.294** | **0.5875** | **3.778** | **Neutral** | **nad5** |
| **2,656** | **1.008** | **0.167** | **0.229** | **1.322** | **0.2503** | **12.859** | **Neutral** | **nad5** |
| **2,657** | **0.084** | **0.057** | **0.064** | **0.083** | **0.7738** | **3.616** | **Neutral** | **nad5** |
| **2,658** | **0.949** | **0.145** | **0.232** | **1.953** | **0.1623** | **13.012** | **Neutral** | **nad5** |
| **2,659** | **1.187** | **0.111** | **0.277** | **3.684** | **0.0549** | **15.584** | **Neutral** | **nad5** |
| **2,660** | **3.434** | **0.185** | **0.25** | **2.257** | **0.133** | **14.054** | **Neutral** | **nad5** |
| **2,661** | **0.279** | **0.13** | **0.171** | **0.199** | **0.6554** | **9.605** | **Neutral** | **nad5** |
| **2,662** | **10000** | **0** | **0.347** | **26.843** | **0** | **19.479** | **Purifying** | **nad5** |
| **2,663** | **0.16** | **0.128** | **0.136** | **0.008** | **0.9268** | **7.654** | **Neutral** | **nad5** |
| **2,664** | **0.959** | **0.08** | **0.205** | **2.717** | **0.0993** | **11.517** | **Neutral** | **nad5** |
| **2,665** | **3.385** | **0** | **0.385** | **23.892** | **0** | **21.627** | **Purifying** | **nad5** |
| **2,666** | **1.483** | **0.035** | **0.065** | **4.333** | **0.0374** | **3.674** | **Purifying** | **nad5** |
| **2,667** | **0** | **0.101** | **0.079** | **2.416** | **0.1201** | **4.451** | **Neutral** | **nad5** |
| **2,668** | **2.561** | **0.033** | **0.626** | **9.739** | **0.0018** | **35.167** | **Purifying** | **nad5** |
| **2,669** | **1.83** | **0.101** | **0.472** | **6.041** | **0.014** | **26.53** | **Purifying** | **nad5** |
| **2,670** | **2.927** | **0.028** | **0.361** | **10.83** | **0.001** | **20.292** | **Purifying** | **nad5** |
| **2,671** | **1.739** | **0.152** | **0.235** | **2.398** | **0.1215** | **13.207** | **Neutral** | **nad5** |
| **2,672** | **1.38** | **0.155** | **0.538** | **3.923** | **0.0476** | **30.236** | **Purifying** | **nad5** |
| **2,673** | **0.24** | **0.025** | **0.073** | **2.113** | **0.1461** | **4.075** | **Neutral** | **nad5** |
| **2,674** | **0.642** | **0.045** | **0.075** | **1.335** | **0.2479** | **4.206** | **Neutral** | **nad5** |
| **2,675** | **0.251** | **0.279** | **0.271** | **0.003** | **0.9566** | **15.202** | **Neutral** | **nad5** |
| **2,676** | **2.259** | **0.008** | **0.494** | **16.561** | **0** | **27.758** | **Purifying** | **nad5** |
| **2,677** | **2.289** | **0.112** | **0.432** | **7.904** | **0.0049** | **24.269** | **Purifying** | **nad5** |
| **2,678** | **2.46** | **0.034** | **0.833** | **9.496** | **0.0021** | **46.815** | **Purifying** | **nad5** |
| **2,679** | **1.593** | **0.058** | **0.366** | **7.299** | **0.0069** | **20.582** | **Purifying** | **nad5** |
| **2,680** | **0.244** | **0.121** | **0.151** | **0.246** | **0.62** | **8.504** | **Neutral** | **nad5** |
| **2,681** | **3.965** | **0.058** | **0.582** | **15.946** | **0.0001** | **32.706** | **Purifying** | **nad5** |
| **2,682** | **5.691** | **0.05** | **0.419** | **13.577** | **0.0002** | **23.563** | **Purifying** | **nad5** |
| **2,683** | **0.942** | **0.036** | **0.098** | **4.451** | **0.0349** | **5.504** | **Purifying** | **nad5** |
| **2,684** | **0.221** | **0.628** | **0.596** | **0.153** | **0.6957** | **33.476** | **Neutral** | **nad5** |
| **2,685** | **4.625** | **0.096** | **0.314** | **7.87** | **0.005** | **17.648** | **Purifying** | **nad5** |
| **2,686** | **2.51** | **0.174** | **0.412** | **5.297** | **0.0214** | **23.151** | **Purifying** | **nad5** |
| **2,687** | **1.098** | **0.165** | **0.316** | **2.722** | **0.099** | **17.772** | **Neutral** | **nad5** |
| **2,688** | **4.954** | **0.09** | **0.403** | **10.887** | **0.001** | **22.638** | **Purifying** | **nad5** |
| **2,689** | **2.415** | **0.058** | **0.498** | **9.844** | **0.0017** | **27.958** | **Purifying** | **nad5** |
| **2,690** | **3.42** | **0.083** | **0.463** | **13.891** | **0.0002** | **26.002** | **Purifying** | **nad5** |
| **2,691** | **6.253** | **0.079** | **0.329** | **9.168** | **0.0025** | **18.489** | **Purifying** | **nad5** |
| **2,692** | **6.858** | **0.041** | **0.507** | **19.761** | **0** | **28.494** | **Purifying** | **nad5** |
| **2,693** | **1.691** | **0.057** | **0.292** | **6.297** | **0.0121** | **16.431** | **Purifying** | **nad5** |
| **2,694** | **1.185** | **0.068** | **0.158** | **5.1** | **0.0239** | **8.888** | **Purifying** | **nad5** |
| **2,695** | **1.027** | **0.061** | **0.26** | **5.215** | **0.0224** | **14.632** | **Purifying** | **nad5** |
| **2,696** | **0.029** | **0.252** | **0.245** | **0.519** | **0.4711** | **13.783** | **Neutral** | **nad5** |
| **2,697** | **0.211** | **0.037** | **0.048** | **0.572** | **0.4496** | **2.708** | **Neutral** | **nad5** |
| **2,698** | **2.052** | **0.028** | **0.349** | **9.785** | **0.0018** | **19.609** | **Purifying** | **nad5** |
| **2,699** | **1.709** | **0.197** | **0.526** | **3.193** | **0.074** | **29.565** | **Neutral** | **nad5** |
| **2,700** | **1.19** | **0.053** | **0.324** | **4.865** | **0.0274** | **18.207** | **Purifying** | **nad5** |
| **2,701** | **1.749** | **0.025** | **0.267** | **8.181** | **0.0042** | **15** | **Purifying** | **nad5** |
| **2,702** | **0.691** | **0** | **0.025** | **16.375** | **0.0001** | **1.387** | **Purifying** | **nad5** |
| **2,703** | **0.727** | **0.025** | **0.041** | **3.376** | **0.0662** | **2.322** | **Neutral** | **nad5** |
| **2,704** | **0.471** | **0.066** | **0.084** | **1.143** | **0.2849** | **4.712** | **Neutral** | **nad5** |
| **2,705** | **1.098** | **0.018** | **0.23** | **7.714** | **0.0055** | **12.937** | **Purifying** | **nad5** |
| **2,706** | **0** | **0.089** | **0.063** | **2.457** | **0.117** | **3.531** | **Neutral** | **nad5** |
| **2,707** | **0.681** | **0.093** | **0.148** | **1.726** | **0.189** | **8.314** | **Neutral** | **nad5** |
| **2,708** | **0.932** | **0.086** | **0.185** | **3.704** | **0.0543** | **10.388** | **Neutral** | **nad5** |
| **2,709** | **1.134** | **0.114** | **0.219** | **2.456** | **0.1171** | **12.326** | **Neutral** | **nad5** |
| **2,710** | **1.045** | **0.026** | **0.244** | **7.178** | **0.0074** | **13.703** | **Purifying** | **nad5** |
| **2,711** | **1.363** | **0** | **0.241** | **17.191** | **0** | **13.562** | **Purifying** | **nad5** |
| **2,712** | **0.189** | **0.061** | **0.09** | **0.534** | **0.4648** | **5.076** | **Neutral** | **nad5** |
| **2,713** | **0.656** | **0.113** | **0.228** | **1.655** | **0.1983** | **12.791** | **Neutral** | **nad5** |
| **2,714** | **1.651** | **0.175** | **0.235** | **1.625** | **0.2024** | **13.218** | **Neutral** | **nad5** |
| **2,715** | **0.652** | **0.108** | **0.196** | **1.859** | **0.1728** | **11.005** | **Neutral** | **nad5** |
| **2,716** | **3.02** | **0.058** | **0.529** | **12.309** | **0.0005** | **29.722** | **Purifying** | **nad5** |
| **2,717** | **3.407** | **0.193** | **0.937** | **8.258** | **0.0041** | **52.648** | **Purifying** | **nad5** |
| **2,718** | **6.301** | **0.057** | **0.464** | **12.211** | **0.0005** | **26.043** | **Purifying** | **nad5** |
| **2,719** | **1.001** | **0.052** | **0.1** | **3.787** | **0.0517** | **5.605** | **Neutral** | **nad5** |
| **2,720** | **0** | **0.132** | **0.095** | **2.344** | **0.1258** | **5.343** | **Neutral** | **nad5** |
| **2,721** | **10.819** | **0.138** | **0.977** | **22.118** | **0** | **54.876** | **Purifying** | **nad5** |
| **2,722** | **1.164** | **0.068** | **0.157** | **5.057** | **0.0245** | **8.811** | **Purifying** | **nad5** |
| **2,723** | **2.033** | **0.099** | **0.289** | **6.003** | **0.0143** | **16.255** | **Purifying** | **nad5** |
| **2,724** | **0.814** | **0.075** | **0.233** | **3.336** | **0.0678** | **13.071** | **Neutral** | **nad5** |
| **2,725** | **4.437** | **0.041** | **0.539** | **17.979** | **0** | **30.258** | **Purifying** | **nad5** |
| **2,726** | **1.616** | **0.121** | **0.686** | **5.68** | **0.0172** | **38.564** | **Purifying** | **nad5** |
| **2,727** | **1.185** | **0.061** | **0.21** | **5.109** | **0.0238** | **11.805** | **Purifying** | **nad5** |
| **2,728** | **1.101** | **0.163** | **0.246** | **1.843** | **0.1745** | **13.83** | **Neutral** | **nad5** |
| **2,729** | **0.773** | **0.083** | **0.201** | **2.306** | **0.1288** | **11.313** | **Neutral** | **nad5** |
| **2,730** | **0** | **0** | **0** | **0** | **1** | **0** | **Invariable** | **nad5** |
| **2,731** | **0.21** | **0.095** | **0.124** | **0.148** | **0.7008** | **6.968** | **Neutral** | **nad5** |
| **2,732** | **0** | **0.209** | **0.22** | **2.151** | **0.1425** | **12.337** | **Neutral** | **nad5** |
| **2,733** | **0.758** | **0.02** | **0.123** | **6.065** | **0.0138** | **6.934** | **Purifying** | **nad5** |
| **2,734** | **0.336** | **0.162** | **0.187** | **0.17** | **0.6804** | **10.527** | **Neutral** | **nad5** |
| **2,735** | **0.962** | **0.166** | **0.203** | **1.216** | **0.2702** | **11.432** | **Neutral** | **nad5** |
| **2,736** | **0.703** | **0.016** | **0.38** | **2.897** | **0.0887** | **21.37** | **Neutral** | **nad5** |
| **2,737** | **1.323** | **0.056** | **0.107** | **3.47** | **0.0625** | **6.01** | **Neutral** | **nad5** |
| **2,738** | **1.956** | **0.127** | **0.404** | **6.833** | **0.0089** | **22.7** | **Purifying** | **nad5** |
| **2,739** | **0.088** | **0.14** | **0.132** | **0.062** | **0.8028** | **7.404** | **Neutral** | **nad5** |
| **2,740** | **0.336** | **0.01** | **0.133** | **3.196** | **0.0738** | **7.469** | **Neutral** | **nad5** |
| **2,741** | **1.648** | **0.167** | **0.222** | **1.646** | **0.1995** | **12.501** | **Neutral** | **nad5** |
| **2,742** | **0.268** | **0.196** | **0.208** | **0.023** | **0.8792** | **11.705** | **Neutral** | **nad5** |
| **2,743** | **0** | **0.054** | **0.041** | **1.667** | **0.1967** | **2.285** | **Neutral** | **nad5** |
| **2,744** | **1.84** | **0.108** | **0.261** | **5.466** | **0.0194** | **14.64** | **Purifying** | **nad5** |
| **2,745** | **0.223** | **0.043** | **0.104** | **0.93** | **0.3349** | **5.823** | **Neutral** | **nad5** |
| **2,746** | **1.135** | **0.107** | **0.27** | **4.283** | **0.0385** | **15.151** | **Purifying** | **nad5** |
| **2,747** | **0.714** | **0.03** | **0.116** | **4.438** | **0.0352** | **6.509** | **Purifying** | **nad5** |
| **2,748** | **0.331** | **0.12** | **0.153** | **0.296** | **0.5861** | **8.606** | **Neutral** | **nad5** |
| **2,749** | **10000** | **0.08** | **0.39** | **14.927** | **0.0001** | **21.922** | **Purifying** | **nad5** |
| **2,750** | **0.244** | **0.34** | **0.318** | **0.031** | **0.8597** | **17.872** | **Neutral** | **nad5** |
| **2,751** | **2.908** | **0.02** | **0.237** | **11.2** | **0.0008** | **13.29** | **Purifying** | **nad5** |
| **2,752** | **2.622** | **0** | **0.771** | **16.883** | **0** | **43.328** | **Purifying** | **nad5** |
| **2,753** | **0.95** | **0.161** | **0.275** | **2.556** | **0.1098** | **15.449** | **Neutral** | **nad5** |
| **2,754** | **0** | **0.108** | **0.087** | **2.56** | **0.1096** | **4.867** | **Neutral** | **nad5** |
| **2,755** | **0.581** | **0.033** | **0.157** | **3.858** | **0.0495** | **8.794** | **Purifying** | **nad5** |
| **2,756** | **2.232** | **0.033** | **0.622** | **10.74** | **0.001** | **34.955** | **Purifying** | **nad5** |
| **2,757** | **2.339** | **0.112** | **0.403** | **7.673** | **0.0056** | **22.647** | **Purifying** | **nad5** |
| **2,758** | **3.24** | **0.04** | **0.333** | **11.283** | **0.0008** | **18.703** | **Purifying** | **nad5** |
| **2,759** | **2.727** | **0.108** | **0.773** | **7.617** | **0.0058** | **43.449** | **Purifying** | **nad5** |
| **2,760** | **1.481** | **0.106** | **0.296** | **4.809** | **0.0283** | **16.649** | **Purifying** | **nad5** |
| **2,761** | **1.199** | **0.028** | **0.358** | **7.41** | **0.0065** | **20.116** | **Purifying** | **nad5** |
| **2,762** | **0.026** | **0.042** | **0.037** | **0.053** | **0.8179** | **2.061** | **Neutral** | **nad5** |
| **2,763** | **0.772** | **0.02** | **0.028** | **1.853** | **0.1735** | **1.592** | **Neutral** | **nad5** |
| **2,764** | **0.736** | **0.073** | **0.251** | **3.321** | **0.0684** | **14.083** | **Neutral** | **nad5** |
| **2,765** | **0.732** | **0.027** | **0.166** | **4.974** | **0.0257** | **9.348** | **Purifying** | **nad5** |
| **2,766** | **1.484** | **0.17** | **0.26** | **2.19** | **0.1389** | **14.626** | **Neutral** | **nad5** |
| **2,767** | **0.732** | **0.03** | **0.2** | **4.473** | **0.0344** | **11.238** | **Purifying** | **nad5** |
| **2,768** | **0.607** | **0.096** | **0.191** | **2.095** | **0.1478** | **10.744** | **Neutral** | **nad5** |
| **2,769** | **0.733** | **0.074** | **0.127** | **1.731** | **0.1882** | **7.16** | **Neutral** | **nad5** |
| **2,770** | **2.384** | **0.102** | **0.265** | **6.019** | **0.0142** | **14.905** | **Purifying** | **nad5** |
| **2,771** | **1.864** | **0.114** | **0.282** | **5.733** | **0.0166** | **15.856** | **Purifying** | **nad5** |
| **2,772** | **1.226** | **0.152** | **0.316** | **2.982** | **0.0842** | **17.767** | **Neutral** | **nad5** |
| **2,773** | **1.701** | **0.173** | **0.329** | **3.837** | **0.0501** | **18.495** | **Neutral** | **nad5** |
| **2,774** | **1.662** | **0.124** | **0.432** | **5.874** | **0.0154** | **24.281** | **Purifying** | **nad5** |
| **2,775** | **1.425** | **0.077** | **0.523** | **4.697** | **0.0302** | **29.407** | **Purifying** | **nad5** |
| **2,776** | **57.755** | **0.11** | **0.626** | **12.955** | **0.0003** | **35.151** | **Purifying** | **nad5** |
| **2,777** | **0.163** | **0.03** | **0.083** | **1.169** | **0.2796** | **4.679** | **Neutral** | **nad5** |
| **2,778** | **0.086** | **0.044** | **0.052** | **0.125** | **0.7236** | **2.927** | **Neutral** | **nad5** |
| **2,779** | **0.628** | **0.01** | **0.098** | **4.374** | **0.0365** | **5.531** | **Purifying** | **nad5** |
| **2,780** | **1.128** | **0.148** | **0.34** | **4.54** | **0.0331** | **19.121** | **Purifying** | **nad5** |
| **2,781** | **0.504** | **0.106** | **0.127** | **0.819** | **0.3655** | **7.124** | **Neutral** | **nad5** |
| **2,782** | **0.108** | **0.033** | **0.056** | **0.703** | **0.4016** | **3.127** | **Neutral** | **nad5** |
| **2,783** | **0.391** | **0.06** | **0.155** | **2.269** | **0.132** | **8.687** | **Neutral** | **nad5** |
| **2,784** | **0.042** | **0.049** | **0.045** | **0.015** | **0.903** | **2.556** | **Neutral** | **nad5** |
| **2,785** | **0.228** | **0.024** | **0.077** | **2.303** | **0.1291** | **4.353** | **Neutral** | **nad5** |
| **2,786** | **0.022** | **0.126** | **0.083** | **1.501** | **0.2205** | **4.66** | **Neutral** | **nad5** |
| **2,787** | **0.064** | **0.044** | **0.053** | **0.044** | **0.8338** | **2.953** | **Neutral** | **nad5** |
| **2,788** | **0.161** | **0** | **0.031** | **5.904** | **0.0151** | **1.755** | **Purifying** | **nad5** |
| **2,789** | **0.601** | **0.11** | **0.335** | **1.843** | **0.1746** | **18.828** | **Neutral** | **nad5** |
| **2,790** | **14.961** | **0.084** | **0.448** | **8.754** | **0.0031** | **25.155** | **Purifying** | **nad5** |
| **2,791** | **1.494** | **0.156** | **0.281** | **3.826** | **0.0504** | **15.801** | **Neutral** | **nad5** |
| **2,792** | **0.874** | **0.017** | **0.028** | **4.086** | **0.0432** | **1.595** | **Purifying** | **nad5** |
| **2,793** | **2.203** | **0.159** | **0.448** | **7.51** | **0.0061** | **25.158** | **Purifying** | **nad5** |
| **2,794** | **0.036** | **0.139** | **0.119** | **0.306** | **0.5802** | **6.697** | **Neutral** | **nad5** |
| **2,795** | **1.06** | **0.024** | **0.193** | **6.282** | **0.0122** | **10.855** | **Purifying** | **nad5** |
| **2,796** | **1.838** | **0.057** | **0.386** | **8.615** | **0.0033** | **21.702** | **Purifying** | **nad5** |
| **2,797** | **1.867** | **0.02** | **0.069** | **8.673** | **0.0032** | **3.85** | **Purifying** | **nad5** |
| **2,798** | **1.803** | **0.071** | **0.252** | **6.833** | **0.009** | **14.156** | **Purifying** | **nad5** |
| **2,799** | **0.561** | **0.125** | **0.156** | **0.854** | **0.3555** | **8.787** | **Neutral** | **nad5** |
| **2,800** | **0.945** | **0.03** | **0.095** | **3.768** | **0.0522** | **5.321** | **Neutral** | **nad5** |
| **2,801** | **1.677** | **0.098** | **0.553** | **5.414** | **0.02** | **31.048** | **Purifying** | **nad5** |
| **2,802** | **1.094** | **0** | **0.374** | **11.919** | **0.0006** | **21.003** | **Purifying** | **nad5** |
| **2,803** | **0.559** | **0.028** | **0.175** | **3.387** | **0.0657** | **9.825** | **Neutral** | **nad5** |
| **2,804** | **0** | **0.121** | **0.091** | **2.796** | **0.0945** | **5.115** | **Neutral** | **nad5** |
| **2,805** | **0** | **0.251** | **0.278** | **2.361** | **0.1244** | **15.593** | **Neutral** | **nad5** |
| **2,806** | **0.792** | **0.038** | **0.098** | **3.042** | **0.0811** | **5.528** | **Neutral** | **nad5** |
| **2,807** | **5.414** | **0.079** | **0.675** | **13.747** | **0.0002** | **37.943** | **Purifying** | **nad5** |
| **2,808** | **1.032** | **0** | **0.316** | **11.689** | **0.0006** | **17.743** | **Purifying** | **nad5** |
| **2,809** | **0.336** | **0.086** | **0.134** | **0.544** | **0.4609** | **7.532** | **Neutral** | **nad5** |
| **2,810** | **0.331** | **0.117** | **0.149** | **0.325** | **0.5688** | **8.382** | **Neutral** | **nad5** |
| **2,811** | **132.901** | **0.111** | **0.324** | **8.115** | **0.0044** | **18.177** | **Purifying** | **nad5** |
| **2,812** | **0.096** | **0.062** | **0.071** | **0.063** | **0.8019** | **4.009** | **Neutral** | **nad5** |
| **2,813** | **3.446** | **0.095** | **0.717** | **11.022** | **0.0009** | **40.268** | **Purifying** | **nad5** |
| **2,814** | **0.717** | **0.02** | **0.029** | **1.698** | **0.1925** | **1.613** | **Neutral** | **nad5** |
| **2,815** | **0.56** | **0.098** | **0.119** | **0.918** | **0.3381** | **6.699** | **Neutral** | **nad5** |
| **2,816** | **2.198** | **0.143** | **0.724** | **6.445** | **0.0111** | **40.658** | **Purifying** | **nad5** |
| **2,817** | **1.259** | **0.157** | **0.269** | **3.296** | **0.0695** | **15.117** | **Neutral** | **nad5** |
| **2,818** | **0.541** | **0.558** | **0.553** | **0.001** | **0.9818** | **31.051** | **Neutral** | **nad5** |
| **2,819** | **0.04** | **0.057** | **0.053** | **0.045** | **0.8323** | **2.963** | **Neutral** | **nad5** |
| **2,820** | **2.928** | **0.105** | **0.688** | **8.255** | **0.0041** | **38.664** | **Purifying** | **nad5** |
| **2,821** | **3.544** | **0.091** | **0.349** | **10.467** | **0.0012** | **19.619** | **Purifying** | **nad5** |
| **2,822** | **1.578** | **0.19** | **0.53** | **4.198** | **0.0405** | **29.796** | **Purifying** | **nad5** |
| **2,823** | **5.637** | **0.073** | **0.448** | **16.748** | **0** | **25.168** | **Purifying** | **nad5** |
| **2,824** | **0.234** | **0.023** | **0.084** | **2.314** | **0.1282** | **4.692** | **Neutral** | **nad5** |
| **2,825** | **1.276** | **0.121** | **0.421** | **3.535** | **0.0601** | **23.647** | **Neutral** | **nad5** |
| **2,826** | **0.966** | **0.11** | **0.225** | **3.311** | **0.0688** | **12.648** | **Neutral** | **nad5** |
| **2,827** | **0.52** | **0.062** | **0.079** | **1.055** | **0.3044** | **4.454** | **Neutral** | **nad5** |
| **2,828** | **0.848** | **0.143** | **0.297** | **1.928** | **0.165** | **16.663** | **Neutral** | **nad5** |
| **2,829** | **11.156** | **0.022** | **0.533** | **19.078** | **0** | **29.954** | **Purifying** | **nad5** |
| **2,830** | **0.522** | **0.041** | **0.059** | **1.56** | **0.2116** | **3.34** | **Neutral** | **nad5** |
| **2,831** | **0.495** | **0.139** | **0.16** | **0.592** | **0.4417** | **8.98** | **Neutral** | **nad5** |
| **2,832** | **0.548** | **0.12** | **0.173** | **1.034** | **0.3092** | **9.736** | **Neutral** | **nad5** |
| **2,833** | **3.733** | **0.018** | **0.319** | **15.733** | **0.0001** | **17.947** | **Purifying** | **nad5** |
| **2,834** | **0.663** | **0.092** | **0.116** | **1.244** | **0.2647** | **6.498** | **Neutral** | **nad5** |
| **2,835** | **2.48** | **0.099** | **0.551** | **7.648** | **0.0057** | **30.936** | **Purifying** | **nad5** |
| **2,836** | **0.227** | **0.026** | **0.078** | **2.196** | **0.1384** | **4.361** | **Neutral** | **nad5** |
| **2,837** | **0** | **0.138** | **0.119** | **2.025** | **0.1547** | **6.708** | **Neutral** | **nad5** |
| **2,838** | **1.056** | **0.077** | **0.212** | **3.112** | **0.0777** | **11.924** | **Neutral** | **nad5** |
| **2,839** | **6.713** | **0.096** | **0.378** | **11.613** | **0.0007** | **21.237** | **Purifying** | **nad5** |
| **2,840** | **0** | **0.064** | **0.046** | **2.196** | **0.1384** | **2.564** | **Neutral** | **nad5** |
| **2,841** | **3.712** | **0.15** | **0.267** | **3.214** | **0.073** | **14.974** | **Neutral** | **nad5** |
| **2,842** | **0.157** | **0.025** | **0.027** | **0.314** | **0.5752** | **1.518** | **Neutral** | **nad5** |
| **2,843** | **1.213** | **0.159** | **0.323** | **3.386** | **0.0658** | **18.161** | **Neutral** | **nad5** |
| **2,844** | **0.996** | **0.102** | **0.219** | **3.526** | **0.0604** | **12.316** | **Neutral** | **nad5** |
| **2,845** | **0.762** | **0.146** | **0.269** | **2.452** | **0.1174** | **15.103** | **Neutral** | **nad5** |
| **2,846** | **0.286** | **0.032** | **0.087** | **1.791** | **0.1808** | **4.893** | **Neutral** | **nad5** |
| **2,847** | **1.733** | **0.03** | **0.351** | **8.639** | **0.0033** | **19.715** | **Purifying** | **nad5** |
| **2,848** | **0.772** | **0.111** | **0.21** | **2.117** | **0.1457** | **11.79** | **Neutral** | **nad5** |
| **2,849** | **3.848** | **0.05** | **0.428** | **11.544** | **0.0007** | **24.053** | **Purifying** | **nad5** |
| **2,850** | **0** | **0.076** | **0.064** | **1.808** | **0.1787** | **3.577** | **Neutral** | **nad5** |
| **2,851** | **7.957** | **0.175** | **0.463** | **8.309** | **0.0039** | **26** | **Purifying** | **nad5** |
| **2,852** | **0.679** | **0.111** | **0.265** | **1.622** | **0.2029** | **14.872** | **Neutral** | **nad5** |
| **2,853** | **0.224** | **0.08** | **0.114** | **0.534** | **0.4651** | **6.382** | **Neutral** | **nad5** |
| **2,854** | **0.752** | **0.112** | **0.403** | **2.336** | **0.1264** | **22.648** | **Neutral** | **nad5** |
| **2,855** | **0.214** | **0.512** | **0.447** | **0.212** | **0.6455** | **25.126** | **Neutral** | **nad5** |
| **2,856** | **0.284** | **0.193** | **0.213** | **0.047** | **0.8285** | **11.969** | **Neutral** | **nad5** |
| **2,857** | **0.259** | **0.057** | **0.111** | **0.627** | **0.4283** | **6.222** | **Neutral** | **nad5** |
| **2,858** | **0** | **0.051** | **0.035** | **1.311** | **0.2523** | **1.974** | **Neutral** | **nad5** |
| **2,859** | **1.108** | **0.061** | **0.269** | **5.406** | **0.0201** | **15.125** | **Purifying** | **nad5** |
| **2,860** | **1.04** | **0.071** | **0.211** | **4.967** | **0.0258** | **11.827** | **Purifying** | **nad5** |
| **2,861** | **0.868** | **0** | **0.272** | **11.725** | **0.0006** | **15.283** | **Purifying** | **nad5** |
| **2,862** | **10000** | **0.143** | **0.43** | **10.926** | **0.0009** | **24.142** | **Purifying** | **nad5** |
| **2,863** | **0.303** | **0.091** | **0.138** | **0.396** | **0.5294** | **7.772** | **Neutral** | **nad5** |
| **2,864** | **0** | **0.091** | **0.067** | **2.59** | **0.1075** | **3.751** | **Neutral** | **nad5** |
| **2,865** | **4.5** | **0.156** | **0.43** | **5.945** | **0.0148** | **24.185** | **Purifying** | **nad5** |
| **2,866** | **0.798** | **0.154** | **0.326** | **1.728** | **0.1887** | **18.314** | **Neutral** | **nad5** |
| **2,867** | **0.633** | **0.03** | **0.166** | **3.943** | **0.0471** | **9.32** | **Purifying** | **nad5** |
| **2,868** | **0.106** | **0.068** | **0.081** | **0.072** | **0.7881** | **4.523** | **Neutral** | **nad5** |
| **2,869** | **1.303** | **0.102** | **0.312** | **3.623** | **0.057** | **17.505** | **Neutral** | **nad5** |
| **2,870** | **0.154** | **0** | **0.009** | **4.498** | **0.0339** | **0.482** | **Purifying** | **nad5** |
| **2,871** | **0.499** | **0.027** | **0.043** | **1.931** | **0.1647** | **2.424** | **Neutral** | **nad5** |
| **2,872** | **2.448** | **0.082** | **0.268** | **7.287** | **0.0069** | **15.031** | **Purifying** | **nad5** |
| **2,873** | **4.283** | **0.073** | **0.44** | **11.072** | **0.0009** | **24.741** | **Purifying** | **nad5** |
| **2,874** | **0.877** | **0.031** | **0.053** | **3.342** | **0.0676** | **2.982** | **Neutral** | **nad5** |
| **2,875** | **1.478** | **0.077** | **0.273** | **5.042** | **0.0247** | **15.325** | **Purifying** | **nad5** |
| **2,876** | **0.237** | **0.141** | **0.159** | **0.084** | **0.772** | **8.912** | **Neutral** | **nad5** |
| **2,877** | **3.018** | **0.124** | **0.275** | **5.588** | **0.0181** | **15.438** | **Purifying** | **nad5** |
| **2,878** | **3.607** | **0.072** | **0.211** | **3.832** | **0.0503** | **11.867** | **Neutral** | **nad5** |
| **2,879** | **0.246** | **0.127** | **0.156** | **0.209** | **0.6479** | **8.739** | **Neutral** | **nad5** |
| **2,880** | **0.013** | **0.132** | **0.093** | **0.606** | **0.4364** | **5.251** | **Neutral** | **nad5** |
| **2,881** | **6.034** | **0.108** | **1.157** | **11.709** | **0.0006** | **65.028** | **Purifying** | **nad5** |
| **2,882** | **2.646** | **0.108** | **0.315** | **6.6** | **0.0102** | **17.705** | **Purifying** | **nad5** |
| **2,883** | **1.319** | **0.138** | **0.367** | **2.192** | **0.1387** | **20.637** | **Neutral** | **nad5** |
| **2,884** | **0.296** | **0.125** | **0.155** | **0.276** | **0.5995** | **8.682** | **Neutral** | **nad5** |
| **2,885** | **0** | **0.102** | **0.081** | **2.515** | **0.1128** | **4.57** | **Neutral** | **nad5** |
| **2,886** | **0.726** | **0.076** | **0.171** | **2.562** | **0.1094** | **9.616** | **Neutral** | **nad5** |
| **2,887** | **0.796** | **0.052** | **0.187** | **3.536** | **0.0601** | **10.496** | **Neutral** | **nad5** |
| **2,888** | **3.066** | **0.026** | **0.572** | **14.466** | **0.0001** | **32.129** | **Purifying** | **nad5** |
| **2,889** | **2.437** | **0.12** | **0.522** | **9.261** | **0.0023** | **29.305** | **Purifying** | **nad5** |
| **2,890** | **7.968** | **0.112** | **0.577** | **12.632** | **0.0004** | **32.442** | **Purifying** | **nad5** |
| **2,891** | **0.098** | **0.035** | **0.055** | **0.45** | **0.5023** | **3.108** | **Neutral** | **nad5** |
| **2,892** | **0.997** | **0.057** | **0.352** | **4.074** | **0.0436** | **19.75** | **Purifying** | **nad5** |
| **2,893** | **0.846** | **0.158** | **0.207** | **1.285** | **0.2569** | **11.622** | **Neutral** | **nad5** |
| **2,894** | **2.402** | **0.058** | **0.186** | **7.548** | **0.006** | **10.457** | **Purifying** | **nad5** |
| **2,895** | **5.585** | **0.143** | **0.369** | **7.093** | **0.0077** | **20.709** | **Purifying** | **nad5** |
| **2,896** | **2.755** | **0.183** | **0.394** | **5.106** | **0.0238** | **22.131** | **Purifying** | **nad5** |
| **2,897** | **6.412** | **0.14** | **0.275** | **6.256** | **0.0124** | **15.46** | **Purifying** | **nad5** |
| **2,898** | **1.327** | **0.076** | **0.23** | **4.876** | **0.0272** | **12.919** | **Purifying** | **nad5** |
| **2,899** | **1.275** | **0.022** | **0.209** | **7.133** | **0.0076** | **11.767** | **Purifying** | **nad5** |
| **2,900** | **0.31** | **0.174** | **0.193** | **0.089** | **0.7658** | **10.817** | **Neutral** | **nad5** |
| **2,901** | **0.282** | **0.232** | **0.24** | **0.008** | **0.9292** | **13.494** | **Neutral** | **nad5** |
| **2,902** | **2.852** | **0.105** | **0.391** | **10.614** | **0.0011** | **21.953** | **Purifying** | **nad5** |
| **2,903** | **1.685** | **0.083** | **0.167** | **5.111** | **0.0238** | **9.357** | **Purifying** | **nad5** |
| **2,904** | **0.289** | **0.297** | **0.297** | **0** | **0.9843** | **16.688** | **Neutral** | **nad5** |
| **2,905** | **0.946** | **0.176** | **0.224** | **1.135** | **0.2867** | **12.562** | **Neutral** | **nad5** |
| **2,906** | **0.091** | **0.067** | **0.074** | **0.032** | **0.8587** | **4.142** | **Neutral** | **nad5** |
| **2,907** | **1.975** | **0.033** | **0.694** | **9.114** | **0.0025** | **38.979** | **Purifying** | **nad5** |
| **2,908** | **0.218** | **0.119** | **0.145** | **0.192** | **0.6612** | **8.132** | **Neutral** | **nad5** |
| **2,909** | **4.211** | **0.05** | **0.468** | **9.765** | **0.0018** | **26.301** | **Purifying** | **nad5** |
| **2,910** | **10000** | **0.106** | **0.626** | **18.531** | **0** | **35.195** | **Purifying** | **nad5** |
| **2,911** | **4.188** | **0.11** | **0.558** | **12.536** | **0.0004** | **31.352** | **Purifying** | **nad5** |
| **2,912** | **0.703** | **0.021** | **0.153** | **6.563** | **0.0104** | **8.592** | **Purifying** | **nad5** |
| **2,913** | **0.734** | **0** | **0.063** | **15.111** | **0.0001** | **3.554** | **Purifying** | **nad5** |
| **2,914** | **1.106** | **0.102** | **0.189** | **2.629** | **0.105** | **10.639** | **Neutral** | **nad5** |
| **2,915** | **1.205** | **0.166** | **0.331** | **2.947** | **0.086** | **18.57** | **Neutral** | **nad5** |
| **2,916** | **2.931** | **0.02** | **0.533** | **15.377** | **0.0001** | **29.956** | **Purifying** | **nad5** |
| **2,917** | **3.764** | **0.107** | **0.889** | **10.183** | **0.0014** | **49.957** | **Purifying** | **nad5** |
| **2,918** | **4.406** | **0.07** | **0.422** | **15.878** | **0.0001** | **23.717** | **Purifying** | **nad5** |
| **2,919** | **1.086** | **0.062** | **0.129** | **3.689** | **0.0548** | **7.223** | **Neutral** | **nad5** |
| **2,920** | **2.643** | **0.116** | **0.768** | **9.042** | **0.0026** | **43.168** | **Purifying** | **nad5** |
| **2,921** | **2.298** | **0.096** | **0.353** | **8.785** | **0.003** | **19.852** | **Purifying** | **nad5** |
| **2,922** | **1.422** | **0.191** | **0.563** | **4.23** | **0.0397** | **31.62** | **Purifying** | **nad5** |
| **2,923** | **1.951** | **0** | **0.32** | **19.471** | **0** | **17.96** | **Purifying** | **nad5** |
| **2,924** | **3.706** | **0.11** | **0.495** | **14.651** | **0.0001** | **27.788** | **Purifying** | **nad5** |
| **2,925** | **2.24** | **0.159** | **0.395** | **6.641** | **0.01** | **22.194** | **Purifying** | **nad5** |
| **2,926** | **1.457** | **0.033** | **0.226** | **6.782** | **0.0092** | **12.693** | **Purifying** | **nad5** |
| **2,927** | **0.966** | **0.137** | **0.181** | **1.477** | **0.2243** | **10.174** | **Neutral** | **nad5** |
| **2,928** | **2.08** | **0.117** | **0.905** | **8.313** | **0.0039** | **50.832** | **Purifying** | **nad5** |
| **2,929** | **1.339** | **0** | **0.243** | **17.262** | **0** | **13.652** | **Purifying** | **nad5** |
| **2,930** | **1.709** | **0.104** | **0.246** | **5.447** | **0.0196** | **13.827** | **Purifying** | **nad5** |
| **2,931** | **0.929** | **0.108** | **0.138** | **1.542** | **0.2143** | **7.753** | **Neutral** | **nad5** |
| **2,932** | **0.924** | **0.032** | **0.102** | **4.364** | **0.0367** | **5.724** | **Purifying** | **nad5** |
| **2,933** | **0.978** | **0.059** | **0.21** | **4.094** | **0.043** | **11.817** | **Purifying** | **nad5** |
| **2,934** | **0.275** | **0.174** | **0.187** | **0.047** | **0.8275** | **10.497** | **Neutral** | **nad5** |
| **2,935** | **3.111** | **0.141** | **0.32** | **6.75** | **0.0094** | **17.966** | **Purifying** | **nad5** |
| **2,936** | **0.547** | **0** | **0.006** | **5.116** | **0.0237** | **0.345** | **Purifying** | **nad5** |
| **2,937** | **3.23** | **0.026** | **0.392** | **12.22** | **0.0005** | **22.014** | **Purifying** | **nad5** |
| **2,938** | **1.657** | **0.112** | **0.27** | **4.846** | **0.0277** | **15.145** | **Purifying** | **nad5** |
| **2,939** | **2.575** | **0.152** | **0.253** | **2.97** | **0.0848** | **14.219** | **Neutral** | **nad5** |
| **2,940** | **0.548** | **0.02** | **0.028** | **1.338** | **0.2473** | **1.578** | **Neutral** | **nad5** |
| **2,941** | **0.597** | **0.114** | **0.231** | **1.571** | **0.21** | **12.955** | **Neutral** | **nad5** |
| **2,942** | **0** | **0.046** | **0.029** | **1.461** | **0.2268** | **1.614** | **Neutral** | **nad5** |
| **2,943** | **0.236** | **0.172** | **0.186** | **0.037** | **0.8484** | **10.461** | **Neutral** | **nad5** |
| **2,944** | **0.535** | **0.135** | **0.168** | **0.791** | **0.3739** | **9.434** | **Neutral** | **nad5** |
| **2,945** | **0.167** | **0.09** | **0.106** | **0.084** | **0.7719** | **5.962** | **Neutral** | **nad5** |
| **2,946** | **1.316** | **0.054** | **0.42** | **5.897** | **0.0152** | **23.604** | **Purifying** | **nad5** |
| **2,947** | **0.11** | **0.094** | **0.098** | **0.011** | **0.9184** | **5.505** | **Neutral** | **nad5** |
| **2,948** | **0.029** | **0.056** | **0.047** | **0.204** | **0.6513** | **2.616** | **Neutral** | **nad5** |
| **2,949** | **0.093** | **0.082** | **0.084** | **0.009** | **0.9251** | **4.732** | **Neutral** | **nad5** |
| **2,950** | **2.079** | **0.034** | **0.78** | **9.097** | **0.0026** | **43.812** | **Purifying** | **nad5** |
| **2,951** | **0.083** | **0.059** | **0.066** | **0.061** | **0.805** | **3.703** | **Neutral** | **nad5** |
| **2,952** | **0.408** | **0.046** | **0.148** | **2.381** | **0.1228** | **8.314** | **Neutral** | **nad5** |
| **2,953** | **1.08** | **0.026** | **0.251** | **7.267** | **0.007** | **14.099** | **Purifying** | **nad5** |
| **2,954** | **0.551** | **0.14** | **0.191** | **0.757** | **0.3843** | **10.722** | **Neutral** | **nad5** |
| **2,955** | **0.866** | **0.113** | **0.428** | **2.674** | **0.102** | **24.05** | **Neutral** | **nad5** |
| **2,956** | **0** | **0.04** | **0.022** | **1.725** | **0.189** | **1.224** | **Neutral** | **nad5** |
| **2,957** | **0.849** | **0.125** | **0.247** | **2.18** | **0.1398** | **13.897** | **Neutral** | **nad5** |
| **2,958** | **0.511** | **0.021** | **0.023** | **0.868** | **0.3515** | **1.268** | **Neutral** | **nad5** |
| **2,959** | **132.808** | **0.14** | **0.29** | **5.827** | **0.0158** | **16.282** | **Purifying** | **nad5** |
| **2,960** | **0.513** | **0.06** | **0.071** | **0.593** | **0.4413** | **3.967** | **Neutral** | **nad5** |
| **2,961** | **0.219** | **0.093** | **0.121** | **0.258** | **0.6112** | **6.807** | **Neutral** | **nad5** |
| **2,962** | **0** | **0.097** | **0.074** | **2.794** | **0.0946** | **4.176** | **Neutral** | **nad5** |
| **2,963** | **4.654** | **0.131** | **0.314** | **6.741** | **0.0094** | **17.651** | **Purifying** | **nad5** |
| **2,964** | **0** | **0.025** | **0.018** | **1.022** | **0.3121** | **0.989** | **Neutral** | **nad5** |
| **2,965** | **1.491** | **0.202** | **0.547** | **3.811** | **0.0509** | **30.708** | **Neutral** | **nad5** |
| **2,966** | **0.444** | **0.065** | **0.097** | **1.169** | **0.2796** | **5.453** | **Neutral** | **nad5** |
| **2,967** | **2.27** | **0.099** | **0.393** | **8.579** | **0.0034** | **22.102** | **Purifying** | **nad5** |
| **2,968** | **1.791** | **0.446** | **0.798** | **2.353** | **0.125** | **44.816** | **Neutral** | **nad5** |
| **2,969** | **1.312** | **0.13** | **0.222** | **2.481** | **0.1152** | **12.458** | **Neutral** | **nad5** |
| **2,970** | **0.783** | **0.09** | **0.197** | **2.478** | **0.1154** | **11.094** | **Neutral** | **nad5** |
| **2,971** | **1.029** | **0** | **0.193** | **14.546** | **0.0001** | **10.852** | **Purifying** | **nad5** |
| **2,972** | **0.533** | **0.11** | **0.119** | **0.807** | **0.369** | **6.664** | **Neutral** | **nad5** |
| **2,973** | **0.332** | **0.364** | **0.361** | **0.002** | **0.9638** | **20.29** | **Neutral** | **nad5** |
| **2,974** | **2.129** | **0.115** | **0.285** | **5.972** | **0.0145** | **15.997** | **Purifying** | **nad5** |
| **2,975** | **0.878** | **0.168** | **0.228** | **1.207** | **0.2719** | **12.805** | **Neutral** | **nad5** |
| **2,976** | **1.351** | **0.175** | **0.351** | **3.205** | **0.0734** | **19.729** | **Neutral** | **nad5** |
| **2,977** | **0.159** | **0** | **0.017** | **8.015** | **0.0046** | **0.963** | **Purifying** | **nad5** |
| **2,978** | **1.826** | **0.031** | **0.271** | **8.574** | **0.0034** | **15.206** | **Purifying** | **nad5** |
| **2,979** | **1.314** | **0.109** | **0.258** | **4.169** | **0.0412** | **14.517** | **Purifying** | **nad5** |
| **2,980** | **0** | **0.117** | **0.096** | **2.606** | **0.1065** | **5.397** | **Neutral** | **nad5** |
| **2,981** | **1.443** | **0.235** | **0.44** | **2.511** | **0.1131** | **24.749** | **Neutral** | **nad5** |
| **2,982** | **3.027** | **0.112** | **0.419** | **9.152** | **0.0025** | **23.549** | **Purifying** | **nad5** |
| **2,983** | **1.626** | **0.114** | **0.396** | **5.692** | **0.017** | **22.254** | **Purifying** | **nad5** |
| **2,984** | **1.684** | **0.06** | **0.384** | **7.922** | **0.0049** | **21.593** | **Purifying** | **nad5** |
| **2,985** | **0.102** | **0.044** | **0.057** | **0.398** | **0.5282** | **3.195** | **Neutral** | **nad5** |
| **2,986** | **0.277** | **0.158** | **0.175** | **0.078** | **0.7804** | **9.82** | **Neutral** | **nad5** |
| **2,987** | **3.111** | **0.158** | **0.304** | **5.017** | **0.0251** | **17.069** | **Purifying** | **nad5** |
| **2,988** | **0.111** | **0.049** | **0.071** | **0.245** | **0.6209** | **3.996** | **Neutral** | **nad5** |
| **2,989** | **1.432** | **0.186** | **0.394** | **3.353** | **0.0671** | **22.15** | **Neutral** | **nad5** |
| **2,990** | **0.704** | **0.03** | **0.044** | **1.214** | **0.2705** | **2.455** | **Neutral** | **nad5** |
| **2,991** | **3.355** | **0.099** | **0.406** | **10.098** | **0.0015** | **22.815** | **Purifying** | **nad5** |
| **2,992** | **1.601** | **0** | **0.335** | **17.11** | **0** | **18.834** | **Purifying** | **nad5** |
| **2,993** | **0.212** | **0.175** | **0.185** | **0.021** | **0.8842** | **10.397** | **Neutral** | **nad5** |
| **2,994** | **3.08** | **0** | **0.432** | **19.945** | **0** | **24.274** | **Purifying** | **nad5** |
| **2,995** | **1.144** | **0.089** | **0.296** | **4.307** | **0.0379** | **16.623** | **Purifying** | **nad5** |
| **2,996** | **10000** | **0** | **0.023** | **25.093** | **0** | **1.311** | **Purifying** | **nad5** |
| **2,997** | **2.079** | **0.127** | **0.394** | **6.621** | **0.0101** | **22.157** | **Purifying** | **nad5** |
| **2,998** | **1.648** | **0.034** | **0.453** | **7.59** | **0.0059** | **25.461** | **Purifying** | **nad5** |
| **2,999** | **2.344** | **0.024** | **0.34** | **11.179** | **0.0008** | **19.083** | **Purifying** | **nad5** |
| **3,000** | **0.051** | **0.068** | **0.063** | **0.023** | **0.8792** | **3.551** | **Neutral** | **nad5** |
| **3,001** | **5.779** | **0.12** | **0.231** | **7.464** | **0.0063** | **12.971** | **Purifying** | **nad5** |
| **3,002** | **1.284** | **0.009** | **0.252** | **10.097** | **0.0015** | **14.139** | **Purifying** | **nad5** |
| **3,003** | **0.648** | **0.122** | **0.22** | **1.708** | **0.1912** | **12.342** | **Neutral** | **nad5** |
| **3,004** | **0.732** | **0.044** | **0.184** | **3.439** | **0.0637** | **10.319** | **Neutral** | **nad5** |
| **3,005** | **0.517** | **0.106** | **0.125** | **0.821** | **0.3648** | **7.023** | **Neutral** | **nad5** |
| **3,006** | **1.173** | **0** | **0.404** | **13.55** | **0.0002** | **22.687** | **Purifying** | **nad5** |
| **3,007** | **2.264** | **0.172** | **0.526** | **6.483** | **0.0109** | **29.564** | **Purifying** | **nad5** |
| **3,008** | **2.497** | **0.088** | **0.521** | **9.46** | **0.0021** | **29.254** | **Purifying** | **nad5** |
| **3,009** | **0** | **0.02** | **0.014** | **1.136** | **0.2865** | **0.81** | **Neutral** | **nad5** |
| **3,010** | **0.232** | **0.031** | **0.102** | **1.554** | **0.2125** | **5.757** | **Neutral** | **nad5** |
| **3,011** | **3.585** | **0.245** | **0.454** | **4.328** | **0.0375** | **25.524** | **Purifying** | **nad5** |
| **3,012** | **2.55** | **0** | **0.4** | **19.378** | **0** | **22.468** | **Purifying** | **nad5** |
| **3,013** | **2.998** | **0.069** | **0.514** | **7.735** | **0.0054** | **28.896** | **Purifying** | **nad5** |
| **3,014** | **4.613** | **0.08** | **0.823** | **14.302** | **0.0002** | **46.213** | **Purifying** | **nad5** |
| **3,015** | **3.143** | **0.107** | **0.771** | **8.325** | **0.0039** | **43.316** | **Purifying** | **nad5** |
| **3,016** | **0** | **0.135** | **0.121** | **2.079** | **0.1494** | **6.799** | **Neutral** | **nad5** |
| **3,017** | **0** | **0.075** | **0.054** | **2.494** | **0.1143** | **3.015** | **Neutral** | **nad5** |
| **3,018** | **0.691** | **0.032** | **0.151** | **4.241** | **0.0395** | **8.508** | **Purifying** | **nad5** |
| **3,019** | **1.449** | **0.057** | **0.291** | **5.775** | **0.0163** | **16.327** | **Purifying** | **nad5** |
| **3,020** | **0.044** | **0.17** | **0.133** | **0.346** | **0.5565** | **7.453** | **Neutral** | **nad5** |
| **3,021** | **2.216** | **0.03** | **0.464** | **10.817** | **0.001** | **26.043** | **Purifying** | **nad5** |
| **3,022** | **0.299** | **0.186** | **0.21** | **0.07** | **0.791** | **11.772** | **Neutral** | **nad5** |
| **3,023** | **2.04** | **0.165** | **0.321** | **4.379** | **0.0364** | **18.039** | **Purifying** | **nad5** |
| **3,024** | **1.113** | **0.033** | **0.282** | **6.426** | **0.0112** | **15.816** | **Purifying** | **nad5** |
| **3,025** | **2.615** | **0.118** | **0.687** | **7.959** | **0.0048** | **38.622** | **Purifying** | **nad5** |
| **3,026** | **1.766** | **0.056** | **0.541** | **6.282** | **0.0122** | **30.392** | **Purifying** | **nad5** |
| **3,027** | **3.005** | **0.151** | **0.349** | **5.772** | **0.0163** | **19.594** | **Purifying** | **nad5** |
| **3,028** | **1.24** | **0.145** | **0.474** | **3.594** | **0.058** | **26.613** | **Neutral** | **nad5** |
| **3,029** | **0.503** | **0.082** | **0.1** | **1.029** | **0.3103** | **5.607** | **Neutral** | **nad5** |
| **3,030** | **2.017** | **0.106** | **0.374** | **7.876** | **0.005** | **21.039** | **Purifying** | **nad5** |
| **3,031** | **1.569** | **0.083** | **0.489** | **5.1** | **0.0239** | **27.488** | **Purifying** | **nad5** |
| **3,032** | **1.528** | **0.802** | **0.958** | **0.415** | **0.5194** | **53.817** | **Neutral** | **nad5** |
| **3,033** | **1.825** | **0.098** | **0.372** | **8.107** | **0.0044** | **20.878** | **Purifying** | **nad5** |
| **3,034** | **1.368** | **0.073** | **0.238** | **4.55** | **0.0329** | **13.353** | **Purifying** | **nad5** |
| **3,035** | **6.414** | **0.361** | **1.084** | **8.421** | **0.0037** | **60.89** | **Purifying** | **nad5** |
| **3,036** | **0.809** | **0.034** | **0.288** | **5.083** | **0.0242** | **16.163** | **Purifying** | **nad5** |
| **3,037** | **0.674** | **0.052** | **0.124** | **3.444** | **0.0635** | **6.97** | **Neutral** | **nad5** |
| **3,038** | **2.149** | **0** | **0.549** | **16.315** | **0.0001** | **30.855** | **Purifying** | **nad5** |
| **3,039** | **3.267** | **0.055** | **0.682** | **11.37** | **0.0007** | **38.341** | **Purifying** | **nad5** |
| **3,040** | **0.98** | **0.235** | **0.554** | **1.036** | **0.3088** | **31.15** | **Neutral** | **nad5** |
| **3,041** | **0.385** | **0.142** | **0.162** | **0.424** | **0.5148** | **9.123** | **Neutral** | **nad5** |
| **3,042** | **0.827** | **0.101** | **0.14** | **1.635** | **0.2011** | **7.852** | **Neutral** | **nad5** |
| **3,043** | **3.948** | **0.151** | **0.252** | **2.924** | **0.0873** | **14.161** | **Neutral** | **nad5** |
| **3,044** | **0.417** | **0.061** | **0.192** | **1.646** | **0.1995** | **10.779** | **Neutral** | **nad5** |
| **3,045** | **1.427** | **0.104** | **0.226** | **4.447** | **0.035** | **12.726** | **Purifying** | **nad5** |
| **3,046** | **1.155** | **0.111** | **0.291** | **4.235** | **0.0396** | **16.338** | **Purifying** | **nad5** |
| **3,047** | **2.42** | **0** | **0.655** | **16.411** | **0.0001** | **36.785** | **Purifying** | **nad5** |
| **3,048** | **0** | **0.07** | **0.053** | **2.101** | **0.1472** | **2.971** | **Neutral** | **nad5** |
| **3,049** | **19.152** | **0.106** | **0.404** | **13.944** | **0.0002** | **22.703** | **Purifying** | **nad5** |
| **3,050** | **0.278** | **0.18** | **0.192** | **0.042** | **0.8367** | **10.766** | **Neutral** | **nad5** |
| **3,051** | **1.231** | **0.079** | **0.098** | **1.717** | **0.1901** | **5.523** | **Neutral** | **nad5** |
| **3,052** | **1.306** | **0.079** | **0.292** | **4.301** | **0.0381** | **16.392** | **Purifying** | **nad5** |
| **3,053** | **0.353** | **0.082** | **0.128** | **0.487** | **0.4851** | **7.218** | **Neutral** | **nad5** |
| **3,054** | **2.333** | **0.245** | **0.733** | **5.17** | **0.023** | **41.174** | **Purifying** | **nad5** |
| **3,055** | **0** | **0.102** | **0.081** | **2.515** | **0.1128** | **4.57** | **Neutral** | **nad5** |
| **3,056** | **0.817** | **0.061** | **0.278** | **4** | **0.0455** | **15.618** | **Purifying** | **nad5** |
| **3,057** | **0.302** | **0.265** | **0.271** | **0.004** | **0.9496** | **15.227** | **Neutral** | **nad5** |
| **3,058** | **1.157** | **0.133** | **0.429** | **4.142** | **0.0418** | **24.131** | **Purifying** | **nad5** |
| **3,059** | **1.782** | **0.691** | **0.911** | **0.909** | **0.3403** | **51.174** | **Neutral** | **nad5** |
| **3,060** | **0.613** | **0.822** | **0.769** | **0.066** | **0.7969** | **43.183** | **Neutral** | **nad5** |
| **3,061** | **37.307** | **0.082** | **0.65** | **14.603** | **0.0001** | **36.532** | **Purifying** | **nad5** |
| **3,062** | **0.958** | **0.042** | **0.073** | **3.018** | **0.0823** | **4.099** | **Neutral** | **nad5** |
| **3,063** | **1.992** | **0.03** | **0.22** | **8.035** | **0.0046** | **12.348** | **Purifying** | **nad5** |
| **3,064** | **0.307** | **0.093** | **0.135** | **0.731** | **0.3927** | **7.558** | **Neutral** | **nad5** |
| **3,065** | **9.654** | **0.158** | **0.303** | **6.777** | **0.0092** | **17.042** | **Purifying** | **nad5** |
| **3,066** | **0.898** | **0.186** | **0.208** | **0.24** | **0.6245** | **11.693** | **Neutral** | **nad5** |
| **3,067** | **2.617** | **0.082** | **0.677** | **7.88** | **0.005** | **38.036** | **Purifying** | **nad5** |
| **3,068** | **3.746** | **0.042** | **0.36** | **12.181** | **0.0005** | **20.248** | **Purifying** | **nad5** |
| **3,069** | **2496.393** | **0.075** | **0.405** | **12.863** | **0.0003** | **22.75** | **Purifying** | **nad5** |
| **3,070** | **0** | **0.079** | **0.057** | **2.349** | **0.1253** | **3.184** | **Neutral** | **nad5** |
| **3,071** | **3.677** | **0.153** | **0.505** | **8.365** | **0.0038** | **28.365** | **Purifying** | **nad5** |
| **3,072** | **0.916** | **0.028** | **0.058** | **4.914** | **0.0266** | **3.275** | **Purifying** | **nad5** |
| **3,073** | **0.965** | **0.017** | **0.107** | **7.154** | **0.0075** | **6.019** | **Purifying** | **nad5** |
| **3,074** | **0.617** | **0.33** | **0.395** | **0.181** | **0.6704** | **22.182** | **Neutral** | **nad5** |
| **3,075** | **0.938** | **0.031** | **0.053** | **3.384** | **0.0658** | **2.98** | **Neutral** | **nad5** |
| **3,076** | **0.915** | **0.028** | **0.058** | **5.744** | **0.0165** | **3.244** | **Purifying** | **nad5** |
| **3,077** | **2.233** | **0.246** | **0.663** | **4.388** | **0.0362** | **37.258** | **Purifying** | **nad5** |
| **3,078** | **0.723** | **0.331** | **0.372** | **0.25** | **0.6171** | **20.875** | **Neutral** | **nad5** |
| **3,079** | **3.146** | **0.033** | **0.543** | **11.656** | **0.0006** | **30.492** | **Purifying** | **nad5** |
| **3,080** | **3.886** | **0.018** | **0.039** | **7.212** | **0.0072** | **2.168** | **Purifying** | **nad5** |
| **3,081** | **2.217** | **0.173** | **0.534** | **6.248** | **0.0124** | **29.975** | **Purifying** | **nad5** |
| **3,082** | **0.277** | **0.09** | **0.132** | **0.315** | **0.5748** | **7.427** | **Neutral** | **nad5** |
| **3,083** | **0.898** | **0.138** | **0.289** | **2.338** | **0.1262** | **16.265** | **Neutral** | **nad5** |
| **3,084** | **1.957** | **0.22** | **0.569** | **4.54** | **0.0331** | **31.962** | **Purifying** | **nad5** |
| **3,085** | **2.423** | **0.173** | **0.68** | **7.786** | **0.0053** | **38.221** | **Purifying** | **nad5** |
| **3,086** | **0.555** | **0.041** | **0.077** | **1.699** | **0.1924** | **4.351** | **Neutral** | **nad5** |
| **3,087** | **0.251** | **0.204** | **0.211** | **0.01** | **0.9215** | **11.877** | **Neutral** | **nad5** |
| **3,088** | **1.016** | **0** | **0.012** | **11.621** | **0.0007** | **0.697** | **Purifying** | **nad5** |
| **3,089** | **1.77** | **0.112** | **0.396** | **6.548** | **0.0105** | **22.262** | **Purifying** | **nad5** |
| **3,090** | **0.086** | **0.097** | **0.094** | **0.008** | **0.93** | **5.264** | **Neutral** | **nad5** |
| **3,091** | **1.057** | **0.017** | **0.045** | **5.754** | **0.0165** | **2.508** | **Purifying** | **nad5** |
| **3,092** | **0** | **0.152** | **0.136** | **1.84** | **0.175** | **7.631** | **Neutral** | **nad5** |
| **3,093** | **0.085** | **0.278** | **0.258** | **0.244** | **0.6212** | **14.473** | **Neutral** | **nad5** |
| **3,094** | **5.577** | **0.199** | **0.83** | **10.843** | **0.001** | **46.63** | **Purifying** | **nad5** |
| **3,095** | **0.034** | **0.087** | **0.07** | **0.35** | **0.5544** | **3.953** | **Neutral** | **nad5** |
| **3,096** | **1.478** | **0.19** | **0.314** | **1.917** | **0.1662** | **17.663** | **Neutral** | **nad5** |
| **3,097** | **0.637** | **0** | **0.077** | **13.225** | **0.0003** | **4.339** | **Purifying** | **nad5** |
| **3,098** | **2.019** | **0.061** | **0.233** | **5.92** | **0.015** | **13.076** | **Purifying** | **nad5** |
| **3,099** | **2.464** | **0.167** | **0.748** | **6.612** | **0.0101** | **42.028** | **Purifying** | **nad5** |
| **3,100** | **0.191** | **0.052** | **0.055** | **0.228** | **0.6329** | **3.1** | **Neutral** | **nad5** |
| **3,101** | **0.349** | **0.088** | **0.137** | **0.474** | **0.4913** | **7.687** | **Neutral** | **nad5** |
| **3,102** | **0.51** | **0.155** | **0.181** | **0.548** | **0.4589** | **10.163** | **Neutral** | **nad5** |
| **3,103** | **0.218** | **0.087** | **0.112** | **0.233** | **0.6293** | **6.299** | **Neutral** | **nad5** |
| **3,104** | **0.21** | **0.149** | **0.166** | **0.046** | **0.8303** | **9.34** | **Neutral** | **nad5** |
| **3,105** | **0** | **0.044** | **0.032** | **0.917** | **0.3382** | **1.804** | **Neutral** | **nad5** |
| **3,106** | **0.528** | **0.058** | **0.105** | **1.923** | **0.1655** | **5.898** | **Neutral** | **nad5** |
| **3,107** | **0.854** | **0.074** | **0.262** | **3.655** | **0.0559** | **14.715** | **Neutral** | **nad5** |
| **3,108** | **0.706** | **0.049** | **0.308** | **3.058** | **0.0803** | **17.328** | **Neutral** | **nad5** |
| **3,109** | **0.96** | **0.06** | **0.235** | **2.741** | **0.0978** | **13.211** | **Neutral** | **nad5** |
| **3,110** | **1.219** | **0** | **0.387** | **13.59** | **0.0002** | **21.738** | **Purifying** | **nad5** |
| **3,111** | **0** | **0.133** | **0.119** | **2.534** | **0.1114** | **6.71** | **Neutral** | **nad5** |
| **3,112** | **0.302** | **0.399** | **0.382** | **0.023** | **0.8792** | **21.455** | **Neutral** | **nad5** |
| **3,113** | **9.817** | **0.074** | **0.822** | **19.366** | **0** | **46.159** | **Purifying** | **nad5** |
| **3,114** | **6.14** | **0.033** | **1.216** | **17.124** | **0** | **68.339** | **Purifying** | **nad5** |
| **3,115** | **1.545** | **0.088** | **0.363** | **5.999** | **0.0143** | **20.395** | **Purifying** | **nad5** |
| **3,116** | **2.464** | **0** | **0.426** | **21.484** | **0** | **23.928** | **Purifying** | **nad5** |
| **3,117** | **1.922** | **0.116** | **0.307** | **5.311** | **0.0212** | **17.222** | **Purifying** | **nad5** |
| **3,118** | **0.233** | **0.02** | **0.063** | **2.953** | **0.0857** | **3.536** | **Neutral** | **nad5** |
| **3,119** | **1.692** | **0.116** | **0.223** | **5.01** | **0.0252** | **12.549** | **Purifying** | **nad5** |
| **3,120** | **1.099** | **0.078** | **0.211** | **2.793** | **0.0947** | **11.859** | **Neutral** | **nad5** |
| **3,121** | **0** | **0.01** | **0.007** | **0.589** | **0.443** | **0.384** | **Neutral** | **nad5** |
| **3,122** | **0** | **0.138** | **0.119** | **2.025** | **0.1547** | **6.708** | **Neutral** | **nad5** |
| **3,123** | **0.285** | **0.206** | **0.218** | **0.031** | **0.8594** | **12.237** | **Neutral** | **nad5** |
| **3,124** | **2.303** | **0.094** | **0.375** | **10.137** | **0.0015** | **21.068** | **Purifying** | **nad5** |
| **3,125** | **0.241** | **0.52** | **0.441** | **0.194** | **0.6596** | **24.794** | **Neutral** | **nad5** |
| **3,126** | **0.758** | **0.091** | **0.165** | **1.568** | **0.2105** | **9.294** | **Neutral** | **nad5** |
| **3,127** | **3.369** | **0.03** | **0.098** | **4.188** | **0.0407** | **5.481** | **Purifying** | **nad5** |
| **3,128** | **1.203** | **0** | **0.238** | **16.489** | **0** | **13.37** | **Purifying** | **nad5** |
| **3,129** | **1.019** | **0** | **0.029** | **12.652** | **0.0004** | **1.625** | **Purifying** | **nad5** |
| **3,130** | **2.196** | **0.068** | **0.481** | **7.037** | **0.008** | **27.047** | **Purifying** | **nad5** |
| **3,131** | **2.212** | **0.115** | **0.31** | **5.604** | **0.0179** | **17.393** | **Purifying** | **nad5** |
| **3,132** | **0.577** | **0.218** | **0.281** | **0.44** | **0.5072** | **15.801** | **Neutral** | **nad5** |
| **3,133** | **3.31** | **0.031** | **0.292** | **10.067** | **0.0015** | **16.406** | **Purifying** | **nad5** |
| **3,134** | **1.043** | **0** | **0.03** | **13.195** | **0.0003** | **1.702** | **Purifying** | **nad5** |
| **3,135** | **1.33** | **0** | **0.323** | **12.964** | **0.0003** | **18.157** | **Purifying** | **nad5** |
| **3,136** | **2.723** | **0.11** | **0.388** | **8.336** | **0.0039** | **21.785** | **Purifying** | **nad5** |
| **3,137** | **1.257** | **0.137** | **0.445** | **4.271** | **0.0388** | **25** | **Purifying** | **nad5** |
| **3,138** | **2.067** | **0.094** | **0.271** | **6.123** | **0.0133** | **15.203** | **Purifying** | **nad5** |
| **3,139** | **0.535** | **0.072** | **0.162** | **1.274** | **0.259** | **9.082** | **Neutral** | **nad5** |
| **3,140** | **0** | **0.138** | **0.118** | **2.625** | **0.1052** | **6.639** | **Neutral** | **nad5** |
| **3,141** | **1.515** | **0.09** | **0.277** | **5.142** | **0.0234** | **15.545** | **Purifying** | **nad5** |
| **3,142** | **0.025** | **0.037** | **0.032** | **0.054** | **0.8167** | **1.801** | **Neutral** | **nad5** |
| **3,143** | **0.084** | **0.024** | **0.042** | **0.735** | **0.3912** | **2.35** | **Neutral** | **nad5** |
| **3,144** | **2.864** | **0.089** | **0.417** | **9.794** | **0.0018** | **23.427** | **Purifying** | **nad5** |
| **3,145** | **0.221** | **0.06** | **0.104** | **0.902** | **0.3422** | **5.861** | **Neutral** | **nad5** |
| **3,146** | **0.741** | **0.114** | **0.255** | **2.319** | **0.1278** | **14.344** | **Neutral** | **nad5** |
| **3,147** | **486.064** | **0.088** | **0.104** | **0.655** | **0.4184** | **5.867** | **Neutral** | **nad6** |
| **3,148** | **0** | **0.05** | **0.021** | **2.658** | **0.103** | **1.183** | **Neutral** | **nad6** |
| **3,149** | **0.433** | **0.125** | **0.134** | **0.484** | **0.4868** | **7.526** | **Neutral** | **nad6** |
| **3,150** | **0** | **0.075** | **0.041** | **4.214** | **0.0401** | **2.317** | **Diversifying** | **nad6** |
| **3,151** | **10000** | **0.089** | **0.111** | **0.849** | **0.357** | **6.263** | **Neutral** | **nad6** |
| **3,152** | **0.083** | **0.045** | **0.063** | **0.168** | **0.6817** | **3.527** | **Neutral** | **nad6** |
| **3,153** | **0.153** | **0.029** | **0.031** | **0.735** | **0.3911** | **1.766** | **Neutral** | **nad6** |
| **3,154** | **0** | **0.073** | **0.042** | **3.882** | **0.0488** | **2.369** | **Diversifying** | **nad6** |
| **3,155** | **2.266** | **0.117** | **0.682** | **7.368** | **0.0066** | **38.339** | **Purifying** | **nad6** |
| **3,156** | **0** | **0.039** | **0.033** | **1.663** | **0.1972** | **1.843** | **Neutral** | **nad6** |
| **3,157** | **0.419** | **0** | **0.008** | **6.113** | **0.0134** | **0.449** | **Purifying** | **nad6** |
| **3,158** | **1.595** | **0** | **0.444** | **13.841** | **0.0002** | **24.958** | **Purifying** | **nad6** |
| **3,159** | **0.709** | **0** | **0.205** | **13.561** | **0.0002** | **11.497** | **Purifying** | **nad6** |
| **3,160** | **0.918** | **0.068** | **0.284** | **3.105** | **0.0781** | **15.957** | **Neutral** | **nad6** |
| **3,161** | **0.397** | **0.027** | **0.055** | **2.11** | **0.1463** | **3.083** | **Neutral** | **nad6** |
| **3,162** | **1.653** | **0.114** | **0.502** | **5.874** | **0.0154** | **28.184** | **Purifying** | **nad6** |
| **3,163** | **0.121** | **0.028** | **0.06** | **0.571** | **0.4499** | **3.391** | **Neutral** | **nad6** |
| **3,164** | **0.934** | **0.093** | **0.186** | **3.805** | **0.0511** | **10.433** | **Neutral** | **nad6** |
| **3,165** | **0.976** | **0.028** | **0.262** | **5.57** | **0.0183** | **14.715** | **Purifying** | **nad6** |
| **3,166** | **0** | **0.093** | **0.058** | **4.465** | **0.0346** | **3.248** | **Diversifying** | **nad6** |
| **3,167** | **0.488** | **0.129** | **0.209** | **1.096** | **0.2952** | **11.749** | **Neutral** | **nad6** |
| **3,168** | **2.224** | **0.103** | **0.316** | **6.182** | **0.0129** | **17.782** | **Purifying** | **nad6** |
| **3,169** | **1.111** | **0.034** | **0.29** | **6.274** | **0.0123** | **16.281** | **Purifying** | **nad6** |
| **3,170** | **1.397** | **0.091** | **0.338** | **6.135** | **0.0133** | **18.969** | **Purifying** | **nad6** |
| **3,171** | **0.588** | **0.142** | **0.223** | **1.132** | **0.2874** | **12.521** | **Neutral** | **nad6** |
| **3,172** | **3.067** | **0.023** | **0.145** | **11.492** | **0.0007** | **8.154** | **Purifying** | **nad6** |
| **3,173** | **10000** | **0.106** | **0.41** | **10.942** | **0.0009** | **23.024** | **Purifying** | **nad6** |
| **3,174** | **0.291** | **0.045** | **0.11** | **1.306** | **0.2532** | **6.207** | **Neutral** | **nad6** |
| **3,175** | **0.072** | **0.03** | **0.047** | **0.357** | **0.55** | **2.662** | **Neutral** | **nad6** |
| **3,176** | **0.78** | **0.032** | **0.328** | **5.837** | **0.0157** | **18.41** | **Purifying** | **nad6** |
| **3,177** | **0.818** | **0.103** | **0.255** | **4.264** | **0.0389** | **14.331** | **Purifying** | **nad6** |
| **3,178** | **0.833** | **0.044** | **0.319** | **5.565** | **0.0183** | **17.906** | **Purifying** | **nad6** |
| **3,179** | **1.073** | **0.056** | **0.322** | **6.183** | **0.0129** | **18.071** | **Purifying** | **nad6** |
| **3,180** | **0.143** | **0** | **0.026** | **7.513** | **0.0061** | **1.452** | **Purifying** | **nad6** |
| **3,181** | **0.23** | **0.048** | **0.055** | **0.414** | **0.5197** | **3.115** | **Neutral** | **nad6** |
| **3,182** | **1.532** | **0.087** | **0.352** | **7.253** | **0.0071** | **19.777** | **Purifying** | **nad6** |
| **3,183** | **0.157** | **0.101** | **0.116** | **0.134** | **0.7138** | **6.504** | **Neutral** | **nad6** |
| **3,184** | **1.294** | **0.102** | **0.6** | **5.614** | **0.0178** | **33.735** | **Purifying** | **nad6** |
| **3,185** | **0** | **0.053** | **0.035** | **3.074** | **0.0796** | **1.965** | **Neutral** | **nad6** |
| **3,186** | **0** | **0.068** | **0.041** | **3.667** | **0.0555** | **2.311** | **Neutral** | **nad6** |
| **3,187** | **0.573** | **0** | **0.022** | **14.235** | **0.0002** | **1.233** | **Purifying** | **nad6** |
| **3,188** | **0.4** | **0.029** | **0.068** | **2.356** | **0.1248** | **3.802** | **Neutral** | **nad6** |
| **3,189** | **1.468** | **0.094** | **0.649** | **6.199** | **0.0128** | **36.482** | **Purifying** | **nad6** |
| **3,190** | **1.604** | **0.058** | **0.443** | **8.407** | **0.0037** | **24.869** | **Purifying** | **nad6** |
| **3,191** | **0.229** | **0.026** | **0.098** | **1.974** | **0.1601** | **5.501** | **Neutral** | **nad6** |
| **3,192** | **1.134** | **0.155** | **0.321** | **3.017** | **0.0824** | **18.052** | **Neutral** | **nad6** |
| **3,193** | **0.883** | **0.099** | **0.28** | **4.568** | **0.0326** | **15.713** | **Purifying** | **nad6** |
| **3,194** | **1.078** | **0.04** | **0.22** | **5.788** | **0.0161** | **12.382** | **Purifying** | **nad6** |
| **3,195** | **0.801** | **0.051** | **0.198** | **3.811** | **0.0509** | **11.138** | **Neutral** | **nad6** |
| **3,196** | **0.618** | **0.023** | **0.118** | **4.519** | **0.0335** | **6.618** | **Purifying** | **nad6** |
| **3,197** | **0.77** | **0.105** | **0.295** | **3.313** | **0.0687** | **16.602** | **Neutral** | **nad6** |
| **3,198** | **2.811** | **0.031** | **0.555** | **12.593** | **0.0004** | **31.17** | **Purifying** | **nad6** |
| **3,199** | **0.132** | **0** | **0.027** | **6.064** | **0.0138** | **1.521** | **Purifying** | **nad6** |
| **3,200** | **0.055** | **0.024** | **0.037** | **0.293** | **0.5883** | **2.059** | **Neutral** | **nad6** |
| **3,201** | **1.261** | **0.117** | **0.457** | **4.281** | **0.0385** | **25.664** | **Purifying** | **nad6** |
| **3,202** | **0** | **0.163** | **0.142** | **1.451** | **0.2283** | **7.952** | **Neutral** | **nad6** |
| **3,203** | **0.94** | **0.034** | **0.365** | **6.488** | **0.0109** | **20.51** | **Purifying** | **nad6** |
| **3,204** | **0.06** | **0.068** | **0.067** | **0.002** | **0.9634** | **3.762** | **Neutral** | **nad6** |
| **3,205** | **0.546** | **0.135** | **0.211** | **1.102** | **0.2938** | **11.86** | **Neutral** | **nad6** |
| **3,206** | **0.737** | **0.173** | **0.315** | **1.738** | **0.1874** | **17.692** | **Neutral** | **nad6** |
| **3,207** | **0.483** | **0.078** | **0.19** | **2.796** | **0.0945** | **10.674** | **Neutral** | **nad6** |
| **3,208** | **0.168** | **0.101** | **0.118** | **0.172** | **0.6784** | **6.642** | **Neutral** | **nad6** |
| **3,209** | **1.028** | **0.064** | **0.081** | **0.802** | **0.3705** | **4.533** | **Neutral** | **nad6** |
| **3,210** | **1.956** | **0.164** | **0.658** | **5.926** | **0.0149** | **36.972** | **Purifying** | **nad6** |
| **3,211** | **0.121** | **0.028** | **0.06** | **0.571** | **0.4499** | **3.391** | **Neutral** | **nad6** |
| **3,212** | **0.442** | **0.1** | **0.21** | **1.825** | **0.1768** | **11.776** | **Neutral** | **nad6** |
| **3,213** | **0** | **0.033** | **0.016** | **2.029** | **0.1543** | **0.882** | **Neutral** | **nad6** |
| **3,214** | **1.051** | **0.024** | **0.266** | **7.867** | **0.005** | **14.919** | **Purifying** | **nad6** |
| **3,215** | **4.583** | **0.029** | **0.443** | **11.737** | **0.0006** | **24.907** | **Purifying** | **nad6** |
| **3,216** | **0.737** | **0** | **0.054** | **13.968** | **0.0002** | **3.036** | **Purifying** | **nad6** |
| **3,217** | **0.506** | **0.058** | **0.092** | **1.589** | **0.2075** | **5.148** | **Neutral** | **nad6** |
| **3,218** | **0.116** | **0.023** | **0.048** | **0.961** | **0.3269** | **2.68** | **Neutral** | **nad6** |
| **3,219** | **0.472** | **0.069** | **0.184** | **1.37** | **0.2418** | **10.323** | **Neutral** | **nad6** |
| **3,220** | **0.614** | **0.139** | **0.25** | **1.992** | **0.1581** | **14.07** | **Neutral** | **nad6** |
| **3,221** | **0.144** | **0.06** | **0.08** | **0.375** | **0.5403** | **4.472** | **Neutral** | **nad6** |
| **3,222** | **0.621** | **0.056** | **0.187** | **3.268** | **0.0706** | **10.482** | **Neutral** | **nad6** |
| **3,223** | **0** | **0.236** | **0.135** | **0.486** | **0.4857** | **7.584** | **Neutral** | **nad6** |
| **3,224** | **2.199** | **0.045** | **0.288** | **7.123** | **0.0076** | **16.206** | **Purifying** | **nad6** |
| **3,225** | **1.083** | **0.109** | **0.361** | **4.48** | **0.0343** | **20.279** | **Purifying** | **nad6** |
| **3,226** | **0** | **0.08** | **0.065** | **5.056** | **0.0245** | **3.64** | **Diversifying** | **nad6** |
| **3,227** | **0** | **0.02** | **0.012** | **1.762** | **0.1843** | **0.669** | **Neutral** | **nad6** |
| **3,228** | **0.797** | **0.049** | **0.303** | **4.266** | **0.0389** | **17.043** | **Purifying** | **nad6** |
| **3,229** | **0.496** | **0** | **0.04** | **17.225** | **0** | **2.249** | **Purifying** | **nad6** |
| **3,230** | **2.204** | **0.116** | **0.326** | **7.864** | **0.005** | **18.321** | **Purifying** | **nad6** |
| **3,231** | **0** | **0.133** | **0.093** | **1.221** | **0.2693** | **5.203** | **Neutral** | **nad6** |
| **3,232** | **0.852** | **0.14** | **0.3** | **2.518** | **0.1125** | **16.871** | **Neutral** | **nad6** |
| **3,233** | **1.384** | **0.028** | **0.368** | **8.006** | **0.0047** | **20.654** | **Purifying** | **nad6** |
| **3,234** | **0** | **0.193** | **0.124** | **0.976** | **0.3232** | **6.978** | **Neutral** | **nad6** |
| **3,235** | **0** | **0.163** | **0.142** | **1.451** | **0.2283** | **7.952** | **Neutral** | **nad6** |
| **3,236** | **0.303** | **0.134** | **0.168** | **0.397** | **0.5286** | **9.427** | **Neutral** | **nad6** |
| **3,237** | **0.479** | **0.144** | **0.157** | **0.191** | **0.6617** | **8.844** | **Neutral** | **nad6** |
| **3,238** | **0** | **0.03** | **0.018** | **0.717** | **0.3973** | **0.99** | **Neutral** | **nad6** |
| **3,239** | **0.03** | **0.079** | **0.083** | **0.107** | **0.7437** | **4.643** | **Neutral** | **nad6** |
| **3,240** | **0** | **0.196** | **0.151** | **0.845** | **0.3579** | **8.502** | **Neutral** | **nad6** |
| **3,241** | **0.007** | **0.154** | **0.162** | **1.199** | **0.2736** | **9.125** | **Neutral** | **nad6** |
| **3,242** | **1.187** | **0.733** | **0.883** | **0.319** | **0.5721** | **49.601** | **Neutral** | **nad6** |
| **3,243** | **0.243** | **0.107** | **0.144** | **0.416** | **0.5191** | **8.067** | **Neutral** | **nad6** |
| **3,244** | **0.491** | **0.029** | **0.06** | **2.565** | **0.1092** | **3.366** | **Neutral** | **nad6** |
| **3,245** | **0.25** | **0.345** | **0.31** | **0.059** | **0.8086** | **17.393** | **Neutral** | **nad6** |
| **3,246** | **0.123** | **0.029** | **0.032** | **0.577** | **0.4475** | **1.794** | **Neutral** | **nad6** |
| **3,247** | **0.163** | **0.059** | **0.09** | **0.565** | **0.4524** | **5.035** | **Neutral** | **nad6** |
| **3,248** | **0.293** | **0.089** | **0.132** | **0.717** | **0.3971** | **7.432** | **Neutral** | **nad6** |
| **3,249** | **0.464** | **0.104** | **0.198** | **1.949** | **0.1627** | **11.148** | **Neutral** | **nad6** |
| **3,250** | **2.236** | **0.059** | **0.86** | **9.839** | **0.0017** | **48.306** | **Purifying** | **nad6** |
| **3,251** | **0.539** | **0.111** | **0.181** | **1.261** | **0.2615** | **10.187** | **Neutral** | **nad6** |
| **3,252** | **0.263** | **0.021** | **0.068** | **2.632** | **0.1047** | **3.823** | **Neutral** | **nad6** |
| **3,253** | **0.689** | **0.027** | **0.212** | **5.762** | **0.0164** | **11.899** | **Purifying** | **nad6** |
| **3,254** | **1.243** | **0.154** | **0.327** | **3.288** | **0.0698** | **18.362** | **Neutral** | **nad6** |
| **3,255** | **0.619** | **0.021** | **0.026** | **4.102** | **0.0428** | **1.439** | **Purifying** | **nad6** |
| **3,256** | **1.099** | **0.068** | **0.203** | **5.261** | **0.0218** | **11.405** | **Purifying** | **nad6** |
| **3,257** | **0.116** | **0.03** | **0.059** | **0.627** | **0.4284** | **3.294** | **Neutral** | **nad6** |
| **3,258** | **2.014** | **0.576** | **0.944** | **1.719** | **0.1899** | **53.024** | **Neutral** | **nad6** |
| **3,259** | **1.478** | **0.115** | **0.295** | **4.675** | **0.0306** | **16.547** | **Purifying** | **nad6** |
| **3,260** | **0.235** | **0.148** | **0.173** | **0.132** | **0.7167** | **9.697** | **Neutral** | **nad6** |
| **3,261** | **0.009** | **0.13** | **0.076** | **2.688** | **0.1011** | **4.25** | **Neutral** | **nad6** |
| **3,262** | **0.395** | **0.07** | **0.148** | **1.216** | **0.2701** | **8.295** | **Neutral** | **nad6** |
| **3,263** | **5.694** | **0.138** | **1.364** | **14.122** | **0.0002** | **76.639** | **Purifying** | **nad6** |
| **3,264** | **0.235** | **0** | **0.022** | **12.497** | **0.0004** | **1.259** | **Purifying** | **nad6** |
| **3,265** | **0.408** | **0.022** | **0.074** | **3.372** | **0.0663** | **4.137** | **Neutral** | **nad6** |
| **3,266** | **0.529** | **0.092** | **0.166** | **1.483** | **0.2234** | **9.327** | **Neutral** | **nad6** |
| **3,267** | **0.041** | **0.2** | **0.198** | **0.368** | **0.5442** | **11.124** | **Neutral** | **nad6** |
| **3,268** | **2.165** | **0.024** | **0.458** | **12.435** | **0.0004** | **25.718** | **Purifying** | **nad6** |
| **3,269** | **0** | **0.079** | **0.027** | **3.838** | **0.0501** | **1.512** | **Neutral** | **nad6** |
| **3,270** | **0.304** | **0.018** | **0.064** | **3.242** | **0.0718** | **3.579** | **Neutral** | **nad6** |
| **3,271** | **0.233** | **0.04** | **0.109** | **1.371** | **0.2416** | **6.147** | **Neutral** | **nad6** |
| **3,272** | **0.533** | **0.069** | **0.16** | **1.685** | **0.1942** | **9.002** | **Neutral** | **nad6** |
| **3,273** | **1.179** | **0.055** | **0.202** | **4.706** | **0.0301** | **11.34** | **Purifying** | **nad6** |
| **3,274** | **1.175** | **0.094** | **0.343** | **6.488** | **0.0109** | **19.291** | **Purifying** | **nad6** |
| **3,275** | **0.443** | **0.105** | **0.177** | **1.662** | **0.1974** | **9.942** | **Neutral** | **nad6** |
| **3,276** | **0.256** | **0.06** | **0.113** | **1.25** | **0.2636** | **6.324** | **Neutral** | **nad6** |
| **3,277** | **0.644** | **0.095** | **0.247** | **3.417** | **0.0645** | **13.888** | **Neutral** | **nad6** |
| **3,278** | **1.372** | **0.033** | **0.468** | **6.257** | **0.0124** | **26.299** | **Purifying** | **nad6** |
| **3,279** | **0** | **0.564** | **0.528** | **0.475** | **0.4908** | **29.644** | **Neutral** | **nad6** |
| **3,280** | **2.779** | **0.145** | **0.359** | **6.05** | **0.0139** | **20.183** | **Purifying** | **nad6** |
| **3,281** | **0.304** | **0.036** | **0.072** | **2.605** | **0.1065** | **4.059** | **Neutral** | **nad6** |
| **3,282** | **0.838** | **0.187** | **0.414** | **2.226** | **0.1357** | **23.271** | **Neutral** | **nad6** |
| **3,283** | **0.406** | **0.105** | **0.173** | **1.481** | **0.2237** | **9.698** | **Neutral** | **nad6** |
| **3,284** | **1.74** | **0.081** | **0.305** | **8.227** | **0.0041** | **17.154** | **Purifying** | **nad6** |
| **3,285** | **0.934** | **0.018** | **0.186** | **8.17** | **0.0043** | **10.46** | **Purifying** | **nad6** |
| **3,286** | **0.062** | **0.179** | **0.17** | **0.23** | **0.6314** | **9.529** | **Neutral** | **nad6** |
| **3,287** | **1.05** | **0** | **0.033** | **10.624** | **0.0011** | **1.866** | **Purifying** | **nad6** |
| **3,288** | **2.625** | **0.144** | **0.664** | **6.28** | **0.0122** | **37.287** | **Purifying** | **nad6** |
| **3,289** | **0.161** | **0.146** | **0.151** | **0.004** | **0.9503** | **8.473** | **Neutral** | **nad6** |
| **3,290** | **0.045** | **0** | **0.01** | **5.324** | **0.021** | **0.554** | **Purifying** | **nad6** |
| **3,291** | **1.571** | **0.02** | **0.403** | **12.234** | **0.0005** | **22.632** | **Purifying** | **nad6** |
| **3,292** | **0.93** | **0.056** | **0.287** | **4.963** | **0.0259** | **16.139** | **Purifying** | **nad6** |
| **3,293** | **1.629** | **0.059** | **0.666** | **7.894** | **0.005** | **37.435** | **Purifying** | **nad6** |
| **3,294** | **0** | **0.157** | **0.092** | **1.126** | **0.2887** | **5.185** | **Neutral** | **nad6** |
| **3,295** | **0.877** | **0.07** | **0.325** | **3.195** | **0.0739** | **18.255** | **Neutral** | **nad6** |
| **3,296** | **0.387** | **0.028** | **0.097** | **3.046** | **0.081** | **5.439** | **Neutral** | **nad6** |
| **3,297** | **0.674** | **0.115** | **0.336** | **2.55** | **0.1103** | **18.9** | **Neutral** | **nad6** |
| **3,298** | **1.087** | **0.118** | **0.25** | **3.549** | **0.0596** | **14.063** | **Neutral** | **nad6** |
| **3,299** | **0.785** | **0.06** | **0.255** | **3.886** | **0.0487** | **14.348** | **Purifying** | **nad6** |
| **3,300** | **1.532** | **0.108** | **0.42** | **6.525** | **0.0106** | **23.619** | **Purifying** | **nad6** |
| **3,301** | **0.358** | **0.186** | **0.214** | **0.194** | **0.6597** | **12.012** | **Neutral** | **nad6** |
| **3,302** | **1.168** | **0.106** | **0.329** | **5.513** | **0.0189** | **18.464** | **Purifying** | **nad6** |
| **3,303** | **1.753** | **0.098** | **0.521** | **5.407** | **0.0201** | **29.276** | **Purifying** | **nad6** |
| **3,304** | **0.722** | **0.073** | **0.218** | **2.969** | **0.0849** | **12.226** | **Neutral** | **nad6** |
| **3,305** | **0.775** | **0.046** | **0.299** | **5.508** | **0.0189** | **16.824** | **Purifying** | **nad6** |
| **3,306** | **0.396** | **0.104** | **0.171** | **1.419** | **0.2335** | **9.582** | **Neutral** | **nad6** |
| **3,307** | **1.801** | **0.172** | **0.657** | **6.887** | **0.0087** | **36.922** | **Purifying** | **nad6** |
| **3,308** | **1.048** | **0.026** | **0.087** | **6.255** | **0.0124** | **4.89** | **Purifying** | **nad6** |
| **3,309** | **0.738** | **0.13** | **0.28** | **2.324** | **0.1274** | **15.746** | **Neutral** | **nad6** |
| **3,310** | **0.719** | **0.051** | **0.174** | **4.93** | **0.0264** | **9.773** | **Purifying** | **nad6** |
| **3,311** | **0.711** | **0.113** | **0.256** | **2.68** | **0.1016** | **14.382** | **Neutral** | **nad6** |
| **3,312** | **0.883** | **0.108** | **0.405** | **4.269** | **0.0388** | **22.764** | **Purifying** | **nad6** |
| **3,313** | **0.103** | **0.043** | **0.064** | **0.419** | **0.5173** | **3.585** | **Neutral** | **nad6** |
| **3,314** | **0** | **0.023** | **0.012** | **1.867** | **0.1718** | **0.69** | **Neutral** | **nad6** |
| **3,315** | **0.083** | **0.058** | **0.07** | **0.055** | **0.8148** | **3.922** | **Neutral** | **nad6** |
| **3,316** | **10000** | **0.058** | **0.095** | **1.295** | **0.2551** | **5.349** | **Neutral** | **cob** |
| **3,317** | **0.217** | **0.043** | **0.101** | **1.04** | **0.3079** | **5.655** | **Neutral** | **cob** |
| **3,318** | **0.1** | **0.074** | **0.08** | **0.045** | **0.832** | **4.507** | **Neutral** | **cob** |
| **3,319** | **0.17** | **0.035** | **0.083** | **1.253** | **0.263** | **4.684** | **Neutral** | **cob** |
| **3,320** | **0** | **0.085** | **0.063** | **2.651** | **0.1035** | **3.56** | **Neutral** | **cob** |
| **3,321** | **0.157** | **0.025** | **0.027** | **0.314** | **0.5752** | **1.518** | **Neutral** | **cob** |
| **3,322** | **0.082** | **0.025** | **0.042** | **0.678** | **0.4103** | **2.374** | **Neutral** | **cob** |
| **3,323** | **1.046** | **0.133** | **0.261** | **2.097** | **0.1476** | **14.644** | **Neutral** | **cob** |
| **3,324** | **1.876** | **0.05** | **0.429** | **7.574** | **0.0059** | **24.095** | **Purifying** | **cob** |
| **3,325** | **0** | **0.148** | **0.128** | **2.767** | **0.0962** | **7.206** | **Neutral** | **cob** |
| **3,326** | **0.479** | **0.036** | **0.199** | **2.536** | **0.1113** | **11.197** | **Neutral** | **cob** |
| **3,327** | **0.09** | **0.044** | **0.052** | **0.288** | **0.5914** | **2.944** | **Neutral** | **cob** |
| **3,328** | **0.05** | **0.105** | **0.097** | **0.103** | **0.7485** | **5.432** | **Neutral** | **cob** |
| **3,329** | **0** | **0.084** | **0.049** | **4.666** | **0.0308** | **2.754** | **Diversifying** | **cob** |
| **3,330** | **0** | **0.136** | **0.123** | **2.551** | **0.1103** | **6.883** | **Neutral** | **cob** |
| **3,331** | **0.104** | **0.019** | **0.037** | **1.393** | **0.238** | **2.101** | **Neutral** | **cob** |
| **3,332** | **0.244** | **0.097** | **0.135** | **0.431** | **0.5117** | **7.591** | **Neutral** | **cob** |
| **3,333** | **0.454** | **0.116** | **0.239** | **0.945** | **0.3311** | **13.419** | **Neutral** | **cob** |
| **3,334** | **0.38** | **0.058** | **0.156** | **1.703** | **0.1918** | **8.78** | **Neutral** | **cob** |
| **3,335** | **0.817** | **0.094** | **0.122** | **1.646** | **0.1995** | **6.877** | **Neutral** | **cob** |
| **3,336** | **1.341** | **0.104** | **0.317** | **3.53** | **0.0603** | **17.824** | **Neutral** | **cob** |
| **3,337** | **0.391** | **0.083** | **0.176** | **1.191** | **0.2751** | **9.895** | **Neutral** | **cob** |
| **3,338** | **0.385** | **0.058** | **0.102** | **1.572** | **0.2099** | **5.737** | **Neutral** | **cob** |
| **3,339** | **1.7** | **0.032** | **0.227** | **7.207** | **0.0073** | **12.729** | **Purifying** | **cob** |
| **3,340** | **1.564** | **0.059** | **0.296** | **5.811** | **0.0159** | **16.641** | **Purifying** | **cob** |
| **3,341** | **0.634** | **0.05** | **0.082** | **2.502** | **0.1137** | **4.611** | **Neutral** | **cob** |
| **3,342** | **1.238** | **0.089** | **0.3** | **4.356** | **0.0369** | **16.878** | **Purifying** | **cob** |
| **3,343** | **0.308** | **0.025** | **0.07** | **1.831** | **0.176** | **3.919** | **Neutral** | **cob** |
| **3,344** | **0** | **0.088** | **0.064** | **2.872** | **0.0901** | **3.609** | **Neutral** | **cob** |
| **3,345** | **0.255** | **0.123** | **0.148** | **0.168** | **0.6818** | **8.339** | **Neutral** | **cob** |
| **3,346** | **0.28** | **0.089** | **0.132** | **0.401** | **0.5264** | **7.398** | **Neutral** | **cob** |
| **3,347** | **0.237** | **0.082** | **0.118** | **0.537** | **0.4638** | **6.604** | **Neutral** | **cob** |
| **3,348** | **0.981** | **0.052** | **0.228** | **4.564** | **0.0326** | **12.806** | **Purifying** | **cob** |
| **3,349** | **1.066** | **0.132** | **0.346** | **3.358** | **0.0669** | **19.432** | **Neutral** | **cob** |
| **3,350** | **0.6** | **0.055** | **0.195** | **2.737** | **0.098** | **10.983** | **Neutral** | **cob** |
| **3,351** | **0.026** | **0.018** | **0.021** | **0.068** | **0.7944** | **1.156** | **Neutral** | **cob** |
| **3,352** | **0** | **0.085** | **0.047** | **2.567** | **0.1091** | **2.638** | **Neutral** | **cob** |
| **3,353** | **0** | **0.094** | **0.075** | **2.541** | **0.1109** | **4.187** | **Neutral** | **cob** |
| **3,354** | **0.236** | **0.126** | **0.133** | **0.115** | **0.7345** | **7.445** | **Neutral** | **cob** |
| **3,355** | **3.529** | **0.06** | **0.547** | **12.614** | **0.0004** | **30.736** | **Purifying** | **cob** |
| **3,356** | **1.01** | **0.075** | **0.247** | **3.937** | **0.0472** | **13.864** | **Purifying** | **cob** |
| **3,357** | **0** | **0.145** | **0.119** | **1.832** | **0.1759** | **6.708** | **Neutral** | **cob** |
| **3,358** | **0.227** | **0.116** | **0.144** | **0.214** | **0.6434** | **8.097** | **Neutral** | **cob** |
| **3,359** | **2.196** | **0.068** | **0.503** | **7.579** | **0.0059** | **28.285** | **Purifying** | **cob** |
| **3,360** | **0.635** | **0.02** | **0.142** | **5.739** | **0.0166** | **7.983** | **Purifying** | **cob** |
| **3,361** | **1.404** | **0.073** | **0.241** | **5.853** | **0.0155** | **13.525** | **Purifying** | **cob** |
| **3,362** | **0.873** | **0.115** | **0.372** | **2.438** | **0.1184** | **20.924** | **Neutral** | **cob** |
| **3,363** | **0** | **0.021** | **0.017** | **0.799** | **0.3714** | **0.946** | **Neutral** | **cob** |
| **3,364** | **1.183** | **0.054** | **0.337** | **4.828** | **0.028** | **18.951** | **Purifying** | **cob** |
| **3,365** | **0.238** | **0.093** | **0.132** | **0.261** | **0.6092** | **7.405** | **Neutral** | **cob** |
| **3,366** | **0** | **0.055** | **0.035** | **1.493** | **0.2217** | **1.966** | **Neutral** | **cob** |
| **3,367** | **7.809** | **0.042** | **0.53** | **14.634** | **0.0001** | **29.752** | **Purifying** | **cob** |
| **3,368** | **10000** | **0** | **0.02** | **17.808** | **0** | **1.118** | **Purifying** | **cob** |
| **3,369** | **0.715** | **0.089** | **0.119** | **1.407** | **0.2355** | **6.699** | **Neutral** | **cob** |
| **3,370** | **1.861** | **0.049** | **0.185** | **6.222** | **0.0126** | **10.371** | **Purifying** | **cob** |
| **3,371** | **3.208** | **0.096** | **0.27** | **7.987** | **0.0047** | **15.155** | **Purifying** | **cob** |
| **3,372** | **0.786** | **0.117** | **0.235** | **1.774** | **0.1828** | **13.187** | **Neutral** | **cob** |
| **3,373** | **0** | **0.087** | **0.068** | **2.365** | **0.124** | **3.81** | **Neutral** | **cob** |
| **3,374** | **0.098** | **0.124** | **0.116** | **0.007** | **0.9314** | **6.502** | **Neutral** | **cob** |
| **3,375** | **1.216** | **0.143** | **0.251** | **3.442** | **0.0636** | **14.108** | **Neutral** | **cob** |
| **3,376** | **0.975** | **0.09** | **0.149** | **2.049** | **0.1523** | **8.367** | **Neutral** | **cob** |
| **3,377** | **0.805** | **0.168** | **0.251** | **1.374** | **0.2411** | **14.115** | **Neutral** | **cob** |
| **3,378** | **0** | **0.117** | **0.096** | **2.606** | **0.1065** | **5.397** | **Neutral** | **cob** |
| **3,379** | **2.955** | **0.024** | **0.499** | **13.698** | **0.0002** | **28.015** | **Purifying** | **cob** |
| **3,380** | **0.531** | **0.03** | **0.042** | **1.468** | **0.2256** | **2.369** | **Neutral** | **cob** |
| **3,381** | **0** | **0.1** | **0.081** | **2.131** | **0.1443** | **4.575** | **Neutral** | **cob** |
| **3,382** | **3.211** | **0.077** | **0.423** | **9.68** | **0.0019** | **23.744** | **Purifying** | **cob** |
| **3,383** | **3.213** | **0.018** | **0.13** | **10.559** | **0.0012** | **7.289** | **Purifying** | **cob** |
| **3,384** | **0.214** | **0.153** | **0.172** | **0.046** | **0.8306** | **9.659** | **Neutral** | **cob** |
| **3,385** | **2.969** | **0.058** | **0.235** | **9.763** | **0.0018** | **13.216** | **Purifying** | **cob** |
| **3,386** | **0.619** | **0.055** | **0.118** | **1.745** | **0.1865** | **6.607** | **Neutral** | **cob** |
| **3,387** | **1.105** | **0.036** | **0.058** | **3.464** | **0.0627** | **3.27** | **Neutral** | **cob** |
| **3,388** | **9.759** | **0.122** | **0.634** | **14.487** | **0.0001** | **35.596** | **Purifying** | **cob** |
| **3,389** | **3.685** | **0.07** | **0.456** | **14.436** | **0.0001** | **25.621** | **Purifying** | **cob** |
| **3,390** | **0.436** | **0.121** | **0.246** | **0.802** | **0.3705** | **13.795** | **Neutral** | **cob** |
| **3,391** | **0.549** | **0.075** | **0.094** | **1.172** | **0.279** | **5.272** | **Neutral** | **cob** |
| **3,392** | **0.824** | **0** | **0.017** | **10.414** | **0.0013** | **0.948** | **Purifying** | **cob** |
| **3,393** | **1.975** | **0.049** | **0.15** | **7.54** | **0.006** | **8.435** | **Purifying** | **cob** |
| **3,394** | **8.701** | **0** | **1.067** | **24.762** | **0** | **59.97** | **Purifying** | **cob** |
| **3,395** | **0.878** | **0.024** | **0.067** | **5.53** | **0.0187** | **3.738** | **Purifying** | **cob** |
| **3,396** | **1.029** | **0.133** | **0.247** | **3.251** | **0.0714** | **13.863** | **Neutral** | **cob** |
| **3,397** | **4.253** | **0.049** | **0.482** | **14.083** | **0.0002** | **27.093** | **Purifying** | **cob** |
| **3,398** | **1.708** | **0.113** | **0.35** | **7.262** | **0.007** | **19.646** | **Purifying** | **cob** |
| **3,399** | **1.055** | **0.133** | **0.237** | **3.231** | **0.0723** | **13.31** | **Neutral** | **cob** |
| **3,400** | **0.605** | **0.059** | **0.087** | **1.33** | **0.2489** | **4.873** | **Neutral** | **cob** |
| **3,401** | **0** | **0.035** | **0.025** | **1.105** | **0.2932** | **1.392** | **Neutral** | **cob** |
| **3,402** | **0.201** | **0.178** | **0.181** | **0.004** | **0.9518** | **10.193** | **Neutral** | **cob** |
| **3,403** | **1.054** | **0.153** | **0.246** | **2.226** | **0.1357** | **13.807** | **Neutral** | **cob** |
| **3,404** | **2.28** | **0.026** | **0.422** | **11.27** | **0.0008** | **23.699** | **Purifying** | **cob** |
| **3,405** | **0** | **0.07** | **0.058** | **1.717** | **0.1901** | **3.265** | **Neutral** | **cob** |
| **3,406** | **1.767** | **0.105** | **0.768** | **7.329** | **0.0068** | **43.149** | **Purifying** | **cob** |
| **3,407** | **8.357** | **0.073** | **0.341** | **8.569** | **0.0034** | **19.135** | **Purifying** | **cob** |
| **3,408** | **2.27** | **0.068** | **0.608** | **8.533** | **0.0035** | **34.185** | **Purifying** | **cob** |
| **3,409** | **1.111** | **0.167** | **0.231** | **1.443** | **0.2297** | **12.975** | **Neutral** | **cob** |
| **3,410** | **0.606** | **0.092** | **0.131** | **1.065** | **0.302** | **7.34** | **Neutral** | **cob** |
| **3,411** | **0.587** | **0.107** | **0.127** | **0.953** | **0.329** | **7.14** | **Neutral** | **cob** |
| **3,412** | **0** | **0.126** | **0.046** | **0.452** | **0.5016** | **2.604** | **Neutral** | **cob** |
| **3,413** | **2.205** | **0.105** | **0.343** | **8.32** | **0.0039** | **19.269** | **Purifying** | **cob** |
| **3,414** | **5.011** | **0.071** | **1.066** | **13.393** | **0.0003** | **59.897** | **Purifying** | **cob** |
| **3,415** | **6.095** | **0.162** | **0.52** | **10.975** | **0.0009** | **29.218** | **Purifying** | **cob** |
| **3,416** | **1.444** | **0.076** | **0.318** | **5.184** | **0.0228** | **17.87** | **Purifying** | **cob** |
| **3,417** | **0.074** | **0.074** | **0.074** | **0** | **1** | **4.139** | **Neutral** | **cob** |
| **3,418** | **1.129** | **0.03** | **0.203** | **5.606** | **0.0179** | **11.429** | **Purifying** | **cob** |
| **3,419** | **1.075** | **0.03** | **0.133** | **3.823** | **0.0506** | **7.492** | **Neutral** | **cob** |
| **3,420** | **2.175** | **0.067** | **0.488** | **6.968** | **0.0083** | **27.438** | **Purifying** | **cob** |
| **3,421** | **2.506** | **0.071** | **0.23** | **6.622** | **0.0101** | **12.902** | **Purifying** | **cob** |
| **3,422** | **0.234** | **0.112** | **0.121** | **0.096** | **0.7569** | **6.82** | **Neutral** | **cob** |
| **3,423** | **2.272** | **0.064** | **0.088** | **2.545** | **0.1106** | **4.927** | **Neutral** | **cob** |
| **3,424** | **0.696** | **0.118** | **0.209** | **1.843** | **0.1747** | **11.769** | **Neutral** | **cob** |
| **3,425** | **0.237** | **0.18** | **0.19** | **0.026** | **0.8708** | **10.666** | **Neutral** | **cob** |
| **3,426** | **1.478** | **0.078** | **0.307** | **5.781** | **0.0162** | **17.271** | **Purifying** | **cob** |
| **3,427** | **0.559** | **0.067** | **0.087** | **1.451** | **0.2284** | **4.864** | **Neutral** | **cob** |
| **3,428** | **15.333** | **0.024** | **0.854** | **25.283** | **0** | **47.989** | **Purifying** | **cob** |
| **3,429** | **2.462** | **0.363** | **0.764** | **3.877** | **0.049** | **42.905** | **Purifying** | **cob** |
| **3,430** | **2.364** | **0.032** | **0.511** | **10.026** | **0.0015** | **28.714** | **Purifying** | **cob** |
| **3,431** | **0.609** | **0.136** | **0.249** | **1.225** | **0.2685** | **13.972** | **Neutral** | **cob** |
| **3,432** | **0.601** | **0.117** | **0.232** | **1.435** | **0.2309** | **13.021** | **Neutral** | **cob** |
| **3,433** | **1.605** | **0.145** | **0.527** | **4.198** | **0.0405** | **29.626** | **Purifying** | **cob** |
| **3,434** | **8.441** | **0** | **0.731** | **29.205** | **0** | **41.045** | **Purifying** | **cob** |
| **3,435** | **0.559** | **0.126** | **0.161** | **0.226** | **0.6345** | **9.073** | **Neutral** | **cob** |
| **3,436** | **2.061** | **0.084** | **0.172** | **5.891** | **0.0152** | **9.659** | **Purifying** | **cob** |
| **3,437** | **1.247** | **0.07** | **0.334** | **5.875** | **0.0154** | **18.775** | **Purifying** | **cob** |
| **3,438** | **0** | **0.088** | **0.064** | **2.872** | **0.0901** | **3.609** | **Neutral** | **cob** |
| **3,439** | **177.81** | **0.031** | **0.55** | **18.145** | **0** | **30.907** | **Purifying** | **cob** |
| **3,440** | **0.688** | **0.092** | **0.194** | **2.296** | **0.1297** | **10.884** | **Neutral** | **cob** |
| **3,441** | **7.272** | **0.04** | **0.418** | **17.645** | **0** | **23.488** | **Purifying** | **cob** |
| **3,442** | **4.167** | **0.142** | **0.306** | **5.71** | **0.0169** | **17.185** | **Purifying** | **cob** |
| **3,443** | **2.662** | **0.1** | **0.278** | **6.149** | **0.0131** | **15.614** | **Purifying** | **cob** |
| **3,444** | **4.877** | **0.136** | **0.861** | **10.693** | **0.0011** | **48.354** | **Purifying** | **cob** |
| **3,445** | **3.254** | **0.109** | **0.464** | **8.836** | **0.003** | **26.049** | **Purifying** | **cob** |
| **3,446** | **0.233** | **0** | **0.015** | **10.581** | **0.0011** | **0.825** | **Purifying** | **cob** |
| **3,447** | **1.818** | **0.108** | **0.225** | **2.884** | **0.0895** | **12.62** | **Neutral** | **cob** |
| **3,448** | **0.485** | **0.158** | **0.191** | **0.239** | **0.6253** | **10.711** | **Neutral** | **cob** |
| **3,449** | **0** | **0.093** | **0.058** | **2.166** | **0.1411** | **3.263** | **Neutral** | **cob** |
| **3,450** | **0.807** | **0.041** | **0.067** | **2.111** | **0.1463** | **3.761** | **Neutral** | **cob** |
| **3,451** | **0.749** | **0.066** | **0.209** | **5.288** | **0.0215** | **11.726** | **Purifying** | **cob** |
| **3,452** | **2.467** | **0.058** | **0.494** | **12.777** | **0.0004** | **27.76** | **Purifying** | **cob** |
| **3,453** | **0.639** | **0.089** | **0.191** | **2.239** | **0.1345** | **10.724** | **Neutral** | **cob** |
| **3,454** | **2.023** | **0.028** | **0.289** | **7.305** | **0.0069** | **16.264** | **Purifying** | **cob** |
| **3,455** | **0.085** | **0.069** | **0.076** | **0.018** | **0.8923** | **4.274** | **Neutral** | **cob** |
| **3,456** | **0.687** | **0.06** | **0.072** | **0.898** | **0.3433** | **4.037** | **Neutral** | **cob** |
| **3,457** | **0.11** | **0.094** | **0.098** | **0.011** | **0.9184** | **5.505** | **Neutral** | **cob** |
| **3,458** | **1.737** | **0.175** | **0.586** | **5.282** | **0.0215** | **32.948** | **Purifying** | **cob** |
| **3,459** | **2.43** | **0.18** | **0.495** | **5.96** | **0.0146** | **27.8** | **Purifying** | **cob** |
| **3,460** | **2.199** | **0.03** | **0.396** | **9.843** | **0.0017** | **22.263** | **Purifying** | **cob** |
| **3,461** | **0.028** | **0.02** | **0.022** | **0.052** | **0.8201** | **1.261** | **Neutral** | **cob** |
| **3,462** | **3.078** | **0.058** | **0.393** | **10.124** | **0.0015** | **22.06** | **Purifying** | **cob** |
| **3,463** | **3.667** | **0.079** | **0.489** | **9.582** | **0.002** | **27.49** | **Purifying** | **cob** |
| **3,464** | **0.735** | **0.06** | **0.11** | **2.771** | **0.096** | **6.179** | **Neutral** | **cob** |
| **3,465** | **3.115** | **0.045** | **0.524** | **12.429** | **0.0004** | **29.457** | **Purifying** | **cob** |
| **3,466** | **2.33** | **0.094** | **0.352** | **10.045** | **0.0015** | **19.804** | **Purifying** | **cob** |
| **3,467** | **3.062** | **0.111** | **0.365** | **8.025** | **0.0046** | **20.49** | **Purifying** | **cob** |
| **3,468** | **0.517** | **0.109** | **0.132** | **0.855** | **0.3551** | **7.424** | **Neutral** | **cob** |
| **3,469** | **2.202** | **0.097** | **0.752** | **7.225** | **0.0072** | **42.246** | **Purifying** | **cob** |
| **3,470** | **1.9** | **0.042** | **0.354** | **8.46** | **0.0036** | **19.877** | **Purifying** | **cob** |
| **3,471** | **0.273** | **0.091** | **0.131** | **0.311** | **0.577** | **7.338** | **Neutral** | **cob** |
| **3,472** | **2.324** | **0.089** | **0.402** | **7.822** | **0.0052** | **22.613** | **Purifying** | **cob** |
| **3,473** | **2.181** | **0.115** | **0.44** | **9.958** | **0.0016** | **24.7** | **Purifying** | **cob** |
| **3,474** | **0.491** | **0.091** | **0.112** | **0.975** | **0.3235** | **6.266** | **Neutral** | **cob** |
| **3,475** | **304.171** | **0.075** | **0.262** | **8.17** | **0.0043** | **14.715** | **Purifying** | **cob** |
| **3,476** | **0.728** | **0.098** | **0.201** | **2.17** | **0.1407** | **11.294** | **Neutral** | **cob** |
| **3,477** | **2.288** | **0.024** | **0.477** | **12.276** | **0.0005** | **26.806** | **Purifying** | **cob** |
| **3,478** | **1** | **0.082** | **0.204** | **2.748** | **0.0974** | **11.474** | **Neutral** | **cob** |
| **3,479** | **0.934** | **0.1** | **0.16** | **1.906** | **0.1675** | **9.005** | **Neutral** | **cob** |
| **3,480** | **0.233** | **0.02** | **0.064** | **3.119** | **0.0774** | **3.62** | **Neutral** | **cob** |
| **3,481** | **0.575** | **0.119** | **0.152** | **0.89** | **0.3456** | **8.537** | **Neutral** | **cob** |
| **3,482** | **4.523** | **0.108** | **0.789** | **8.808** | **0.003** | **44.349** | **Purifying** | **cob** |
| **3,483** | **1.062** | **0.161** | **0.275** | **2.829** | **0.0926** | **15.456** | **Neutral** | **cob** |
| **3,484** | **0.61** | **0.043** | **0.099** | **2.472** | **0.1159** | **5.555** | **Neutral** | **cob** |
| **3,485** | **0.106** | **0.068** | **0.081** | **0.072** | **0.7881** | **4.523** | **Neutral** | **cob** |
| **3,486** | **0.886** | **0** | **0.007** | **7.77** | **0.0053** | **0.407** | **Purifying** | **cob** |
| **3,487** | **4.451** | **0.095** | **0.34** | **11.698** | **0.0006** | **19.113** | **Purifying** | **cob** |
| **3,488** | **0** | **0.094** | **0.076** | **2.083** | **0.149** | **4.292** | **Neutral** | **cob** |
| **3,489** | **0.067** | **0.085** | **0.084** | **0.008** | **0.9303** | **4.731** | **Neutral** | **cob** |
| **3,490** | **0.267** | **0.115** | **0.145** | **0.279** | **0.5972** | **8.164** | **Neutral** | **cob** |
| **3,491** | **2.636** | **0.143** | **0.8** | **8.692** | **0.0032** | **44.974** | **Purifying** | **cob** |
| **3,492** | **1.363** | **0.054** | **0.33** | **5.519** | **0.0188** | **18.514** | **Purifying** | **cob** |
| **3,493** | **1.391** | **0.177** | **0.319** | **3.258** | **0.0711** | **17.908** | **Neutral** | **cob** |
| **3,494** | **1.378** | **0.139** | **0.199** | **2.639** | **0.1043** | **11.205** | **Neutral** | **cob** |
| **3,495** | **4.591** | **0.12** | **0.696** | **13.916** | **0.0002** | **39.078** | **Purifying** | **cob** |
| **3,496** | **0.396** | **0.356** | **0.37** | **0.008** | **0.9274** | **20.809** | **Neutral** | **cob** |
| **3,497** | **0.243** | **0.043** | **0.112** | **1.089** | **0.2968** | **6.309** | **Neutral** | **cob** |
| **3,498** | **0** | **0.089** | **0.063** | **2.457** | **0.117** | **3.531** | **Neutral** | **cob** |
| **3,499** | **3.339** | **0.099** | **0.479** | **10.662** | **0.0011** | **26.927** | **Purifying** | **cob** |
| **3,500** | **0.584** | **0.059** | **0.181** | **2.508** | **0.1133** | **10.166** | **Neutral** | **cob** |
| **3,501** | **2.42** | **0.178** | **0.695** | **6.713** | **0.0096** | **39.021** | **Purifying** | **cob** |
| **3,502** | **1.484** | **0** | **0.366** | **17.109** | **0** | **20.556** | **Purifying** | **cob** |
| **3,503** | **4.89** | **0.031** | **0.473** | **14.172** | **0.0002** | **26.575** | **Purifying** | **cob** |
| **3,504** | **4.89** | **0.172** | **0.877** | **11.328** | **0.0008** | **49.298** | **Purifying** | **cob** |
| **3,505** | **2.935** | **0** | **0.387** | **19.474** | **0** | **21.733** | **Purifying** | **cob** |
| **3,506** | **0.355** | **0.024** | **0.069** | **2.093** | **0.148** | **3.853** | **Neutral** | **cob** |
| **3,507** | **3.397** | **0.033** | **0.905** | **12.299** | **0.0005** | **50.854** | **Purifying** | **cob** |
| **3,508** | **1.054** | **0** | **0.009** | **7.512** | **0.0061** | **0.495** | **Purifying** | **cob** |
| **3,509** | **0** | **0.126** | **0.112** | **2.189** | **0.139** | **6.311** | **Neutral** | **cob** |
| **3,510** | **0.212** | **0.175** | **0.182** | **0.011** | **0.9173** | **10.237** | **Neutral** | **cob** |
| **3,511** | **3.37** | **0.095** | **0.319** | **10.455** | **0.0012** | **17.93** | **Purifying** | **cob** |
| **3,512** | **0.711** | **0** | **0.008** | **4.892** | **0.027** | **0.423** | **Purifying** | **cob** |
| **3,513** | **4.235** | **0.036** | **0.377** | **13.932** | **0.0002** | **21.165** | **Purifying** | **cob** |
| **3,514** | **1.011** | **0.025** | **0.06** | **4.957** | **0.026** | **3.365** | **Purifying** | **cob** |
| **3,515** | **1.615** | **0.165** | **0.31** | **3.457** | **0.063** | **17.428** | **Neutral** | **cob** |
| **3,516** | **2.158** | **0** | **0.698** | **15.136** | **0.0001** | **39.22** | **Purifying** | **cob** |
| **3,517** | **1.436** | **0.105** | **0.244** | **4.866** | **0.0274** | **13.688** | **Purifying** | **cob** |
| **3,518** | **1.317** | **0.151** | **0.499** | **3.614** | **0.0573** | **28.018** | **Neutral** | **cob** |
| **3,519** | **0.033** | **0.138** | **0.114** | **0.652** | **0.4194** | **6.433** | **Neutral** | **cob** |
| **3,520** | **1.615** | **0.031** | **0.238** | **6.303** | **0.0121** | **13.367** | **Purifying** | **cob** |
| **3,521** | **1.07** | **0.083** | **0.209** | **2.736** | **0.0981** | **11.757** | **Neutral** | **cob** |
| **3,522** | **0** | **0.216** | **0.234** | **2.508** | **0.1133** | **13.165** | **Neutral** | **cob** |
| **3,523** | **1.923** | **0.142** | **0.303** | **4.966** | **0.0258** | **17.047** | **Purifying** | **cob** |
| **3,524** | **0.317** | **0.082** | **0.12** | **0.363** | **0.5471** | **6.723** | **Neutral** | **cob** |
| **3,525** | **1.423** | **0.061** | **0.194** | **5.52** | **0.0188** | **10.908** | **Purifying** | **cob** |
| **3,526** | **0.779** | **0.052** | **0.182** | **3.51** | **0.061** | **10.213** | **Neutral** | **cob** |
| **3,527** | **1.053** | **0.109** | **0.164** | **1.934** | **0.1643** | **9.227** | **Neutral** | **cob** |
| **3,528** | **0** | **0.108** | **0.089** | **2.446** | **0.1178** | **5.024** | **Neutral** | **cob** |
| **3,529** | **2.379** | **0.033** | **0.423** | **10.4** | **0.0013** | **23.793** | **Purifying** | **cob** |
| **3,530** | **0.961** | **0.156** | **0.268** | **1.836** | **0.1754** | **15.083** | **Neutral** | **cob** |
| **3,531** | **1.118** | **0.032** | **0.171** | **4.835** | **0.0279** | **9.628** | **Purifying** | **cob** |
| **3,532** | **2.819** | **0.022** | **0.585** | **14.773** | **0.0001** | **32.871** | **Purifying** | **cob** |
| **3,533** | **0.036** | **0.047** | **0.042** | **0.025** | **0.8737** | **2.339** | **Neutral** | **cob** |
| **3,534** | **9.454** | **0.153** | **0.271** | **3.991** | **0.0457** | **15.204** | **Purifying** | **cob** |
| **3,535** | **1.014** | **0.018** | **0.028** | **4.321** | **0.0376** | **1.575** | **Purifying** | **cob** |
| **3,536** | **1.989** | **0** | **0.47** | **15.366** | **0.0001** | **26.384** | **Purifying** | **cob** |
| **3,537** | **1.871** | **0** | **0.527** | **13.927** | **0.0002** | **29.627** | **Purifying** | **cob** |
| **3,538** | **1.242** | **0** | **0.026** | **20.472** | **0** | **1.475** | **Purifying** | **cob** |
| **3,539** | **1.1** | **0.121** | **0.256** | **3.536** | **0.0601** | **14.383** | **Neutral** | **cob** |
| **3,540** | **0.236** | **0.043** | **0.109** | **1.208** | **0.2717** | **6.118** | **Neutral** | **cob** |
| **3,541** | **0.238** | **0.093** | **0.132** | **0.261** | **0.6092** | **7.405** | **Neutral** | **cob** |
| **3,542** | **1.756** | **0.202** | **0.551** | **3.924** | **0.0476** | **30.971** | **Purifying** | **cob** |
| **3,543** | **4.048** | **0.033** | **0.763** | **10.952** | **0.0009** | **42.854** | **Purifying** | **cob** |
| **3,544** | **0.843** | **0.026** | **0.176** | **6.002** | **0.0143** | **9.862** | **Purifying** | **cob** |
| **3,545** | **0.519** | **0.158** | **0.205** | **0.508** | **0.4758** | **11.497** | **Neutral** | **cob** |
| **3,546** | **1.315** | **0.125** | **0.508** | **4.084** | **0.0433** | **28.563** | **Purifying** | **cob** |
| **3,547** | **1.418** | **0.071** | **0.253** | **5.081** | **0.0242** | **14.214** | **Purifying** | **cob** |
| **3,548** | **0.596** | **0.027** | **0.152** | **4.712** | **0.0299** | **8.525** | **Purifying** | **cob** |
| **3,549** | **3.782** | **0** | **1.034** | **18.45** | **0** | **58.107** | **Purifying** | **cob** |
| **3,550** | **0** | **0.023** | **0.016** | **1.047** | **0.3062** | **0.905** | **Neutral** | **cob** |
| **3,551** | **3.904** | **0.138** | **0.86** | **9.392** | **0.0022** | **48.312** | **Purifying** | **cob** |
| **3,552** | **5.741** | **0.069** | **0.228** | **8.842** | **0.0029** | **12.832** | **Purifying** | **cob** |
| **3,553** | **3.502** | **0.027** | **0.414** | **13.107** | **0.0003** | **23.288** | **Purifying** | **cob** |
| **3,554** | **3.881** | **0.081** | **0.445** | **8.743** | **0.0031** | **24.991** | **Purifying** | **cob** |
| **3,555** | **0.893** | **0.055** | **0.09** | **2.119** | **0.1455** | **5.043** | **Neutral** | **cob** |
| **3,556** | **2.6** | **0.171** | **0.499** | **6.185** | **0.0129** | **28.046** | **Purifying** | **cob** |
| **3,557** | **3.163** | **0.035** | **0.98** | **12.455** | **0.0004** | **55.033** | **Purifying** | **cob** |
| **3,558** | **0** | **0.019** | **0.016** | **0.703** | **0.4017** | **0.903** | **Neutral** | **cob** |
| **3,559** | **0.98** | **0.025** | **0.067** | **5.242** | **0.0221** | **3.769** | **Purifying** | **cob** |
| **3,560** | **1.666** | **0.074** | **0.393** | **7.127** | **0.0076** | **22.063** | **Purifying** | **cob** |
| **3,561** | **4.192** | **0.141** | **0.445** | **10.793** | **0.001** | **24.975** | **Purifying** | **cob** |
| **3,562** | **0.97** | **0.142** | **0.186** | **1.453** | **0.2281** | **10.444** | **Neutral** | **cob** |
| **3,563** | **0.993** | **0.089** | **0.149** | **2.084** | **0.1489** | **8.373** | **Neutral** | **cob** |
| **3,564** | **1.2** | **0.154** | **0.268** | **3.223** | **0.0726** | **15.037** | **Neutral** | **cob** |
| **3,565** | **0.539** | **0.049** | **0.074** | **1.172** | **0.279** | **4.137** | **Neutral** | **cob** |
| **3,566** | **2.97** | **0.122** | **0.649** | **9.857** | **0.0017** | **36.485** | **Purifying** | **cob** |
| **3,567** | **0.231** | **0.177** | **0.185** | **0.023** | **0.88** | **10.422** | **Neutral** | **cob** |
| **3,568** | **4.444** | **0.065** | **0.457** | **10.519** | **0.0012** | **25.674** | **Purifying** | **cob** |
| **3,569** | **2.231** | **0.192** | **0.883** | **4.423** | **0.0355** | **49.599** | **Purifying** | **cob** |
| **3,570** | **6.546** | **0.077** | **0.916** | **13.942** | **0.0002** | **51.471** | **Purifying** | **cob** |
| **3,571** | **2.854** | **0.073** | **0.416** | **9.142** | **0.0025** | **23.366** | **Purifying** | **cob** |
| **3,572** | **1.535** | **0.177** | **0.321** | **3.44** | **0.0636** | **18.06** | **Neutral** | **cob** |
| **3,573** | **0.61** | **0.171** | **0.203** | **0.596** | **0.4399** | **11.381** | **Neutral** | **cob** |
| **3,574** | **1.425** | **0.084** | **0.314** | **4.554** | **0.0328** | **17.633** | **Purifying** | **cob** |
| **3,575** | **4.055** | **0.078** | **0.68** | **14.082** | **0.0002** | **38.209** | **Purifying** | **cob** |
| **3,576** | **2.436** | **0.052** | **0.458** | **12.606** | **0.0004** | **25.721** | **Purifying** | **cob** |
| **3,577** | **1.289** | **0.111** | **0.274** | **3.907** | **0.0481** | **15.421** | **Purifying** | **cob** |
| **3,578** | **3.246** | **0.144** | **0.298** | **5.958** | **0.0147** | **16.753** | **Purifying** | **cob** |
| **3,579** | **18.482** | **0.152** | **0.521** | **13.431** | **0.0002** | **29.298** | **Purifying** | **cob** |
| **3,580** | **0.11** | **0.029** | **0.051** | **0.697** | **0.4038** | **2.867** | **Neutral** | **cob** |
| **3,581** | **1.356** | **0** | **0.475** | **12.79** | **0.0003** | **26.7** | **Purifying** | **cob** |
| **3,582** | **5.739** | **0.089** | **0.51** | **13.095** | **0.0003** | **28.662** | **Purifying** | **cob** |
| **3,583** | **2.404** | **0.03** | **0.486** | **12.871** | **0.0003** | **27.302** | **Purifying** | **cob** |
| **3,584** | **0.829** | **0.153** | **0.193** | **1.159** | **0.2816** | **10.859** | **Neutral** | **cob** |
| **3,585** | **0.086** | **0.097** | **0.094** | **0.008** | **0.93** | **5.264** | **Neutral** | **cob** |
| **3,586** | **1.559** | **0.024** | **0.211** | **7.511** | **0.0061** | **11.853** | **Purifying** | **cob** |
| **3,587** | **1.996** | **0** | **0.519** | **15.028** | **0.0001** | **29.146** | **Purifying** | **cob** |
| **3,588** | **0.008** | **0.15** | **0.101** | **1.233** | **0.2668** | **5.658** | **Neutral** | **cob** |
| **3,589** | **1.062** | **0.093** | **0.271** | **4.168** | **0.0412** | **15.199** | **Purifying** | **cob** |
| **3,590** | **1.976** | **0.024** | **0.37** | **10.219** | **0.0014** | **20.803** | **Purifying** | **cob** |
| **3,591** | **0.787** | **0** | **0.238** | **14.428** | **0.0001** | **13.381** | **Purifying** | **cob** |
| **3,592** | **113.511** | **0.058** | **0.254** | **11.771** | **0.0006** | **14.294** | **Purifying** | **cob** |
| **3,593** | **0.307** | **0.174** | **0.192** | **0.086** | **0.7698** | **10.786** | **Neutral** | **cob** |
| **3,594** | **0.395** | **0.107** | **0.214** | **0.796** | **0.3721** | **12.013** | **Neutral** | **cob** |
| **3,595** | **1.176** | **0.083** | **0.261** | **6.392** | **0.0115** | **14.684** | **Purifying** | **cob** |
| **3,596** | **1.361** | **0.117** | **0.22** | **4.061** | **0.0439** | **12.355** | **Purifying** | **cob** |
| **3,597** | **0.198** | **0.114** | **0.133** | **0.1** | **0.7518** | **7.476** | **Neutral** | **cob** |
| **3,598** | **0.537** | **0.08** | **0.3** | **2.243** | **0.1342** | **16.877** | **Neutral** | **cob** |
| **3,599** | **3.762** | **0.111** | **0.486** | **12.3** | **0.0005** | **27.296** | **Purifying** | **cob** |
| **3,600** | **0.633** | **0.137** | **0.255** | **1.375** | **0.2409** | **14.305** | **Neutral** | **cob** |
| **3,601** | **2.567** | **0** | **0.699** | **15.323** | **0.0001** | **39.271** | **Purifying** | **cob** |
| **3,602** | **0** | **0.02** | **0.015** | **0.967** | **0.3255** | **0.839** | **Neutral** | **cob** |
| **3,603** | **4.542** | **0.022** | **0.437** | **15.184** | **0.0001** | **24.574** | **Purifying** | **cob** |
| **3,604** | **0** | **0.174** | **0.144** | **0.596** | **0.4403** | **8.117** | **Neutral** | **cob** |
| **3,605** | **3.303** | **0.033** | **0.805** | **13.213** | **0.0003** | **45.255** | **Purifying** | **cob** |
| **3,606** | **0** | **0.054** | **0.027** | **2.005** | **0.1568** | **1.544** | **Neutral** | **cob** |
| **3,607** | **1.37** | **0.132** | **0.429** | **4.799** | **0.0285** | **24.1** | **Purifying** | **cob** |
| **3,608** | **0** | **0.436** | **0.418** | **1.092** | **0.2961** | **23.466** | **Neutral** | **cob** |
| **3,609** | **2.875** | **0.045** | **0.374** | **8.467** | **0.0036** | **21.009** | **Purifying** | **cob** |
| **3,610** | **1.708** | **0.059** | **0.348** | **7.414** | **0.0065** | **19.564** | **Purifying** | **cob** |
| **3,611** | **1.993** | **0.074** | **0.325** | **6.334** | **0.0118** | **18.258** | **Purifying** | **cob** |
| **3,612** | **1.567** | **0.026** | **0.201** | **7.564** | **0.006** | **11.318** | **Purifying** | **cob** |
| **3,613** | **1.446** | **0** | **0.217** | **12.772** | **0.0004** | **12.191** | **Purifying** | **cob** |
| **3,614** | **4.587** | **0.154** | **0.501** | **8.95** | **0.0028** | **28.127** | **Purifying** | **cob** |
| **3,615** | **0.659** | **0.02** | **0.143** | **5.799** | **0.016** | **8.024** | **Purifying** | **cob** |
| **3,616** | **2.365** | **0.163** | **0.433** | **6.75** | **0.0094** | **24.311** | **Purifying** | **cob** |
| **3,617** | **0.336** | **0.113** | **0.147** | **0.33** | **0.5658** | **8.237** | **Neutral** | **cob** |
| **3,618** | **1.714** | **0.155** | **0.281** | **2.272** | **0.1317** | **15.764** | **Neutral** | **cob** |
| **3,619** | **0.193** | **0.154** | **0.167** | **0.018** | **0.8932** | **9.355** | **Neutral** | **cob** |
| **3,620** | **1.852** | **0.104** | **0.266** | **5.945** | **0.0148** | **14.925** | **Purifying** | **cob** |
| **3,621** | **0.883** | **0.141** | **0.287** | **2.088** | **0.1484** | **16.099** | **Neutral** | **cob** |
| **3,622** | **0.245** | **0.026** | **0.079** | **2.252** | **0.1334** | **4.428** | **Neutral** | **cob** |
| **3,623** | **0** | **0.089** | **0.068** | **2.319** | **0.1278** | **3.837** | **Neutral** | **cob** |
| **3,624** | **3.01** | **0.018** | **0.399** | **12.528** | **0.0004** | **22.438** | **Purifying** | **cob** |
| **3,625** | **0.221** | **0.131** | **0.158** | **0.133** | **0.7157** | **8.857** | **Neutral** | **cob** |
| **3,626** | **1.49** | **0.114** | **0.492** | **3** | **0.0833** | **27.618** | **Neutral** | **cob** |
| **3,627** | **2.699** | **0.06** | **0.44** | **10.983** | **0.0009** | **24.715** | **Purifying** | **cob** |
| **3,628** | **2.484** | **0.028** | **0.054** | **5.001** | **0.0253** | **3.055** | **Purifying** | **cob** |
| **3,629** | **0.118** | **0.1** | **0.103** | **0.01** | **0.9192** | **5.799** | **Neutral** | **cob** |
| **3,630** | **1.126** | **0.033** | **0.23** | **5.612** | **0.0178** | **12.946** | **Purifying** | **cob** |
| **3,631** | **146.222** | **0.028** | **0.228** | **10.027** | **0.0015** | **12.814** | **Purifying** | **cob** |
| **3,632** | **0.52** | **0.026** | **0.078** | **3.19** | **0.0741** | **4.371** | **Neutral** | **cob** |
| **3,633** | **1.974** | **0.101** | **0.472** | **6.359** | **0.0117** | **26.525** | **Purifying** | **cob** |
| **3,634** | **1.286** | **0.024** | **0.137** | **7.109** | **0.0077** | **7.697** | **Purifying** | **cob** |
| **3,635** | **0.222** | **0.043** | **0.108** | **0.994** | **0.3187** | **6.095** | **Neutral** | **cob** |
| **3,636** | **0.961** | **0.097** | **0.175** | **2.464** | **0.1165** | **9.856** | **Neutral** | **cob** |
| **3,637** | **1.686** | **0.024** | **0.327** | **10.016** | **0.0016** | **18.362** | **Purifying** | **cob** |
| **3,638** | **0.638** | **0.323** | **0.377** | **0.245** | **0.6207** | **21.206** | **Neutral** | **cob** |
| **3,639** | **2.303** | **0.112** | **0.429** | **7.849** | **0.0051** | **24.124** | **Purifying** | **cob** |
| **3,640** | **1.374** | **0.075** | **0.485** | **4.863** | **0.0274** | **27.268** | **Purifying** | **cob** |
| **3,641** | **0** | **0.086** | **0.069** | **2.242** | **0.1343** | **3.888** | **Neutral** | **cob** |
| **3,642** | **1.361** | **0.148** | **0.283** | **3.801** | **0.0512** | **15.896** | **Neutral** | **cob** |
| **3,643** | **0.487** | **0.092** | **0.111** | **0.96** | **0.3272** | **6.252** | **Neutral** | **cob** |
| **3,644** | **0.701** | **0.042** | **0.167** | **3.236** | **0.0721** | **9.372** | **Neutral** | **cob** |
| **3,645** | **0** | **0.118** | **0.092** | **1.987** | **0.1586** | **5.185** | **Neutral** | **cob** |
| **3,646** | **0.08** | **0.071** | **0.074** | **0.007** | **0.9318** | **4.177** | **Neutral** | **cob** |
| **3,647** | **0** | **0.056** | **0.03** | **1.511** | **0.219** | **1.673** | **Neutral** | **cob** |
| **3,648** | **1.686** | **0.103** | **0.206** | **3.228** | **0.0724** | **11.558** | **Neutral** | **cob** |
| **3,649** | **0.244** | **0.175** | **0.189** | **0.034** | **0.8544** | **10.595** | **Neutral** | **cob** |
| **3,650** | **1.502** | **0.08** | **0.29** | **4.687** | **0.0304** | **16.297** | **Purifying** | **cob** |
| **3,651** | **0** | **0.108** | **0.087** | **2.56** | **0.1096** | **4.867** | **Neutral** | **cob** |
| **3,652** | **0.437** | **0.073** | **0.074** | **0.674** | **0.4118** | **4.174** | **Neutral** | **cob** |
| **3,653** | **0.23** | **0.024** | **0.081** | **2.254** | **0.1332** | **4.556** | **Neutral** | **cob** |
| **3,654** | **0.669** | **0.078** | **0.189** | **2.43** | **0.119** | **10.62** | **Neutral** | **cob** |
| **3,655** | **0.631** | **0.106** | **0.179** | **2.162** | **0.1414** | **10.05** | **Neutral** | **cob** |
| **3,656** | **3.534** | **0.028** | **0.241** | **11.072** | **0.0009** | **13.532** | **Purifying** | **cob** |
| **3,657** | **0.103** | **0.343** | **0.254** | **0.572** | **0.4494** | **14.286** | **Neutral** | **cob** |
| **3,658** | **0.202** | **0.117** | **0.135** | **0.105** | **0.7464** | **7.598** | **Neutral** | **cob** |
| **3,659** | **0.232** | **0.031** | **0.102** | **1.554** | **0.2125** | **5.757** | **Neutral** | **cob** |
| **3,660** | **0** | **0.054** | **0.027** | **2.005** | **0.1568** | **1.544** | **Neutral** | **cob** |
| **3,661** | **2.522** | **0.138** | **0.655** | **6.458** | **0.011** | **36.811** | **Purifying** | **cob** |
| **3,662** | **0.121** | **0.052** | **0.055** | **0.091** | **0.7631** | **3.111** | **Neutral** | **cob** |
| **3,663** | **0.297** | **0.056** | **0.115** | **0.734** | **0.3915** | **6.438** | **Neutral** | **cob** |
| **3,664** | **5.993** | **0** | **0.533** | **18.813** | **0** | **29.929** | **Purifying** | **cob** |
| **3,665** | **2.538** | **0** | **0.564** | **7.522** | **0.0061** | **31.69** | **Purifying** | **cob** |
| **3,666** | **0** | **0** | **0** | **0** | **1** | **0** | **Neutral** | **cob** |
| **3,667** | **0** | **0** | **0** | **0** | **1** | **0** | **Neutral** | **cob** |
| **3,668** | **1.437** | **0** | **0.266** | **6.324** | **0.0119** | **14.931** | **Purifying** | **cob** |
| **3,669** | **4.858** | **0.326** | **0.775** | **6.201** | **0.0128** | **43.567** | **Purifying** | **cob** |
| **3,670** | **1.193** | **0** | **0.247** | **6.038** | **0.014** | **13.884** | **Purifying** | **cob** |
| **3,671** | **0.595** | **0** | **0.141** | **2.794** | **0.0946** | **7.937** | **Neutral** | **cob** |
| **3,672** | **2.166** | **0** | **0.268** | **7.954** | **0.0048** | **15.052** | **Purifying** | **cob** |
| **3,673** | **0** | **0** | **0** | **0** | **1** | **0** | **Neutral** | **cob** |
| **3,674** | **0** | **0** | **0** | **0** | **1** | **0** | **Neutral** | **cob** |
| **3,675** | **0.843** | **0** | **0.33** | **3.512** | **0.0609** | **18.566** | **Neutral** | **cob** |
| **3,676** | **0** | **0** | **0** | **0** | **1** | **0** | **Neutral** | **cob** |
| **3,677** | **1.074** | **0** | **0.137** | **3.841** | **0.05** | **7.675** | **Neutral** | **cob** |
| **3,678** | **0** | **0** | **0** | **0** | **1** | **0** | **Neutral** | **cob** |
| **3,679** | **0** | **0** | **0** | **0** | **1** | **0** | **Neutral** | **cob** |
| **3,680** | **0.591** | **0** | **0.155** | **2.615** | **0.1059** | **8.732** | **Neutral** | **cob** |
| **3,681** | **0** | **0** | **0** | **0** | **1** | **0** | **Neutral** | **cob** |
| **3,682** | **0.034** | **0.036** | **0** | **-0.552** | **1** | **0** | **Neutral** | **cob** |
| **3,683** | **0** | **0.155** | **0.122** | **0.447** | **0.5036** | **6.827** | **Neutral** | **cob** |
