# Supplementary material for: Comparative Mitogenome Analyses Uncover Mitogenome Features and Phylogenetic Implications of the Rabbitfishes (Perciformes: Siganidae)
Source: Ecol Evol. 2026 Aug 14;16(8):e74188. doi: 10.1002/ece3.74188 (PMC13476152; doi:10.1002/ece3.74188)
Supplement: Supplementary file 2 [file ECE3-16-e74188-s002.docx]

Figure S2. Multiple sequence alignment of mitochondrial control regions from the 14 Siganidae mitogenomes.

Siganus_canaliculatus	-AGTTTTTAGTACATATATGTATTATCACCATATTATTATATTAACCATA

Siganus_fuscescens	-AATTTTTAGTACATATATGTATTATCACCATATTATTATATTAACCATA

Siganus_sutor	-AATTTTTAGTACATATATGTATTATCACCATATTATTATATTAACCATA

Siganus_rivulatus	-AATTTTTAGTACATATATGTATTATCACCATATTATTATATTAACCATA

Siganus_luridus	-AGTTCATAGTACATATATGTATTATCACCATATTATTATATTAACCATA

Siganus_virgatus	-AATTTCATATGCATATATGTATTATCACCATACAACTATATTAACCATG

Siganus_guttatus	-AGTTTTTAATGCATATATGTATTATCACCATGAAACTATATTAACCATA

Siganus_vulpinus	AAATTGCTAGTACATGTATGTATTATCACCATATAACCATATTAACCATA

Siganus_unimaculatus	AAATTGCTAGTACATGTATGTATTATCACCATATAACCATATTAACCATA

Siganus_puellus	-AATTGATAGTACATATATGTATTATCACCATACAATTATATTAACCATA

Siganus_corallinus	---TACCTTCCTTACCCTCTTAACAT-ATTTTATTATAACATTCTCCACA

Siganus_punctatus	---------CCTTACCCTCTTAACAT-ACTTTATCATGACATTCTCTACA

Siganus_argenteus	-------------ACACTCCTGACAT-ACTTTGTAATAACATTCTCCACA

Siganus_spinus	-----------TTACCCTTCTGACAT-ACTTCGTCATGACATTCTCCACA

*	*	** *	* * *** * *

Siganus_canaliculatus	CATAGGGG-TAATCAGGTACA-TACATGTATAATAACCATTAACAGATAT

Siganus_fuscescens	CATAGGGG-TAATCAGGTACA-TACATGTATAATAACCATTAACAGATAT

Siganus_sutor	CATAGGGGGTAATCAGGTACA-TACATGTATAATAACCATTCATAGATAT

Siganus_rivulatus	CATAGGGGGTAATCAGGTACA-TACATGTATAATAACCATTCACAGATAT

Siganus_luridus	TATAGGGA-TAATCAGGTACA-TATATGTATAATAACCATTAACAGATAT

Siganus_virgatus	AAATAGGGGTAATTAAATACA-TAAATGTATAATAAACATGATAAGATGT

Siganus_guttatus	TAATAAGAGTAATCAAGTACA-TAAATGTATAATAAACATAGTAAGGTGT

Siganus_vulpinus	TAATGAGAGTAATGAGGTACAATATATGTATAATAACCATTAAAAGATAT

Siganus_unimaculatus	TAATGAGAGTAATGAGGTACAACATATGTATAATAACCATTAAAAGATAT

Siganus_puellus	TAATAAGGGTAATTAGGTACATTA-ATGTATAATAACCATTAAAAGATAT

Siganus_corallinus	TTCCTTG--TATTTAAACTCAACAAAT---CCACTAACATCAACACAC-T

Siganus_punctatus	TTTCTTG--TATTTAAACTCAACAAAT---CCACAAATATTAATACAC-T

Siganus_argenteus	TTCCTTG--TCTTCAAGCTCAACAAAG---CCACCAACATCAACACAC-T

Siganus_spinus	TTTCTTG--TATTCAAGCTTAACAAAT---CAACCAACATCAACACAC-T

* * * *	* * *	* * **	*	*

Siganus_canaliculatus	TAACCATTTCTGTTTAGTAATATATGAAGAAGACTCAAATAAACCAACAA

Siganus_fuscescens	TAACCATTTCTGTTTAGTAATATATGAAGAAGACTCAAATAAACCAACAA

Siganus_sutor	TAACCATTTCTGTTTAGTAATATATGAAGAAGACTCAAATAAACCAGTAA

Siganus_rivulatus	TAACCATCTTTGCTTAGTAATATATGAAGAAGACTCAAATAAACCAATAA

Siganus_luridus	TACCCATTTATGTTTAGTAATATATGAAGAAGACTCAAATAAACCAATAA

Siganus_virgatus	AAACCATTTTTGCTTAGTATTATATAAAGAAGAAATACATTAACCATTAA

Siganus_guttatus	CAACCATTTTTGCTTAGTATTATATAAAGAAAAAATACATAAACCATCAA

Siganus_vulpinus	TAACCATTTCTGCTTAGTAATATAAAAATAAGAATTACATAAACCATTAA

Siganus_unimaculatus	TAACCATTTCTGCTTAGTAATATAAGAATAAGAATTACATAAACCATTAA

Siganus_puellus	CAACCATTTTTGCCTAGTAATATAAGAATAAGAATTACATAAACCATTAA

Siganus_corallinus	CGCCACCTCATGAGCGAAAGCCCCGGCCCTTACAGCCCTCACTCCCCTGA

Siganus_punctatus	CGCCACTTCCTGAGCAAAAGCCCCAACCCTTACAGCCCTTACCCCCCTAA

Siganus_argenteus	AGCCACCTCCTGAGCAAAAGCCCCAACCCTAACAGCCCTCACTCCCTTGA

Siganus_spinus	CGCCACCTCCTGAGCAAAAGCGCCGGCCCTCACAGCCCTGACCCCACTTG

*	**	*	**

Siganus_canaliculatus	GTAGAA-T-ATTCAATATGTTTTGAGTTAAA-TACTGG--CGAAATCGAA

Siganus_fuscescens	ATAGAA-T-ATTCAATATACCT-GAGTTAAA-TACTGG--CGAAATTGAA

Siganus_sutor	ATAGAA-TTATTCAAAATAGCTTGGATTAAA-TACTGG--CGAAATTGAA

Siganus_rivulatus	TAAGAA-TTTTTCAAAATAGTTTGAATTAAA-TACTGG--CGAAATCGAA

Siganus_luridus	GTAGAT-TTATTCAAAATACCTTGAATTAAA-TACTGG--CGAAATTGAA

Siganus_virgatus	TAGCTC-TAACACAATTAA-CATGAAATAGGCTGCTAG--CGAAATTGAT

Siganus_guttatus	CAATTT-TA-TACAACTAAACATGAAGTAAATTGTTAG--CGAAATTGAT

Siganus_vulpinus	TATTTAATGATTCAATTATACTTGAAATAAA-TGTTGG--CGAAATTGAA

Siganus_unimaculatus	CATTTAATGATTCAATTATACTTGAAATAAA-TGTTGG--CGAAATTGAA

Siganus_puellus	AATTCA-TAATTCAATTATATCTGAATAAAA-TGCTGG--CGAAATTGAA

Siganus_corallinus	TCCTTC-TCTCCCTTGGAGGACTCCCCCCTCTCACCGGATTTATACCAAA

Siganus_punctatus	TCCTCC-TCTCTCTTGGAGGGCTACCCCCACTTACCGGGTTCATGCCAAA

Siganus_argenteus	TTCTTC-TCTCCCTAGGAGGCCTCCCCCCGCTCACCGGATTCATGCCTAA

Siganus_spinus	TCCTAC-TCTCCCTTGGAGGCCTACCTCCGCTTACTGGGTTTATGCCTAA

*	*	*	*	*

Siganus_canaliculatus	G-ATCTAATTATA-ATTTCATTGTCAAATTTATACCACGAACTCAACATC

Siganus_fuscescens	G-ATCTAATTATA-ATTTCATTGCCAAATTTATACCACGAACTCAACATC

Siganus_sutor	G-ATCTAATTATA-ATTTCATTGTCAAATTTATACCACGAACTCAACATC

Siganus_rivulatus	G-ATCTAATTATA-ATTTCATTGTCAAATTTATACCACGGACTCAACATC

Siganus_luridus	G-ATCTAGTTATA-AATTCATTGTCAAATATATACCACGGACTCAACATC

Siganus_virgatus	CTATTCAATTGTTAATCTCATTAGTAAAGTTATACCACGAACTCAACATC

Siganus_guttatus	CTATCTAATTGTTTATCTCATTAGTAAAGTTATACCACGAACTCAACATC

Siganus_vulpinus	G-ATTTAACCGTTTAAGTCATGAGTAAAGTTATACCATGAATTCAACATC

Siganus_unimaculatus	G-ATTTAACCGTTTAAATCATGAGTAAAGTTATACCATGAATTCAACATC

Siganus_puellus	G-ATTTAAATGTA-AAACCACGAGTAAAGTTATACCACGAACTCAACATC

Siganus_corallinus	ATGACTAATCCTACAAGA-ACTAGCCAAGCAAGACCTTGG-CTTAACGGC

Siganus_punctatus	ATGACTAATTCTCCAAGA-ATTAACTAAACAAGACCTTGG-CTTAACAGC

Siganus_argenteus	ATGACTAATTCTTCAAGA-GTTAGCAAAACAAGACCTTGG-CTTAACAGC

Siganus_spinus	ATGACTTATCCTCCAAGA-ACTTACCAAACAAGATCTCGG-CCTAACAGC

* *	**	* * * *	*** *

Siganus_canaliculatus	CCGTCAAAGTCAA----CATCTCGCCCAATAAGAACCTACCATCAGTTGA

Siganus_fuscescens	CCGTCTAAGTCAA----CATCTCGCCCAATAAGAACCTACCATCAGTTGA

Siganus_sutor	CCGTCAAAACCAA----CATCTCGCCCAATAAGAACCTACCATCAGTTGA

Siganus_rivulatus	CCGTCAAAATCAA----CATCTCGCCCAATAAGAACCTACCATCAGTTGA

Siganus_luridus	TCGACAAAGTCAA----CATCTCGCCCAATAAGAACCTACCATCAGTTGA

Siganus_virgatus	TCGACAAATCTAAAA--CATCTCGCCCAATAAGAACCTACCATCAGTTGA

Siganus_guttatus	TCGACAAAGCTAAA---CATCTCGCCCAATAAGAACCTACCATCAGTTGA

Siganus_vulpinus	TCGACAAACTCAA----CATCTCACCCAATAAGAACCGACCATCAGTTGA

Siganus_unimaculatus	CCGACAAACTCAA----CATCTCGCCCAATAAGAACCGACCATCAGTTGA

Siganus_puellus	TCGACCAACTCAAA---CATCTCGCCCAATAAGAACCGACCATCAGTTGA

Siganus_corallinus	TACACTAGCAGCACTAACTGCCCTCCTAAGCCTATACTTTTATCTCCGAT

Siganus_punctatus	TACACTAGCGGCACTAACCGCCCTCCTAAGTCTATACTTCTATCTCCGAT

Siganus_argenteus	TACACTAGCTGCACTAACAGCCCTCTTAAGCTTGTACTTCTACCTCCGCC

Siganus_spinus	CACGCTAGCAGCACTTACCGCTCTCCTAAGCCTATACTTCTACCTTCGAC

* *	*	* * * * **	*	* *

Siganus_canaliculatus	TATCTTAATGCTCACGGTTATTGATGGTCAGGGACAGATATCGTGGGGGT

Siganus_fuscescens	TATCTTAATGCTCACGGTTATTGATGGTCAGGGACAGATATCGTGGGGGT

Siganus_sutor	TATCTTAATGTTAACGGTTATTGATGGTCAGGGACAGATATCGTGGGGGT

Siganus_rivulatus	TATCTTAATGTTAACGGTTATTGATGGTCAGGGACAGATATTGTGGGGGT

Siganus_luridus	TATCTTAATGATCACGGTTATTGATGGTCAGGGACAGATATCGTGGGGGT

Siganus_virgatus	TATCTTAATGCATACTGTTATTGATGGTCAGGGACAGAAATTGTGGGGGT

Siganus_guttatus	TATCTTAATGCATACTTTTATTGATGGTCAGGGACAGATACTGTGGGGGT

Siganus_vulpinus	TATCTTAATGCATACTCTTATTGATGGTCAGGGACAGTAATTGTGGGGGT

Siganus_unimaculatus	TATCTTAATGCATACTCTTATTGATGGTCAGGGACAGTAATTGTGGGGGT

Siganus_puellus	TAACTTAAGACATACTCTTATTGATGGTCAGGGACAGTAATTGTGGGGGT

Siganus_corallinus	TATCCTACGCCATAACCCTAACAATCTCCCCAAATAACCTAATCGGCACA

Siganus_punctatus	TATCCTATGCTATGACCCTAACAATTTCCCCAAACAACCTAATCGGCACA

Siganus_argenteus	TATCCTACGCCATGACCCTAACAATTTCACCCAACAACCTAATTGGAACA

Siganus_spinus	TGTCCTACGCCCTAACCCTAACAATGTCCCCAAATAATTTAATTGGCACA

* * **	**	**	* *	**

Siganus_canaliculatus	TTCACTTAGTGAACTAT-TCCTGGCATTTGGTTCCTACTTCAGGGCCATG

Siganus_fuscescens	TTCACTTAGTGAATTAT-TCCTGGCATTTGGTTCCTACTTCAGGGCCATG

Siganus_sutor	TTCACTCAGTGAATTAT-TCCTGGCATTTGGTTCCTACTTCAGGGCCATA

Siganus_rivulatus	TTCACTCAGTGAATTAT-TCCTGGCATTTGGTTCCTACTTCAGGGCCATA

Siganus_luridus	TTCACTTAGTGAATTAT-TCCTGGCATTTGGTTCCTACTTCAGGGCCATA

Siganus_virgatus	TTCACTTAGTGAACTAT-TCCTGGCATTTGGTTCCTACTTCAGGGCCATA

Siganus_guttatus	TTCACTTAATGAATTAT-TCCTGGCATTTGGTTCCTACTTCAGGGCCATA

Siganus_vulpinus	TTCACTTAGTGAATTAT-TCCTGGCATTTGGTTCCTACTTCAGGGCCATG

Siganus_unimaculatus	TTCACTTAGTGAATTAT-TCCTGGCATTTGGTTCCTACTTCAGGGCCATG

Siganus_puellus	TTCACTTAGTGAACTAT-TCCTGGCATTTGGTTCCTACTTCAGGGCCATT

Siganus_corallinus	ACACCCTGACGACTCCCCTCCTCACAACTAACCCTTCCCCTAACCATTTC

Siganus_punctatus	ACACCCTGACGACTTCCATCCTCACAATTAACCCTCCCACTGACAATTTC

Siganus_argenteus	ACACCATGACGCCTTCCCTCCTCACAACCAACACTCCCCCTGACTATTTC

Siganus_spinus	ACGCCCTGACGCCTTCCATCATCTCAACTTACCCTCCCCCTGGCTATCTC

*	*	** * **	*	*	*

Siganus_canaliculatus	AAT--TGATAT-ATTCCCCTATCTTTCATCGACGCTTGCATAAGTTAATG

Siganus_fuscescens	AAT--TGATATTATTCCCCTATCTTTCATCGACGCTTGCATAAGTTAATG

Siganus_sutor	AAT--TGATATTACTCCCCTATCTTTCATCGACGCTTGCATAAGTTAATG

Siganus_rivulatus	AAT--TGATATTATTCCCCTATCTTTCATCGACGCTTGCATAAGTTAATG

Siganus_luridus	AAT--TGATATTATTCCCCTATCTTTCATCGACGCTTGCATAAGTTAATG

Siganus_virgatus	AAT--TGATATTACTCCTCTAACTTTCATTGACGCTTGCATAAGTTAATG

Siganus_guttatus	AAT--TGATATTACTCCCCTAACTTTCATCGACGCTTGCATAAGTTAATG

Siganus_vulpinus	GAT--TGATATTATTCCCCTAACTTTCATCGACGCTTGCATAAGTTAATG

Siganus_unimaculatus	AAT--TGATATTATTCCCCTAACTTTCATCGACGCTTGCATAAGTTAATG

Siganus_puellus	TAT--TGATATTACTCCCCTAACTTTCATTGACGCTTGCATAAGTTAATG

Siganus_corallinus	AACCATAGCAACCATCTCCCTACTCCCCCTCATACCAGC-TACAGTAGCC

Siganus_punctatus	AACCATAGCAACCATTTCCCTACTCCCCCTCACACCAGC-TACAATAGCC

Siganus_argenteus	AACCATGGGAACAATCTCCCTGCTTCCCCTCACACCGGC-TATAACGGCC

Siganus_spinus	AACAATGGCAACCCTCTCCCTCCTGCCCCTGACACCAGC-TGCAATAGCA

*	*	*	*	*	** *	* * ** *

Siganus_canaliculatus	GTGGAGTACATATGGCGC----GATTACCCACCATGCCGGGCGTTCTCTC

Siganus_fuscescens	GTGGAGTACATATGGCGC----GATTACCCACCATGCCGGGCGTTCTTTC

Siganus_sutor	GTGGAGTACATATGGCGC----GATTACCCACCATGCCGAGCGTTCTCTC

Siganus_rivulatus	GTGGAGTACATATGGCGC----GATTACCCACCATGCCGGGCGTTCTTTC

Siganus_luridus	GTGGAGTACATATGGCGC----GATTACCCACCATGCCGGGCGTTCTCTC

Siganus_virgatus	GTGGGGTACATATGGCGC----GATTACCCACCATGCCGGGCGTTCTTTC

Siganus_guttatus	GTGGAGTACATATGGCGC----GATTACCCACCATGCCGGGCGTTCTCTC

Siganus_vulpinus	GTGGAGTACATATGGCGC----GATTACCCACCATGCCGGGCGTTCTTTC

Siganus_unimaculatus	GTGGAGTACATATGGCGC----GATTACCCACCATGCCGGGCGTTCTTTC

Siganus_puellus	GTGGCGTACATATGGCGC----GATTACCCACCATGCCGGGCGTTCTTTC

Siganus_corallinus	CTCCTCTCCCTCTAGGAACTTAGGCTAACCACTAGACCAAGGG--CCTTC

Siganus_punctatus	CTCCTCTCCCTGTAGGAACTTAGGCTAACCACTAGACCAAGGG--CCTTC

Siganus_argenteus	CTCCTCTCCCTGTAGGAACTTAGGCTAACCACTAGACCAAGGG--CCTTC

Siganus_spinus	CTTCTATCCCTCTAGGGACTTAGGCTAACTACCAGACCAAGGG--CCTTC

*	* * * * *	* ** * ** * ** * * * **

Siganus_canaliculatus	CAGCGGGTCAGGGGTTCTCTTTTT-CTCTTTCCTTTCATTTTGCATTTCA

Siganus_fuscescens	CAGCGGGTCAGGGGTTCTCTTTTT-CTCTTTCCTTTCATTTTGCATTTCA

Siganus_sutor	CAGCGGGTCAGGGGTTCTCTTTTT-CTCTTTCCTTTCATTTTGCATTTCA

Siganus_rivulatus	CAGCGGGTCAGGGGTTCTCTTTTT-CTCTTTCCTTTCATTTTGCATTTCA

Siganus_luridus	CAGCGGGTCACTGGTTCTCTTTTT-CTCTTTCCTTTCATTTTGCATTTCA

Siganus_virgatus	TAGGGGGTTACGGGTTTCCTTTTTTCTCTTTCCTTTCACTTGACATTTCA

Siganus_guttatus	TAGGGGGTTACGGGTTTCCTTTTTTCTCTTTCCTTTCACTTGACATTTCA

Siganus_vulpinus	CAGGGGGTCAGTGGTTCTCTTTTTTCTCTTTCCTTTCACTTGGCATTTCA

Siganus_unimaculatus	CAGGGGGTCAGTGGTTCTCTTTTTTCTCTTTCCTTTCACTTGGCATTTCA

Siganus_puellus	CAGAGGGTCACTGGTTCTCTTTTTTCTCTTTCCTTTCACTTTGCATTTCA

Siganus_corallinus	AAAGCCCTAAGTGGGAGTGAAAAT----CTCCCAGTTCCTGTAAGACTTG

Siganus_punctatus	AAAGCCCTAAGTGGGAGTGAAAAT----CTCCCAGTTCCTGTAAGACTTG

Siganus_argenteus	AAAGCCCTAAGCGGGAGTGAGAAC----CTCCCAGTCCCTGTAAGACTTG

Siganus_spinus	AAAGCCCTAAGCGGGAGTGAAAAC----CTCCCAGTCCCTGTAAGACTTG

*	* * **	* ** *	*	*

Siganus_canaliculatus	GAGTGCACACGGGAATAAAAAAT-AAGGTTGAACATTTCCTTGCGTGCAA

Siganus_fuscescens	GAGTGCACACGGGAATAAAAAAT-AAGGTTGAACATTTCCTTGCGTGCAA

Siganus_sutor	GAGTGCACACGGGAATAAAAAAT-AAGGTTGAACATTTCCTTGCGTGCAA

Siganus_rivulatus	GAGTGCACACGGGAATAAAAAAT-AAGGTTGAACATTTCCTTGCGTGCAA

Siganus_luridus	GAGTGCACACGGGAATAAAAAAT-AAGGTTGAACATTTCCTTGCATGCAA

Siganus_virgatus	CAGTGCATACAGAAATGTTATATTAAGGTTGAACATTTCCTTGCTTGAAG

Siganus_guttatus	CAGTGCATACAGAAATGTTAAATTAAGGTTGAACATTTCCTTGCTTTGAG

Siganus_vulpinus	CAGTGCATACAGAAATGGTATATTAAGGTTGAACATTTCCTTGCTT-GAA

Siganus_unimaculatus	CAGTGCATACAGAAATGGTATATTAAGGTTGAACATTTCCTTGCTT-GAA

Siganus_puellus	CAGTGCACACAGAAATGACATATCAAGGTTGAACATTTCCTTGCCT-GAC

Siganus_corallinus	CGGGATATTACCCCACATCTCCTGCATGCAAAACAGACACTTTAATTAAG

Siganus_punctatus	CGGGACACTACCCCACATCTCCTGCATGCAAAACAGACACTTTAATTAAG

Siganus_argenteus	CGGGATATTACCCCACATCTCCTGCATGCAAAACAGACACTTTAATTAAG

Siganus_spinus	CGGGACACTACCCCACATCTCCTGCATGCAAAACAGACACTTTAATTAAG

*	*	*	* * *	****	***	* *

Siganus_canaliculatus	GAACCCTTGAATGAGTGGTGAAAAGATATTACATAAGAATTGCATAATTG

Siganus_fuscescens	GAACTCTTTAATGAGTGGTGGAAAGATATTACACAAGAATTGCATAATTG

Siganus_sutor	GAACTATTAAATGAATGGTGGAAAGATATTACACAAGAATTGCATAATTG

Siganus_rivulatus	GAACTTTTAAATGAATGTTGGAAAGATATTACACAAGAATTGCATAATTG

Siganus_luridus	AAACTATTTAATGAGTGGTGAAAAGATATTACATGAGAATTGCATAATTG

Siganus_virgatus	T-ACATATGAGTGAATGGTGAAAAGATATTACACAAGAATAGCATAATCG

Siganus_guttatus	TTACATATTAGTGAATGGTGAAAAGACATTACACAAGAATAGCATAGTAG

Siganus_vulpinus	TTAT-TATAAGTGAAAGGTGGAAAGACATTACACAAGAATTGCATAATTG

Siganus_unimaculatus	TTAT-TATAAGTGAAAGGTGGAAAGACATTACACAAGAATTGCATAATTG

Siganus_puellus	TTAT-TGTAAATGAAGGGTGGAAAGACATTACATAAGAGTTGCATATTTG

Siganus_corallinus	CTAAAGCCTTATCTAGATAGGTAGGCCTCGATCCTACAAACTCTTAGTTA

Siganus_punctatus	CTAAAGCCTTATCTAGATGAGTAGGCCTCGATCCTACAAACTCTTAGTTA

Siganus_argenteus	CTAAAGCCTTATCTAGATAGGTAGGCCTCGATCCTACAAACTCTTAGTTA

Siganus_spinus	CTAAAGCCTTATCTAGATAGGTAGGCCTCGATCCTACAAACTCTTAGTTA

*	*	* *	*	* *	* ** *

Siganus_canaliculatus	GATATCAAGAGCATAAATGTATGATATTTCTCCTAACATATCCTGATATG

Siganus_fuscescens	GATATCAAGAGCATAAGTGTATGATATTTCTCCTAACATATCCTGATATG

Siganus_sutor	GATATCAAGAGCATAAGTGTGTGATATTTCTCCTAACATATCCTGATATG

Siganus_rivulatus	GATATCAAGAGCATAAGTATGTGATATTTCTCCTAACATATCCTGATATG

Siganus_luridus	GATATCAAGAGCATAAGTGGGTGATATTTCTCCTAACATATCCTGATATG

Siganus_virgatus	GATATCAAGAGCATAAATAGATGAAATTTCTCCTAACATATCCTTATATG

Siganus_guttatus	GATATCAAGAGCATAAGTAGATGATATTTCTCCTAACATATCCTTATATA

Siganus_vulpinus	GATATCAAGAGCATAAGTATCTGATATTTCTCCTAACATATCCTTATATG

Siganus_unimaculatus	GATATCAAGAGCATAAGTATCTGATATTTCTCCTAACATATCCTTATATG

Siganus_puellus	GATATCAAGAGCATAAGTGTGTGATATTTCTCCTAACATATCCTTATATG

Siganus_corallinus	ACAGCTAAGCGCTCAAACCAGCGAGCATCCATCTACCTTTCCCCCGCCTA

Siganus_punctatus	ACAGCTAAGCGCTCAAACCAGCGAGCATCCATCTACCTTTCCCCCGCCTG

Siganus_argenteus	ACAGCTAAGCGCTCAAACCAGCGAGCATCCATCTACCTTTCCCCCGCCGA

Siganus_spinus	ACAGCTAAGCGCTCAAACCAGCGAGCATCCATCTACCTTTCCCCCGCCTA

*** ** **	**	* * *** * * **

Siganus_canaliculatus	CCCCCCGGGGGGTTTTTGAGCGTAAAACCCCC------CTACCCCCCTAT

Siganus_fuscescens	CCCCCTGGGGG-TTTTTGAGCGTAAAACCCCC------CTACCCCCCTAT

Siganus_sutor	CCCCCCGTGGG-TTTTTGAGCGTAAAACCCCC------CTACCCCCCTAT

Siganus_rivulatus	CCCCCCGTGGG-TTTTTGAGCGTAAA-CCCCC------CTACCCCCCTAT

Siganus_luridus	CCCCCCGGGGG-TTTTTGAGCGTAAAACCCCC------CTACCCCCCTAT

Siganus_virgatus	CCCCC-GGGG--TTTTTGCGCGTTAAACCCCC------CTACCCCCCTAT

Siganus_guttatus	CCCCC-GGGGG-TTTTTGCGCGTTAAACCCCC------CTACCCCCCTAT

Siganus_vulpinus	CCCCC-GGGGG-TTTTTGCGCGTTAAACCCCC------CTACCCCCCTAC

Siganus_unimaculatus	CCCCC-GGGGG-TTTTTGCGCGTTAAACCCCC------CTACCCCCCTAC

Siganus_puellus	CCCCC-GGGGG-TTTTTGCGCGTTAAACCCCC------CTACCCCCCTAC

Siganus_corallinus	ACA---GGCGTGCCAAGGCGGGGGAAAGCCCCGGCAGGCTTTACCCTGCG

Siganus_punctatus	ATA---GGCGCGCCAAGGCGGGGGAAAGCCCCGGCAGGCTTTACCCTGCT

Siganus_argenteus	ATC---GGCGGGCCAAGGCGGGGGAAAGCCCCGGCAGGCTTTACCCTGCT

Siganus_spinus	A-A---GGCGCGCCAAGGCGGGGGAAAGCCCCGGCAGGCTTTACCCTGCT

* *	* * * ** ****	**	***

Siganus_canaliculatus	ACTCCTGAGATCACTATCATTCCTG-AAAACCCCCCGGAA----ACAGGA

Siganus_fuscescens	ACTCCTGAGATCACTATCATTCCTG-AAAACCCCCCGGAA----ACAGGA

Siganus_sutor	ACTCCTGAGATCACTATCATTCCTG-AAAACCCCCCGGAA----ACAGGA

Siganus_rivulatus	ACTCCTGAGATCATTATCATTCCTG-AAAACCCCCCGGAA----ACAGGA

Siganus_luridus	ACTCCTGAGATCACTATCATTCCTG-AAAACCCCCCGGAA----ACAGGA

Siganus_virgatus	ACTCCCGAGATCACTATTATTCCTG-AAAACCCCCCGGAA----ACAGGA

Siganus_guttatus	ACTCCCGAGATCACTATCATTCCTG-AAAACCCCCCGGAA----ACAGGA

Siganus_vulpinus	ACTCCCGAGATCACTATCATTCCTG-AAAACCCCCCGGAA----ACAGGA

Siganus_unimaculatus	ACTCCCGAGATCACTATCATTCCTG-AAAACCCCCCGGAA----ACAGGA

Siganus_puellus	ACTCCCGAGATCGCTATTATTCCTG-AAAACCCCCCGGAA----ACAGGA

Siganus_corallinus	ACTTCAGAT-TTGCAATCTGACATGTAAAACACCTCAGAGCTTGGTAAGA

Siganus_punctatus	ACTTCAGAT-TTGCAATCTAACATGTAAAACACCTCAGAGCTTGGTAAGA

Siganus_argenteus	ACTTCAGAT-TTGCAATCTGACGTGTAAAACACCTCAAGACTTGGCAAGA

Siganus_spinus	ACTTCAGAT-TTGCAATCTAACATGTAAAACACCTCAGAGCTTGGTAAGA

*** * ** *	**	* ** ***** ** *	* **

Siganus_canaliculatus	AAAC--CTCGAGTAGCATATTTTT-AGCCCAAAACGTGTCTATTACATTA

Siganus_fuscescens	AAAC--CTCGAGTAGCATATTTTT-AGCCCAAAACGTGTCTATTACATTA

Siganus_sutor	AAAC--CTCGAGTAGCATATTTTT-AGCCCAAAACGCGTCTATTACATTA

Siganus_rivulatus	AAAC--CTCGAGTAGCATATTTTT-AGCCCAAAACGCGTCTATTACATTA

Siganus_luridus	AAAC--CTCGAGTAGCATATTTTT-AGCCCAAAACGCGTCTATTACATTA

Siganus_virgatus	AAAC--CTCGAGTAGCATATTTTTCAACCTAAAACGTGTCTATTACATTA

Siganus_guttatus	AAAC--CTCGAGTAGCATATTTTTCAACCTAAAACGTGTCTATTACATTA

Siganus_vulpinus	AAAC--CTCGAGTAGCATATTTCTTAACCTAAAACGTGTCTATTACATTA

Siganus_unimaculatus	AAAC--CTCGAGTAGCATATTTCTTAACCTAAAACGTGTCTATTACATTA

Siganus_puellus	AAAC--CTCGAGTAGCATATTTTTTAACCTAAAACGTGTCTATTACATTA

Siganus_corallinus	AGAGGATTCAAACCTC-TGTCCATGGGGCTACAATCCACCGCTTAAAC-G

Siganus_punctatus	AGAGGACTCAAACCTC-TGTCCATGGGGCTACAATCCACCGCTTAGAC-G

Siganus_argenteus	AGAGGACTCAAACCTC-TGTGCATGGGGCTACAATCCACCGCTTAAAC-A

Siganus_spinus	AGAGGACTTAAACCTC-TGTCCATGGGGCTACAATCCACCGCTTAAAC-A

* *	* *	* * *	*	* * **	* *** *

Siganus_canaliculatus	TTTAAATATTGCACAT

Siganus_fuscescens	TTTAAATATTGCACAT

Siganus_sutor	TTTAAATATTGCACAT

Siganus_rivulatus	TTTAAATATTGCACAT

Siganus_luridus	TTTAAATATTGCACAT

Siganus_virgatus	TTTAAATAATGCACAT

Siganus_guttatus	TTTAAATAATGCACAT

Siganus_vulpinus	TTTAAATAATACACAT

Siganus_unimaculatus	TTTAAATAATACACAT

Siganus_puellus	TTTAAATAATGCACAT

Siganus_corallinus	CTCAGCCATCTTACCT

Siganus_punctatus	CTCAGCCATCTTACCT

Siganus_argenteus	CTCAGCCATCTTACCT

Siganus_spinus	CTCAGCCATCTTACCT

* *	*	** *
