# Supplementary material for: Comparative Mitogenome Analyses Uncover Mitogenome Features and Phylogenetic Implications of the Rabbitfishes (Perciformes: Siganidae)
Source: Ecol Evol. 2026 Aug 14;16(8):e74188. doi: 10.1002/ece3.74188 (PMC13476152; doi:10.1002/ece3.74188)
Supplement: Supplementary file 4 [file ECE3-16-e74188-s004.docx]

Table S1. Sequencing, assembly, and assembly-support metrics for the four newly generated *Siganus* mitogenomes.

| **GenBank accession** | **Mitogenome length**  **(bp)** | **N50 length**  **(bp)** | **Raw data**  **(Mb)** | **Clean data**  **(Mb)** | **Clean data Q20**  **(%)** | **Clean data Q30**  **(%)** | **Clean data GC**  **(%)** | **Mitogenome GC content (%)** | **N rate**  **(%)** | **Total Clean bases**  **(bp)** | **Estimated theoretical depth (×)** | **Mean estimated theoretical depth(×)** | **Final assembly sequence number** | **Assembled sequences** | **Complete 37-gene set recovered** |
| --- | --- | --- | --- | --- | --- | --- | --- | --- | --- | --- | --- | --- | --- | --- | --- |
| NC_086723 | 16,495 | 16,495 | 16,245 | 16199.1 | 97.12 | 93.25 | 43.09 | 46.07 | 0 | 16,199,126,804 | 982,061.23 | 791,380.53 | 1 | 1 | Yes |
| PX112429 | 16,510 | 16,510 | 10,145 | 9992.2 | 97.45 | 92.45 | 43.32 | 45.11 | 0 | 9,992,480,160 | 605,221.08 | 791,380.53 | 1 | 1 | Yes |
| PX112430 | 16,505 | 16,505 | 13,158 | 13123.3 | 95.96 | 90.61 | 42.26 | 45.74 | 0 | 13,123,395,882 | 795,110.57 | 791,380.53 | 1 | 1 | Yes |
| PX112431 | 16,496 | 16,496 | 12,986 | 12918.5 | 96.10 | 90.92 | 43.45 | 46.31 | 0 | 12,918,055,520 | 783,129.24 | 791,380.53 | 1 | 1 | Yes |
