# Supplementary material for: Comparative Mitogenome Analyses Uncover Mitogenome Features and Phylogenetic Implications of the Rabbitfishes (Perciformes: Siganidae)
Source: Ecol Evol. 2026 Aug 14;16(8):e74188. doi: 10.1002/ece3.74188 (PMC13476152; doi:10.1002/ece3.74188)
Supplement: Supplementary file 5 [file ECE3-16-e74188-s005.docx]

Figure S5. Putative secondary structures of the origin of light-strand replication (O*_L_*) across the 14 Siganidae mitogenomes.
