# Supplementary material for: Comparative Mitogenome Analyses Uncover Mitogenome Features and Phylogenetic Implications of the Rabbitfishes (Perciformes: Siganidae)
Source: Ecol Evol. 2026 Aug 14;16(8):e74188. doi: 10.1002/ece3.74188 (PMC13476152; doi:10.1002/ece3.74188)
Supplement: Supplementary file 6 [file ECE3-16-e74188-s006.docx]

Figure S1. Circular mitochondrial genome maps of the 14 Siganidae species analyzed in this study.

*Siganus virgatus*

*Siganus vulpinus*

*Siganus canaliculatus*

*Siganus guttatus*

*Siganus puellus*

*Siganus fuscescens*

*Siganus sutor*

*Siganus unimaculatus*

*Siganus rivulatus*

*Siganus luridus*

*Siganus argenteus*

*Siganus corallinus*

*Siganus punctatus*

*Siganus spinus*
