# Supplementary material for: Comparative Mitogenome Analyses Uncover Mitogenome Features and Phylogenetic Implications of the Rabbitfishes (Perciformes: Siganidae)
Source: Ecol Evol. 2026 Aug 14;16(8):e74188. doi: 10.1002/ece3.74188 (PMC13476152; doi:10.1002/ece3.74188)
Supplement: Supplementary file 7 [file ECE3-16-e74188-s007.docx]

Table S2. Gene organization and annotation information for the 14 Siganidae mitogenomes analyzed in this study.

| **Genome** | **Gene** | **Start** | **End** | **Strand** | **Length(bp)** | **Intergenic region (bp)** | **Start codon** | **Stop codon** | **Anticodon** | **Amino acid** |
| --- | --- | --- | --- | --- | --- | --- | --- | --- | --- | --- |
| Siganus argenteus | trnF-GAA | 1 | 68 | F | 68 | - | - | - | GAA | Phe |
| Siganus argenteus | rrnS | 69 | 1014 | F | 946 | 0 | - | - | - |  |
| Siganus argenteus | trnV-TAC | 1015 | 1086 | F | 72 | 0 | - | - | TAC | Val |
| Siganus argenteus | rrnL | 1126 | 2778 | F | 1653 | 39 | - | - | - |  |
| Siganus argenteus | trnL2-TAA | 2779 | 2852 | F | 74 | 0 | - | - | TAA | Leu2 |
| Siganus argenteus | nad1 | 2853 | 3827 | F | 975 | 0 | ATG | TAA | - |  |
| Siganus argenteus | trnI-GAT | 3832 | 3901 | F | 70 | 4 | - | - | GAT | Ile |
| Siganus argenteus | trnQ-TTG | 3901 | 3971 | R | 71 | -1 | - | - | TTG | Gln |
| Siganus argenteus | trnM-CAT | 3971 | 4039 | F | 69 | -1 | - | - | CAT | Met |
| Siganus argenteus | nad2 | 4040 | 5086 | F | 1047 | 0 | ATG | TAG | - |  |
| Siganus argenteus | trnW-TCA | 5085 | 5156 | F | 72 | -2 | - | - | TCA | Trp |
| Siganus argenteus | trnA-TGC | 5157 | 5225 | R | 69 | 0 | - | - | TGC | Ala |
| Siganus argenteus | trnN-GTT | 5228 | 5300 | R | 73 | 2 | - | - | GTT | Asn |
| Siganus argenteus | trnC-GCA | 5338 | 5403 | R | 66 | 37 | - | - | GCA | Cys |
| Siganus argenteus | trnY-GTA | 5404 | 5474 | R | 71 | 0 | - | - | GTA | Tyr |
| Siganus argenteus | cox1 | 5476 | 7026 | F | 1551 | 1 | GTG | TAA | - |  |
| Siganus argenteus | trnS2-TGA | 7029 | 7099 | R | 71 | 2 | - | - | TGA | Ser2 |
| Siganus argenteus | trnD-GTC | 7103 | 7174 | F | 72 | 3 | - | - | GTC | Asp |
| Siganus argenteus | cox2 | 7183 | 7873 | F | 691 | 8 | ATG | T | - |  |
| Siganus argenteus | trnK-TTT | 7874 | 7948 | F | 75 | 0 | - | - | TTT | Lys |
| Siganus argenteus | atp8 | 7950 | 8117 | F | 168 | 1 | ATG | TAA | - |  |
| Siganus argenteus | atp6 | 8108 | 8791 | F | 684 | -10 | ATG | TAA | - |  |
| Siganus argenteus | cox3 | 8791 | 9576 | F | 786 | -1 | ATG | TAA | - |  |
| Siganus argenteus | trnG-TCC | 9576 | 9648 | F | 73 | -1 | - | - | TCC | Gly |
| Siganus argenteus | nad3 | 9649 | 9999 | F | 351 | 0 | ATG | TAG | - |  |
| Siganus argenteus | trnR-TCG | 9998 | 10066 | F | 69 | -2 | - | - | TCG | Arg |
| Siganus argenteus | nad4l | 10067 | 10363 | F | 297 | 0 | ATG | TAA | - |  |
| Siganus argenteus | nad4 | 10357 | 11737 | F | 1381 | -7 | ATG | T | - |  |
| Siganus argenteus | trnH-GTG | 11738 | 11806 | F | 69 | 0 | - | - | GTG | His |
| Siganus argenteus | trnS1-GCT | 11807 | 11874 | F | 68 | 0 | - | - | GCT | Ser1 |
| Siganus argenteus | trnL1-TAG | 11880 | 11952 | F | 73 | 5 | - | - | TAG | Leu1 |
| Siganus argenteus | nad5 | 11953 | 13791 | F | 1839 | 0 | ATG | TAA | - |  |
| Siganus argenteus | nad6 | 13788 | 14309 | R | 522 | -4 | ATG | TAG | - |  |
| Siganus argenteus | trnE-TTC | 14310 | 14378 | R | 69 | 0 | - | - | TTC | Glu |
| Siganus argenteus | cob | 14383 | 15523 | F | 1141 | 4 | ATG | T | - |  |
| Siganus argenteus | trnT-TGT | 15524 | 15595 | F | 72 | 0 | - | - | TGT | Thr |
| Siganus argenteus | trnP-TGG | 15595 | 15664 | R | 70 | -1 | - | - | TGG | Pro |
| Siganus argenteus | control region | 15665 | 16495 | - | 831 | 0 | - | - | - |  |
| Siganus corallinus | trnF-GAA | 1 | 68 | F | 68 | - | - | - | GAA | Phe |
| Siganus corallinus | rrnS | 69 | 1017 | F | 949 | 0 | - | - | - |  |
| Siganus corallinus | trnV-TAC | 1018 | 1089 | F | 72 | 0 | - | - | TAC | Val |
| Siganus corallinus | rrnL | 1128 | 2781 | F | 1654 | 38 | - | - | - |  |
| Siganus corallinus | trnL2-TAA | 2782 | 2855 | F | 74 | 0 | - | - | TAA | Leu2 |
| Siganus corallinus | nad1 | 2856 | 3830 | F | 975 | 0 | ATG | TAA | - |  |
| Siganus corallinus | trnI-GAT | 3835 | 3904 | F | 70 | 4 | - | - | GAT | Ile |
| Siganus corallinus | trnQ-TTG | 3904 | 3974 | R | 71 | -1 | - | - | TTG | Gln |
| Siganus corallinus | trnM-CAT | 3974 | 4042 | F | 69 | -1 | - | - | CAT | Met |
| Siganus corallinus | nad2 | 4043 | 5089 | F | 1047 | 0 | ATG | TAG | - |  |
| Siganus corallinus | trnW-TCA | 5088 | 5159 | F | 72 | -2 | - | - | TCA | Trp |
| Siganus corallinus | trnA-TGC | 5160 | 5228 | R | 69 | 0 | - | - | TGC | Ala |
| Siganus corallinus | trnN-GTT | 5231 | 5303 | R | 73 | 2 | - | - | GTT | Asn |
| Siganus corallinus | trnC-GCA | 5341 | 5406 | R | 66 | 37 | - | - | GCA | Cys |
| Siganus corallinus | trnY-GTA | 5407 | 5477 | R | 71 | 0 | - | - | GTA | Tyr |
| Siganus corallinus | cox1 | 5479 | 7029 | F | 1551 | 1 | GTG | TAG | - |  |
| Siganus corallinus | trnS2-TGA | 7032 | 7102 | R | 71 | 2 | - | - | TGA | Ser2 |
| Siganus corallinus | trnD-GTC | 7106 | 7177 | F | 72 | 3 | - | - | GTC | Asp |
| Siganus corallinus | cox2 | 7186 | 7876 | F | 691 | 8 | ATG | T | - |  |
| Siganus corallinus | trnK-TTT | 7877 | 7951 | F | 75 | 0 | - | - | TTT | Lys |
| Siganus corallinus | atp8 | 7953 | 8120 | F | 168 | 1 | ATG | TAA | - |  |
| Siganus corallinus | atp6 | 8111 | 8794 | F | 684 | -10 | ATG | TAA | - |  |
| Siganus corallinus | cox3 | 8794 | 9579 | F | 786 | -1 | ATG | TAA | - |  |
| Siganus corallinus | trnG-TCC | 9579 | 9651 | F | 73 | -1 | - | - | TCC | Gly |
| Siganus corallinus | nad3 | 9652 | 10002 | F | 351 | 0 | ATG | TAG | - |  |
| Siganus corallinus | trnR-TCG | 10001 | 10069 | F | 69 | -2 | - | - | TCG | Arg |
| Siganus corallinus | nad4l | 10070 | 10366 | F | 297 | 0 | ATG | TAA | - |  |
| Siganus corallinus | nad4 | 10360 | 11740 | F | 1381 | -7 | ATG | T | - |  |
| Siganus corallinus | trnH-GTG | 11741 | 11809 | F | 69 | 0 | - | - | GTG | His |
| Siganus corallinus | trnS1-GCT | 11810 | 11877 | F | 68 | 0 | - | - | GCT | Ser1 |
| Siganus corallinus | trnL1-TAG | 11884 | 11956 | F | 73 | 6 | - | - | TAG | Leu1 |
| Siganus corallinus | nad5 | 11957 | 13795 | F | 1839 | 0 | ATG | TAA | - |  |
| Siganus corallinus | nad6 | 13792 | 14313 | R | 522 | -4 | ATG | TAA | - |  |
| Siganus corallinus | trnE-TTC | 14314 | 14382 | R | 69 | 0 | - | - | TTC | Glu |
| Siganus corallinus | cob | 14388 | 15528 | F | 1141 | 5 | ATG | T | - |  |
| Siganus corallinus | trnT-TGT | 15529 | 15600 | F | 72 | 0 | - | - | TGT | Thr |
| Siganus corallinus | trnP-TGG | 15600 | 15669 | R | 70 | -1 | - | - | TGG | Pro |
| Siganus corallinus | control region | 15670 | 16510 | - | 841 | 0 | - | - | - |  |
| Siganus punctatus | trnF-GAA | 1 | 68 | F | 68 | - | - | - | GAA | Phe |
| Siganus punctatus | rrnS | 69 | 1015 | F | 947 | 0 | - | - |  |  |
| Siganus punctatus | trnV-TAC | 1016 | 1087 | F | 72 | 0 | - | - | TAC | Val |
| Siganus punctatus | rrnL | 1126 | 2781 | F | 1656 | 38 | - | - |  |  |
| Siganus punctatus | trnL2-TAA | 2782 | 2855 | F | 74 | 0 | - | - | TAA | Leu2 |
| Siganus punctatus | nad1 | 2856 | 3830 | F | 975 | 0 | ATG | TAA |  |  |
| Siganus punctatus | trnI-GAT | 3835 | 3904 | F | 70 | 4 | - | - | GAT | Ile |
| Siganus punctatus | trnQ-TTG | 3904 | 3974 | R | 71 | -1 | - | - | TTG | Gln |
| Siganus punctatus | trnM-CAT | 3974 | 4042 | F | 69 | -1 | - | - | CAT | Met |
| Siganus punctatus | nad2 | 4043 | 5089 | F | 1047 | 0 | ATG | TAG |  |  |
| Siganus punctatus | trnW-TCA | 5088 | 5159 | F | 72 | -2 | - | - | TCA | Trp |
| Siganus punctatus | trnA-TGC | 5160 | 5228 | R | 69 | 0 | - | - | TGC | Ala |
| Siganus punctatus | trnN-GTT | 5231 | 5303 | R | 73 | 2 | - | - | GTT | Asn |
| Siganus punctatus | trnC-GCA | 5341 | 5406 | R | 66 | 37 | - | - | GCA | Cys |
| Siganus punctatus | trnY-GTA | 5407 | 5477 | R | 71 | 0 | - | - | GTA | Tyr |
| Siganus punctatus | cox1 | 5479 | 7029 | F | 1551 | 1 | GTG | TAG |  |  |
| Siganus punctatus | trnS2-TGA | 7032 | 7102 | R | 71 | 2 | - | - | TGA | Ser2 |
| Siganus punctatus | trnD-GTC | 7106 | 7177 | F | 72 | 3 | - | - | GTC | Asp |
| Siganus punctatus | cox2 | 7187 | 7877 | F | 691 | 9 | ATG | T |  |  |
| Siganus punctatus | trnK-TTT | 7878 | 7952 | F | 75 | 0 | - | - | TTT | Lys |
| Siganus punctatus | atp8 | 7954 | 8121 | F | 168 | 1 | ATG | TAA |  |  |
| Siganus punctatus | atp6 | 8112 | 8795 | F | 684 | -10 | ATG | TAA |  |  |
| Siganus punctatus | cox3 | 8795 | 9580 | F | 786 | -1 | ATG | TAA |  |  |
| Siganus punctatus | trnG-TCC | 9580 | 9652 | F | 73 | -1 | - | - | TCC | Gly |
| Siganus punctatus | nad3 | 9653 | 10003 | F | 351 | 0 | ATG | TAG |  |  |
| Siganus punctatus | trnR-TCG | 10002 | 10070 | F | 69 | -2 | - | - | TCG | Arg |
| Siganus punctatus | nad4l | 10071 | 10367 | F | 297 | 0 | ATG | TAA |  |  |
| Siganus punctatus | nad4 | 10361 | 11741 | F | 1381 | -7 | ATG | T |  |  |
| Siganus punctatus | trnH-GTG | 11742 | 11810 | F | 69 | 0 | - | - | GTG | His |
| Siganus punctatus | trnS1-GCT | 11811 | 11878 | F | 68 | 0 | - | - | GCT | Ser1 |
| Siganus punctatus | trnL1-TAG | 11885 | 11957 | F | 73 | 6 | - | - | TAG | Leu1 |
| Siganus punctatus | nad5 | 11958 | 13796 | F | 1839 | 0 | ATG | TAA |  |  |
| Siganus punctatus | nad6 | 13793 | 14314 | R | 522 | -4 | ATG | TAG |  |  |
| Siganus punctatus | trnE-TTC | 14315 | 14383 | R | 69 | 0 | - | - | TTC | Glu |
| Siganus punctatus | cob | 14389 | 15529 | F | 1141 | 5 | ATG | T |  |  |
| Siganus punctatus | trnT-TGT | 15530 | 15601 | F | 72 | 0 | - | - | TGT | Thr |
| Siganus punctatus | trnP-TGG | 15601 | 15670 | R | 70 | -1 | - | - | TGG | Pro |
| Siganus punctatus | control region | 15671 | 16505 | - | 835 | 0 | - | - | - |  |
| Siganus spinus | trnF-GAA | 1 | 68 | F | 68 | - | - | - | GAA | Phe |
| Siganus spinus | rrnS | 69 | 1014 | F | 946 | 0 | - | - | - |  |
| Siganus spinus | trnV-TAC | 1015 | 1086 | F | 72 | 0 | - | - | TAC | Val |
| Siganus spinus | rrnL | 1125 | 2778 | F | 1654 | 38 | - | - | - |  |
| Siganus spinus | trnL2-TAA | 2779 | 2852 | F | 74 | 0 | - | - | TAA | Leu2 |
| Siganus spinus | nad1 | 2853 | 3827 | F | 975 | 0 | ATG | TAA | - |  |
| Siganus spinus | trnI-GAT | 3832 | 3901 | F | 70 | 4 | - | - | GAT | Ile |
| Siganus spinus | trnQ-TTG | 3901 | 3971 | R | 71 | -1 | - | - | TTG | Gln |
| Siganus spinus | trnM-CAT | 3971 | 4039 | F | 69 | -1 | - | - | CAT | Met |
| Siganus spinus | nad2 | 4040 | 5086 | F | 1047 | 0 | ATG | TAG | - |  |
| Siganus spinus | trnW-TCA | 5085 | 5156 | F | 72 | -2 | - | - | TCA | Trp |
| Siganus spinus | trnA-TGC | 5157 | 5225 | R | 69 | 0 | - | - | TGC | Ala |
| Siganus spinus | trnN-GTT | 5228 | 5300 | R | 73 | 2 | - | - | GTT | Asn |
| Siganus spinus | trnC-GCA | 5337 | 5402 | R | 66 | 36 | - | - | GCA | Cys |
| Siganus spinus | trnY-GTA | 5403 | 5473 | R | 71 | 0 | - | - | GTA | Tyr |
| Siganus spinus | cox1 | 5475 | 7025 | F | 1551 | 1 | GTG | TAA | - |  |
| Siganus spinus | trnS2-TGA | 7028 | 7098 | R | 71 | 2 | - | - | TGA | Ser2 |
| Siganus spinus | trnD-GTC | 7102 | 7173 | F | 72 | 3 | - | - | GTC | Asp |
| Siganus spinus | cox2 | 7182 | 7872 | F | 691 | 8 | ATG | T | - |  |
| Siganus spinus | trnK-TTT | 7873 | 7947 | F | 75 | 0 | - | - | TTT | Lys |
| Siganus spinus | atp8 | 7949 | 8116 | F | 168 | 1 | ATG | TAA | - |  |
| Siganus spinus | atp6 | 8107 | 8790 | F | 684 | -10 | ATG | TAA | - |  |
| Siganus spinus | cox3 | 8790 | 9575 | F | 786 | -1 | ATG | TAA | - |  |
| Siganus spinus | trnG-TCC | 9575 | 9647 | F | 73 | -1 | - | - | TCC | Gly |
| Siganus spinus | nad3 | 9648 | 9998 | F | 351 | 0 | ATG | TAG | - |  |
| Siganus spinus | trnR-TCG | 9997 | 10064 | F | 68 | -2 | - | - | TCG | Arg |
| Siganus spinus | nad4l | 10065 | 10361 | F | 297 | 0 | ATG | TAA | - |  |
| Siganus spinus | nad4 | 10355 | 11735 | F | 1381 | -7 | ATG | T | - |  |
| Siganus spinus | trnH-GTG | 11736 | 11804 | F | 69 | 0 | - | - | GTG | His |
| Siganus spinus | trnS1-GCT | 11805 | 11872 | F | 68 | 0 | - | - | GCT | Ser1 |
| Siganus spinus | trnL1-TAG | 11880 | 11952 | F | 73 | 7 | - | - | TAG | Leu1 |
| Siganus spinus | nad5 | 11953 | 13791 | F | 1839 | 0 | ATG | TAA | - |  |
| Siganus spinus | nad6 | 13788 | 14309 | R | 522 | -4 | ATG | TAA | - |  |
| Siganus spinus | trnE-TTC | 14310 | 14378 | R | 69 | 0 | - | - | TTC | Glu |
| Siganus spinus | cob | 14383 | 15523 | F | 1141 | 4 | ATG | T | - |  |
| Siganus spinus | trnT-TGT | 15524 | 15595 | F | 72 | 0 | - | - | TGT | Thr |
| Siganus spinus | trnP-TGG | 15595 | 15664 | R | 70 | -1 | - | - | TGG | Pro |
| Siganus spinus | control region | 15665 | 16496 | - | 832 | 0 | - | - | - |  |
| Siganus virgatus | trnF-GAA | 1 | 68 | F | 68 | - | - | - | GAA | Phe |
| Siganus virgatus | rrnS | 69 | 1017 | F | 949 | 0 | - | - | - |  |
| Siganus virgatus | trnV-TAC | 1018 | 1089 | F | 72 | 0 | - | - | TAC | Val |
| Siganus virgatus | rrnL | 1090 | 2782 | F | 1693 | 0 | - | - | - |  |
| Siganus virgatus | trnL-TAA | 2783 | 2856 | F | 74 | 0 | - | - | TAA | Leu2 |
| Siganus virgatus | nad1 | 2857 | 3831 | F | 975 | 0 | ATG | TAA | - |  |
| Siganus virgatus | trnI-GAT | 3836 | 3905 | F | 70 | 4 | - | - | GAT | Ile |
| Siganus virgatus | trnQ-TTG | 3905 | 3975 | R | 71 | -1 | - | - | TTG | Gln |
| Siganus virgatus | trnM-CAT | 3975 | 4043 | F | 69 | -1 | - | - | CAT | Met |
| Siganus virgatus | nad2 | 4044 | 5088 | F | 1045 | 0 | ATG | TAG | - |  |
| Siganus virgatus | trnW-TCA | 5089 | 5160 | F | 72 | 0 | - | - | TCA | Trp |
| Siganus virgatus | trnA-TGC | 5161 | 5229 | R | 69 | 0 | - | - | TGC | Ala |
| Siganus virgatus | trnN-GTT | 5232 | 5304 | R | 73 | 2 | - | - | GTT | Asn |
| Siganus virgatus | trnC-GCA | 5342 | 5407 | R | 66 | 37 | - | - | GCA | Cys |
| Siganus virgatus | trnY-GTA | 5408 | 5478 | R | 71 | 0 | - | - | GTA | Tyr |
| Siganus virgatus | cox1 | 5480 | 7030 | F | 1551 | 1 | GTG | TAG | - |  |
| Siganus virgatus | trnS-TGA | 7033 | 7103 | R | 71 | 2 | - | - | TGA | Ser2 |
| Siganus virgatus | trnD-GTC | 7107 | 7178 | F | 72 | 3 | - | - | GTC | Asp |
| Siganus virgatus | cox2 | 7187 | 7877 | F | 691 | 8 | ATG | T | - |  |
| Siganus virgatus | trnK-TTT | 7878 | 7952 | F | 75 | 0 | - | - | TTT | Lys |
| Siganus virgatus | atp8 | 7954 | 8121 | F | 168 | 1 | ATG | TAA | - |  |
| Siganus virgatus | atp6 | 8112 | 8794 | F | 683 | -10 | ATG | TAA | - |  |
| Siganus virgatus | cox3 | 8795 | 9579 | F | 785 | 0 | ATG | TAA | - |  |
| Siganus virgatus | trnG-TCC | 9580 | 9652 | F | 73 | 0 | - | - | TCC | Gly |
| Siganus virgatus | nad3 | 9653 | 10001 | F | 349 | 0 | ATG | TAG | - |  |
| Siganus virgatus | trnR-TCG | 10002 | 10070 | F | 69 | 0 | - | - | TCG | Arg |
| Siganus virgatus | nad4l | 10071 | 10367 | F | 297 | 0 | ATG | TAA | - |  |
| Siganus virgatus | nad4 | 10361 | 11741 | F | 1381 | -7 | ATG | T | - |  |
| Siganus virgatus | trnH-GTG | 11742 | 11810 | F | 69 | 0 | - | - | GTG | His |
| Siganus virgatus | trnS-GCT | 11811 | 11878 | F | 68 | 0 | - | - | GCT | Ser1 |
| Siganus virgatus | trnL-TAG | 11885 | 11957 | F | 73 | 6 | - | - | TAG | Leu1 |
| Siganus virgatus | nad5 | 11958 | 13796 | F | 1839 | 0 | ATG | TAA | - |  |
| Siganus virgatus | nad6 | 13793 | 14314 | R | 522 | -4 | ATG | TAA | - |  |
| Siganus virgatus | trnE-TTC | 14315 | 14383 | R | 69 | 0 | - | - | TTC | Glu |
| Siganus virgatus | cob | 14389 | 15529 | F | 1141 | 5 | ATG | T | - |  |
| Siganus virgatus | trnT-TGT | 15530 | 15601 | F | 72 | 0 | - | - | TGT | Thr |
| Siganus virgatus | trnP-TGG | 15601 | 15670 | R | 70 | -1 | - | - | TGG | Pro |
| Siganus virgatus | control region | 15671 | 16505 | - | 835 | 0 | - | - | - |  |
| Siganus vulpinus | trnF-GAA | 1 | 68 | F | 68 | - | - | - | GAA | Phe |
| Siganus vulpinus | rrnS | 69 | 1015 | F | 947 | 0 | - | - | - |  |
| Siganus vulpinus | trnV-TAC | 1016 | 1087 | F | 72 | 0 | - | - | TAC | Val |
| Siganus vulpinus | rrnL | 1088 | 2781 | F | 1694 | 0 | - | - | - |  |
| Siganus vulpinus | trnL-TAA | 2782 | 2855 | F | 74 | 0 | - | - | TAA | Leu2 |
| Siganus vulpinus | nad1 | 2856 | 3830 | F | 975 | 0 | ATG | TAA | - |  |
| Siganus vulpinus | trnI-GAT | 3835 | 3904 | F | 70 | 4 | - | - | GAT | Ile |
| Siganus vulpinus | trnQ-TTG | 3904 | 3974 | R | 71 | -1 | - | - | TTG | Gln |
| Siganus vulpinus | trnM-CAT | 3974 | 4042 | F | 69 | -1 | - | - | CAT | Met |
| Siganus vulpinus | nad2 | 4043 | 5087 | F | 1045 | 0 | ATG | TAG | - |  |
| Siganus vulpinus | trnW-TCA | 5088 | 5159 | F | 72 | 0 | - | - | TCA | Trp |
| Siganus vulpinus | trnA-TGC | 5160 | 5228 | R | 69 | 0 | - | - | TGC | Ala |
| Siganus vulpinus | trnN-GTT | 5231 | 5303 | R | 73 | 2 | - | - | GTT | Asn |
| Siganus vulpinus | trnC-GCA | 5341 | 5406 | R | 66 | 37 | - | - | GCA | Cys |
| Siganus vulpinus | trnY-GTA | 5407 | 5477 | R | 71 | 0 | - | - | GTA | Tyr |
| Siganus vulpinus | cox1 | 5479 | 7029 | F | 1551 | 1 | GTG | TAG | - |  |
| Siganus vulpinus | trnS-TGA | 7032 | 7102 | R | 71 | 2 | - | - | TGA | Ser2 |
| Siganus vulpinus | trnD-GTC | 7106 | 7177 | F | 72 | 3 | - | - | GTC | Asp |
| Siganus vulpinus | cox2 | 7187 | 7877 | F | 691 | 9 | ATG | T | - |  |
| Siganus vulpinus | trnK-TTT | 7878 | 7952 | F | 75 | 0 | - | - | TTT | Lys |
| Siganus vulpinus | atp8 | 7954 | 8121 | F | 168 | 1 | ATG | TAA | - |  |
| Siganus vulpinus | atp6 | 8112 | 8794 | F | 683 | -10 | ATG | TAA | - |  |
| Siganus vulpinus | cox3 | 8795 | 9579 | F | 785 | 0 | ATG | TAA | - |  |
| Siganus vulpinus | trnG-TCC | 9580 | 9652 | F | 73 | 0 | - | - | TCC | Gly |
| Siganus vulpinus | nad3 | 9653 | 10001 | F | 349 | 0 | ATG | TAG | - |  |
| Siganus vulpinus | trnR-TCG | 10002 | 10070 | F | 69 | 0 | - | - | TCG | Arg |
| Siganus vulpinus | nad4l | 10071 | 10367 | F | 297 | 0 | ATG | TAA | - |  |
| Siganus vulpinus | nad4 | 10361 | 11741 | F | 1381 | -7 | ATG | T | - |  |
| Siganus vulpinus | trnH-GTG | 11742 | 11810 | F | 69 | 0 | - | - | GTG | His |
| Siganus vulpinus | trnS-GCT | 11811 | 11878 | F | 68 | 0 | - | - | GCT | Ser1 |
| Siganus vulpinus | trnL-TAG | 11885 | 11957 | F | 73 | 6 | - | - | TAG | Leu1 |
| Siganus vulpinus | nad5 | 11958 | 13796 | F | 1839 | 0 | ATG | TAA | - |  |
| Siganus vulpinus | nad6 | 13793 | 14314 | R | 522 | -4 | ATG | TAA | - |  |
| Siganus vulpinus | trnE-TTC | 14315 | 14383 | R | 69 | 0 | - | - | TTC | Glu |
| Siganus vulpinus | cob | 14389 | 15529 | F | 1141 | 5 | ATG | T | - |  |
| Siganus vulpinus | trnT-TGT | 15530 | 15601 | F | 72 | 0 | - | - | TGT | Thr |
| Siganus vulpinus | trnP-TGG | 15601 | 15670 | R | 70 | -1 | - | - | TGG | Pro |
| Siganus vulpinus | control region | 15671 | 16505 | - | 835 | 0 | - | - | - |  |
| Siganus canaliculatus | trnF-GAA | 1 | 68 | F | 68 | - | - | - | GAA | Phe |
| Siganus canaliculatus | rrnS | 69 | 1015 | F | 947 | 0 | - | - | - |  |
| Siganus canaliculatus | trnV-TAC | 1016 | 1087 | F | 72 | 0 | - | - | TAC | Val |
| Siganus canaliculatus | rrnL | 1088 | 2779 | F | 1692 | 0 | - | - | - |  |
| Siganus canaliculatus | trnL-TAA | 2780 | 2853 | F | 74 | 0 | - | - | TAA | Leu2 |
| Siganus canaliculatus | nad1 | 2854 | 3828 | F | 975 | 0 | ATG | TAA | - |  |
| Siganus canaliculatus | trnI-GAT | 3833 | 3902 | F | 70 | 4 | - | - | GAT | Ile |
| Siganus canaliculatus | trnQ-TTG | 3902 | 3972 | R | 71 | -1 | - | - | TTG | Gln |
| Siganus canaliculatus | trnM-CAT | 3972 | 4040 | F | 69 | -1 | - | - | CAT | Met |
| Siganus canaliculatus | nad2 | 4041 | 5085 | F | 1045 | 0 | ATG | TAG | - |  |
| Siganus canaliculatus | trnW-TCA | 5086 | 5157 | F | 72 | 0 | - | - | TCA | Trp |
| Siganus canaliculatus | trnA-TGC | 5158 | 5226 | R | 69 | 0 | - | - | TGC | Ala |
| Siganus canaliculatus | trnN-GTT | 5229 | 5301 | R | 73 | 2 | - | - | GTT | Asn |
| Siganus canaliculatus | trnC-GCA | 5336 | 5401 | R | 66 | 34 | - | - | GCA | Cys |
| Siganus canaliculatus | trnY-GTA | 5402 | 5472 | R | 71 | 0 | - | - | GTA | Tyr |
| Siganus canaliculatus | cox1 | 5474 | 7024 | F | 1551 | 1 | GTG | TAA | - |  |
| Siganus canaliculatus | trnS-TGA | 7027 | 7097 | R | 71 | 2 | - | - | TGA | Ser2 |
| Siganus canaliculatus | trnD-GTC | 7101 | 7172 | F | 72 | 3 | - | - | GTC | Asp |
| Siganus canaliculatus | cox2 | 7181 | 7871 | F | 691 | 8 | ATG | T | - |  |
| Siganus canaliculatus | trnK-TTT | 7872 | 7946 | F | 75 | 0 | - | - | TTT | Lys |
| Siganus canaliculatus | atp8 | 7948 | 8115 | F | 168 | 1 | ATG | TAA | - |  |
| Siganus canaliculatus | atp6 | 8106 | 8787 | F | 682 | -10 | ATG | TAA | - |  |
| Siganus canaliculatus | cox3 | 8789 | 9572 | F | 784 | 1 | ATG | TAA | - |  |
| Siganus canaliculatus | trnG-TCC | 9574 | 9646 | F | 73 | 1 | - | - | TCC | Gly |
| Siganus canaliculatus | nad3 | 9647 | 9995 | F | 349 | 0 | ATG | TAG | - |  |
| Siganus canaliculatus | trnR-TCG | 9996 | 10064 | F | 69 | 0 | - | - | TCG | Arg |
| Siganus canaliculatus | nad4l | 10065 | 10361 | F | 297 | 0 | ATG | TAA | - |  |
| Siganus canaliculatus | nad4 | 10355 | 11735 | F | 1381 | -7 | ATG | T | - |  |
| Siganus canaliculatus | trnH-GTG | 11736 | 11804 | F | 69 | 0 | - | - | GTG | His |
| Siganus canaliculatus | trnS-GCT | 11805 | 11872 | F | 68 | 0 | - | - | GCT | Ser1 |
| Siganus canaliculatus | trnL-TAG | 11880 | 11952 | F | 73 | 7 | - | - | TAG | Leu1 |
| Siganus canaliculatus | nad5 | 11953 | 13791 | F | 1839 | 0 | ATG | TAA | - |  |
| Siganus canaliculatus | nad6 | 13788 | 14309 | R | 522 | -4 | ATG | TAA | - |  |
| Siganus canaliculatus | trnE-TTC | 14310 | 14378 | R | 69 | 0 | - | - | TTC | Glu |
| Siganus canaliculatus | cob | 14383 | 15523 | F | 1141 | 4 | ATG | T | - |  |
| Siganus canaliculatus | trnT-TGT | 15526 | 15597 | F | 72 | 2 | - | - | TGT | Thr |
| Siganus canaliculatus | trnP-TGG | 15595 | 15663 | R | 69 | -3 | - | - | TGG | Pro |
| Siganus canaliculatus | control region | 15664 | 16492 | - | 829 | 0 | - | - | - |  |
| Siganus guttatus | trnF-GAA | 1 | 68 | F | 68 | - | - | - | GAA | Phe |
| Siganus guttatus | rrnS | 69 | 1017 | F | 949 | 0 | - | - | - |  |
| Siganus guttatus | trnV-TAC | 1018 | 1089 | F | 72 | 0 | - | - | TAC | Val |
| Siganus guttatus | rrnL | 1090 | 2781 | F | 1692 | 0 | - | - | - |  |
| Siganus guttatus | trnL-TAA | 2782 | 2855 | F | 74 | 0 | - | - | TAA | Leu2 |
| Siganus guttatus | nad1 | 2856 | 3830 | F | 975 | 0 | ATG | TAA | - |  |
| Siganus guttatus | trnI-GAT | 3835 | 3904 | F | 70 | 4 | - | - | GAT | Ile |
| Siganus guttatus | trnQ-TTG | 3904 | 3974 | R | 71 | -1 | - | - | TTG | Gln |
| Siganus guttatus | trnM-CAT | 3974 | 4042 | F | 69 | -1 | - | - | CAT | Met |
| Siganus guttatus | nad2 | 4043 | 5087 | F | 1045 | 0 | ATG | TAG | - |  |
| Siganus guttatus | trnW-TCA | 5088 | 5159 | F | 72 | 0 | - | - | TCA | Trp |
| Siganus guttatus | trnA-TGC | 5160 | 5228 | R | 69 | 0 | - | - | TGC | Ala |
| Siganus guttatus | trnN-GTT | 5231 | 5303 | R | 73 | 2 | - | - | GTT | Asn |
| Siganus guttatus | trnC-GCA | 5341 | 5406 | R | 66 | 37 | - | - | GCA | Cys |
| Siganus guttatus | trnY-GTA | 5407 | 5477 | R | 71 | 0 | - | - | GTA | Tyr |
| Siganus guttatus | cox1 | 5479 | 7029 | F | 1551 | 1 | GTG | TAG | - |  |
| Siganus guttatus | trnS-TGA | 7032 | 7102 | R | 71 | 2 | - | - | TGA | Ser2 |
| Siganus guttatus | trnD-GTC | 7106 | 7177 | F | 72 | 3 | - | - | GTC | Asp |
| Siganus guttatus | cox2 | 7186 | 7876 | F | 691 | 8 | ATG | T | - |  |
| Siganus guttatus | trnK-TTT | 7877 | 7951 | F | 75 | 0 | - | - | TTT | Lys |
| Siganus guttatus | atp8 | 7953 | 8120 | F | 168 | 1 | ATG | TAA | - |  |
| Siganus guttatus | atp6 | 8111 | 8793 | F | 683 | -10 | ATG | TAA | - |  |
| Siganus guttatus | cox3 | 8794 | 9578 | F | 785 | 0 | ATG | TAA | - |  |
| Siganus guttatus | trnG-TCC | 9579 | 9651 | F | 73 | 0 | - | - | TCC | Gly |
| Siganus guttatus | nad3 | 9652 | 10000 | F | 349 | 0 | ATG | TAG | - |  |
| Siganus guttatus | trnR-TCG | 10001 | 10069 | F | 69 | 0 | - | - | TCG | Arg |
| Siganus guttatus | nad4l | 10070 | 10366 | F | 297 | 0 | ATG | TAA | - |  |
| Siganus guttatus | nad4 | 10360 | 11740 | F | 1381 | -7 | ATG | T | - |  |
| Siganus guttatus | trnH-GTG | 11741 | 11809 | F | 69 | 0 | - | - | GTG | His |
| Siganus guttatus | trnS-GCT | 11810 | 11877 | F | 68 | 0 | - | - | GCT | Ser1 |
| Siganus guttatus | trnL-TAG | 11884 | 11956 | F | 73 | 6 | - | - | TAG | Leu1 |
| Siganus guttatus | nad5 | 11957 | 13795 | F | 1839 | 0 | ATG | TAA | - |  |
| Siganus guttatus | nad6 | 13792 | 14313 | R | 522 | -4 | ATG | TAA | - |  |
| Siganus guttatus | trnE-TTC | 14314 | 14382 | R | 69 | 0 | - | - | TTC | Glu |
| Siganus guttatus | cob | 14388 | 15528 | F | 1141 | 5 | ATG | T | - |  |
| Siganus guttatus | trnT-TGT | 15529 | 15600 | F | 72 | 0 | - | - | TGT | Thr |
| Siganus guttatus | trnP-TGG | 15600 | 15669 | R | 70 | -1 | - | - | TGG | Pro |
| Siganus guttatus | control region | 15670 | 16505 | - | 836 | 0 | - | - | - |  |
| Siganus puellus | trnF-GAA | 1 | 68 | F | 68 | - | - | - | GAA | Phe |
| Siganus puellus | rrnS | 69 | 1015 | F | 947 | 0 | - | - | - |  |
| Siganus puellus | trnV-TAC | 1016 | 1087 | F | 72 | 0 | - | - | TAC | Val |
| Siganus puellus | rrnL | 1088 | 2783 | F | 1696 | 0 | - | - | - |  |
| Siganus puellus | trnL-TAA | 2784 | 2857 | F | 74 | 0 | - | - | TAA | Leu2 |
| Siganus puellus | nad1 | 2858 | 3832 | F | 975 | 0 | ATG | TAA | - |  |
| Siganus puellus | trnI-GAT | 3837 | 3906 | F | 70 | 4 | - | - | GAT | Ile |
| Siganus puellus | trnQ-TTG | 3906 | 3976 | R | 71 | -1 | - | - | TTG | Gln |
| Siganus puellus | trnM-CAT | 3976 | 4044 | F | 69 | -1 | - | - | CAT | Met |
| Siganus puellus | nad2 | 4045 | 5089 | F | 1045 | 0 | ATG | TAG | - |  |
| Siganus puellus | trnW-TCA | 5090 | 5161 | F | 72 | 0 | - | - | TCA | Trp |
| Siganus puellus | trnA-TGC | 5162 | 5230 | R | 69 | 0 | - | - | TGC | Ala |
| Siganus puellus | trnN-GTT | 5233 | 5305 | R | 73 | 2 | - | - | GTT | Asn |
| Siganus puellus | trnC-GCA | 5343 | 5408 | R | 66 | 37 | - | - | GCA | Cys |
| Siganus puellus | trnY-GTA | 5409 | 5479 | R | 71 | 0 | - | - | GTA | Tyr |
| Siganus puellus | cox1 | 5481 | 7031 | F | 1551 | 1 | GTG | TAG | - |  |
| Siganus puellus | trnS-TGA | 7034 | 7104 | R | 71 | 2 | - | - | TGA | Ser2 |
| Siganus puellus | trnD-GTC | 7108 | 7179 | F | 72 | 3 | - | - | GTC | Asp |
| Siganus puellus | cox2 | 7189 | 7879 | F | 691 | 9 | ATG | T | - |  |
| Siganus puellus | trnK-TTT | 7880 | 7954 | F | 75 | 0 | - | - | TTT | Lys |
| Siganus puellus | atp8 | 7956 | 8123 | F | 168 | 1 | ATG | TAA | - |  |
| Siganus puellus | atp6 | 8114 | 8796 | F | 683 | -10 | ATG | TAA | - |  |
| Siganus puellus | cox3 | 8797 | 9581 | F | 785 | 0 | ATG | TAA | - |  |
| Siganus puellus | trnG-TCC | 9582 | 9654 | F | 73 | 0 | - | - | TCC | Gly |
| Siganus puellus | nad3 | 9655 | 10003 | F | 349 | 0 | ATG | TAG | - |  |
| Siganus puellus | trnR-TCG | 10004 | 10072 | F | 69 | 0 | - | - | TCG | Arg |
| Siganus puellus | nad4l | 10073 | 10369 | F | 297 | 0 | ATG | TAA | - |  |
| Siganus puellus | nad4 | 10363 | 11743 | F | 1381 | -7 | ATG | T | - |  |
| Siganus puellus | trnH-GTG | 11744 | 11812 | F | 69 | 0 | - | - | GTG | His |
| Siganus puellus | trnS-GCT | 11813 | 11880 | F | 68 | 0 | - | - | GCT | Ser1 |
| Siganus puellus | trnL-TAG | 11887 | 11959 | F | 73 | 6 | - | - | TAG | Leu1 |
| Siganus puellus | nad5 | 11960 | 13798 | F | 1839 | 0 | ATG | TAA | - |  |
| Siganus puellus | nad6 | 13795 | 14316 | R | 522 | -4 | ATG | TAA | - |  |
| Siganus puellus | trnE-TTC | 14317 | 14385 | R | 69 | 0 | - | - | TTC | Glu |
| Siganus puellus | cob | 14391 | 15531 | F | 1141 | 5 | ATG | T | - |  |
| Siganus puellus | trnT-TGT | 15532 | 15603 | F | 72 | 0 | - | - | TGT | Thr |
| Siganus puellus | trnP-TGG | 15603 | 15672 | R | 70 | -1 | - | - | TGG | Pro |
| Siganus puellus | control region | 15673 | 16504 | - | 832 | 0 | - | - | - |  |
| Siganus fuscescens | trnF-GAA | 1 | 68 | F | 68 | - | - | - | GAA | Phe |
| Siganus fuscescens | rrnS | 69 | 1015 | F | 947 | 0 | - | - | - |  |
| Siganus fuscescens | trnV-TAC | 1016 | 1087 | F | 72 | 0 | - | - | TAC | Val |
| Siganus fuscescens | rrnL | 1088 | 2778 | F | 1691 | 0 | - | - | - |  |
| Siganus fuscescens | trnL-TAA | 2779 | 2852 | F | 74 | 0 | - | - | TAA | Leu2 |
| Siganus fuscescens | nad1 | 2853 | 3827 | F | 975 | 0 | ATG | TAA | - |  |
| Siganus fuscescens | trnI-GAT | 3832 | 3901 | F | 70 | 4 | - | - | GAT | Ile |
| Siganus fuscescens | trnQ-TTG | 3901 | 3971 | R | 71 | -1 | - | - | TTG | Gln |
| Siganus fuscescens | trnM-CAT | 3971 | 4039 | F | 69 | -1 | - | - | CAT | Met |
| Siganus fuscescens | nad2 | 4040 | 5084 | F | 1045 | 0 | ATG | TAG | - |  |
| Siganus fuscescens | trnW-TCA | 5085 | 5156 | F | 72 | 0 | - | - | TCA | Trp |
| Siganus fuscescens | trnA-TGC | 5157 | 5225 | R | 69 | 0 | - | - | TGC | Ala |
| Siganus fuscescens | trnN-GTT | 5228 | 5300 | R | 73 | 2 | - | - | GTT | Asn |
| Siganus fuscescens | trnC-GCA | 5335 | 5400 | R | 66 | 34 | - | - | GCA | Cys |
| Siganus fuscescens | trnY-GTA | 5401 | 5471 | R | 71 | 0 | - | - | GTA | Tyr |
| Siganus fuscescens | cox1 | 5473 | 7023 | F | 1551 | 1 | GTG | TAA | - |  |
| Siganus fuscescens | trnS-TGA | 7026 | 7096 | R | 71 | 2 | - | - | TGA | Ser2 |
| Siganus fuscescens | trnD-GTC | 7100 | 7171 | F | 72 | 3 | - | - | GTC | Asp |
| Siganus fuscescens | cox2 | 7180 | 7870 | F | 691 | 8 | ATG | T | - |  |
| Siganus fuscescens | trnK-TTT | 7871 | 7945 | F | 75 | 0 | - | - | TTT | Lys |
| Siganus fuscescens | atp8 | 7947 | 8114 | F | 168 | 1 | ATG | TAA | - |  |
| Siganus fuscescens | atp6 | 8105 | 8786 | F | 682 | -10 | ATG | TAA | - |  |
| Siganus fuscescens | cox3 | 8788 | 9571 | F | 784 | 1 | ATG | TAA | - |  |
| Siganus fuscescens | trnG-TCC | 9573 | 9645 | F | 73 | 1 | - | - | TCC | Gly |
| Siganus fuscescens | nad3 | 9646 | 9994 | F | 349 | 0 | ATG | TAG | - |  |
| Siganus fuscescens | trnR-TCG | 9995 | 10063 | F | 69 | 0 | - | - | TCG | Arg |
| Siganus fuscescens | nad4l | 10064 | 10360 | F | 297 | 0 | ATG | TAA | - |  |
| Siganus fuscescens | nad4 | 10354 | 11734 | F | 1381 | -7 | ATG | T | - |  |
| Siganus fuscescens | trnH-GTG | 11735 | 11803 | F | 69 | 0 | - | - | GTG | His |
| Siganus fuscescens | trnS-GCT | 11804 | 11871 | F | 68 | 0 | - | - | GCT | Ser1 |
| Siganus fuscescens | trnL-TAG | 11879 | 11951 | F | 73 | 7 | - | - | TAG | Leu1 |
| Siganus fuscescens | nad5 | 11952 | 13790 | F | 1839 | 0 | ATG | TAA | - |  |
| Siganus fuscescens | nad6 | 13787 | 14308 | R | 522 | -4 | ATG | TAA | - |  |
| Siganus fuscescens | trnE-TTC | 14309 | 14377 | R | 69 | 0 | - | - | TTC | Glu |
| Siganus fuscescens | cob | 14382 | 15522 | F | 1141 | 4 | ATG | T | - |  |
| Siganus fuscescens | trnT-TGT | 15523 | 15594 | F | 72 | 0 | - | - | TGT | Thr |
| Siganus fuscescens | trnP-TGG | 15594 | 15663 | R | 70 | -1 | - | - | TGG | Pro |
| Siganus fuscescens | control region | 15664 | 16491 | - | 828 | 0 | - | - | - |  |
| Siganus sutor | trnF-GAA | 1 | 68 | F | 68 | - | - | - | GAA | Phe |
| Siganus sutor | rrnS | 69 | 1015 | F | 947 | 0 | - | - | - |  |
| Siganus sutor | trnV-TAC | 1016 | 1087 | F | 72 | 0 | - | - | TAC | Val |
| Siganus sutor | rrnL | 1088 | 2779 | F | 1692 | 0 | - | - | - |  |
| Siganus sutor | trnL-TAA | 2780 | 2853 | F | 74 | 0 | - | - | TAA | Leu2 |
| Siganus sutor | nad1 | 2854 | 3828 | F | 975 | 0 | ATG | TAA | - |  |
| Siganus sutor | trnI-GAT | 3833 | 3902 | F | 70 | 4 | - | - | GAT | Ile |
| Siganus sutor | trnQ-TTG | 3902 | 3972 | R | 71 | -1 | - | - | TTG | Gln |
| Siganus sutor | trnM-CAT | 3972 | 4040 | F | 69 | -1 | - | - | CAT | Met |
| Siganus sutor | nad2 | 4041 | 5085 | F | 1045 | 0 | ATG | TAG | - |  |
| Siganus sutor | trnW-TCA | 5086 | 5157 | F | 72 | 0 | - | - | TCA | Trp |
| Siganus sutor | trnA-TGC | 5158 | 5226 | R | 69 | 0 | - | - | TGC | Ala |
| Siganus sutor | trnN-GTT | 5229 | 5301 | R | 73 | 2 | - | - | GTT | Asn |
| Siganus sutor | trnC-GCA | 5338 | 5403 | R | 66 | 36 | - | - | GCA | Cys |
| Siganus sutor | trnY-GTA | 5404 | 5474 | R | 71 | 0 | - | - | GTA | Tyr |
| Siganus sutor | cox1 | 5476 | 7026 | F | 1551 | 1 | GTG | TAA | - |  |
| Siganus sutor | trnS-TGA | 7029 | 7099 | R | 71 | 2 | - | - | TGA | Ser2 |
| Siganus sutor | trnD-GTC | 7103 | 7174 | F | 72 | 3 | - | - | GTC | Asp |
| Siganus sutor | cox2 | 7183 | 7873 | F | 691 | 8 | ATG | T | - |  |
| Siganus sutor | trnK-TTT | 7874 | 7948 | F | 75 | 0 | - | - | TTT | Lys |
| Siganus sutor | atp8 | 7950 | 8117 | F | 168 | 1 | ATG | TAA | - |  |
| Siganus sutor | atp6 | 8108 | 8791 | F | 684 | -10 | ATG | TAA | - |  |
| Siganus sutor | cox3 | 8791 | 9575 | F | 785 | -1 | ATG | TAA | - |  |
| Siganus sutor | trnG-TCC | 9576 | 9648 | F | 73 | 0 | - | - | TCC | Gly |
| Siganus sutor | nad3 | 9649 | 9997 | F | 349 | 0 | ATG | TAG | - |  |
| Siganus sutor | trnR-TCG | 9998 | 10066 | F | 69 | 0 | - | - | TCG | Arg |
| Siganus sutor | nad4l | 10067 | 10363 | F | 297 | 0 | ATG | TAA | - |  |
| Siganus sutor | nad4 | 10357 | 11737 | F | 1381 | -7 | ATG | T | - |  |
| Siganus sutor | trnH-GTG | 11738 | 11806 | F | 69 | 0 | - | - | GTG | His |
| Siganus sutor | trnS-GCT | 11807 | 11874 | F | 68 | 0 | - | - | GCT | Ser1 |
| Siganus sutor | trnL-TAG | 11882 | 11954 | F | 73 | 7 | - | - | TAG | Leu1 |
| Siganus sutor | nad5 | 11955 | 13793 | F | 1839 | 0 | ATG | TAA | - |  |
| Siganus sutor | nad6 | 13790 | 14311 | R | 522 | -4 | ATG | TAA | - |  |
| Siganus sutor | trnE-TTC | 14312 | 14380 | R | 69 | 0 | - | - | TTC | Glu |
| Siganus sutor | cob | 14385 | 15525 | F | 1141 | 4 | ATG | T | - |  |
| Siganus sutor | trnT-TGT | 15526 | 15597 | F | 72 | 0 | - | - | TGT | Thr |
| Siganus sutor | trnP-TGG | 15597 | 15666 | R | 70 | -1 | - | - | TGG | Pro |
| Siganus sutor | control region | 15667 | 16497 | - | 831 | 0 | - | - | - |  |
| Siganus unimaculatus | trnF-GAA | 1 | 68 | F | 68 | - | - | - | GAA | Phe |
| Siganus unimaculatus | rrnS | 69 | 1015 | F | 947 | 0 | - | - | - |  |
| Siganus unimaculatus | trnV-TAC | 1016 | 1087 | F | 72 | 0 | - | - | TAC | Val |
| Siganus unimaculatus | rrnL | 1088 | 2781 | F | 1694 | 0 | - | - | - |  |
| Siganus unimaculatus | trnL-TAA | 2782 | 2855 | F | 74 | 0 | - | - | TAA | Leu2 |
| Siganus unimaculatus | nad1 | 2856 | 3830 | F | 975 | 0 | ATG | TAA | - |  |
| Siganus unimaculatus | trnI-GAT | 3835 | 3904 | F | 70 | 4 | - | - | GAT | Ile |
| Siganus unimaculatus | trnQ-TTG | 3904 | 3974 | R | 71 | -1 | - | - | TTG | Gln |
| Siganus unimaculatus | trnM-CAT | 3974 | 4042 | F | 69 | -1 | - | - | CAT | Met |
| Siganus unimaculatus | nad2 | 4043 | 5087 | F | 1045 | 0 | ATG | TAG | - |  |
| Siganus unimaculatus | trnW-TCA | 5088 | 5159 | F | 72 | 0 | - | - | TCA | Trp |
| Siganus unimaculatus | trnA-TGC | 5160 | 5228 | R | 69 | 0 | - | - | TGC | Ala |
| Siganus unimaculatus | trnN-GTT | 5231 | 5303 | R | 73 | 2 | - | - | GTT | Asn |
| Siganus unimaculatus | trnC-GCA | 5341 | 5406 | R | 66 | 37 | - | - | GCA | Cys |
| Siganus unimaculatus | trnY-GTA | 5407 | 5477 | R | 71 | 0 | - | - | GTA | Tyr |
| Siganus unimaculatus | cox1 | 5479 | 7029 | F | 1551 | 1 | GTG | TAG | - |  |
| Siganus unimaculatus | trnS-TGA | 7032 | 7102 | R | 71 | 2 | - | - | TGA | Ser2 |
| Siganus unimaculatus | trnD-GTC | 7106 | 7177 | F | 72 | 3 | - | - | GTC | Asp |
| Siganus unimaculatus | cox2 | 7187 | 7877 | F | 691 | 9 | ATG | T | - |  |
| Siganus unimaculatus | trnK-TTT | 7878 | 7952 | F | 75 | 0 | - | - | TTT | Lys |
| Siganus unimaculatus | atp8 | 7954 | 8121 | F | 168 | 1 | ATG | TAA | - |  |
| Siganus unimaculatus | atp6 | 8112 | 8794 | F | 683 | -10 | ATG | TAA | - |  |
| Siganus unimaculatus | cox3 | 8795 | 9579 | F | 785 | 0 | ATG | TAA | - |  |
| Siganus unimaculatus | trnG-TCC | 9580 | 9652 | F | 73 | 0 | - | - | TCC | Gly |
| Siganus unimaculatus | nad3 | 9653 | 10001 | F | 349 | 0 | ATG | TAG | - |  |
| Siganus unimaculatus | trnR-TCG | 10002 | 10070 | F | 69 | 0 | - | - | TCG | Arg |
| Siganus unimaculatus | nad4l | 10071 | 10367 | F | 297 | 0 | ATG | TAA | - |  |
| Siganus unimaculatus | nad4 | 10361 | 11741 | F | 1381 | -7 | ATG | T | - |  |
| Siganus unimaculatus | trnH-GTG | 11742 | 11810 | F | 69 | 0 | - | - | GTG | His |
| Siganus unimaculatus | trnS-GCT | 11811 | 11878 | F | 68 | 0 | - | - | GCT | Ser1 |
| Siganus unimaculatus | trnL-TAG | 11885 | 11957 | F | 73 | 6 | - | - | TAG | Leu1 |
| Siganus unimaculatus | nad5 | 11958 | 13796 | F | 1839 | 0 | ATG | TAA | - |  |
| Siganus unimaculatus | nad6 | 13793 | 14314 | R | 522 | -4 | ATG | TAA | - |  |
| Siganus unimaculatus | trnE-TTC | 14315 | 14383 | R | 69 | 0 | - | - | TTC | Glu |
| Siganus unimaculatus | cob | 14389 | 15529 | F | 1141 | 5 | ATG | T | - |  |
| Siganus unimaculatus | trnT-TGT | 15530 | 15601 | F | 72 | 0 | - | - | TGT | Thr |
| Siganus unimaculatus | trnP-TGG | 15601 | 15670 | R | 70 | -1 | - | - | TGG | Pro |
| Siganus unimaculatus | control region | 15671 | 16505 | - | 835 | 0 | - | - | - |  |
| Siganus rivulatus | trnF-GAA | 1 | 68 | F | 68 | - | - | - | GAA | Phe |
| Siganus rivulatus | rrnS | 69 | 1014 | F | 946 | 0 | - | - | - |  |
| Siganus rivulatus | trnV-TAC | 1015 | 1086 | F | 72 | 0 | - | - | TAC | Val |
| Siganus rivulatus | rrnL | 1087 | 2778 | F | 1692 | 0 | - | - | - |  |
| Siganus rivulatus | trnL-TAA | 2779 | 2852 | F | 74 | 0 | - | - | TAA | Leu2 |
| Siganus rivulatus | nad1 | 2853 | 3827 | F | 975 | 0 | ATG | TAA | - |  |
| Siganus rivulatus | trnI-GAT | 3832 | 3901 | F | 70 | 4 | - | - | GAT | Ile |
| Siganus rivulatus | trnQ-TTG | 3901 | 3971 | R | 71 | -1 | - | - | TTG | Gln |
| Siganus rivulatus | trnM-CAT | 3971 | 4039 | F | 69 | -1 | - | - | CAT | Met |
| Siganus rivulatus | nad2 | 4040 | 5084 | F | 1045 | 0 | ATG | TAG | - |  |
| Siganus rivulatus | trnW-TCA | 5085 | 5156 | F | 72 | 0 | - | - | TCA | Trp |
| Siganus rivulatus | trnA-TGC | 5157 | 5225 | R | 69 | 0 | - | - | TGC | Ala |
| Siganus rivulatus | trnN-GTT | 5228 | 5300 | R | 73 | 2 | - | - | GTT | Asn |
| Siganus rivulatus | trnC-GCA | 5337 | 5402 | R | 66 | 36 | - | - | GCA | Cys |
| Siganus rivulatus | trnY-GTA | 5403 | 5473 | R | 71 | 0 | - | - | GTA | Tyr |
| Siganus rivulatus | cox1 | 5475 | 7025 | F | 1551 | 1 | GTG | TAA | - |  |
| Siganus rivulatus | trnS-TGA | 7028 | 7098 | R | 71 | 2 | - | - | TGA | Ser2 |
| Siganus rivulatus | trnD-GTC | 7102 | 7173 | F | 72 | 3 | - | - | GTC | Asp |
| Siganus rivulatus | cox2 | 7182 | 7872 | F | 691 | 8 | ATG | T | - |  |
| Siganus rivulatus | trnK-TTT | 7873 | 7947 | F | 75 | 0 | - | - | TTT | Lys |
| Siganus rivulatus | atp8 | 7949 | 8116 | F | 168 | 1 | ATG | TAA | - |  |
| Siganus rivulatus | atp6 | 8107 | 8790 | F | 684 | -10 | ATG | TAA | - |  |
| Siganus rivulatus | cox3 | 8790 | 9574 | F | 785 | -1 | ATG | TAA | - |  |
| Siganus rivulatus | trnG-TCC | 9575 | 9647 | F | 73 | 0 | - | - | TCC | Gly |
| Siganus rivulatus | nad3 | 9648 | 9996 | F | 349 | 0 | ATG | TAG | - |  |
| Siganus rivulatus | trnR-TCG | 9997 | 10065 | F | 69 | 0 | - | - | TCG | Arg |
| Siganus rivulatus | nad4l | 10066 | 10362 | F | 297 | 0 | ATG | TAA | - |  |
| Siganus rivulatus | nad4 | 10356 | 11736 | F | 1381 | -7 | ATG | T | - |  |
| Siganus rivulatus | trnH-GTG | 11737 | 11805 | F | 69 | 0 | - | - | GTG | His |
| Siganus rivulatus | trnS-GCT | 11806 | 11873 | F | 68 | 0 | - | - | GCT | Ser1 |
| Siganus rivulatus | trnL-TAG | 11881 | 11953 | F | 73 | 7 | - | - | TAG | Leu1 |
| Siganus rivulatus | nad5 | 11954 | 13792 | F | 1839 | 0 | ATG | TAA | - |  |
| Siganus rivulatus | nad6 | 13789 | 14310 | R | 522 | -4 | ATG | TAA | - |  |
| Siganus rivulatus | trnE-TTC | 14311 | 14379 | R | 69 | 0 | - | - | TTC | Glu |
| Siganus rivulatus | cob | 14384 | 15524 | F | 1141 | 4 | ATG | T | - |  |
| Siganus rivulatus | trnT-TGT | 15525 | 15596 | F | 72 | 0 | - | - | TGT | Thr |
| Siganus rivulatus | trnP-TGG | 15596 | 15665 | R | 70 | -1 | - | - | TGG | Pro |
| Siganus rivulatus | control region | 15666 | 16495 | - | 830 | 0 | - | - | - |  |
| Siganus luridus | trnF-GAA | 1 | 68 | F | 68 | - | - | - | GAA | Phe |
| Siganus luridus | rrnS | 69 | 1014 | F | 946 | 0 | - | - | - |  |
| Siganus luridus | trnV-TAC | 1015 | 1086 | F | 72 | 0 | - | - | TAC | Val |
| Siganus luridus | rrnL | 1087 | 2778 | F | 1692 | 0 | - | - | - |  |
| Siganus luridus | trnL-TAA | 2779 | 2852 | F | 74 | 0 | - | - | TAA | Leu2 |
| Siganus luridus | nad1 | 2853 | 3827 | F | 975 | 0 | ATG | TAA | - |  |
| Siganus luridus | trnI-GAT | 3832 | 3901 | F | 70 | 4 | - | - | GAT | Ile |
| Siganus luridus | trnQ-TTG | 3902 | 3971 | R | 70 | 0 | - | - | TTG | Gln |
| Siganus luridus | trnM-CAT | 3971 | 4039 | F | 69 | -1 | - | - | CAT | Met |
| Siganus luridus | nad2 | 4040 | 5084 | F | 1045 | 0 | ATG | TAG | - |  |
| Siganus luridus | trnW-TCA | 5085 | 5156 | F | 72 | 0 | - | - | TCA | Trp |
| Siganus luridus | trnA-TGC | 5157 | 5225 | R | 69 | 0 | - | - | TGC | Ala |
| Siganus luridus | trnN-GTT | 5228 | 5300 | R | 73 | 2 | - | - | GTT | Asn |
| Siganus luridus | trnC-GCA | 5337 | 5402 | R | 66 | 36 | - | - | GCA | Cys |
| Siganus luridus | trnY-GTA | 5403 | 5473 | R | 71 | 0 | - | - | GTA | Tyr |
| Siganus luridus | cox1 | 5475 | 7025 | F | 1551 | 1 | GTG | TAA | - |  |
| Siganus luridus | trnS-TGA | 7028 | 7098 | R | 71 | 2 | - | - | TGA | Ser2 |
| Siganus luridus | trnD-GTC | 7102 | 7173 | F | 72 | 3 | - | - | GTC | Asp |
| Siganus luridus | cox2 | 7182 | 7872 | F | 691 | 8 | ATG | T | - |  |
| Siganus luridus | trnK-TTT | 7873 | 7947 | F | 75 | 0 | - | - | TTT | Lys |
| Siganus luridus | atp8 | 7949 | 8116 | F | 168 | 1 | ATG | TAA | - |  |
| Siganus luridus | atp6 | 8107 | 8790 | F | 684 | -10 | ATG | TAA | - |  |
| Siganus luridus | cox3 | 8790 | 9574 | F | 785 | -1 | ATG | TAA | - |  |
| Siganus luridus | trnG-TCC | 9575 | 9647 | F | 73 | 0 | - | - | TCC | Gly |
| Siganus luridus | nad3 | 9648 | 9996 | F | 349 | 0 | ATG | TAG | - |  |
| Siganus luridus | trnR-TCG | 9997 | 10065 | F | 69 | 0 | - | - | TCG | Arg |
| Siganus luridus | nad4l | 10066 | 10362 | F | 297 | 0 | ATG | TAA | - |  |
| Siganus luridus | nad4 | 10356 | 11736 | F | 1381 | -7 | ATG | T | - |  |
| Siganus luridus | trnH-GTG | 11737 | 11805 | F | 69 | 0 | - | - | GTG | His |
| Siganus luridus | trnS-GCT | 11806 | 11873 | F | 68 | 0 | - | - | GCT | Ser1 |
| Siganus luridus | trnL-TAG | 11881 | 11953 | F | 73 | 7 | - | - | TAG | Leu1 |
| Siganus luridus | nad5 | 11954 | 13792 | F | 1839 | 0 | ATG | TAA | - |  |
| Siganus luridus | nad6 | 13789 | 14310 | R | 522 | -4 | ATG | TAA | - |  |
| Siganus luridus | trnE-TTC | 14311 | 14379 | R | 69 | 0 | - | - | TTC | Glu |
| Siganus luridus | cob | 14384 | 15524 | F | 1141 | 4 | ATG | T | - |  |
| Siganus luridus | trnT-TGT | 15525 | 15596 | F | 72 | 0 | - | - | TGT | Thr |
| Siganus luridus | trnP-TGG | 15596 | 15665 | F | 70 | -1 | - | - | TGG | Pro |
| Siganus luridus | control region | 15666 | 16495 | - | 830 | 0 | - | - | - |  |
