# Supplementary material for: Comparative Mitogenome Analyses Uncover Mitogenome Features and Phylogenetic Implications of the Rabbitfishes (Perciformes: Siganidae)
Source: Ecol Evol. 2026 Aug 14;16(8):e74188. doi: 10.1002/ece3.74188 (PMC13476152; doi:10.1002/ece3.74188)
Supplement: Supplementary file 8 [file ECE3-16-e74188-s008.docx]

Table S4. Relative synonymous codon usage (RSCU) values of mitochondrial protein-coding genes in the 14 sampled siganid mitogenomes.

| **Amino**  **acid** | **Codon** | **Siganus virgatus** | **Siganus vulpinus** | **Siganus canaliculatus** | **Siganus guttatus** | **Siganus puellus** | **Siganus fuscescens** | **Siganus sutor** | **Siganus unimaculatus** | **Siganus rivulatus** | **Siganus luridus** | **Siganus argenteus** | **Siganus corallinus** | **Siganus punctatus** | **Siganus spinus** |
| --- | --- | --- | --- | --- | --- | --- | --- | --- | --- | --- | --- | --- | --- | --- | --- |
| Phe | UUU | 0.85 | 0.85 | 0.64 | 0.95 | 0.84 | 0.65 | 0.71 | 0.83 | 0.68 | 0.73 | 0.9 | 0.88 | 0.91 | 1.05 |
|  | UUC | 1.15 | 1.15 | 1.36 | 1.05 | 1.16 | 1.35 | 1.29 | 1.17 | 1.32 | 1.27 | 1.1 | 1.12 | 1.09 | 0.95 |
| Leu | UUA | 1.03 | 1 | 0.91 | 1.01 | 1.04 | 0.97 | 1.08 | 1.02 | 1.05 | 0.99 | 0.83 | 0.91 | 0.89 | 0.8 |
|  | UUG | 0.66 | 0.68 | 0.49 | 0.64 | 0.6 | 0.46 | 0.48 | 0.68 | 0.5 | 0.52 | 0.64 | 0.61 | 0.59 | 0.69 |
|  | CUU | 1.39 | 1.38 | 1.09 | 1.36 | 1.35 | 1.11 | 0.93 | 1.4 | 0.99 | 1.04 | 1.49 | 1.39 | 1.58 | 1.55 |
|  | CUC | 1.16 | 1.13 | 1.41 | 1.17 | 1.03 | 1.37 | 1.42 | 1.12 | 1.43 | 1.4 | 1.1 | 1.11 | 0.98 | 1.02 |
|  | CUA | 1.13 | 1.16 | 1.59 | 1.13 | 1.21 | 1.51 | 1.4 | 1.14 | 1.39 | 1.33 | 1.31 | 1.43 | 1.38 | 1.2 |
|  | CUG | 0.63 | 0.64 | 0.51 | 0.7 | 0.76 | 0.58 | 0.7 | 0.65 | 0.63 | 0.72 | 0.63 | 0.55 | 0.58 | 0.74 |
| Ile | AUU | 1.3 | 1.28 | 1.1 | 1.24 | 1.25 | 1.12 | 1.2 | 1.27 | 1.02 | 1.23 | 1.54 | 1.38 | 1.35 | 1.48 |
|  | AUC | 0.93 | 0.89 | 1.18 | 0.97 | 0.97 | 1.2 | 1.14 | 0.89 | 1.35 | 1.09 | 0.92 | 0.95 | 1.03 | 0.87 |
|  | AUA | 0.77 | 0.82 | 0.72 | 0.79 | 0.78 | 0.67 | 0.66 | 0.84 | 0.63 | 0.68 | 0.54 | 0.67 | 0.62 | 0.65 |
| Met | AUG | 1 | 1 | 1 | 1 | 1 | 1 | 1 | 1 | 1 | 1 | 1 | 1 | 1 | 1 |
| Val | GUU | 1.13 | 1.33 | 1.06 | 1.17 | 1.19 | 1.07 | 1.05 | 1.33 | 1.27 | 1.15 | 1.02 | 1.28 | 1.26 | 1.02 |
|  | GUC | 1.01 | 1.03 | 1.25 | 0.98 | 0.96 | 1.2 | 1.29 | 1.03 | 1.06 | 1.15 | 1.18 | 0.84 | 0.94 | 1.09 |
|  | GUA | 1.25 | 0.97 | 1.17 | 1.17 | 1.25 | 1.2 | 1.05 | 0.97 | 1.06 | 1.03 | 1.22 | 1.47 | 1.3 | 1.47 |
|  | GUG | 0.6 | 0.67 | 0.52 | 0.68 | 0.6 | 0.52 | 0.6 | 0.67 | 0.6 | 0.67 | 0.58 | 0.4 | 0.5 | 0.42 |
| Ser | UCU | 1.33 | 1.29 | 1.34 | 1.35 | 1.45 | 1.31 | 1.39 | 1.29 | 1.38 | 1.38 | 1.54 | 1.41 | 1.38 | 1.33 |
|  | UCC | 1.33 | 1.26 | 1.63 | 1.3 | 1.31 | 1.67 | 1.71 | 1.24 | 1.69 | 1.62 | 1.25 | 1.32 | 1.33 | 1.44 |
|  | UCA | 1.03 | 1.29 | 1.28 | 1.03 | 1.13 | 1.31 | 1.18 | 1.28 | 1.2 | 1.14 | 1.12 | 1.19 | 1.32 | 1.28 |
|  | UCG | 0.69 | 0.59 | 0.37 | 0.68 | 0.64 | 0.32 | 0.48 | 0.62 | 0.46 | 0.4 | 0.59 | 0.64 | 0.6 | 0.52 |
| Pro | CCU | 1.47 | 1.46 | 1.13 | 1.47 | 1.48 | 1.09 | 1.14 | 1.46 | 1.1 | 1.12 | 1.59 | 1.66 | 1.61 | 1.55 |
|  | CCC | 1.21 | 1.24 | 1.7 | 1.23 | 1.24 | 1.72 | 1.68 | 1.23 | 1.69 | 1.59 | 1.2 | 1.16 | 1.23 | 1.21 |
|  | CCA | 0.97 | 0.97 | 0.85 | 0.97 | 0.95 | 0.86 | 0.83 | 0.98 | 0.84 | 0.91 | 0.89 | 0.9 | 0.91 | 0.88 |
|  | CCG | 0.35 | 0.33 | 0.32 | 0.32 | 0.32 | 0.33 | 0.36 | 0.33 | 0.37 | 0.38 | 0.31 | 0.28 | 0.25 | 0.35 |
| Thr | ACU | 1.19 | 1.08 | 0.8 | 1.19 | 1.11 | 0.81 | 0.85 | 1.07 | 0.89 | 0.94 | 1.16 | 1.21 | 1.08 | 1.12 |
|  | ACC | 1.22 | 1.28 | 1.61 | 1.28 | 1.19 | 1.6 | 1.53 | 1.26 | 1.55 | 1.61 | 1.12 | 1.13 | 1.19 | 1.28 |
|  | ACA | 1.15 | 1.17 | 1.31 | 1.08 | 1.21 | 1.3 | 1.24 | 1.16 | 1.19 | 1.14 | 1.31 | 1.23 | 1.32 | 1.21 |
|  | ACG | 0.44 | 0.47 | 0.29 | 0.45 | 0.49 | 0.28 | 0.39 | 0.51 | 0.36 | 0.31 | 0.4 | 0.43 | 0.41 | 0.39 |
| Ala | GCU | 0.99 | 0.95 | 0.74 | 0.86 | 1 | 0.7 | 0.76 | 0.94 | 0.75 | 0.71 | 1.01 | 0.96 | 0.98 | 1.13 |
|  | GCC | 1.86 | 1.9 | 1.81 | 1.97 | 1.95 | 1.85 | 1.81 | 1.86 | 1.83 | 1.91 | 1.68 | 1.74 | 1.68 | 1.62 |
|  | GCA | 1.04 | 0.95 | 1.19 | 1.09 | 0.95 | 1.2 | 1.17 | 0.97 | 1.22 | 1.16 | 1.12 | 1.17 | 1.15 | 1.07 |
|  | GCG | 0.11 | 0.2 | 0.26 | 0.09 | 0.1 | 0.25 | 0.26 | 0.22 | 0.21 | 0.22 | 0.19 | 0.13 | 0.2 | 0.19 |
| Tyr | UAU | 1.05 | 1.06 | 0.94 | 1.08 | 1.1 | 0.92 | 0.98 | 1.08 | 0.94 | 0.98 | 0.91 | 1.02 | 0.99 | 0.96 |
|  | UAC | 0.95 | 0.94 | 1.06 | 0.92 | 0.9 | 1.08 | 1.02 | 0.92 | 1.06 | 1.02 | 1.09 | 0.98 | 1.01 | 1.04 |
| TER | UAA | 1.1 | 1.13 | 0.88 | 1.1 | 1.05 | 0.9 | 0.95 | 1.15 | 0.98 | 0.9 | 1.02 | 0.91 | 0.89 | 1.05 |
|  | UAG | 1.14 | 1.06 | 0.73 | 1.11 | 1.12 | 0.75 | 0.88 | 1.02 | 0.82 | 0.78 | 0.95 | 1.1 | 1.1 | 0.96 |
| His | CAU | 1.04 | 1.01 | 0.74 | 1.01 | 1.12 | 0.77 | 0.93 | 1.01 | 0.93 | 0.77 | 0.87 | 0.91 | 0.94 | 0.91 |
|  | CAC | 0.96 | 0.99 | 1.26 | 0.99 | 0.88 | 1.23 | 1.07 | 0.99 | 1.07 | 1.23 | 1.13 | 1.09 | 1.06 | 1.09 |
| Gln | CAA | 1.31 | 1.3 | 1.35 | 1.33 | 1.29 | 1.34 | 1.38 | 1.32 | 1.37 | 1.3 | 1.26 | 1.33 | 1.34 | 1.21 |
|  | CAG | 0.69 | 0.7 | 0.65 | 0.67 | 0.71 | 0.66 | 0.62 | 0.68 | 0.63 | 0.7 | 0.74 | 0.67 | 0.66 | 0.79 |
| Asn | AAU | 1.03 | 1.03 | 0.79 | 1.04 | 0.99 | 0.82 | 0.86 | 1.04 | 0.84 | 0.82 | 0.88 | 0.9 | 0.88 | 0.91 |
|  | AAC | 0.97 | 0.97 | 1.21 | 0.96 | 1.01 | 1.18 | 1.14 | 0.96 | 1.16 | 1.18 | 1.12 | 1.1 | 1.12 | 1.09 |
| Lys | AAA | 1.41 | 1.45 | 1.62 | 1.39 | 1.41 | 1.58 | 1.47 | 1.45 | 1.5 | 1.47 | 1.48 | 1.51 | 1.49 | 1.46 |
|  | AAG | 0.59 | 0.55 | 0.38 | 0.61 | 0.59 | 0.42 | 0.53 | 0.55 | 0.5 | 0.53 | 0.52 | 0.49 | 0.51 | 0.54 |
| Asp | GAU | 0.93 | 1.08 | 0.84 | 1.13 | 1.05 | 0.79 | 0.9 | 1.08 | 0.86 | 0.99 | 0.92 | 0.88 | 0.82 | 1.07 |
|  | GAC | 1.07 | 0.92 | 1.16 | 0.87 | 0.95 | 1.21 | 1.1 | 0.92 | 1.14 | 1.01 | 1.08 | 1.12 | 1.18 | 0.93 |
| Glu | GAA | 1.21 | 1.09 | 1.33 | 1.18 | 1.14 | 1.32 | 1.27 | 1.08 | 1.29 | 1.2 | 1.25 | 1.32 | 1.24 | 1.32 |
|  | GAG | 0.79 | 0.91 | 0.67 | 0.82 | 0.86 | 0.68 | 0.73 | 0.92 | 0.71 | 0.8 | 0.75 | 0.68 | 0.76 | 0.68 |
| Cys | UGU | 0.95 | 1.02 | 0.77 | 0.96 | 1 | 0.7 | 0.68 | 1.01 | 0.76 | 0.75 | 0.78 | 0.98 | 1 | 0.81 |
|  | UGC | 1.05 | 0.98 | 1.23 | 1.04 | 1 | 1.3 | 1.32 | 0.99 | 1.24 | 1.25 | 1.22 | 1.02 | 1 | 1.19 |
| TER | UGA | 0.76 | 0.81 | 1.39 | 0.79 | 0.83 | 1.35 | 1.17 | 0.83 | 1.2 | 1.33 | 1.04 | 0.99 | 1.01 | 0.98 |
| Trp | UGG | 1 | 1 | 1 | 1 | 1 | 1 | 1 | 1 | 1 | 1 | 1 | 1 | 1 | 1 |
| Arg | CGU | 0.74 | 0.79 | 0.75 | 0.78 | 0.71 | 0.82 | 0.87 | 0.83 | 0.82 | 0.67 | 1.02 | 0.75 | 0.79 | 0.68 |
|  | CGC | 0.74 | 0.84 | 0.67 | 0.78 | 0.93 | 0.66 | 0.62 | 0.88 | 0.82 | 0.89 | 0.83 | 0.96 | 0.96 | 1.09 |
|  | CGA | 0.79 | 0.76 | 1.74 | 0.81 | 0.77 | 1.68 | 1.53 | 0.75 | 1.42 | 1.37 | 1.17 | 1.06 | 1.23 | 1.16 |
|  | CGG | 0.95 | 0.87 | 0.83 | 1 | 0.93 | 0.86 | 1.02 | 0.86 | 1.01 | 1.15 | 0.6 | 0.79 | 0.65 | 0.99 |
| Ser | AGU | 0.66 | 0.64 | 0.48 | 0.63 | 0.59 | 0.49 | 0.45 | 0.63 | 0.41 | 0.54 | 0.55 | 0.57 | 0.52 | 0.6 |
|  | AGC | 0.95 | 0.93 | 0.9 | 1.02 | 0.89 | 0.89 | 0.79 | 0.94 | 0.86 | 0.92 | 0.95 | 0.87 | 0.85 | 0.84 |
| Arg | AGA | 1.36 | 1.29 | 1.11 | 1.31 | 1.32 | 1.09 | 1.02 | 1.24 | 1.04 | 0.96 | 0.94 | 0.99 | 0.99 | 0.72 |
|  | AGG | 1.42 | 1.46 | 0.91 | 1.33 | 1.35 | 0.9 | 0.95 | 1.44 | 0.89 | 0.96 | 1.43 | 1.44 | 1.37 | 1.36 |
| Gly | GGU | 0.8 | 0.8 | 0.53 | 0.82 | 0.75 | 0.57 | 0.59 | 0.83 | 0.57 | 0.4 | 0.72 | 0.75 | 0.75 | 0.62 |
|  | GGC | 1.04 | 1.14 | 1.13 | 0.94 | 1.21 | 1.09 | 1.09 | 1.13 | 1.1 | 1.28 | 0.95 | 0.93 | 0.97 | 1.08 |
|  | GGA | 1.28 | 0.99 | 1.35 | 1.25 | 0.89 | 1.36 | 1.23 | 0.98 | 1.31 | 1.16 | 1.28 | 1.42 | 1.13 | 1.34 |
|  | GGG | 0.88 | 1.07 | 0.98 | 0.98 | 1.15 | 0.98 | 1.09 | 1.06 | 1.01 | 1.16 | 1.05 | 0.9 | 1.15 | 0.95 |
