# Supplementary material for: Comparative Mitogenome Analyses Uncover Mitogenome Features and Phylogenetic Implications of the Rabbitfishes (Perciformes: Siganidae)
Source: Ecol Evol. 2026 Aug 14;16(8):e74188. doi: 10.1002/ece3.74188 (PMC13476152; doi:10.1002/ece3.74188)
Supplement: Supplementary file 9 [file ECE3-16-e74188-s009.docx]

Figure S3. Representative putative secondary structures of the 22 mitochondrial tRNAs in Siganidae, shown using *Siganus argenteus* as an example.
