# Supplementary material for: Comparative Mitogenome Analyses Uncover Mitogenome Features and Phylogenetic Implications of the Rabbitfishes (Perciformes: Siganidae)
Source: Ecol Evol. 2026 Aug 14;16(8):e74188. doi: 10.1002/ece3.74188 (PMC13476152; doi:10.1002/ece3.74188)
Supplement: Supplementary file 10 [file ECE3-16-e74188-s010.docx]

Table S3. Nucleotide composition and compositional skew of complete mitogenomes, mitochondrial genomic components, and the 13 protein-coding genes in Siganidae.

|  | Length (bp) | G% | A% | T/U% | C% | GC% | AT% | AT-skew (A-T)/(A+T) | GC-skew (G-C)/(G+C) |
| --- | --- | --- | --- | --- | --- | --- | --- | --- | --- |
| Genome | | | | | | | | | |
| Siganus virgatus | 16505 | 15.75 | 29.25 | 25.77 | 29.24 | 44.99 | 55.01 | 0.063249727 | -0.29984441 |
| Siganus vulpinus | 16505 | 15.96 | 28.94 | 25.48 | 29.62 | 45.58 | 54.42 | 0.063579566 | -0.299692848 |
| Siganus canaliculatus | 16492 | 16.68 | 28.15 | 25.43 | 29.74 | 46.42 | 53.58 | 0.050765211 | -0.281344248 |
| Siganus guttatus | 16505 | 15.7 | 29.26 | 25.74 | 29.3 | 45 | 55 | 0.064 | -0.302222222 |
| Siganus puellus | 16504 | 16.34 | 28.57 | 25.55 | 29.54 | 45.88 | 54.12 | 0.055801922 | -0.287707062 |
| Siganus fuscescens | 16491 | 16.76 | 28.05 | 25.4 | 29.79 | 46.55 | 53.45 | 0.049579046 | -0.279914071 |
| Siganus sutor | 16497 | 16.75 | 28.04 | 25.39 | 29.82 | 46.57 | 53.43 | 0.049597604 | -0.280652781 |
| Siganus unimaculatus | 16505 | 16.03 | 28.87 | 25.45 | 29.65 | 45.68 | 54.32 | 0.062960236 | -0.298161121 |
| Siganus rivulatus | 16495 | 16.79 | 27.97 | 25.4 | 29.85 | 46.64 | 53.37 | 0.048154394 | -0.280017153 |
| Siganus luridus | 16495 | 17.02 | 27.78 | 25.33 | 29.86 | 46.89 | 53.11 | 0.046130672 | -0.273890785 |
| Siganus argenteus | 16495 | 16.69 | 28.06 | 25.86 | 29.38 | 46.07 | 53.93 | 0.040801187 | -0.275450402 |
| Siganus corallinus | 16510 | 15.83 | 29.18 | 25.72 | 29.28 | 45.11 | 54.89 | 0.063023679 | -0.298160053 |
| Siganus punctatus | 16505 | 16.06 | 28.78 | 25.48 | 29.68 | 45.74 | 54.26 | 0.060818282 | -0.297770004 |
| Siganus spinus | 16496 | 16.87 | 28.13 | 25.56 | 29.44 | 46.31 | 53.69 | 0.047867387 | -0.271431656 |
| Protein_coding_genes | | | | | | | | | |
| Siganus virgatus | 11427 | 15.06 | 27.08 | 27.65 | 30.22 | 45.28 | 54.72 | -0.010414763 | -0.334805654 |
| Siganus vulpinus | 11427 | 15.36 | 26.63 | 27.31 | 30.7 | 46.06 | 53.94 | -0.0126066 | -0.333043856 |
| Siganus canaliculatus | 11425 | 16.11 | 25.72 | 27.17 | 31 | 47.11 | 52.89 | -0.02741539 | -0.316068775 |
| Siganus guttatus | 11427 | 15.07 | 27.03 | 27.68 | 30.22 | 45.29 | 54.71 | -0.011880826 | -0.33451093 |
| Siganus puellus | 11427 | 15.86 | 26.14 | 27.39 | 30.61 | 46.47 | 53.53 | -0.023351392 | -0.317409081 |
| Siganus fuscescens | 11425 | 16.24 | 25.58 | 27.15 | 31.03 | 47.27 | 52.73 | -0.029774322 | -0.312883436 |
| Siganus sutor | 11428 | 16.21 | 25.6 | 27.08 | 31.11 | 47.32 | 52.68 | -0.028094153 | -0.31487743 |
| Siganus unimaculatus | 11427 | 15.45 | 26.54 | 27.29 | 30.73 | 46.18 | 53.83 | -0.013932751 | -0.330879168 |
| Siganus rivulatus | 11428 | 16.28 | 25.5 | 27.04 | 31.18 | 47.46 | 52.54 | -0.029311001 | -0.313948588 |
| Siganus luridus | 11428 | 16.48 | 25.25 | 26.88 | 31.39 | 47.87 | 52.13 | -0.031267984 | -0.311468561 |
| Siganus argenteus | 11433 | 16.28 | 25.55 | 27.8 | 30.38 | 46.65 | 53.35 | -0.042174321 | -0.302186027 |
| Siganus corallinus | 11433 | 15.11 | 27.05 | 27.58 | 30.26 | 45.37 | 54.63 | -0.009701629 | -0.333921093 |
| Siganus punctatus | 11433 | 15.59 | 26.36 | 27.38 | 30.67 | 46.26 | 53.74 | -0.018980275 | -0.325983571 |
| Siganus spinus | 11433 | 16.35 | 25.58 | 27.48 | 30.59 | 46.93 | 53.07 | -0.035808519 | -0.303365999 |
| tRNA | | | | | | | | | |
| Siganus virgatus | 1556 | 23.65 | 27.96 | 27.19 | 21.21 | 44.86 | 55.14 | 0.013961922 | 0.05439144 |
| Siganus vulpinus | 1556 | 19.73 | 30.59 | 24.68 | 25 | 44.73 | 55.27 | 0.106929618 | -0.117818019 |
| Siganus canaliculatus | 1555 | 23.99 | 27.85 | 26.82 | 21.35 | 45.34 | 54.67 | 0.018840315 | 0.058226731 |
| Siganus guttatus | 1556 | 19.86 | 30.59 | 24.61 | 24.94 | 44.8 | 55.2 | 0.108333333 | -0.113392857 |
| Siganus puellus | 1556 | 19.86 | 30.53 | 24.61 | 25 | 44.86 | 55.14 | 0.107363076 | -0.114578689 |
| Siganus fuscescens | 1556 | 20.5 | 30.08 | 24.23 | 25.19 | 45.69 | 54.31 | 0.10771497 | -0.102648282 |
| Siganus sutor | 1556 | 20.57 | 29.88 | 24.49 | 25.06 | 45.63 | 54.37 | 0.099135553 | -0.098400175 |
| Siganus unimaculatus | 1556 | 19.73 | 30.59 | 24.68 | 25 | 44.73 | 55.27 | 0.106929618 | -0.117818019 |
| Siganus rivulatus | 1556 | 20.37 | 30.14 | 24.42 | 25.06 | 45.43 | 54.56 | 0.10483871 | -0.103235747 |
| Siganus luridus | 1555 | 20.32 | 30.16 | 24.5 | 25.02 | 45.34 | 54.66 | 0.103549213 | -0.103661226 |
| Siganus argenteus | 1556 | 23.91 | 27.76 | 27.06 | 21.27 | 45.18 | 54.82 | 0.012769062 | 0.058432935 |
| Siganus corallinus | 1556 | 23.39 | 28.21 | 27.19 | 21.21 | 44.6 | 55.4 | 0.018411552 | 0.048878924 |
| Siganus punctatus | 1556 | 23.71 | 27.96 | 27.06 | 21.27 | 44.99 | 55.01 | 0.016357688 | 0.054246332 |
| Siganus spinus | 1555 | 23.79 | 27.97 | 26.88 | 21.35 | 45.14 | 54.86 | 0.019872379 | 0.054054054 |
| rRNA | | | | | | | | | |
| Siganus virgatus | 2642 | 20.55 | 32.44 | 21.16 | 25.85 | 46.4 | 53.6 | 0.210447761 | -0.114224138 |
| Siganus vulpinus | 2641 | 20.6 | 32.53 | 20.83 | 26.05 | 46.65 | 53.36 | 0.219265367 | -0.116827438 |
| Siganus canaliculatus | 2639 | 20.88 | 32.25 | 21.22 | 25.65 | 46.53 | 53.47 | 0.206283898 | -0.102514507 |
| Siganus guttatus | 2641 | 20.56 | 32.41 | 21.05 | 25.98 | 46.54 | 53.46 | 0.212495324 | -0.11645896 |
| Siganus puellus | 2643 | 20.73 | 32.35 | 21.23 | 25.69 | 46.42 | 53.58 | 0.207540127 | -0.106850495 |
| Siganus fuscescens | 2638 | 20.92 | 32.18 | 21.19 | 25.7 | 46.62 | 53.37 | 0.205920929 | -0.102531103 |
| Siganus sutor | 2639 | 20.8 | 32.25 | 21.18 | 25.77 | 46.57 | 53.43 | 0.207186974 | -0.106721065 |
| Siganus unimaculatus | 2641 | 20.64 | 32.49 | 20.86 | 26.01 | 46.65 | 53.35 | 0.217994377 | -0.11511254 |
| Siganus rivulatus | 2638 | 20.74 | 32.3 | 21.08 | 25.89 | 46.63 | 53.38 | 0.210191083 | -0.11044392 |
| Siganus luridus | 2638 | 20.7 | 32.34 | 21.34 | 25.63 | 46.33 | 53.68 | 0.204918033 | -0.106410533 |
| Siganus argenteus | 2599 | 21.12 | 32.13 | 21.32 | 25.43 | 46.56 | 53.44 | 0.202245089 | -0.092588614 |
| Siganus corallinus | 2603 | 20.86 | 32.31 | 21.28 | 25.55 | 46.41 | 53.59 | 0.205821982 | -0.101055807 |
| Siganus punctatus | 2603 | 20.63 | 32.58 | 21.28 | 25.51 | 46.14 | 53.86 | 0.209803193 | -0.105765063 |
| Siganus spinus | 2600 | 21 | 32.19 | 21.35 | 25.46 | 46.46 | 53.54 | 0.202465446 | -0.095996556 |

| **PCG** | **Species** | **Length (bp)** | **G%** | **A%** | **T/U%** | **C%** | **GC%** | **AT%** | **AT-skew (A-T)/(A+T)** | **GC-skew (G-C)/(G+C)** |
| --- | --- | --- | --- | --- | --- | --- | --- | --- | --- | --- |
| nad1 | Siganus virgatus | 975 | 14.05 | 26.56 | 25.95 | 33.44 | 47.49 | 52.51 | 0.011616835 | -0.408296483 |
| nad1 | Siganus vulpinus | 975 | 13.95 | 26.56 | 26.87 | 32.62 | 46.57 | 53.43 | -0.005801984 | -0.400901868 |
| nad1 | Siganus canaliculatus | 975 | 14.67 | 25.85 | 26.56 | 32.92 | 47.59 | 52.41 | -0.013547033 | -0.383483925 |
| nad1 | Siganus guttatus | 975 | 13.74 | 26.56 | 26.15 | 33.54 | 47.28 | 52.72 | 0.00777841 | -0.418781726 |
| nad1 | Siganus puellus | 975 | 14.77 | 25.85 | 27.69 | 31.69 | 46.46 | 53.54 | -0.034366829 | -0.364184245 |
| nad1 | Siganus fuscescens | 975 | 15.28 | 25.23 | 26.77 | 32.72 | 48 | 52 | -0.029615385 | -0.363333333 |
| nad1 | Siganus sutor | 975 | 15.79 | 24.82 | 25.85 | 33.54 | 49.33 | 50.67 | -0.02032761 | -0.35982161 |
| nad1 | Siganus unimaculatus | 975 | 13.95 | 26.67 | 26.87 | 32.51 | 46.46 | 53.54 | -0.003735525 | -0.399483427 |
| nad1 | Siganus rivulatus | 975 | 14.97 | 25.54 | 26.15 | 33.33 | 48.3 | 51.69 | -0.011801122 | -0.380124224 |
| nad1 | Siganus luridus | 975 | 15.9 | 23.49 | 26.56 | 34.05 | 49.95 | 50.05 | -0.061338661 | -0.363363363 |
| nad1 | Siganus argenteus | 975 | 16.1 | 24.72 | 26.36 | 32.82 | 48.92 | 51.08 | -0.0321065 | -0.341782502 |
| nad1 | Siganus corallinus | 975 | 13.85 | 26.67 | 26.15 | 33.33 | 47.18 | 52.82 | 0.009844756 | -0.412886816 |
| nad1 | Siganus punctatus | 975 | 14.56 | 25.85 | 27.08 | 32.51 | 47.07 | 52.93 | -0.023238239 | -0.38134693 |
| nad1 | Siganus spinus | 975 | 14.77 | 26.05 | 27.69 | 31.69 | 46.46 | 53.74 | -0.030517306 | -0.364184245 |
| nad2 | Siganus virgatus | 1045 | 11.39 | 28.9 | 24.78 | 34.93 | 46.32 | 53.68 | 0.076751118 | -0.5082038 |
| nad2 | Siganus vulpinus | 1045 | 11.67 | 29.57 | 24.4 | 34.35 | 46.03 | 53.97 | 0.09579396 | -0.492829205 |
| nad2 | Siganus canaliculatus | 1045 | 12.63 | 28.13 | 23.73 | 35.5 | 48.13 | 51.87 | 0.08484381 | -0.475171411 |
| nad2 | Siganus guttatus | 1045 | 11.1 | 29.28 | 24.11 | 35.5 | 46.6 | 53.4 | 0.096834613 | -0.52360515 |
| nad2 | Siganus puellus | 1045 | 11.96 | 29.57 | 23.35 | 35.12 | 47.08 | 52.92 | 0.117535903 | -0.491928632 |
| nad2 | Siganus fuscescens | 1045 | 12.73 | 28.04 | 23.64 | 35.6 | 48.33 | 51.68 | 0.085139319 | -0.473205049 |
| nad2 | Siganus sutor | 1045 | 12.34 | 28.33 | 24.31 | 35.02 | 47.36 | 52.64 | 0.076367781 | -0.478885135 |
| nad2 | Siganus unimaculatus | 1045 | 11.96 | 29.28 | 24.5 | 34.26 | 46.22 | 53.78 | 0.088880625 | -0.482475119 |
| nad2 | Siganus rivulatus | 1045 | 13.01 | 27.46 | 24.02 | 35.5 | 48.51 | 51.48 | 0.066822067 | -0.463615749 |
| nad2 | Siganus luridus | 1045 | 12.63 | 27.66 | 24.88 | 34.83 | 47.46 | 52.54 | 0.052912067 | -0.467762326 |
| nad2 | Siganus argenteus | 1047 | 13.28 | 27.13 | 24.93 | 34.67 | 47.95 | 52.06 | 0.042258932 | -0.446089677 |
| nad2 | Siganus corallinus | 1047 | 11.37 | 28.94 | 24.36 | 35.34 | 46.71 | 53.3 | 0.085928705 | -0.513166346 |
| nad2 | Siganus punctatus | 1047 | 11.17 | 30.09 | 24.55 | 34.19 | 45.36 | 54.64 | 0.101390922 | -0.507495591 |
| nad2 | Siganus spinus | 1047 | 13.94 | 27.03 | 24.36 | 34.67 | 48.61 | 51.39 | 0.051955633 | -0.426455462 |
| cox1 | Siganus virgatus | 1551 | 17.73 | 25.6 | 30.05 | 26.63 | 44.36 | 55.64 | -0.079964061 | -0.200631199 |
| cox1 | Siganus vulpinus | 1551 | 18.31 | 24.89 | 29.46 | 27.34 | 45.65 | 54.35 | -0.084084637 | -0.197809419 |
| cox1 | Siganus canaliculatus | 1551 | 18.63 | 24.44 | 29.46 | 27.47 | 46.1 | 53.9 | -0.093135436 | -0.19175705 |
| cox1 | Siganus guttatus | 1551 | 17.67 | 25.73 | 30.17 | 26.43 | 44.1 | 55.9 | -0.079427549 | -0.198639456 |
| cox1 | Siganus puellus | 1551 | 19.15 | 24.24 | 30.05 | 26.56 | 45.71 | 54.29 | -0.107017867 | -0.162108948 |
| cox1 | Siganus fuscescens | 1551 | 18.63 | 24.44 | 29.46 | 27.47 | 46.1 | 53.9 | -0.093135436 | -0.19175705 |
| cox1 | Siganus sutor | 1551 | 18.57 | 24.44 | 29.14 | 27.85 | 46.42 | 53.58 | -0.087719298 | -0.19991383 |
| cox1 | Siganus unimaculatus | 1551 | 18.31 | 24.89 | 29.34 | 27.47 | 45.78 | 54.23 | -0.082057902 | -0.200087374 |
| cox1 | Siganus rivulatus | 1551 | 18.63 | 24.11 | 29.4 | 27.85 | 46.48 | 53.51 | -0.098860026 | -0.198364888 |
| cox1 | Siganus luridus | 1551 | 18.5 | 24.5 | 28.5 | 28.5 | 47 | 53 | -0.075471698 | -0.212765957 |
| cox1 | Siganus argenteus | 1551 | 18.44 | 24.89 | 29.27 | 27.4 | 45.84 | 54.16 | -0.080871492 | -0.195462478 |
| cox1 | Siganus corallinus | 1551 | 18.18 | 25.21 | 29.85 | 26.76 | 44.94 | 55.06 | -0.084271704 | -0.190921228 |
| cox1 | Siganus punctatus | 1551 | 18.76 | 24.44 | 29.34 | 27.47 | 46.23 | 53.78 | -0.091111938 | -0.188405797 |
| cox1 | Siganus spinus | 1551 | 18.57 | 24.44 | 29.53 | 27.47 | 46.04 | 53.97 | -0.094311655 | -0.193310165 |
| cox2 | Siganus virgatus | 691 | 15.34 | 30.82 | 28.65 | 25.18 | 40.52 | 59.48 | 0.036488986 | -0.24284304 |
| cox2 | Siganus vulpinus | 691 | 15.34 | 30.82 | 28.08 | 25.76 | 41.1 | 58.9 | 0.046519525 | -0.253527981 |
| cox2 | Siganus canaliculatus | 691 | 16.21 | 28.51 | 28.94 | 26.34 | 42.55 | 57.45 | -0.007484769 | -0.238072855 |
| cox2 | Siganus guttatus | 691 | 15.34 | 30.97 | 28.65 | 25.04 | 40.38 | 59.62 | 0.038913116 | -0.24021793 |
| cox2 | Siganus puellus | 691 | 15.2 | 30.97 | 28.08 | 25.76 | 40.96 | 59.04 | 0.048941575 | -0.2578125 |
| cox2 | Siganus fuscescens | 691 | 15.92 | 28.65 | 28.22 | 27.21 | 43.13 | 56.87 | 0.007561104 | -0.261766752 |
| cox2 | Siganus sutor | 691 | 15.92 | 28.94 | 27.64 | 27.5 | 43.42 | 56.58 | 0.022976317 | -0.266697374 |
| cox2 | Siganus unimaculatus | 691 | 15.63 | 30.54 | 28.08 | 25.76 | 41.39 | 58.62 | 0.0419652 | -0.244745108 |
| cox2 | Siganus rivulatus | 691 | 16.5 | 28.36 | 28.22 | 26.92 | 43.42 | 56.58 | 0.002474373 | -0.239981575 |
| cox2 | Siganus luridus | 691 | 16.06 | 29.09 | 27.93 | 26.92 | 42.98 | 57.02 | 0.020343739 | -0.252675663 |
| cox2 | Siganus argenteus | 691 | 15.48 | 31.11 | 27.35 | 26.05 | 41.53 | 58.46 | 0.064317482 | -0.254514809 |
| cox2 | Siganus corallinus | 691 | 15.34 | 30.82 | 29.09 | 24.75 | 40.09 | 59.91 | 0.028876648 | -0.234721876 |
| cox2 | Siganus punctatus | 691 | 15.34 | 30.82 | 27.93 | 25.9 | 41.24 | 58.75 | 0.049191489 | -0.256062076 |
| cox2 | Siganus spinus | 691 | 16.06 | 28.65 | 29.09 | 26.19 | 42.25 | 57.74 | -0.007620367 | -0.239763314 |
| atp8 | Siganus virgatus | 168 | 10.71 | 31.55 | 25.6 | 32.14 | 42.85 | 57.15 | 0.104111986 | -0.500116686 |
| atp8 | Siganus vulpinus | 168 | 10.71 | 30.95 | 22.62 | 35.71 | 46.43 | 53.57 | 0.15549748 | -0.538560965 |
| atp8 | Siganus canaliculatus | 168 | 11.31 | 31.55 | 22.02 | 35.12 | 46.43 | 53.57 | 0.177898077 | -0.51281499 |
| atp8 | Siganus guttatus | 168 | 10.71 | 31.55 | 25.6 | 32.14 | 42.85 | 57.15 | 0.104111986 | -0.500116686 |
| atp8 | Siganus puellus | 168 | 11.9 | 28.57 | 25 | 34.52 | 46.43 | 53.57 | 0.066641777 | -0.487289961 |
| atp8 | Siganus fuscescens | 168 | 11.31 | 31.55 | 22.02 | 35.12 | 46.43 | 53.57 | 0.177898077 | -0.51281499 |
| atp8 | Siganus sutor | 168 | 11.31 | 31.55 | 23.21 | 33.93 | 45.24 | 54.76 | 0.15230095 | -0.5 |
| atp8 | Siganus unimaculatus | 168 | 10.71 | 30.95 | 22.02 | 36.31 | 47.02 | 52.97 | 0.168585992 | -0.544449171 |
| atp8 | Siganus rivulatus | 168 | 11.9 | 30.95 | 22.62 | 34.52 | 46.42 | 53.57 | 0.15549748 | -0.487289961 |
| atp8 | Siganus luridus | 168 | 10.71 | 30.95 | 22.02 | 36.31 | 47.02 | 52.97 | 0.168585992 | -0.544449171 |
| atp8 | Siganus argenteus | 168 | 10.71 | 31.55 | 23.21 | 34.52 | 45.23 | 54.76 | 0.15230095 | -0.526420517 |
| atp8 | Siganus corallinus | 168 | 10.12 | 32.14 | 25.6 | 32.14 | 42.26 | 57.74 | 0.113266366 | -0.521060104 |
| atp8 | Siganus punctatus | 168 | 10.71 | 30.36 | 22.62 | 36.31 | 47.02 | 52.98 | 0.146092865 | -0.544449171 |
| atp8 | Siganus spinus | 168 | 11.31 | 29.76 | 23.81 | 35.12 | 46.43 | 53.57 | 0.111069629 | -0.51281499 |
| atp6 | Siganus virgatus | 683 | 12.01 | 26.65 | 27.38 | 33.97 | 45.97 | 54.03 | -0.013511012 | -0.477598956 |
| atp6 | Siganus vulpinus | 683 | 12.45 | 26.21 | 27.38 | 33.97 | 46.41 | 53.59 | -0.021832431 | -0.463593279 |
| atp6 | Siganus canaliculatus | 682 | 13.34 | 25.51 | 26.98 | 34.16 | 47.51 | 52.49 | -0.028005334 | -0.438315789 |
| atp6 | Siganus guttatus | 683 | 12.01 | 26.65 | 28.11 | 33.24 | 45.24 | 54.76 | -0.026661797 | -0.469171271 |
| atp6 | Siganus puellus | 683 | 13.32 | 24.74 | 28.26 | 33.67 | 47 | 53 | -0.066415094 | -0.433070866 |
| atp6 | Siganus fuscescens | 683 | 13.34 | 25.37 | 27.27 | 34.02 | 47.36 | 52.64 | -0.036094225 | -0.436655405 |
| atp6 | Siganus sutor | 684 | 13.01 | 25.73 | 27.19 | 34.06 | 47.07 | 52.92 | -0.027588813 | -0.447206289 |
| atp6 | Siganus unimaculatus | 683 | 12.59 | 26.06 | 27.23 | 34.11 | 46.7 | 53.29 | -0.021955339 | -0.460813704 |
| atp6 | Siganus rivulatus | 684 | 13.6 | 25.29 | 27.34 | 33.77 | 47.37 | 52.63 | -0.038951169 | -0.425796918 |
| atp6 | Siganus luridus | 684 | 14.04 | 25.73 | 25.88 | 34.36 | 48.4 | 51.61 | -0.002906413 | -0.419834711 |
| atp6 | Siganus argenteus | 684 | 14.33 | 25 | 27.63 | 33.04 | 47.37 | 52.63 | -0.049971499 | -0.394975723 |
| atp6 | Siganus corallinus | 684 | 11.84 | 26.75 | 27.49 | 33.92 | 45.76 | 54.24 | -0.013643068 | -0.482517483 |
| atp6 | Siganus punctatus | 684 | 12.28 | 25.88 | 28.22 | 33.63 | 45.91 | 54.1 | -0.043253235 | -0.465040296 |
| atp6 | Siganus spinus | 684 | 13.6 | 24.85 | 27.63 | 33.92 | 47.52 | 52.48 | -0.052972561 | -0.427609428 |
| cox3 | Siganus virgatus | 785 | 16.43 | 27.01 | 25.48 | 31.08 | 47.51 | 52.49 | 0.029148409 | -0.308356136 |
| cox3 | Siganus vulpinus | 785 | 16.31 | 26.5 | 26.24 | 30.96 | 47.26 | 52.74 | 0.004929845 | -0.309921726 |
| cox3 | Siganus canaliculatus | 784 | 17.09 | 25.13 | 27.17 | 30.61 | 47.7 | 52.3 | -0.039005736 | -0.283438155 |
| cox3 | Siganus guttatus | 785 | 16.69 | 26.5 | 26.88 | 29.94 | 46.62 | 53.38 | -0.007118771 | -0.284151834 |
| cox3 | Siganus puellus | 785 | 16.69 | 26.24 | 26.24 | 30.83 | 47.52 | 52.48 | 0 | -0.297558923 |
| cox3 | Siganus fuscescens | 785 | 17.22 | 25 | 27.68 | 30.1 | 47.32 | 52.68 | -0.050873197 | -0.272189349 |
| cox3 | Siganus sutor | 785 | 17.2 | 25.22 | 27.13 | 30.45 | 47.65 | 52.35 | -0.036485196 | -0.278069255 |
| cox3 | Siganus unimaculatus | 785 | 16.31 | 26.5 | 26.37 | 30.83 | 47.14 | 52.87 | 0.002458861 | -0.308018668 |
| cox3 | Siganus rivulatus | 785 | 17.2 | 24.97 | 26.62 | 31.21 | 48.41 | 51.59 | -0.031982942 | -0.289403016 |
| cox3 | Siganus luridus | 785 | 17.45 | 25.22 | 27.39 | 29.94 | 47.39 | 52.61 | -0.041246911 | -0.263557713 |
| cox3 | Siganus argenteus | 786 | 16.92 | 25.83 | 27.23 | 30.03 | 46.95 | 53.06 | -0.026385224 | -0.279233227 |
| cox3 | Siganus corallinus | 786 | 16.41 | 27.1 | 25.95 | 30.53 | 46.94 | 53.05 | 0.021677663 | -0.300809544 |
| cox3 | Siganus punctatus | 786 | 17.18 | 26.08 | 25.7 | 31.04 | 48.22 | 51.78 | 0.007338741 | -0.287432601 |
| cox3 | Siganus spinus | 786 | 17.3 | 25.06 | 25.95 | 31.68 | 48.98 | 51.01 | -0.017447559 | -0.29358922 |
| nad3 | Siganus virgatus | 349 | 14.33 | 23.5 | 31.52 | 30.66 | 44.99 | 55.01 | -0.145765176 | -0.362969549 |
| nad3 | Siganus vulpinus | 349 | 15.47 | 21.78 | 32.09 | 30.66 | 46.13 | 53.87 | -0.191386672 | -0.329286798 |
| nad3 | Siganus canaliculatus | 349 | 15.76 | 21.2 | 31.81 | 31.23 | 46.99 | 53.01 | -0.200150915 | -0.329218983 |
| nad3 | Siganus guttatus | 349 | 15.76 | 21.78 | 32.66 | 29.8 | 45.56 | 54.44 | -0.199853049 | -0.308165057 |
| nad3 | Siganus puellus | 349 | 16.33 | 21.2 | 33.52 | 28.94 | 45.27 | 54.73 | -0.225146199 | -0.278550917 |
| nad3 | Siganus fuscescens | 349 | 15.47 | 21.49 | 32.09 | 30.95 | 46.42 | 53.58 | -0.197835013 | -0.33347695 |
| nad3 | Siganus sutor | 349 | 16.33 | 20.92 | 30.95 | 31.81 | 48.14 | 51.87 | -0.193368035 | -0.321562111 |
| nad3 | Siganus unimaculatus | 349 | 15.47 | 21.78 | 32.38 | 30.37 | 45.84 | 54.16 | -0.195716396 | -0.32504363 |
| nad3 | Siganus rivulatus | 349 | 15.47 | 21.78 | 31.52 | 31.23 | 46.7 | 53.3 | -0.182739212 | -0.337473233 |
| nad3 | Siganus luridus | 349 | 16.33 | 20.34 | 30.66 | 32.66 | 48.99 | 51 | -0.202352941 | -0.333333333 |
| nad3 | Siganus argenteus | 351 | 16.24 | 20.8 | 33.9 | 29.06 | 45.3 | 54.7 | -0.239488117 | -0.283002208 |
| nad3 | Siganus corallinus | 351 | 15.38 | 23.08 | 31.62 | 29.91 | 45.29 | 54.7 | -0.156124314 | -0.320821373 |
| nad3 | Siganus punctatus | 351 | 16.52 | 21.08 | 31.91 | 30.48 | 47 | 52.99 | -0.204378185 | -0.297021277 |
| nad3 | Siganus spinus | 351 | 17.09 | 20.51 | 30.48 | 31.91 | 49 | 50.99 | -0.195528535 | -0.30244898 |
| nad4l | Siganus virgatus | 297 | 15.15 | 23.23 | 26.94 | 34.68 | 49.83 | 50.17 | -0.073948575 | -0.391932571 |
| nad4l | Siganus vulpinus | 297 | 15.82 | 22.22 | 25.93 | 36.03 | 51.85 | 48.15 | -0.077050883 | -0.389778206 |
| nad4l | Siganus canaliculatus | 297 | 15.49 | 20.88 | 27.27 | 36.36 | 51.85 | 48.15 | -0.13271028 | -0.402507232 |
| nad4l | Siganus guttatus | 297 | 15.49 | 22.9 | 26.94 | 34.68 | 50.17 | 49.83 | -0.08105939 | -0.382499502 |
| nad4l | Siganus puellus | 297 | 15.15 | 22.9 | 26.94 | 35.02 | 50.17 | 49.83 | -0.08105939 | -0.396053418 |
| nad4l | Siganus fuscescens | 297 | 15.82 | 20.54 | 27.27 | 36.36 | 52.18 | 47.81 | -0.14076553 | -0.393637409 |
| nad4l | Siganus sutor | 297 | 16.16 | 19.87 | 26.94 | 37.04 | 53.2 | 46.81 | -0.151036103 | -0.392481203 |
| nad4l | Siganus unimaculatus | 297 | 15.82 | 22.22 | 25.93 | 36.03 | 51.85 | 48.15 | -0.077050883 | -0.389778206 |
| nad4l | Siganus rivulatus | 297 | 15.82 | 20.54 | 27.61 | 36.03 | 51.85 | 48.15 | -0.146832814 | -0.389778206 |
| nad4l | Siganus luridus | 297 | 16.84 | 19.53 | 26.6 | 37.04 | 53.88 | 46.13 | -0.153262519 | -0.374907201 |
| nad4l | Siganus argenteus | 297 | 16.5 | 20.88 | 28.96 | 33.67 | 50.17 | 49.84 | -0.16211878 | -0.342236396 |
| nad4l | Siganus corallinus | 297 | 15.49 | 22.9 | 27.27 | 34.34 | 49.83 | 50.17 | -0.087103847 | -0.378286173 |
| nad4l | Siganus punctatus | 297 | 15.82 | 22.56 | 26.6 | 35.02 | 50.84 | 49.16 | -0.082180635 | -0.377655389 |
| nad4l | Siganus spinus | 297 | 15.82 | 20.2 | 27.27 | 36.7 | 52.52 | 47.47 | -0.14893617 | -0.397562833 |
| nad4 | Siganus virgatus | 1381 | 13.54 | 28.17 | 27.08 | 31.21 | 44.75 | 55.25 | 0.019728507 | -0.394860335 |
| nad4 | Siganus vulpinus | 1381 | 14.27 | 27.3 | 25.56 | 32.87 | 47.14 | 52.86 | 0.03291714 | -0.394569368 |
| nad4 | Siganus canaliculatus | 1381 | 14.99 | 27.01 | 26.29 | 31.72 | 46.71 | 53.29 | 0.013508443 | -0.358167416 |
| nad4 | Siganus guttatus | 1381 | 13.47 | 28.24 | 26 | 32.3 | 45.76 | 54.24 | 0.041297935 | -0.41140485 |
| nad4 | Siganus puellus | 1381 | 15.06 | 26.5 | 25.27 | 33.16 | 48.23 | 51.77 | 0.023758934 | -0.37536292 |
| nad4 | Siganus fuscescens | 1381 | 15.28 | 26.65 | 26 | 32.08 | 47.36 | 52.65 | 0.012345679 | -0.35472973 |
| nad4 | Siganus sutor | 1381 | 15.57 | 26.57 | 25.2 | 32.66 | 48.23 | 51.77 | 0.026463203 | -0.354343769 |
| nad4 | Siganus unimaculatus | 1381 | 14.41 | 27.15 | 25.49 | 32.95 | 47.36 | 52.64 | 0.031534954 | -0.391469595 |
| nad4 | Siganus rivulatus | 1381 | 16 | 26.21 | 26.14 | 31.64 | 47.64 | 52.35 | 0.001337154 | -0.32829555 |
| nad4 | Siganus luridus | 1381 | 16.22 | 26 | 24.91 | 32.87 | 49.09 | 50.91 | 0.021410332 | -0.339172948 |
| nad4 | Siganus argenteus | 1381 | 15.13 | 26.5 | 26.21 | 32.15 | 47.28 | 52.71 | 0.005501802 | -0.35998308 |
| nad4 | Siganus corallinus | 1381 | 13.25 | 28.31 | 26.94 | 31.5 | 44.75 | 55.25 | 0.02479638 | -0.407821229 |
| nad4 | Siganus punctatus | 1381 | 14.12 | 27.37 | 25.05 | 33.45 | 47.57 | 52.42 | 0.044257917 | -0.406348539 |
| nad4 | Siganus spinus | 1381 | 15.57 | 26.57 | 25.71 | 32.15 | 47.72 | 52.28 | 0.016449885 | -0.34744342 |
| nad5 | Siganus virgatus | 1839 | 12.94 | 29.31 | 25.29 | 32.46 | 45.4 | 54.6 | 0.073626374 | -0.429955947 |
| nad5 | Siganus vulpinus | 1839 | 13.05 | 29.04 | 25.72 | 32.19 | 45.24 | 54.76 | 0.060628196 | -0.423076923 |
| nad5 | Siganus canaliculatus | 1839 | 14.25 | 27.95 | 25.01 | 32.79 | 47.04 | 52.96 | 0.055513595 | -0.394132653 |
| nad5 | Siganus guttatus | 1839 | 12.78 | 29.58 | 25.29 | 32.35 | 45.13 | 54.87 | 0.0781848 | -0.433636162 |
| nad5 | Siganus puellus | 1839 | 13 | 28.98 | 25.39 | 32.63 | 45.63 | 54.37 | 0.06602906 | -0.43019943 |
| nad5 | Siganus fuscescens | 1839 | 14.52 | 27.84 | 25.07 | 32.57 | 47.09 | 52.91 | 0.052353052 | -0.383308558 |
| nad5 | Siganus sutor | 1839 | 14.03 | 28.33 | 25.77 | 31.87 | 45.9 | 54.1 | 0.047319778 | -0.388671024 |
| nad5 | Siganus unimaculatus | 1839 | 13 | 29.09 | 25.72 | 32.19 | 45.19 | 54.81 | 0.06148513 | -0.424651472 |
| nad5 | Siganus rivulatus | 1839 | 13.92 | 28.55 | 24.96 | 32.57 | 46.49 | 53.51 | 0.067090264 | -0.40116154 |
| nad5 | Siganus luridus | 1839 | 14.19 | 28.11 | 25.94 | 31.76 | 45.95 | 54.05 | 0.040148011 | -0.382372144 |
| nad5 | Siganus argenteus | 1839 | 13.97 | 27.41 | 26.81 | 31.81 | 45.78 | 54.22 | 0.011066027 | -0.389689821 |
| nad5 | Siganus corallinus | 1839 | 13.1 | 29.26 | 25.01 | 32.63 | 45.73 | 54.27 | 0.078312143 | -0.427071944 |
| nad5 | Siganus punctatus | 1839 | 13.1 | 28.71 | 25.77 | 32.41 | 45.51 | 54.48 | 0.053964758 | -0.424302351 |
| nad5 | Siganus spinus | 1839 | 14.19 | 28.98 | 26.1 | 30.72 | 44.91 | 55.08 | 0.052287582 | -0.368069472 |
| nad6 | Siganus virgatus | 522 | 32.95 | 16.67 | 39.85 | 10.54 | 43.49 | 56.51 | -0.410120311 | 0.515290871 |
| nad6 | Siganus vulpinus | 522 | 34.1 | 15.33 | 39.08 | 11.49 | 45.59 | 54.41 | -0.436500643 | 0.495942093 |
| nad6 | Siganus canaliculatus | 522 | 34.1 | 14.94 | 37.93 | 13.03 | 47.13 | 52.87 | -0.434840174 | 0.44706132 |
| nad6 | Siganus guttatus | 522 | 33.72 | 15.71 | 40.04 | 10.54 | 44.25 | 55.75 | -0.436412556 | 0.523723452 |
| nad6 | Siganus puellus | 522 | 34.48 | 14.75 | 38.31 | 12.45 | 46.93 | 53.07 | -0.444025631 | 0.469422544 |
| nad6 | Siganus fuscescens | 522 | 33.91 | 15.13 | 38.31 | 12.64 | 46.55 | 53.44 | -0.433757485 | 0.456928034 |
| nad6 | Siganus sutor | 522 | 34.1 | 14.94 | 36.97 | 13.98 | 48.08 | 51.91 | -0.424388364 | 0.418469218 |
| nad6 | Siganus unimaculatus | 522 | 34.1 | 15.33 | 38.89 | 11.69 | 45.79 | 54.22 | -0.434526005 | 0.489408168 |
| nad6 | Siganus rivulatus | 522 | 33.72 | 15.33 | 36.78 | 14.18 | 47.9 | 52.11 | -0.411629246 | 0.407933194 |
| nad6 | Siganus luridus | 522 | 32.57 | 15.52 | 36.21 | 15.71 | 48.28 | 51.73 | -0.399961338 | 0.349212925 |
| nad6 | Siganus argenteus | 522 | 35.06 | 13.98 | 37.55 | 13.41 | 48.47 | 51.53 | -0.457403454 | 0.446668042 |
| nad6 | Siganus corallinus | 522 | 32.76 | 16.67 | 40.23 | 10.34 | 43.1 | 56.9 | -0.414059754 | 0.520185615 |
| nad6 | Siganus punctatus | 522 | 34.1 | 15.33 | 39.46 | 11.11 | 45.21 | 54.79 | -0.440408834 | 0.508515815 |
| nad6 | Siganus spinus | 522 | 33.33 | 13.79 | 37.93 | 14.94 | 48.27 | 51.72 | -0.466744006 | 0.380981976 |
| cob | Siganus virgatus | 1141 | 14.29 | 27.17 | 27.7 | 30.85 | 45.14 | 54.86 | -0.009659194 | -0.366858662 |
| cob | Siganus vulpinus | 1141 | 14.02 | 26.64 | 26.56 | 32.78 | 46.8 | 53.2 | 0.001503759 | -0.400854701 |
| cob | Siganus canaliculatus | 1141 | 15.16 | 25.5 | 25.68 | 33.65 | 48.82 | 51.18 | -0.003516999 | -0.378815816 |
| cob | Siganus guttatus | 1141 | 14.29 | 26.99 | 27.78 | 30.94 | 45.23 | 54.77 | -0.014423955 | -0.368118505 |
| cob | Siganus puellus | 1141 | 14.9 | 25.85 | 26.47 | 32.78 | 47.68 | 52.32 | -0.011850153 | -0.375 |
| cob | Siganus fuscescens | 1141 | 15.16 | 25.42 | 25.33 | 34.09 | 49.25 | 50.75 | 0.001773399 | -0.384365482 |
| cob | Siganus sutor | 1141 | 15.07 | 24.89 | 26.73 | 33.3 | 48.37 | 51.62 | -0.035645099 | -0.3768865 |
| cob | Siganus unimaculatus | 1141 | 14.29 | 26.29 | 26.56 | 32.87 | 47.16 | 52.85 | -0.005108798 | -0.393977947 |
| cob | Siganus rivulatus | 1141 | 15.25 | 24.8 | 25.85 | 34.09 | 49.34 | 50.65 | -0.020730503 | -0.381840292 |
| cob | Siganus luridus | 1141 | 16.21 | 24.1 | 25.68 | 34.01 | 50.22 | 49.78 | -0.031739654 | -0.354440462 |
| cob | Siganus argenteus | 1141 | 14.72 | 25.42 | 27.96 | 31.9 | 46.62 | 53.38 | -0.047583365 | -0.368511369 |
| cob | Siganus corallinus | 1141 | 14.29 | 27.26 | 27.17 | 31.29 | 45.58 | 54.43 | 0.0016535 | -0.372970601 |
| cob | Siganus punctatus | 1141 | 14.9 | 25.85 | 27.26 | 31.99 | 46.89 | 53.11 | -0.026548673 | -0.364470036 |
| cob | Siganus spinus | 1141 | 15.34 | 25.42 | 26.64 | 32.6 | 47.94 | 52.06 | -0.023434499 | -0.360033375 |
